# A Range Finding Protocol to Support Design for Transcriptomics Experimentation: Examples of *In-Vitro* and *In-Vivo* Murine UV Exposure

**DOI:** 10.1371/journal.pone.0097089

**Published:** 2014-05-13

**Authors:** Oskar Bruning, Wendy Rodenburg, Conny T. van Oostrom, Martijs J. Jonker, Mark de Jong, Rob J. Dekker, Han Rauwerda, Wim A. Ensink, Annemieke de Vries, Timo M. Breit

**Affiliations:** 1 RNA Biology & Applied Bioinformatics research group and MAD: Dutch Genomics Service & Support Provider, Swammerdam Institute for Life Sciences, Faculty of Science, University of Amsterdam, Amsterdam, the Netherlands; 2 Netherlands Bioinformatics Centre (NBIC), Nijmegen, the Netherlands; 3 Centre for Health Protection (GZB), National Institute of Public Health and the Environment (RIVM), Bilthoven, the Netherlands; University College London, United Kingdom

## Abstract

In transcriptomics research, design for experimentation by carefully considering biological, technological, practical and statistical aspects is very important, because the experimental design space is essentially limitless. Usually, the ranges of variable biological parameters of the design space are based on common practices and in turn on phenotypic endpoints. However, specific sub-cellular processes might only be partially reflected by phenotypic endpoints or outside the associated parameter range. Here, we provide a generic protocol for range finding in design for transcriptomics experimentation based on small-scale gene-expression experiments to help in the search for the right location in the design space by analyzing the activity of already known genes of relevant molecular mechanisms. Two examples illustrate the applicability: *in-vitro* UV-C exposure of mouse embryonic fibroblasts and *in-vivo* UV-B exposure of mouse skin. Our pragmatic approach is based on: framing a specific biological question and associated gene-set, performing a wide-ranged experiment without replication, eliminating potentially non-relevant genes, and determining the experimental ‘sweet spot’ by gene-set enrichment plus dose-response correlation analysis. Examination of many cellular processes that are related to UV response, such as DNA repair and cell-cycle arrest, revealed that basically each cellular (sub-) process is active at its own specific spot(s) in the experimental design space. Hence, the use of range finding, based on an affordable protocol like this, enables researchers to conveniently identify the ‘sweet spot’ for their cellular process of interest in an experimental design space and might have far-reaching implications for experimental standardization.

## Introduction

Design for experimentation plays an important role in transcriptomics research. There are several aspects that need to be considered: biological, technological, statistical and practical. Statistical principles for experimental design are well established [1]–[3] and usually applied. The technological, e.g. choice of (microarray) platform, or practical, e.g. available budget, considerations are of great importance, but depend mostly on the individual experimenter's setting. This leaves biological aspects, such as those related to time, dose and space, which are frequently intuitively considered or based on common practice. In molecular biology based research, common practices in perturbation experiments are by tradition regularly tuned to phenotypic observations, such as the apoptosis, cellular responses, or cell growth. This could be based on the assumption that these measurable phenotypic endpoints coincide with changes in gene expression that are directly relevant to the mechanism under study, which might not always be the case. In addition, phenotypic endpoints might (partially) originate from other biological processes, such as general stress, than the biological mechanism under study. As a consequence, the relevant genetic processes could occur at other experimental ranges than those investigated, causing these significant processes to be missed or polluted by non-specific stress processes. Hence, the selection of optimal experimental ranges within the design space should be an integral part of transcriptomics experimentation.

This holds especially true for time-series experiments, for instance in toxicogenomics exposure studies [4], [5]. Selecting a dose that is too high or low or a time point that is too early or late will have a profound effect on the insights that can be gained. In the recent past, we came across such an issue in a transcriptomics study regarding the role of p53 in response to UV-C exposure of Mouse Embryonic Fibroblasts (MEFs) [6]. The applied dose and time-scale were based on traditional experiment settings from the literature and the phenotypic endpoint apoptosis. Over one third of all genes were found to show differential expression (DEGs). In depth analysis revealed that this was a result of a general stress response, rather than a specific UV-C response [7]. There are other experiments with high numbers of DEGs that might suffer from similar problems. For example, in these two studies of UV exposure, one on cardiac cells [8] and one on human skin [9], about 40% of all genes were found to be differentially expressed.

**Figure 1:**
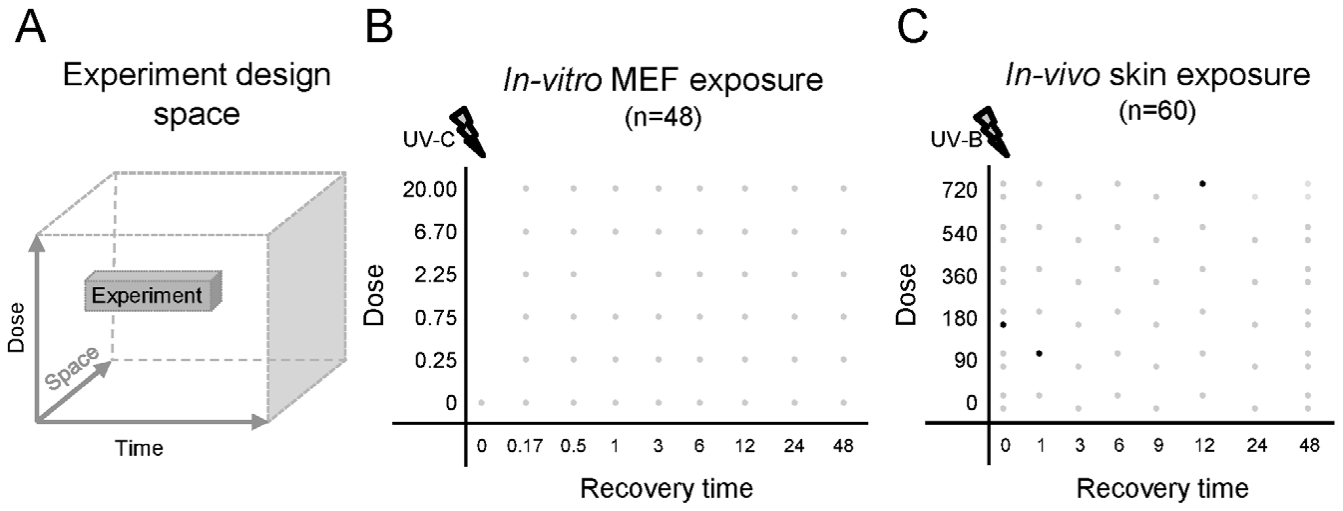
Experimental design space. Any experiment is designed in a design space defined by variable experimental parameters. A: a visualization of a hypothetical experiment in a design space defined by three variable experimental parameters. B: The *in-vitro* range-finding experimental setup with two variable experimental parameters: pulse exposure of MEFS by UV-C and recovery time after exposure. C; The *in-vivo* range-finding experimental setup with two variable experiment parameters: pulse exposure of mouse skin by UV-B and recovery time after exposure. Each dot represents a sample. Black dots indicate failed samples.

Any experimental design space in a mechanism-oriented transcriptomics experiment usually has multiple axes, e.g. time, dose, space, etc. (Figure 1A) and is in essence limitless. Given that technological and practical considerations restrict the number of experimental samples, it is essential to select parameter ranges that yield the relevant, most important information with respect to the biological question under study. As most biological processes are modular and each module often has its own optimal spot in the design space, there is a demand for a rather strict biological question for each experiment. Also, some responses are induced quickly after the perturbation and last shortly, whereas others will be different in these respects. Given all these uncertainties, it is quite impossible to upfront guess the optimal spot, i.e. ‘sweet spot’ (as coined in [10]) for a transcriptomics experiment in the total design space. Small-scale range finding tests can be helpful to discover an optimal experimental setup for a specific biological study.

Here, we provide a proof-of-concept range finding protocol for transcriptomics by determining the optimal dose and time ranges for studying several specific cellular processes in response to UV exposure. We show the value of executing a transcriptome-wide range-finding test before designing an in depth transcriptomics study, as was previously suggested [7], [10], [11]. Our approach is easy to set up, cost-effective, and covers a substantial part of the design-space. It can be used as blueprint protocol for designing expensive omics experiments. Both an *in-vitro* and an *in-vivo* case study are presented, emphasizing the broad applicability of the approach.

## Materials and Methods

### Ethics Statement

This study was agreed upon by the Animal Experimentation Ethical Committee of the RIVM in Bilthoven, the Netherlands under permit number 201200128. Animal handling in this study was carried out in accordance with relevant Dutch national legislation, including the 1997 Dutch Act on Animal Experimentation.

Biopsies were taken under Isoflurane anesthesia, at the end of the study animals were euthanized by cervical dislocation and all efforts were made to minimize suffering.

### *In-vitro* UV exposure experiment

Primary Mouse Embryonic Fibroblasts (MEFs) were isolated from E13.5 embryos in a C57BL/6 background (backcrossed for more than F8 generations). MEFs were cultured in Dulbecco's modified Eagle medium (Invitrogen, Breda, The Netherlands) containing 10% fetal bovine serum (Biocell, Rancho Dominguez, CA), 1% nonessential amino acids (Invitrogen, Breda, The Netherlands), penicillin (0.6 µg/ml), and streptomycin (1 µg/ml) at 37°C and under 5% CO2, 3% O2 conditions. The experiment was performed with early-passage MEFs (prior to passage five). MEFs were expanded and plated at 3.5×10^5^ cells per 6-cm plate. Six hours later when almost all cells are in G1 phase [12], cells were washed with phosphate-buffered saline and exposed to UV-C light at various doses (0.25, 0.75, 2.25, 6.7, 20.0 J/m^2^). Control samples were mock treated. At various time points after treatment (10 mins, 30 mins, 1 hrs, 3 hrs, 6 hrs, 12 hrs, 24 hrs, 48 hrs), MEFs were rinsed with phosphate-buffered saline and collected in 350 µl RLT buffer (RNeasy mini kit). An overview of all samples is shown in Figure 1B. Total RNA was isolated using the RNeasy mini kit (Qiagen, Valencia, CA). RNA quality was assessed with the Bioanalyzer 2100 (Agilent Technologies, Palo Alto, CA).

### *In-vivo* UV exposure experiment

All mice were males of 7–10 weeks of age and at least 10 times backcrossed in SKH hairless strain. Mice received normal feed and water ad libitum. Mice were UV-B exposed at various doses (90, 180, 360, 450, 720 J/m^2^) in a chamber containing Phillips TL12 lamps. Control mice were mock treated. At various time points after treatment (1, 3, 6, 9, 12, 24 hr) both treated and untreated mice were anaesthetized by isoflurane and 1.5 mm biopsies were sampled from the back by punching a half moon shape on folded skin. Biopsies were immediately snap frozen in liquid nitrogen and stored at −80°C until further processing. Total RNA was isolated as previously described in [13]. At 48 hrs after treatment all mice were euthanized by cervical dislocation after biopsies were taken. An overview of all samples is shown in Figure 1C. RNA quality was assessed with the Bioanalyzer 2100 (Agilent Technologies, Palo Alto, CA).

### Microarrays with custom Mouse Roche NimbleGen platform

Gene expression levels of the mouse samples were analyzed with a 12×135 k *Mus. musculus* microarray (Custom design GEO Platform accession number GPL17736) containing 24,302 genes based on NCBI-GeneID. Per RNA sample, 200 ng total RNA was amplified according to the Agilent LRILAK kit manual (Agilent technologies). Amino-allyl modified nucleotides were incorporated during the aRNA synthesis (2.5 mM rGAU (GE Healthcare), 0.75 mM rCTP (GE Healthcare), 0.75 mM AA-rCTP (TriLink Biotechnologies). Synthesized aRNA was purified with the E.Z.N.A. MicroElute RNA Clean Up Kit (Omega Bio-Tek). Each individual aRNA test sample was labeled with Cy3 and a reference sample, which was made by pooling equimolar amounts of RNA from either all *in-vitro* or *in-vivo* test samples, was labeled with Cy5. 5 µg of aRNA was dried down and dissolved in 50 mM carbonate buffer pH 8.5. Individual vials of Cy3/Cy5 from the mono-reactive dye packs (GE Healthcare) were dissolved in 200 µl DMSO. To each sample, 10 µl of the appropriate CyDye dissolved in DMSO was added and the mixture was incubated for 1 h. Reactions were quenched with the addition of 5 µl 4 M hydroxylamine (Sigma-Aldrich). The labeled aRNA was purified with the E.Z.N.A. MicroElute RNA Clean Up Kit. The yields of aRNA and CyDye incorporation were measured on the NanoDrop ND-1000. For the *in-vivo* experiment 3 samples failed the quality requirements and were discarded for further processing.

For both the *in-vitro* and *in-vivo* experiment each hybridization mixture was made up from a 1.1 µg test (Cy3) and 1.1 µg reference (Cy5) sample. Samples were dried and dissolved in 2 µl water. The hybridization cocktail was made according to the manufacturer's instructions (Roche NimbleGen Arrays User's Guide – Gene Expression Arrays Version 5.0, Roche NimbleGen). 5.2 µl from this mix was added to each sample. The samples were incubated for 5 min at 65°C and 5 min at 42°C prior to loading. Hybridization samples were loaded onto the microarrays and hybridized for 18 hours at 42°C with the Roche NimbleGen Hybridization System 4. Afterwards, the slides were washed according to the Roche NimbleGen Arrays User's Guide – Gene Expression Arrays Version 5.0 and scanned in an ozone-free room with a DNA microarray scanner G2565CA (Agilent Technologies). Feature extraction was performed with NimbleScan v2.5 (Roche NimbleGen). The array data have been deposited in NCBI's Gene Expression Omnibus (GEO) and is accessible through GEO Series accession numbers GSE50930 for the *in-vitro* experiment and GSE51348 for the *in-vivo* experiment

### Microarray data processing

The quality of the microarray data was assessed via multiple quality-control checks, i.e. visual inspection of the scans, testing against criteria for foreground and background signals, testing for consistent performance of the labeling dyes, checking for spatial effects through pseudo-color plots, and inspection of pre- and post-normalized data with box plots, ratio-intensity (RI) plots and PCA plots. All arrays passed the minimal criteria for quality assessment of the microarray data and were used in the analyses.

Handling, analysis and visualization of all data was performed in R (http://cran.r-project.org/) using the Bioconductor (http://www.bioconductor.org/) packages limma and maanova.

Log2 transformed data was normalized within-array using LOESS on an MA-plot of the Cy3 test sample data vs. the corresponding Cy5 reference sample data. Subsequently, the robust multi-array average (RMA) algorithm was performed on only the normalized Cy3-sample data for between-array normalization through summarization of the intensity values of the probes in a NCBI-GeneID probe set.

A mixed linear model was fitted on the *in-vivo* data to correct for the effects of the individual mice on the gene expression levels.

These normalized expression values were used for the generation of log2 ratios of zero dose points in time compared to the dose and time point zero. These log2 ratios were used to filter out the genes with a log2 FC >1 in the zero dose range from the whole data set.

The filtered data sets were used to make log2 ratios for the dose points per time point compared to the zero dose point of that time point. These log2 ratios were tested against 64 manually selected different gene sets (Table S5) for enrichment using the geneSetTest function from the Bioconductor package limma. The resulting p-values were corrected for false discoveries using the Benjamini-Hochberg procedure. Corrected p-values were used to generate experiment diagrams per gene set. These diagrams were colored using an adaptive color key based on the lowest corrected p-values found in the gene set. If the lowest corrected p-value was above 0.3 the coloring ranged from 0.3 (red) to 0.5 (grey). If the lowest corrected p-value was below 0.3 the coloring ranged from pmin (red) to pmin + pmax (grey), where pmin is the lowest corrected p-value, and pmax depended on the experiment. Pmax was set to 0.2 in the in-vitro experiment and to 0.01 in the in-vivo experiment. ‘Sweet spots’ were defined as the areas in the diagrams that were colored red.

Dose-response relations for each gene per gene set were generated from the filtered data sets and tested using Pearson correlation. Absolute correlations >0.8 were marked as relevant. Genes with no correlations >0.8 in any of the time points were removed from Tables 1 and 2 and Tables S3 and S4. All time points were visualized in the tables and the time points per gene set that contained a ‘sweet spot’ in any dose were marked with grey.

**Table 1:** *In-vitro* example of dose-response correlations of individual genes per time point.

Nucleotide Excision Repair					
gene	10 h	30 h	60 h	180 h	360 h*
Rfc3		0.82			0.8
Ercc2			−0.93		
Pole3			0.88	0.95	
Cetn2			0.88		0.94
Pold3			−0.85		
Pold4			0.81		
Mnat1				−0.97	−0.86
Xpa				0.88	
Rbx1				0.87	
Gtf2h2				−0.85	
Ercc5				0.82	0.88
Gtf2h1				0.82	
Rfc4					0.91
Rfc5					0.89
Pold2					−0.88
Rad23b					0.88
Ercc4					0.87
Ercc8					0.85
Gtf2h5					0.83
Ercc1					−0.82
Xpc					0.82

Per time point of the *in-vitro* range-finding experiment, dose-response correlations are depicted for each gene of the KEGG nucleotide excision repair gene set that is found at least once significantly correlated (>0.8). Columns with an “*” indicate the time points in which at least one sample was found significantly differential expressed in the gene-set enrichment (Figure 4).

*location of the sweet spot.

**Table 2:** *In-vivo* example of dose-response correlations of individual genes per time point.

p53 Responsive Elements							
gene	1 h*	3 h	6 h*	9 h	12 h	24 h*	48 h
Msh2	−0.97						
Btg2	0.95						
Igfbp3	−0.87	−0.86		−0.8	−0.86	−0.86	
Sesn1		−0.98		−0.87	−1		
S100a2		0.94					
Pmaip1		0.87	0.81		0.97		
Fas		−0.85					
Mdm2			0.92	0.89	0.8	0.88	
Cdkn1a			0.91	0.86	0.95	0.94	0.85
Gml			0.91				
Tnfrsf10b			0.9	0.89	0.86	0.89	
Pcna				0.81	0.86		
Sfn						0.86	

Same setup as for Table 1, now for the *in-vivo* range-finding experiment and the IARC p53 responsive elements gene set.

*location of the sweet spot.

## Results

### Setting the experimental parameters

Before looking for the location of the ‘sweet spot’ for follow-up UV experiments in a design space defined by variable parameters (Figure 1A), we also evaluated the non-variable experimental parameters. As a consequence, we choose optimized standard values of important constant experimental parameters associated with this design space based on current knowledge. For the *in-vitro* study, the oxygen level, under which the MEFs were cultured, was lowered from 21% to 3% [14] and the cells were synchronized before the start of the experiment [12]. As no information was available to determine several of the *in-vivo* constant experimental parameters, we performed a number of tests that determined the optimal biopsy punch diameter size and RNA isolation protocol [12], as well as the maximum number of subsequent biopsy sampling from the same animal. For the latter, the tests indicated that six biopsies can be taken over time from one animal, without these biopsies affecting each other (Data S1). These optimized parameters set the constant parameter values for the experiments. This left us with both an *in-vitro* and an *in-vivo* experimental design space defined by two variable parameters: UV dose and recovery-time-after-exposure.

### Defining the experimental design space

To determine the optimal spot for any specific biological process in a given experimental design space defined by relevant variable experimental parameters, this design space needs to be explored. Such an exploration can be done by using small-scale range-finding transcriptomics experiments that identify transcriptome-wide gene expression in the relevant section of the design space. As proof-of-concept, we performed two such range-finding experiments with our two variable parameters: UV-exposure dose and recovery time-after-exposure (Figure 1B and C). For this both *in-vitro* UV-C exposed MEFs, as well as *in-vivo* UV-B exposed mouse skin were used. The range-finding approach that we suggest here aims to explore the design-space at a high density without replicate sampling. The *in-vitro* range-finding experiment consisted of 48 samples from synchronized MEF cell-cultures grown under a 3% oxygen level after exposure to various doses of UV-C exposure and different recovery periods (Figure 1B). The *in-vivo* range-finding experiment consisted of 60 samples from mouse skin-biopsies after exposure to various doses of UV-B exposure and different recovery periods. To estimate the inter-individual variation, two mice were used per UV-B dose and a control sample was taken from each mouse before exposure. Samples at the remaining time points were collected alternating between mice with the same exposure dose (Figure 1C). Both UV-B and UV-C induce p53 through similar DNA damage response. UV-C was selected as radiation source for *in-vitro* as this is common practice for *in-vitro* studies on UV and p53 [6], [12], [15] whereas UV-B is commonly used in *in-vivo* studies [16]–[18]. In addition, UV-C seems to be too harmful for *in-vivo* use.

Both the *in-vitro* and *in-vivo* timeframes and UV-doses are based on a range of p53 protein accumulation and phosphorylation and on apoptosis studies in previous experiments on wild type p53+/+ MEFs or mice. For the *in-vitro* time range we used earlier *in-vitro* experiments that showed p53 accumulation at early time points (3 hr), peaking around 12 hours and returning to normal 24 hours after irradiation. In addition, the apoptosis response is shown to occur from 8–12 hours, peaking around 16–20 hours and continuing to increase until 48 hours after irradiation. We enriched the early time points since gene expression often precedes these phenomena and because we observed a clear change at t = 6 in previous gene expression studies [12], [19], [20]. The *in-vivo* time range was chosen based on previous *in-vivo* experiments in p53+/+ mice (hairless). These showed p53 accumulation and apoptosis at 6 and at 24 hours after UV exposure [15]. The selection of the *in-vitro* dose points was based on experiments that studied the effect of different doses *in-vitro* on p53 accumulation, phosporylation and apoptosis [6], [19], [20]. The most and at the same time highest dose studied is 20 J/m^2^. Based on earlier gene expression range studies we made the observation that 20 J/m^2^ was high (no cell growth). A recent study [20] shows that 37% of cells survive at a UV dose of 5 J/m^2^. Lower doses were selected since gene expression is more sensitive than endpoint apoptosis/cell survival. The *in-vivo* dose point selections were based on the minimal erythemal dose (MED, appearance of red skin) for p53+/+ mice that was 900 J/m^2^. Accumulation of p53 is observed 24 hours after UV exposure at a dose of 300 J/m^2^ [15]. Stepwise doses were selected lower and higher. Here, lower doses were included as gene expression may be more sensitive than p53 staining or caspase 3 staining of the skin.

### Assessing the RNA yield

**Figure 2:**
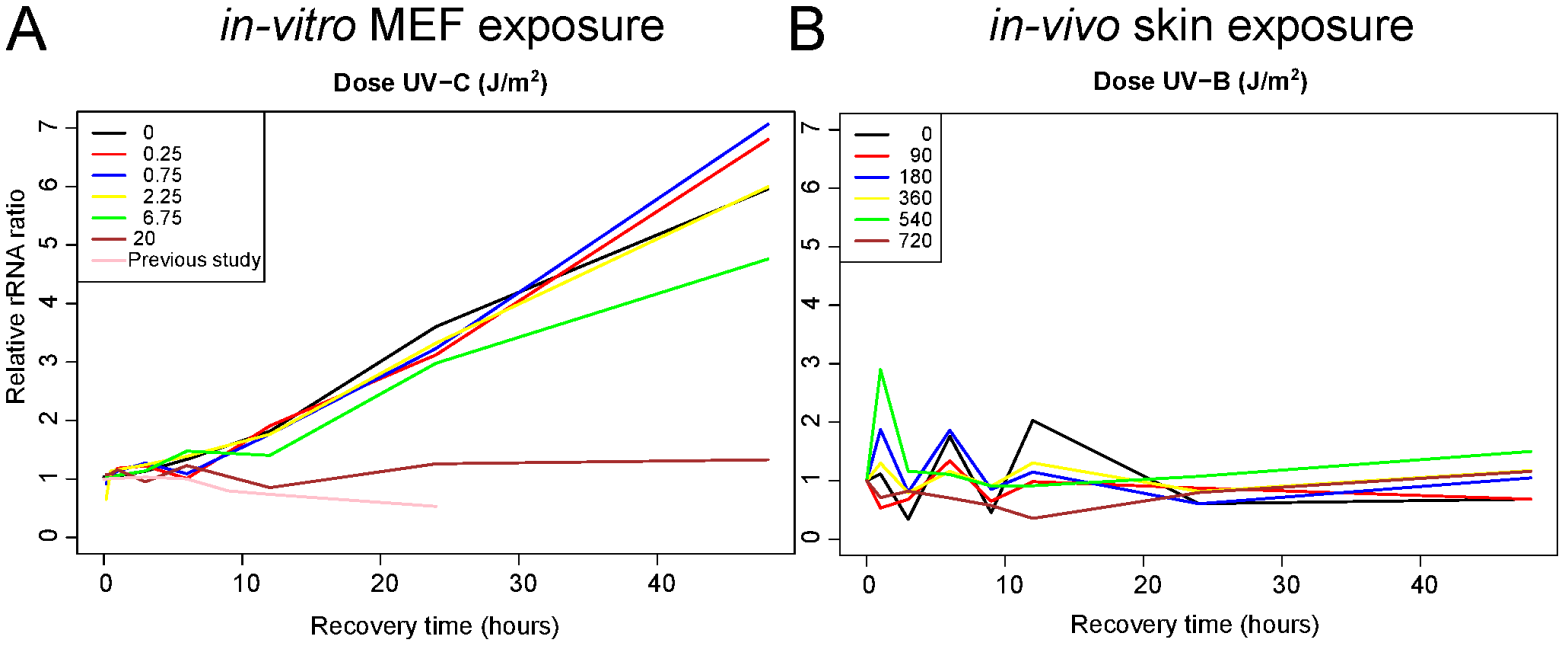
Effect of UV exposure on RNA yield. Relative RNA yields for all A: *in-vitro* and B: *in-vivo* experimental samples are given as compared to the RNA yield of the t = 0 sample in each experiment. In A also the RNA yields from a previous *in-vitro* UV exposure experiment with 21% oxygen, non-synchronized culture conditions and 20 J/m^2^ UV-C [6] exposure are presented as reference. The RNA quality of the individual samples was of good quality with a minimum RIN value of at least 9.1 in the *in-vitro* experiment and 6.8 in the *in-vivo* experiment (Tables S1A and S1B).

In previous studies [6], [7], we observed large effects on the transcriptome caused by strong stress perturbations, which were reflected by substantial changes in the rRNA and mRNA yields (Figure 2A). We therefore assessed the total RNA yields in both experiments (Figure 2). In the *in-vitro* experiment, unexposed cell growth causes an about six-fold increase in total RNA yield per culture plate between the first and last time point. Here, a clear dose-related effect was present: at low UV-C doses there is a slight additional increase in total RNA yield as compared to the unexposed control sample and the increase in total RNA yield is reduced until the point of arrest at the highest UV-C dose (Figure 2A). In comparison, the previous *in-vitro* experiment with high oxygen level and 20 J/m^2^ UV-C exposure showed a decrease in total RNA yields over recovery time, most likely caused by cell death and/or systemic RNA degradation. In contrast, there was no noticeable dose-related effect on total RNA yield in the *in-vivo* experiment (Figure 2B)

A rRNA/mRNA ratio that changes significantly during an experiment can severely hamper data analysis as input total RNA consists mostly of rRNA [12]. To approximate relative mRNA levels, we used the aRNA levels after linear amplification via *in-vitro* transcription (IVT). In both experiments the IVT amplification was started with the same amount of RNA, effectively normalizing the existing differences in total RNA yields. Three RNA samples in the *in-vitro* experiment did not amplify properly and were excluded from further analysis (Figure 1C and Table S1B). In the previous *in-vitro* UV exposure experiment, there was a slight decrease in mRNA level over recovery time (Figure S1A). In the current *in-vitro* experiment there seems little change in mRNA level (Figure S1A). Although in the *in-vivo* experiment there were differences in mRNA levels, there seems no relation to the UV dose (Figure S1B). Combining the total RNA and mRNA estimations indicates that the rRNA/mRNA ratio was not substantially changed in these experiments.

### Restricting the experimental design space by estimating differentially expressed genes

**Figure 3:**
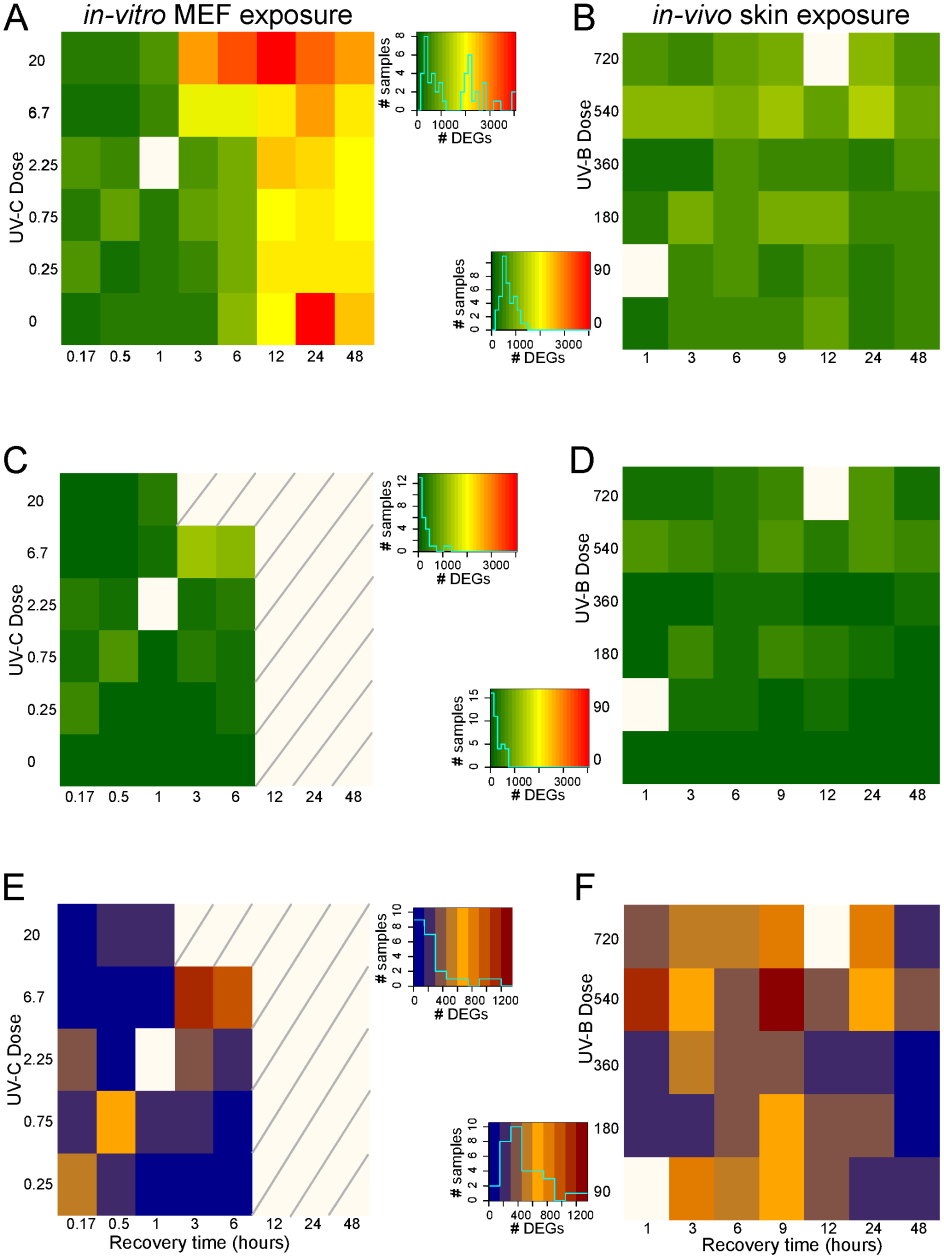
Number of differentially expressed genes. A: The number of differentially expressed genes (DEGs) for each *in-vitro* experimental sample as compared to the t = 0 samples applying a gene-expression ratio cut off of log2 FC>1. B: As A for the *in-vivo* experiment. The colors represent the number of DEGs according to the schemes in the middle. The blue lines in the middle schemes represent the number of samples with a given number of DEGs for the *in-vitro* (upper) and *in-vivo* (lower) experiment. C: The number of DEGs for the *in-vitro* experimental samples with less than 2.000 DEGs as compared to the t = 0, applying a gene-expression ratio cut off of log2 FC>1, after removal of the genes with non-relevant differential expression (i.e. genes that were differentially expressed in any untreated sample). D: As C for the *in-vivo* experiment. E: The number of DEGs for the *in-vitro* experimental samples compared to the associated dose = 0 sample applying a gene-expression ratio cut off of log2 FC>1. F: As E for the *in-vivo* experiment. The colors represent the number of DEGs according to the schemes in the middle. The blue lines in the middle schemes represent the number of samples with a given number of DEGs.

In experimental range finding, the aim is usually to detect those ranges at which specific responses to a given perturbation are induced. A high number of differentially expressed genes (DEGs) is an indication of a perturbation range that induces non-specific responses. As such, we determined the DEGs in both experiments. Log2 fold change (FC) ratios of normalized gene-expression values compared to the dose and recovery time point zero were generated for each point in our experimental design spaces. In the *in-vivo* experiment an additional correction for individual mouse effects was applied. An arbitrary cut-off of log2 FC>1 was used to estimate the number of genes in the transcriptome with a changed expression. Visualizing the numbers of DEGs in both experimental design spaces revealed huge differences (Figures 3, Figure S2 and Table S2). If we assume that around 2,000 (∼10% of all genes assessed) or more DEGs are indicative for non-specific responses, which would be in concordance with yeast [21], it becomes clear that in the *in-vitro* experiment any recovery time-point from 12 hours on, will only yield non-specific responses. This might be a result of the fact that the synchronization of cells at the start of the experiment begins to collapse due to continuous cell growth [12]. A strong indication is the fact that this effect is quite prominent even without any UV exposure (Figure 3A, Figure S2A and Table S2A). It is also clear that the highest dose of 20 J/m^2^ induces huge changes in the transcriptome, already after three hours of recovery time. All the areas of the *in-vitro* experimental design space with over 2,000 DEGs are considered unusable to study specific UV-C responses (Figure 3A, Figure S2A and Table S2A). In contrast, in the *in-vivo* experimental design space, the maximum number of DEGs was about 1,350, which means that using the same criterion, no restriction in this design space was needed (Figure 3B, Figure S2B and Table S2B).

### Removing genes from baseline cellular processes with expression changes

Even with all experiment setup efforts towards synchronization of cells and standardization of protocols, there are still genes that showed differential gene expression without the perturbation, i.e. UV dose zero (Figures 3A, 3B, Figures S2A, S2B, Tables S2A and S2B). As already noted, in the *in-vitro* experiment this is to be expected, as cells continue to grow and as such their environment, to which they respond, changes. In the *in-vivo* experiment this phenomenon is less prominent, but still present. Our previous, unpublished, study (Data S1) showed that for instance genes related to circadian rhythm show differential expression *in-vivo*. Although explicable, the consequence is that any differential expression of these genes in the experimental design space outside the zero dose range cannot or at least not exclusively be attributed to the cellular response associated with the perturbation. In this analysis we do not want to further explore their role. As such, we decided to remove these genes with differential non-exposure ‘background’ expression from the data sets altogether. In total, 1,854 and 1,814 (Tables S2A and S2B) UV-unrelated DEGs (FC>2 in relation to t = 0) were identified in the untreated samples from the *in-vitro* and *in-vivo* data set, respectively and were removed (Figures 3C, 3D, Figures S2C, S2D, Tables S2C and S2D). The number of DEGs in the experimental design spaces were recalculated using the associated untreated sample, which resulted in 73% of the samples in the *in-vitro* experiment having less than 300 DEGs, whereas 70% of the samples in the *in-vivo* experiment have over 300 DEGs (Figures 3E, 3F, Figures S2E, S2F, Tables S2E and S2F). There seems to be little overlap over time or dose between the genes found with FC>2. This effect stays consistent throughout the filtering steps (Tables S2A–S2F).

### Identifying the experimental ‘sweet spot’ in the design space

**Figure 4:**
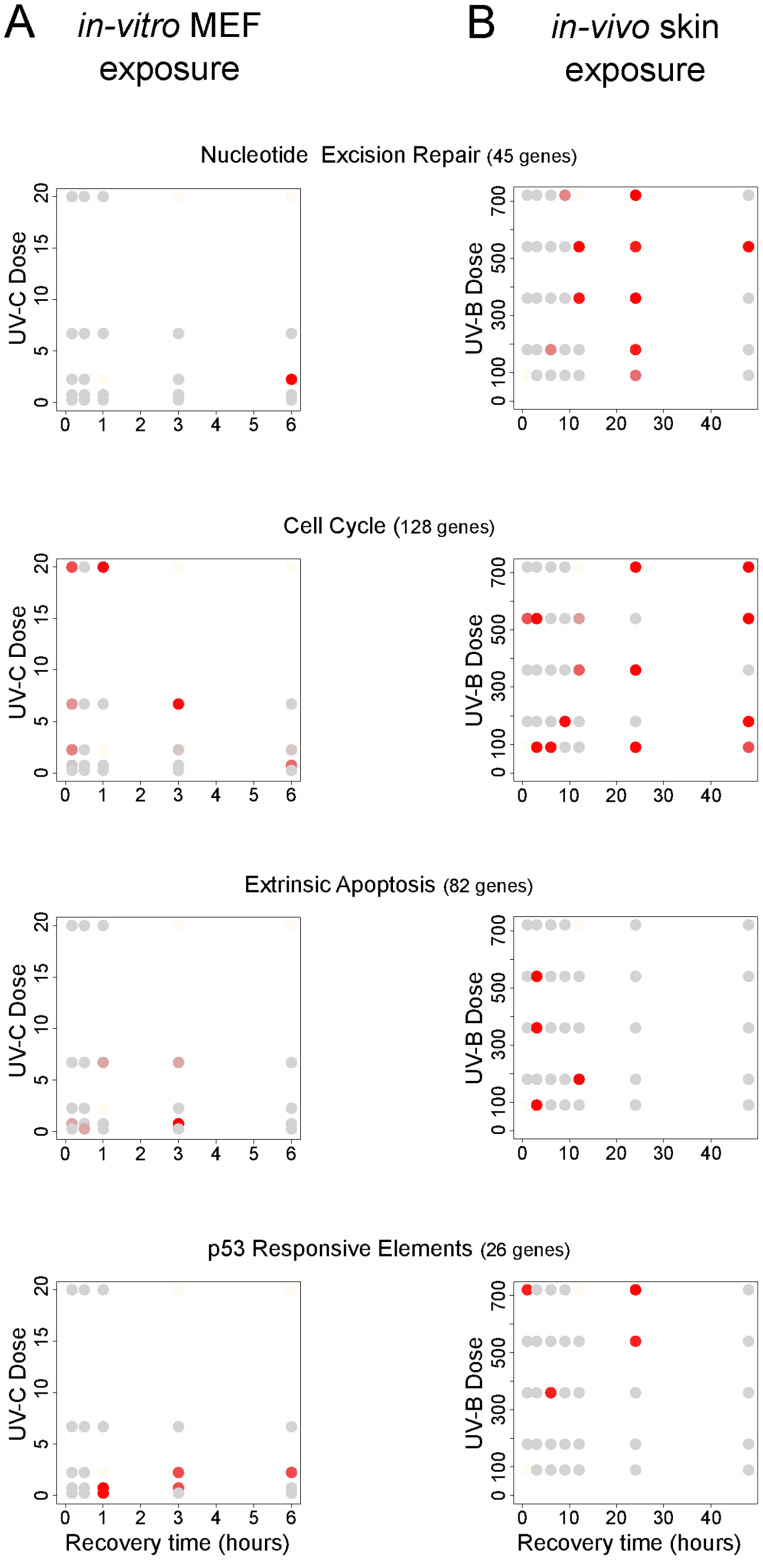
Cellular process specific responses in the experiment design space. For each specified gene set, the spots that potentially are a so-called ‘sweet spot’ in the experimental design space are indicated (red). The sweet spots are identified as those samples that have the lowest p-values within the defined range in a gene set enrichment analysis.

Although obvious, the ‘sweet spot’ in any experimental design space primarily depends on what cellular mechanism the ultimate experiment is aimed at. In our (restricted) design spaces, all occurring cellular processes can in principle be evaluated. An advantageous and more focused way is to explore an experimental design space using prior knowledge. This can be done by determining whether the genes known to be involved in a cellular process of interest have a changed expression as compared to the onset of the experiment. To substantiate this search, we explored the design spaces by performing gene-set enrichment analyses for 64 predefined, expert-selected, cellular processes and pathways retrieved from various commercial or freely available databases such as KEGG, BioCarta, Metacore, Ingenuity, and so on (Table S5), combined with an intuitive visualization of the results (Figure 4, Figures S3 and S4). Also, randomly created pathways were tested to check whether or not the patterns in the visualizations are likely be generated by chance, which turned out not to be the case (data not shown).

In the *in-vitro* experiment for instance, a set of known genes (n = 45) of the nucleotide excision repair (NER) pathway, showed to be most active at one specific spot in the design space: 6 hours and 2.25 J/m^2^ UV-C dose (Figure 4A). At other dose-time points the enrichment of this gene set was not significant. In contrast, in the *in-vivo* experiment, NER genes change mostly at 12–48 hours and 180–720 J/m^2^ UV-B doses. Compared to NER genes, cell cycle genes (n = 128) are changed in the *in-vitro* experiment at an earlier time and a much higher UV dose (Figure 4A). As can been construed from the presented examples (Figure 4), each cellular process, pathway, or collection of genes shows its own preferential spot in the design space where genes of this process are most significantly enriched in the high FCs. This indicates the sought after ‘sweet spot’ for each of those groups of genes to be studied in the context of the associated cellular process and its involvement in UV response.

However, as is clear from the examples, some gene sets do show change in several spots in the experiment design space. This is most obvious in the *in-vivo* design space and could be the result of the fact that a specific gene set is widely used, or too broadly defined so that it actually represents more than one biological process. To still enable an informed decision, we zoomed in on the individual genes of these gene sets. The doses-response (i.e. gene expression) relation of each gene is an important indicator for the involvement of a gene in the response to an exposure. Each gene has a doses-response curve over each time point. The dose-response correlations were determined per time point for each gene in a gene set that was still present in the filtered data. (Anti-) correlations >0.8 indicate a strong relation between the exposure and the gene-expression (Tables 1, 2, Tables S3 and S4). In the *in-vivo* example of NER genes, at the gene set ‘sweet spot’ time point 6 h, 13 genes show a strong correlation. At time point 3 h, only 7 such genes were found, of which only 2 overlapped with those from time point 6 h (Table 1). Also at 1 h correlated genes are found, like those at 3 h these could be different sub processes. In the *in-vivo* example of the p53 responsive elements, the same phenomenon occurs in that each time point has a different set of dose-correlating genes. There are clearly genes that are involved in an early, middle, or late responses to UV exposure (Table 2). Depending on the ever advancing knowledge of these genes, an interpretation can be made as to at what time point the genes and/or cellular processes of interest are active. By presenting the dose-response relation of individual genes, biologist have a tool to qualitatively make a choice where in the experimental design space they want to perform their ultimate experiment, focused on a specific gene or biological process.

## Discussion

This study addresses several issues that are involved with design for transcriptomics experimentation. To find the best spot in an experimental design space defined by the variable parameters, we developed a protocol that allows for determining the ranges within the experimental design space where relevant responses of the process under study occur. Instead of using phenotypic markers, we examined gene expression directly, thereby avoiding the potential problem that relevant transcriptome changes often precede phenotypic changes as well as that phenotypic changes can be a combined outcome of multiple, including non-relevant biological processes. Our proof-of-concept protocol can be summarized as follows: 1) frame a specific biological question with an associated gene-set; 2) define a wide-ranged sample space and sampling scheme without replication; 3) obtain transcriptomics data and estimate DEGs; 4) restrict the experimental design space by eliminating areas with potentially stress-related and noise-related DEGs; 5) determine the experimental ‘sweet spot(s)’ for the biological question using the selected gene set in gene set enrichment analysis and in dose-response correlation analysis for each point in the design space.

By this pragmatic approach, the complex modularity of biological processes is taken into account by the possibility of using gene sets that comprise different levels of organization. Larger processes can be investigated, but also smaller sub-processes can be checked. Crucial here is that different cellular processes will occur at different ranges in the design space, so the processes of interest must be well defined to find the right spot in the design space. For instance, we found the spots in our *in-vivo* experimental design space where NER occurs, to be much later in time than those with extrinsic apoptosis (Figure 4). In the overview of all tested 64 processes (Figures S3 and S4), most spots show relevant transcriptome changes for one or more processes in the design space. This is in line with the fact that at each spot specific cellular processes are occurring.

There are also instances where for one particular process many spots in the design space show transcriptome changes. For instance, the process Cell Cycle showed significant transcriptome changes almost throughout the whole *in-vivo* experiment design space (Figure 4B). This could imply that the 128 genes of this gene set are not defined specific enough and need to be further subdivided in gene sets that are indicative of cell-cycle arrest or cell cycle initiation etc. Another example is the gene-set p53 responsive elements. Given the important role p53 plays in many cellular processes, it is to be anticipated that range-finding with this gene set will result in many potentially interesting spots in the design space. Again, using smaller subsets or exploring the behavior of individual genes as proposed in our protocol, might overcome this issue. However, we have found that when gene sets become too small (below 20 genes), the removal of single genes, for instance due to the application of different filtering steps, can render the results for such a set unstable.

Considering the experimental design, one might argue that the experimental setup of this study could be improved upon. For instance, the use of a common reference sample pool to co-hybridize with the samples instead of directly comparing against the zero dose for the same time point and the time point zero for the mock-treated samples. This approach can result in inflated technical variation [22]. However, due to the exploratory nature of this study, we wanted to be able to compare all samples with one another. This approach kept all options open. The initial choices for our design ranges were derived from common practice. In hindsight, these choices seem to have been sufficient for our goals, as we obtained a good insight in the relevant ranges for the processes we want to study. Only the high dose, late in time combinations for the *in-vitro* study might have been left out to save on costs as these gave uninterpretable results as was expected based on the known biology.

The used gene set test is an important component of the approach presented. It is based on permutation of genes and assumes that the genes in the set are no more correlated on average than randomly chosen genes. It is well known that this assumption is likely to be violated, as inter-gene correlations are likely to occur. Hence, we would preferably have used a methodology that takes these correlations into account. However, this would require biological replicates [23], [24], which we do not have in these range finding studies. If the assumption is violated, then the gene set test results in p-values that are generally too low, and the test results may therefore be over-optimistic. Yet, in this study we are not interested in determining an exact p-value. Rather, we are screening for ranges in which certain pathways are most activated. The gene set test results are sufficient to define such ranges, and exact p-values can be calculated in the follow-up study with biological replication.

Altogether, these findings do underline the importance of defining the exact biological question before the execution of a study. This would argue in favor of hypothesis-driven rather than data-driven research. Also, the approach often used by molecular biologists to put as many questions, as they possibly can come up with, in one (combined) experiment is not suitable for transcriptomics research. Often the argument of costs is employed as an excuse not to perform range-finding studies. The small-scale range finding studies proposed here are relatively cheap, as no replications are used and the costs for these types of experiments are still decreasing. In fact, this approach could in essence also be done by quantitative PCR only using the genes of interest. Vice versa, one could argue that an experiment executed at an less informative range will be more costly in the end. Of course, the amount of samples used here might be equal to the size of a simple study and might not be cost-effective in comparison. Again, the use of a qPCR based approach might be more interesting in this case. Also, these small studies are often part of a larger research strategy, for which it will be useful to run small-scale range finding studies upfront.

Previous studies also recommended the use of range finding for the selection of experiment sampling points for transcriptomics [25], [26]. However, these methods were focused solely on the discovery of optimal time points and not on any other axes in experiment design space, such as dose. Others have looked at the phenotypic level [27] and might therefore, as discussed before, run a greater risk of being outside the optimal ranges on the transcriptomics level. We are convinced that our proposed approach can help in advancing transcriptomics experiment design and thus improving the insights in specific responses of a system to perturbations by avoiding non-specific responses.

Given the clear nature of the results presented here and the common-sense rationale that is behind it, we anticipate that range-finding tests will become general practice in transcriptomics experimentation in the near future. To support this, we suggest that the results of all range-finding studies are deposited in world-wide data repositories, so other researchers can use these data to determine for their specific cellular process of interest where they should design their experiment. At the same time it could provide means by which researchers can see in which -unexpected- experimental setups, i.e. cell state or response to perturbations, their favorite cellular process or gene is involved. Obviously, this only applies if the experimental setup is (near) identical. As such, range finding might even lead to more experimental standardization of specific research domains.

## Supporting Information

Figure S1 **Effect of UV exposure on mRNA yield.** Relative mRNA yields for all *in-vitro* (A) and *in-vivo* (B) experimental samples compared to the mRNA yield of the t = 0 sample in each experiment. The RNA yields from a previous *in-vitro* UV exposure experiment are presented as reference (A).
(PDF)

Figure S2 **Number of differentially expressed genes over time.** Profile plots over time of the number of DEGs for each dose in both experiments (See also Table S2).
(PDF)

Figure S3 **Cellular process specific responses in the** ***in-vitro*** **experiment design space.** The potential sweet spots in the in-vitro range-finding experiment diagrams for all 64 tested gene sets (same set up as Figure 4A).
(PDF)

Figure S4 **Cellular process specific responses in the** ***in-vivo*** **experiment design space.** The potential sweet spots in the in-vivo range-finding experiment diagrams for all 64 tested gene sets (same set up as Figure 4A).
(PDF)

Table S1 **RNA isolation metrics.** Detailed RNA sample information for the in-vitro experiment (A) and the in-vivo experiment (B).
(PDF)

Table S2 **Differentially expressed genes.** The numbers of differentially expressed genes (DEGs, log2 FC>1) found in both experiments, if compared to time-point 0 (A & B, Figure 3A & B); after additional removal of genes that were differentially expressed in any untreated sample (C & D, Figure 3C & D); after re-calculation using the associated dose = 0 sample (log2 FC>1) (E & F, Figure 3E & F). Excluded samples are indicated in grey.
(PDF)

Table S3 ***In-vitro*** **examples of dose-response correlations of individual genes per time point.** Dose response correlations per time point of individual genes that belong to gene sets: KEGG nucleotide excision repair, KEGG cell cycle, KEGG extrinsic apoptosis and IARC p53 responsive elements, in the in-vitro experiment (set up as Table 1).
(PDF)

Table S4 ***In-vivo*** **examples of dose-response correlations of individual genes per time point.** Dose response correlations per time point of individual genes that belong to gene sets: KEGG nucleotide excision repair, KEGG cell cycle, KEGG extrinsic apoptosis and IARC p53 responsive elements, in the in-vivo experiment (set up as Table 2).
(PDF)

Table S5 **Overview of GeneID's per gene sets used for GeneSetTesting.**
(XLSX)

Data S1 **Multiple longitudinal biopsies sampling in individual mice.**
(PDF)
