# Supplementary material for: A Range Finding Protocol to Support Design for Transcriptomics Experimentation: Examples of In-Vitro and In-Vivo Murine UV Exposure
Source: PLoS One. 2014 May 13;9(5):e97089. doi: 10.1371/journal.pone.0097089 (PMC4019648; doi:10.1371/journal.pone.0097089)

Figure S1. Effect of UV exposure on mRNA yield

Relative mRNA yields for all *in-vitro* (A) and *in-vivo* (B) experimental samples compared to the mRNA yield of the t=0 sample in each experiment. The RNA yields from a previous *in-vitro* UV exposure experiment are presented as reference (A).

A *in-vitro* MEF exposure

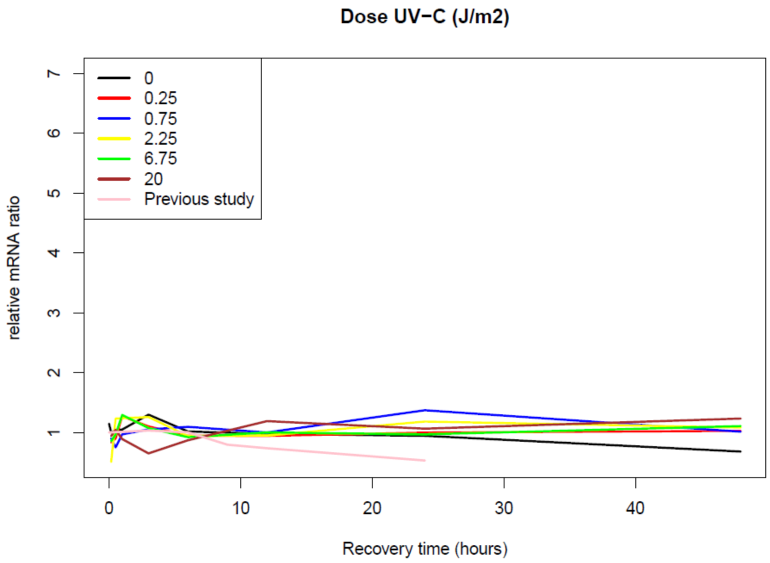

B *in-vivo* skin exposure

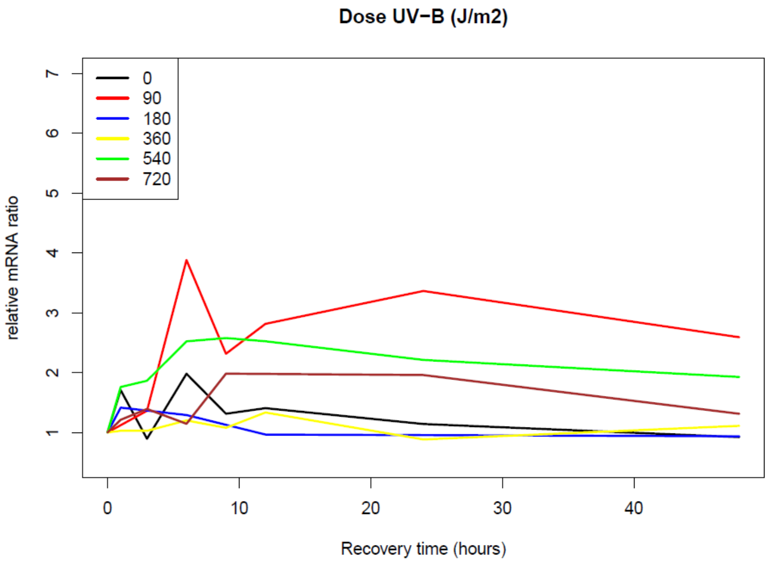

Supplement: Figure S1 — Effect of UV exposure on mRNA yield. Relative mRNA yields for all in-vitro (A) and in-vivo (B) experimental samples compared to the mRNA yield of the t = 0 sample in each experiment. The RNA yields from a previous in-vitro UV exposure experiment are presented as reference (A). (PDF) [file pone.0097089.s001.pdf]
