# Supplementary material for: A Range Finding Protocol to Support Design for Transcriptomics Experimentation: Examples of In-Vitro and In-Vivo Murine UV Exposure
Source: PLoS One. 2014 May 13;9(5):e97089. doi: 10.1371/journal.pone.0097089 (PMC4019648; doi:10.1371/journal.pone.0097089)

Figure S2. Number of differentially expressed genes over time

Profile plots over time of the number of DEGs for each dose in both experiments (See also Table S2).

In-vitro MEF exposure | *In-vivo skin exposure*

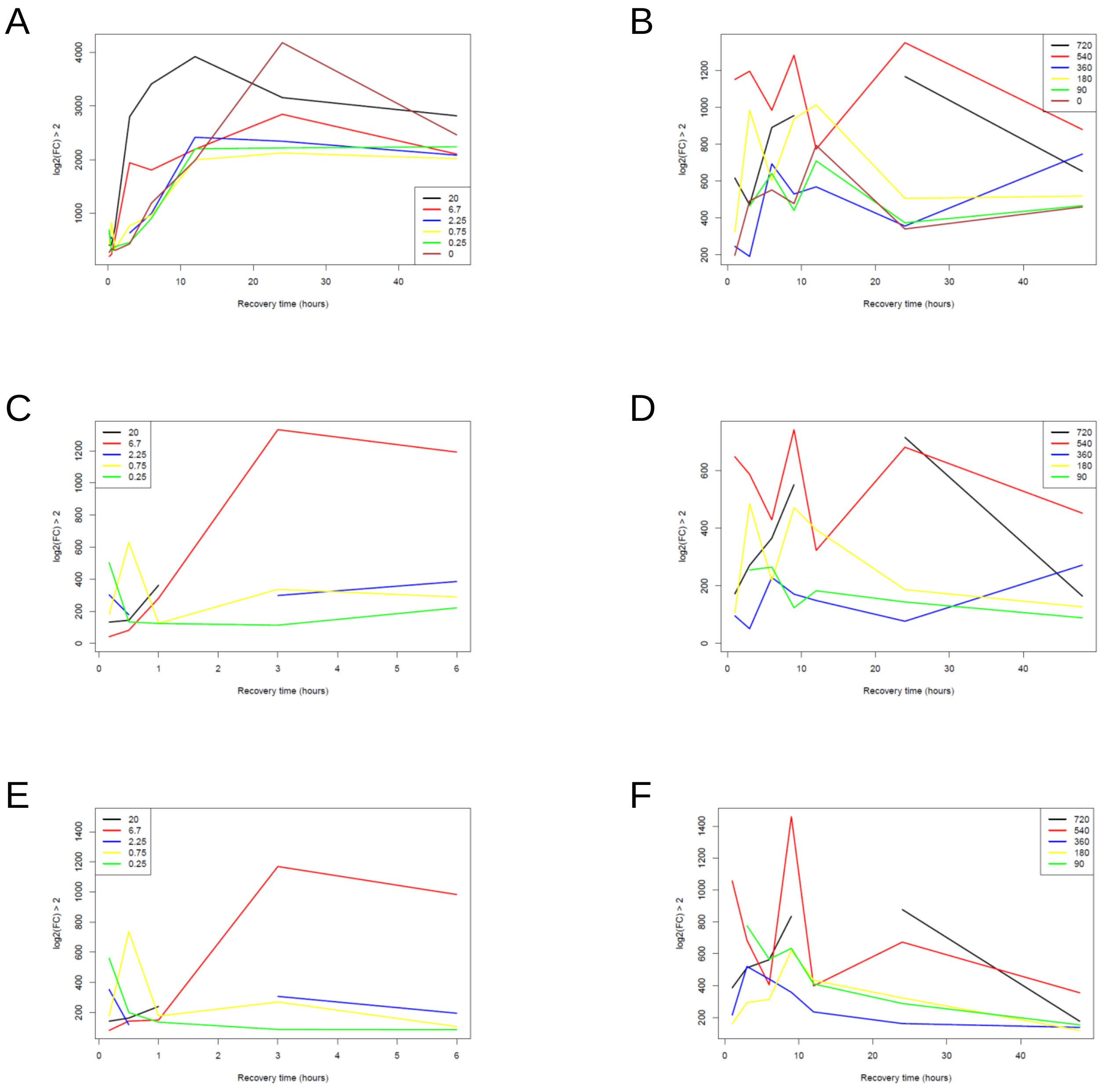

Supplement: Figure S2 — Number of differentially expressed genes over time. Profile plots over time of the number of DEGs for each dose in both experiments (See also Table S2). (PDF) [file pone.0097089.s002.pdf]
