# Supplementary material for: A Range Finding Protocol to Support Design for Transcriptomics Experimentation: Examples of In-Vitro and In-Vivo Murine UV Exposure
Source: PLoS One. 2014 May 13;9(5):e97089. doi: 10.1371/journal.pone.0097089 (PMC4019648; doi:10.1371/journal.pone.0097089)
Supplement: Figure S3 — Cellular process specific responses in the in-vitro experiment design space. The potential sweet spots in the in-vitro range-finding experiment diagrams for all 64 tested gene sets (same set up as Figure 4A). (PDF) [file pone.0097089.s003.pdf]

Figure S3. Cellular process specific responses in the *in-vitro* experiment design space

The potential sweet spots in the *in-vitro* range-finding experiment diagrams for all 64 tested gene sets (same set up as Figure 4A).

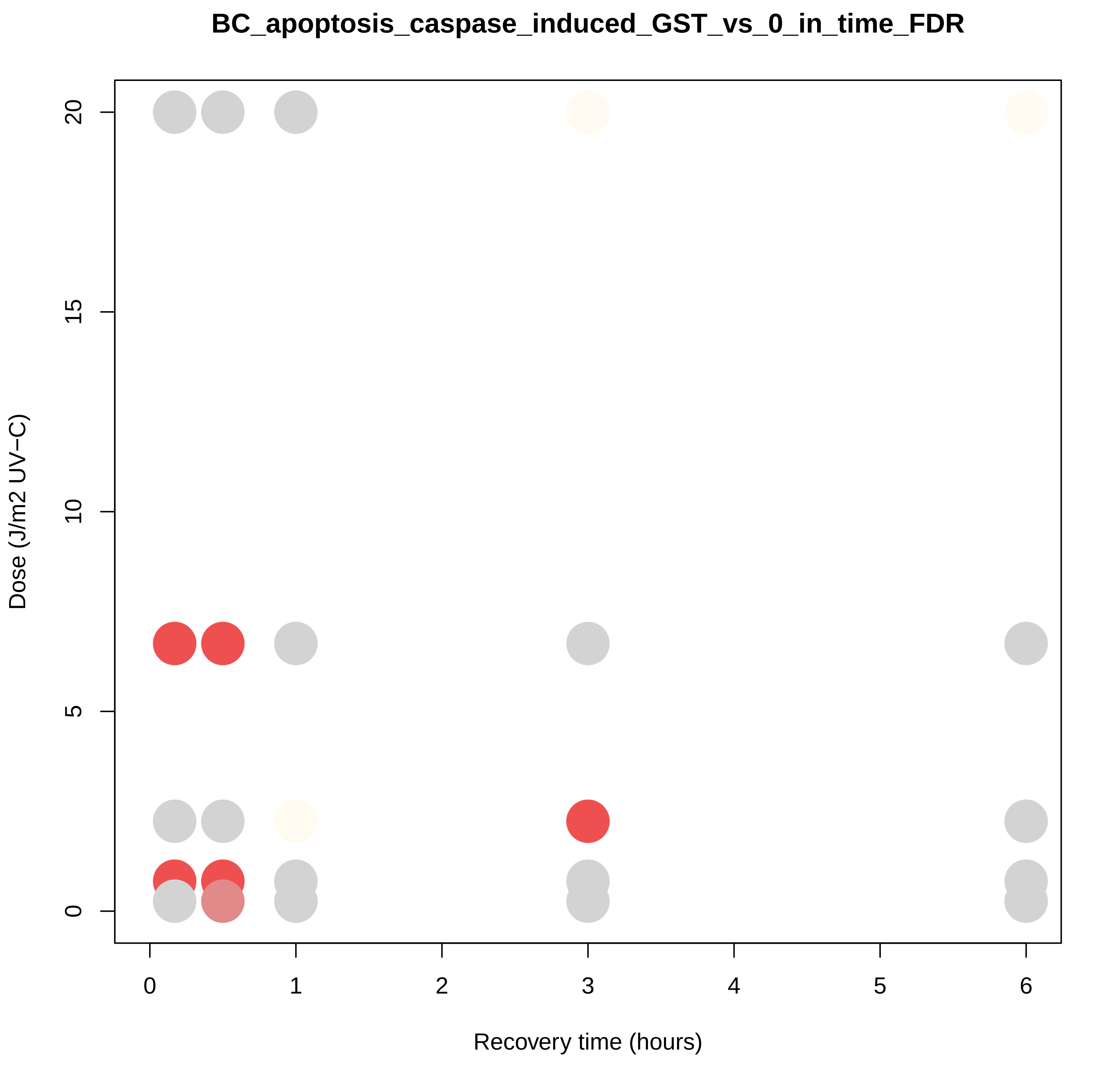
BC_apoptosis_caspase_induced_GST_vs_0_in_time_FDR
Dose (J/m2 UV−C)
0
5
10
15
20
Recovery time (hours)
0
1
2
3
4
5
6

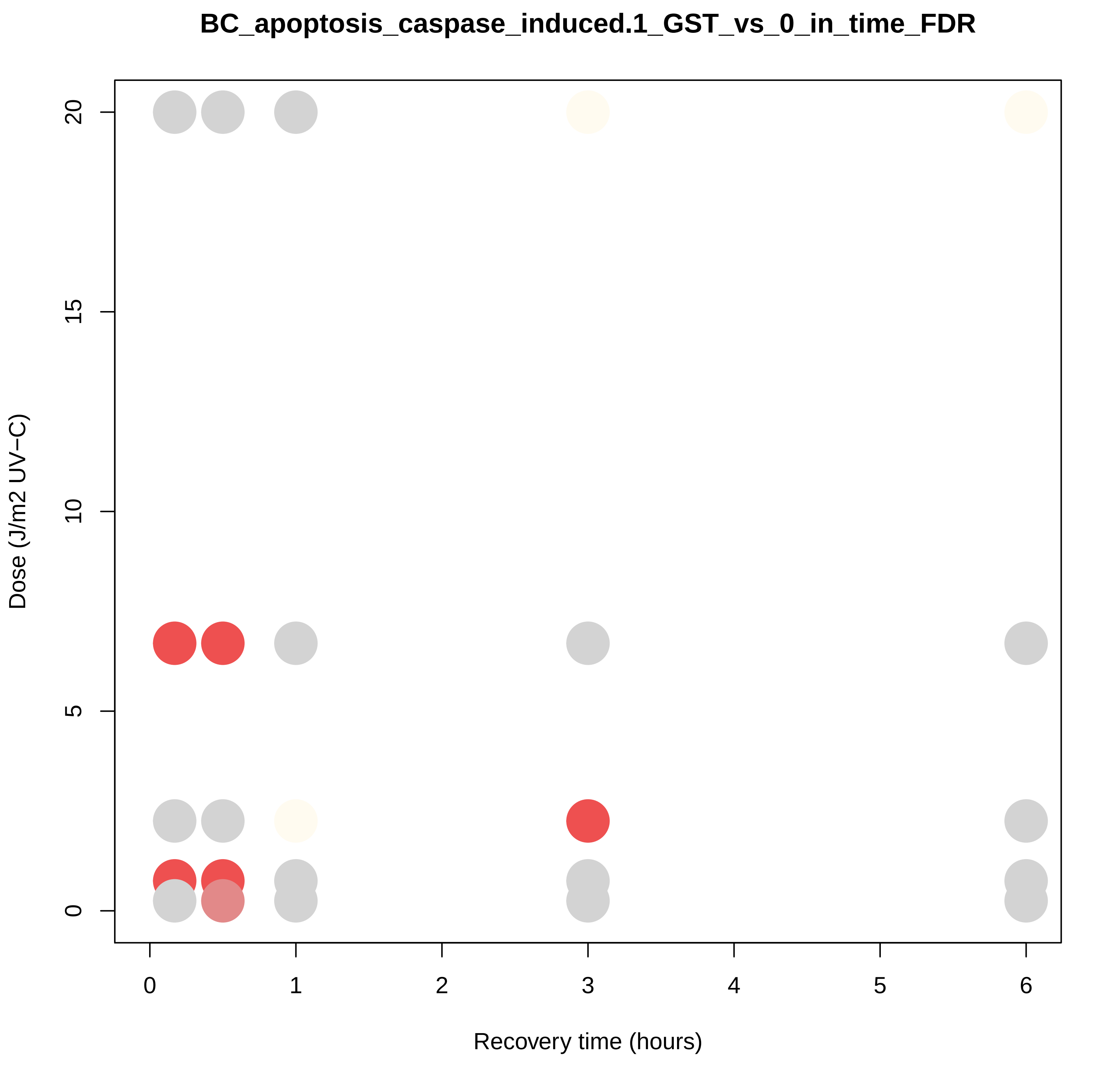

BC_apoptosis_caspase_induced.1_GST_vs_0_in_time_FDR
Dose (J/m2 UV−C)
0
5
10
15
20
Recovery time (hours)
0
1
2
3
4
5
6

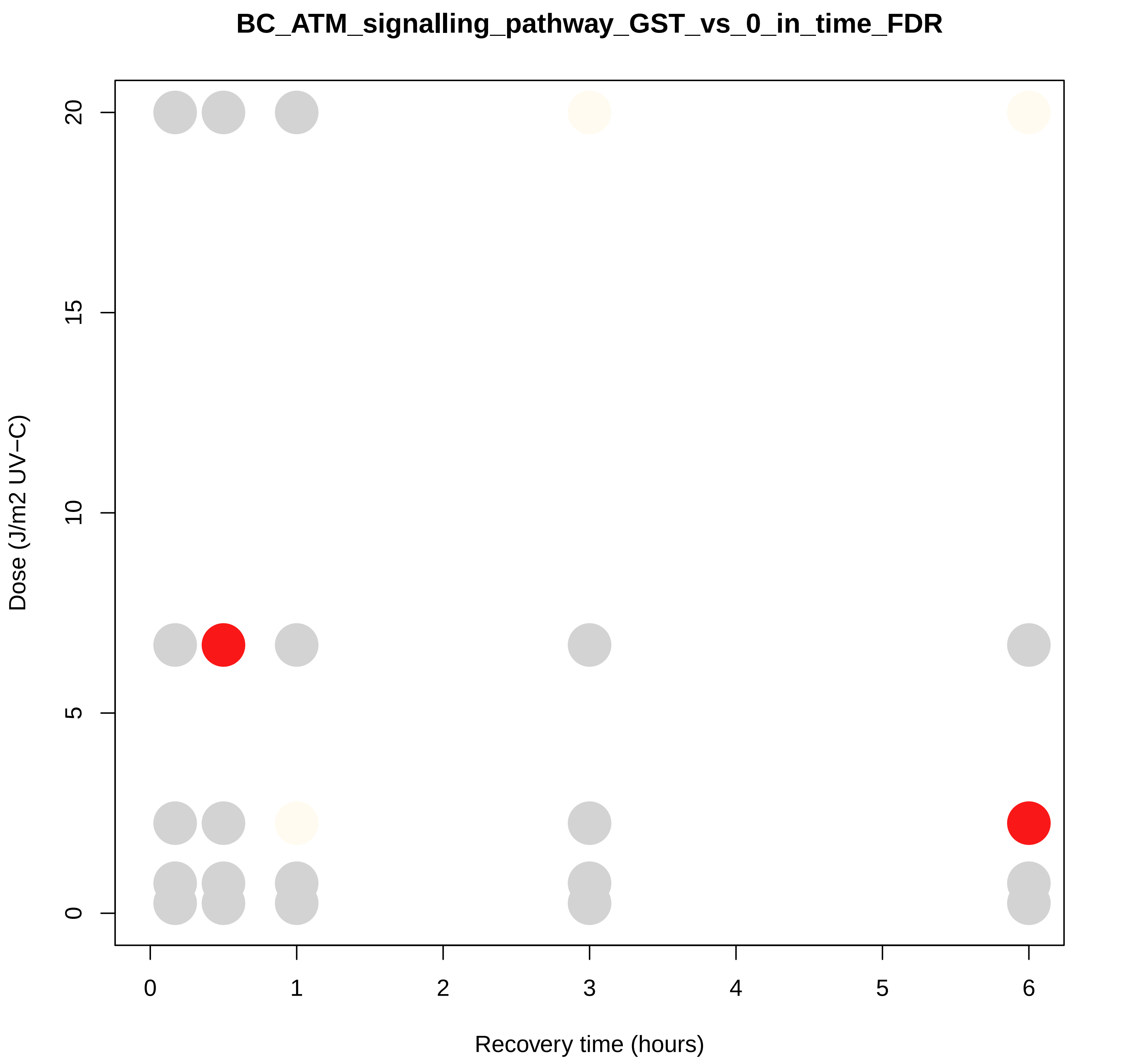
BC_ATM_signalling_pathway_GST_vs_0_in_time_FDR
Dose (J/m2 UV−C)
0
5
10
15
20
Recovery time (hours)
0
1
2
3
4
5
6

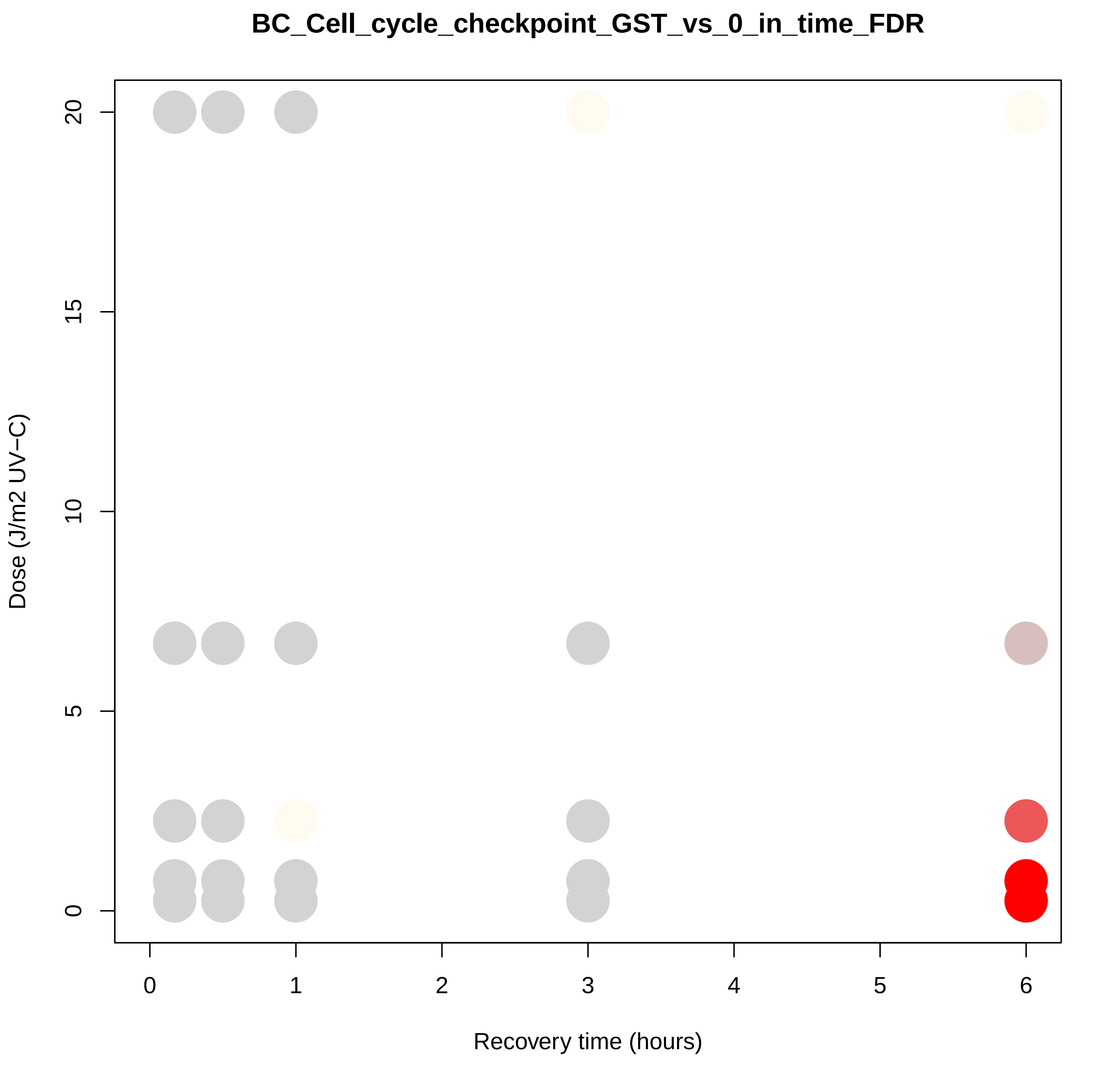

BC_Cell_cycle_checkpoint_GST_vs_0_in_time_FDR
Dose (J/m2 UV−C)
0
5
10
15
20
0
1
2
3
4
5
6
Recovery time (hours)

BC_Cyclins_and_Cell_Cycle_Regulation_pathway_GST_vs_0_in_time_FDR

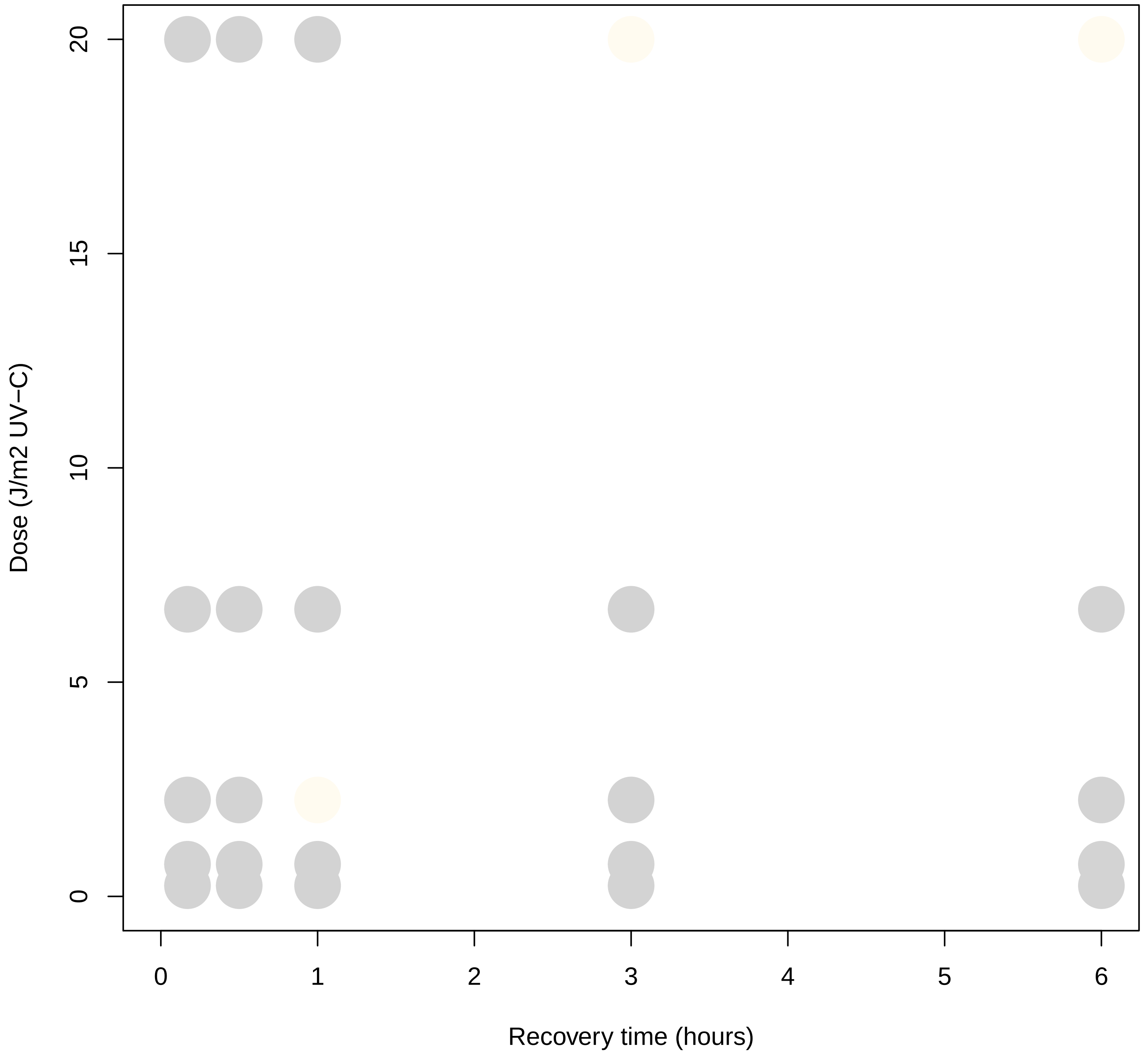

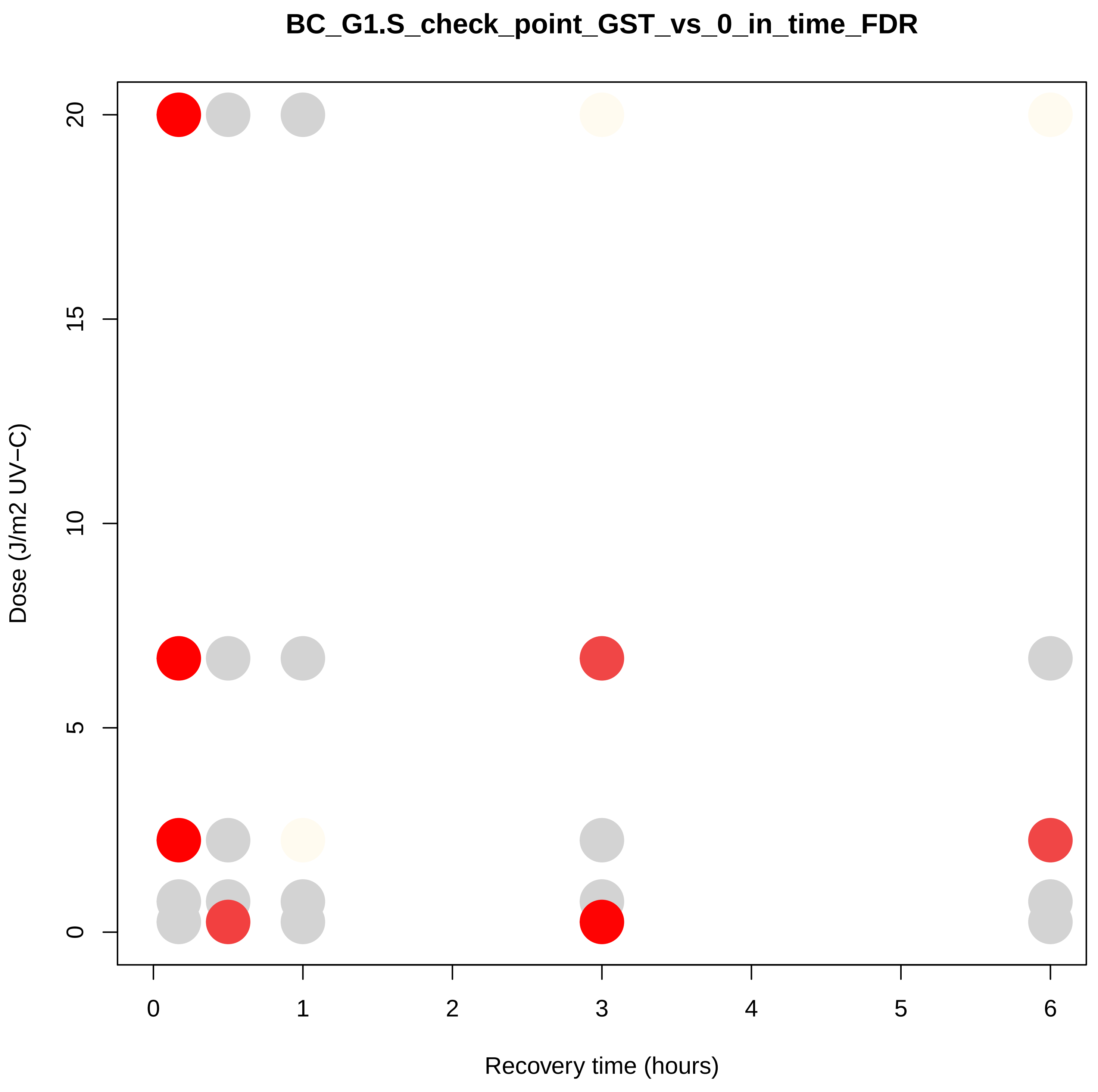
BC_G1.S_check_point_GST_vs_0_in_time_FDR
Dose (J/m2 UV−C)
Recovery time (hours)
0
5
10
15
20
0
1
2
3
4
5
6

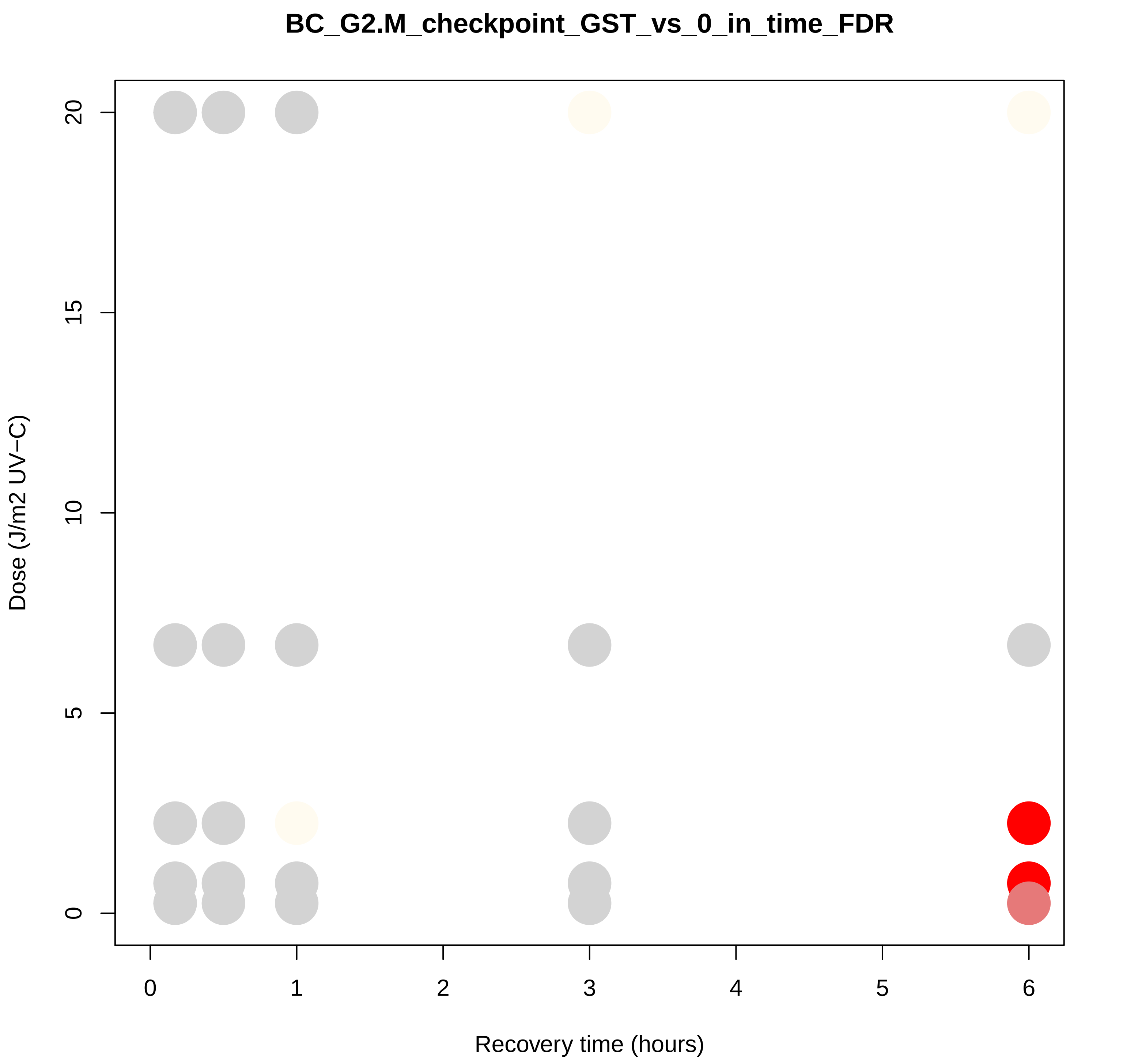
BC_G2.M_checkpoint_GST_vs_0_in_time_FDR
Dose (J/m2 UV−C)
Recovery time (hours)
0
5
10
15
20
0
1
2
3
4
5
6

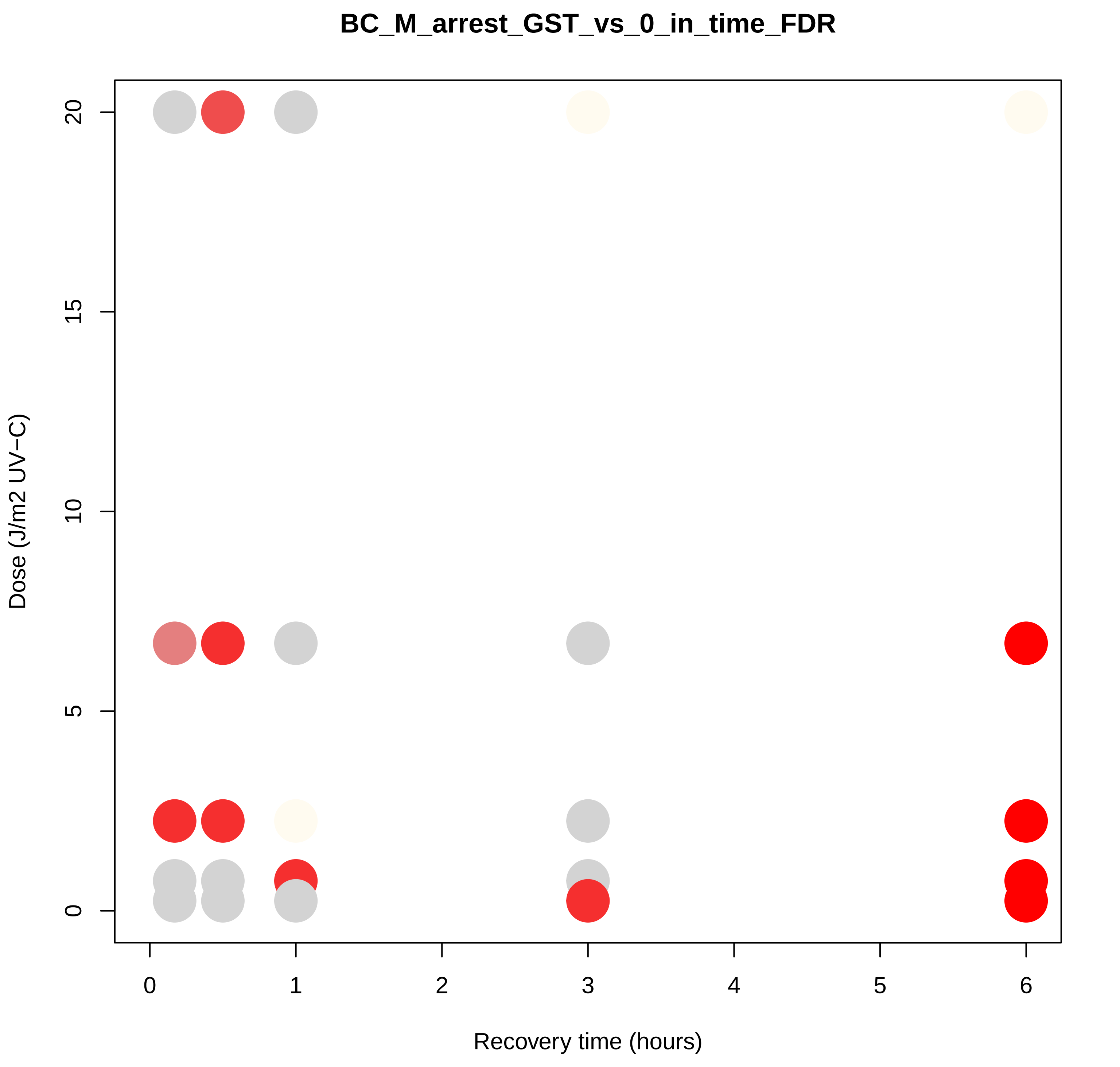

BC_M_arrest_GST_vs_0_in_time_FDR
Dose (J/m2 UV−C)
Recovery time (hours)
0
5
10
15
20
0
1
2
3
4
5
6

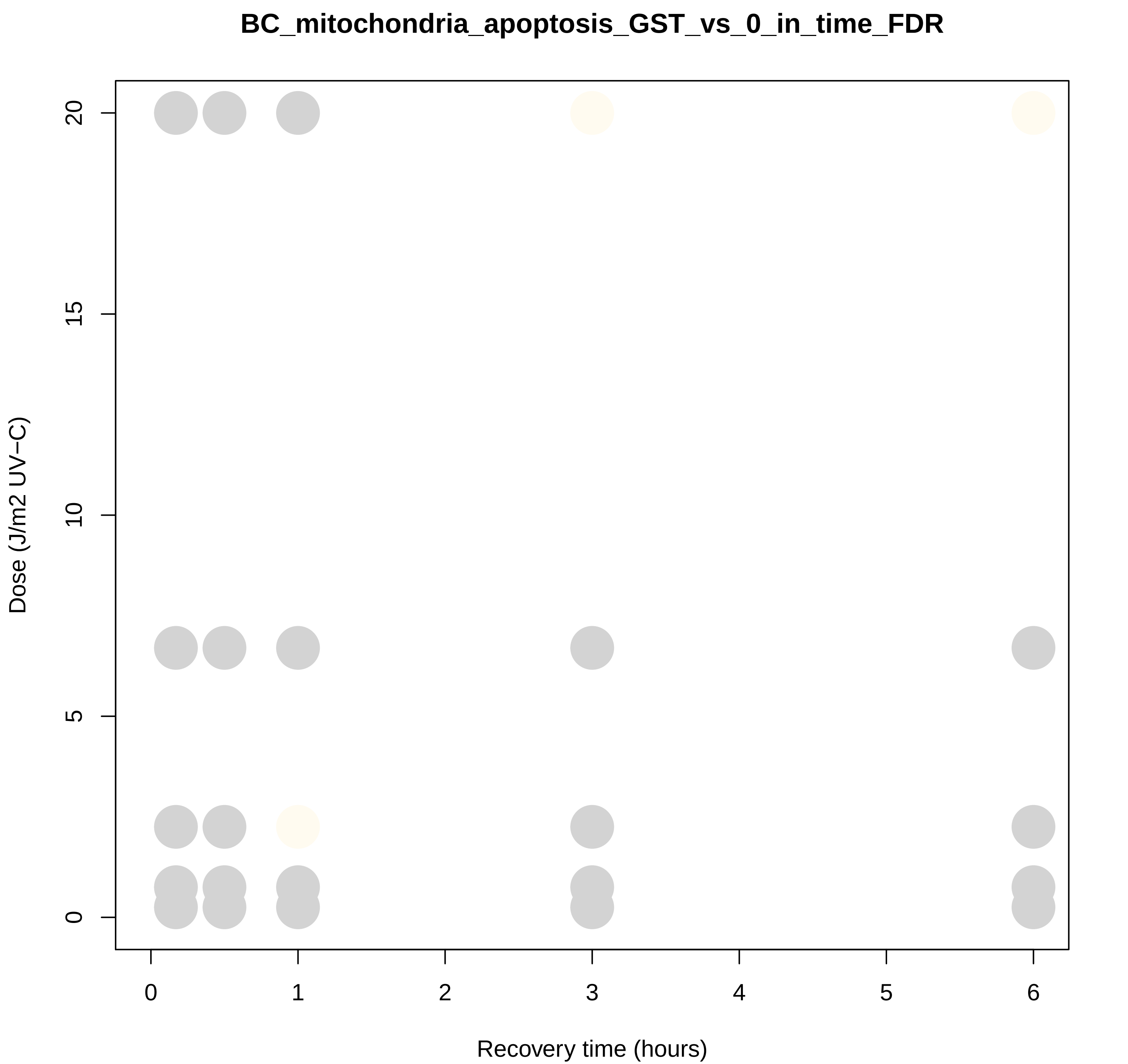
BC_mitochondria_apoptosis_GST_vs_0_in_time_FDR
Dose (J/m2 UV−C)
0
5
10
15
20
Recovery time (hours)
0
1
2
3
4
5
6

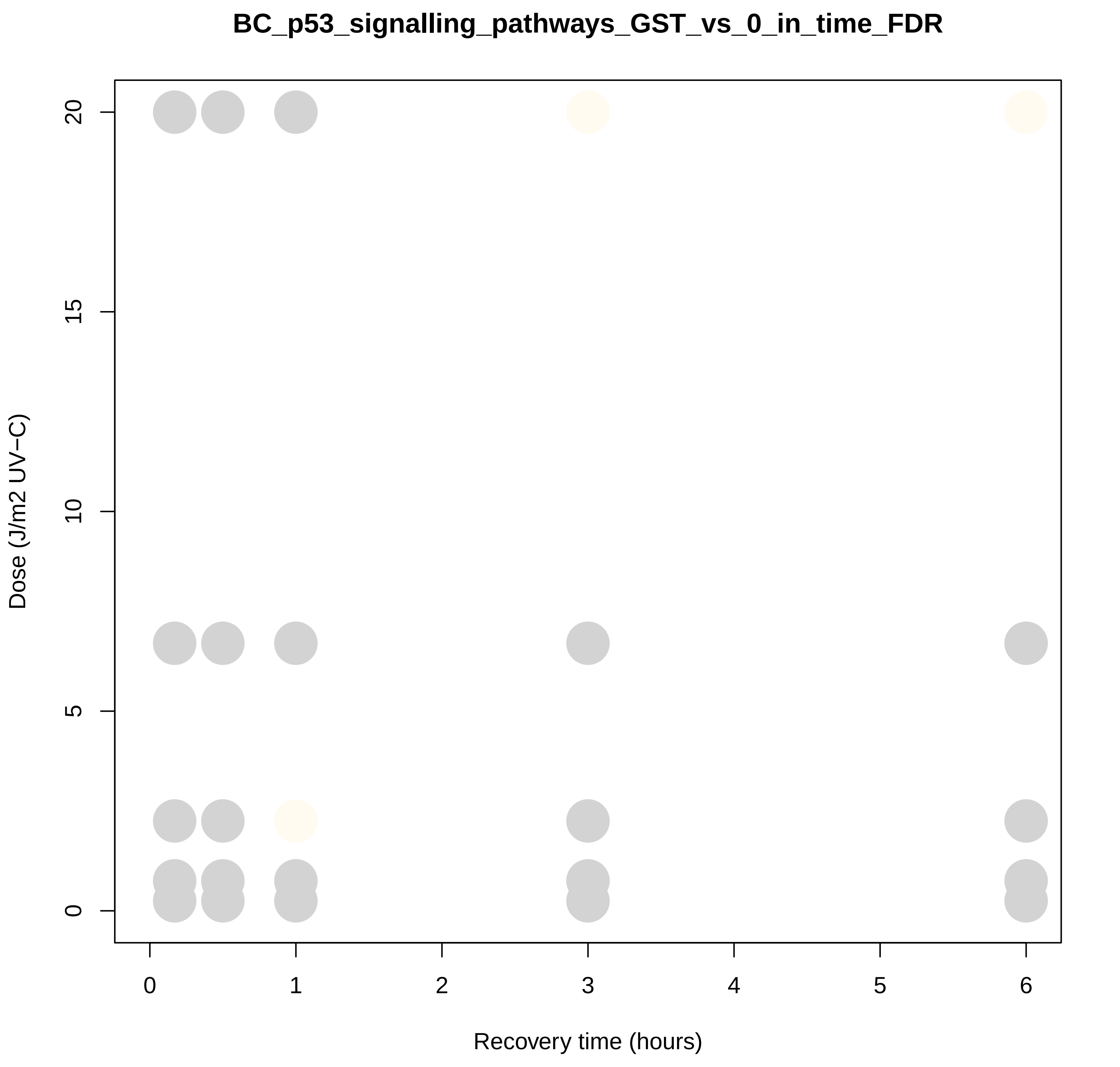

BC_p53_signalling_pathways_GST_vs_0_in_time_FDR
Dose (J/m2 UV−C)
0
5
10
15
20
Recovery time (hours)
0
1
2
3
4
5
6

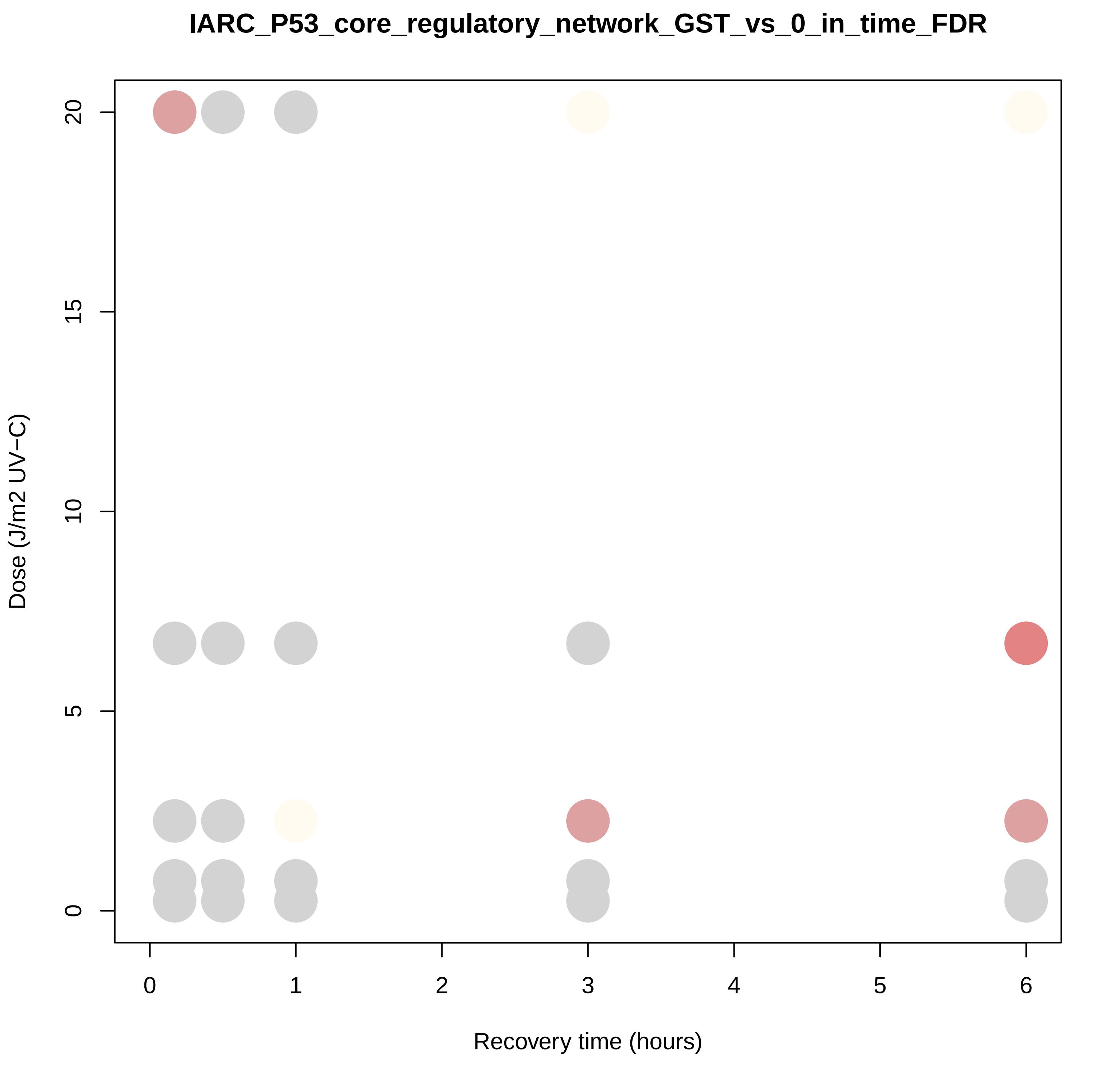

IARC_P53_core_regulatory_network_GST_vs_0_in_time_FDR
Dose (J/m2 UV−C)
0
5
10
15
20
0
1
2
3
4
5
6
Recovery time (hours)

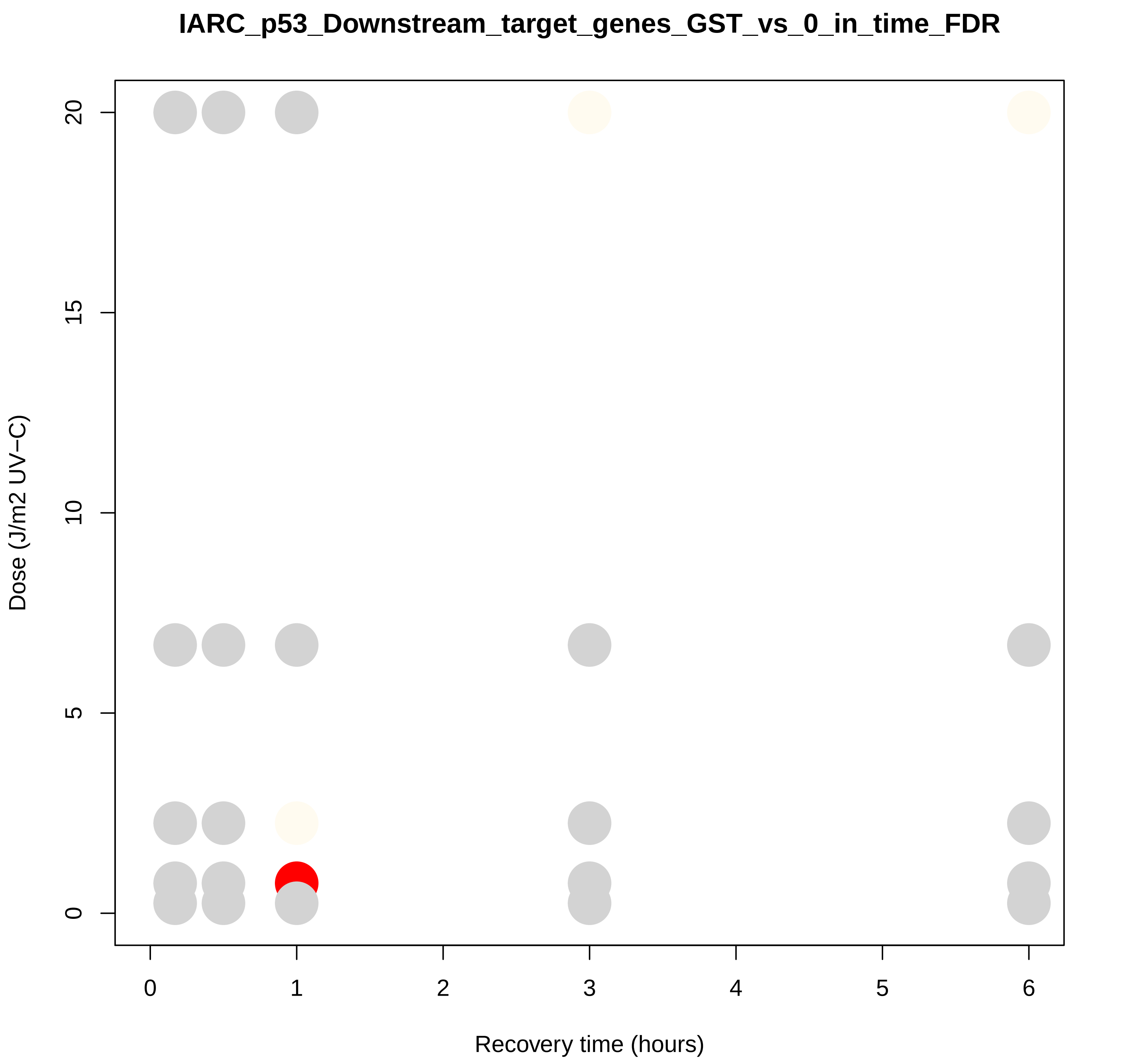
IARC_p53_Downstream_target_genes_GST_vs_0_in_time_FDR
Dose (J/m2 UV−C)
0
5
10
15
20
0
1
2
3
4
5
6
Recovery time (hours)

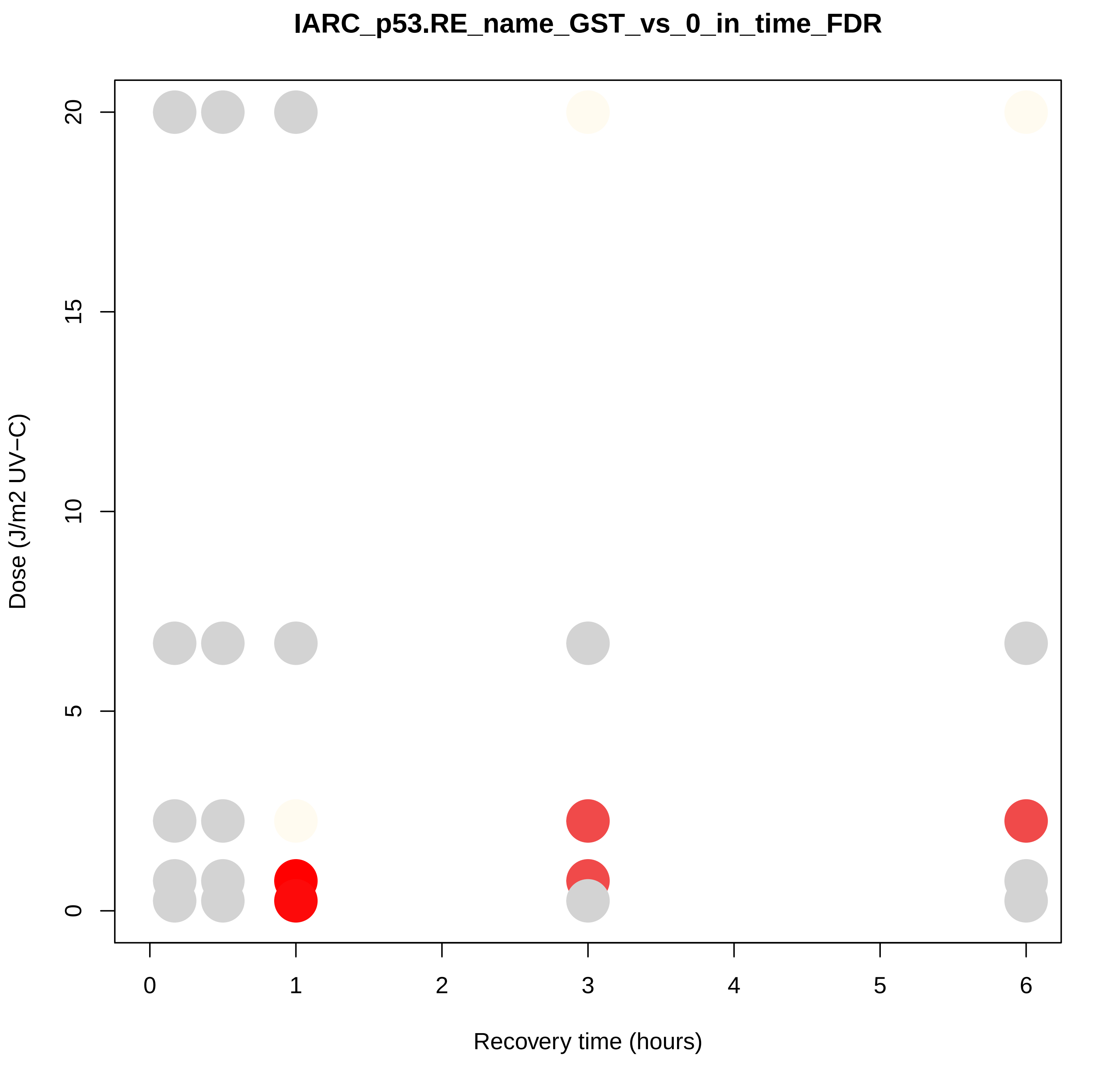
IARC_p53.RE_name_GST_vs_0_in_time_FDR
Dose (J/m2 UV−C)
0
5
10
15
20
Recovery time (hours)
0
1
2
3
4
5
6

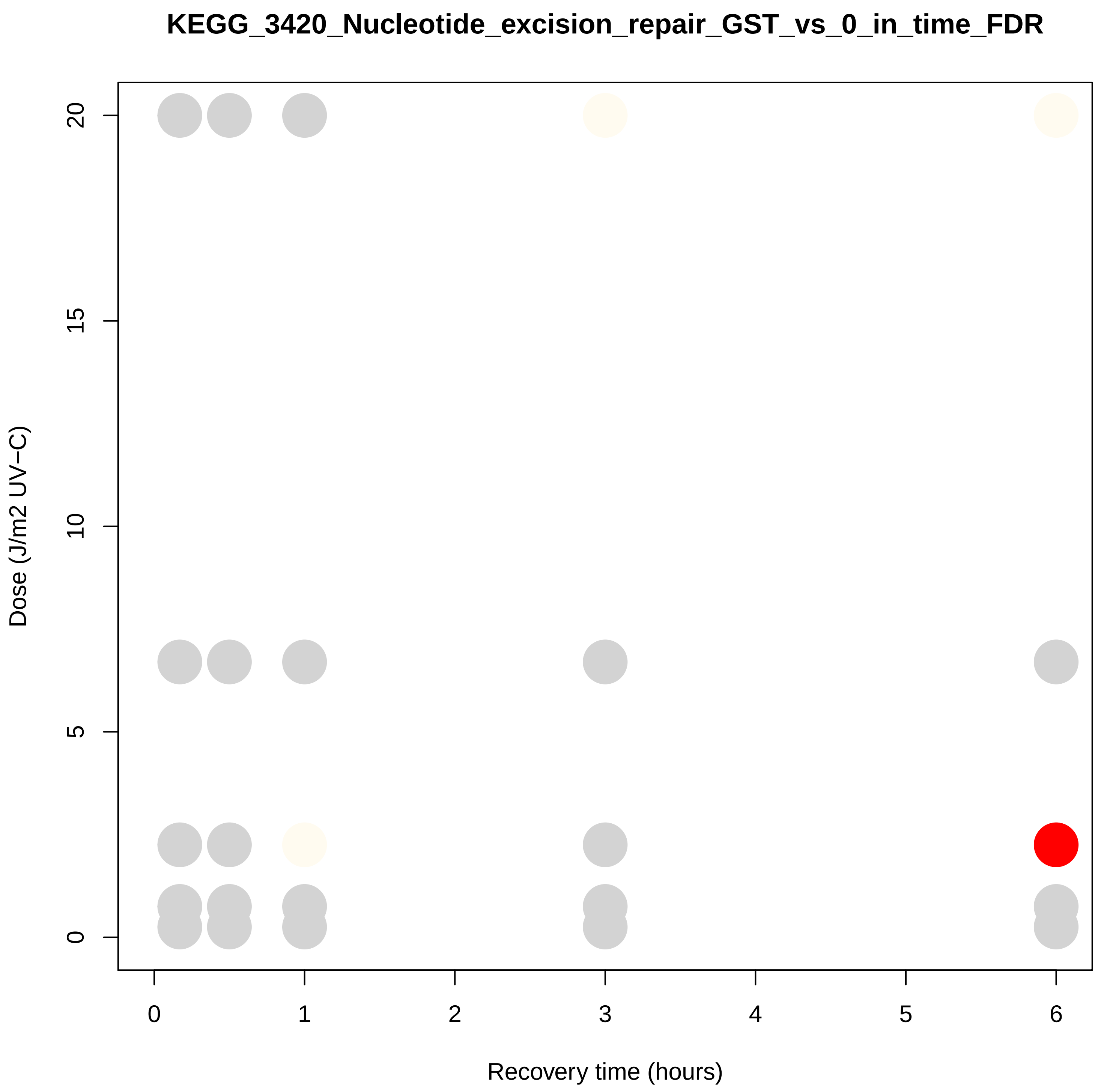
KEGG_3420_Nucleotide_excision_repair_GST_vs_0_in_time_FDR
Dose (J/m2 UV−C)
0
5
10
15
20
0
1
2
3
4
5
6
Recovery time (hours)

C_maps_apoptosis_and_survival_DNA_damage_induced_apoptosis_GST_vs_0_in_tim

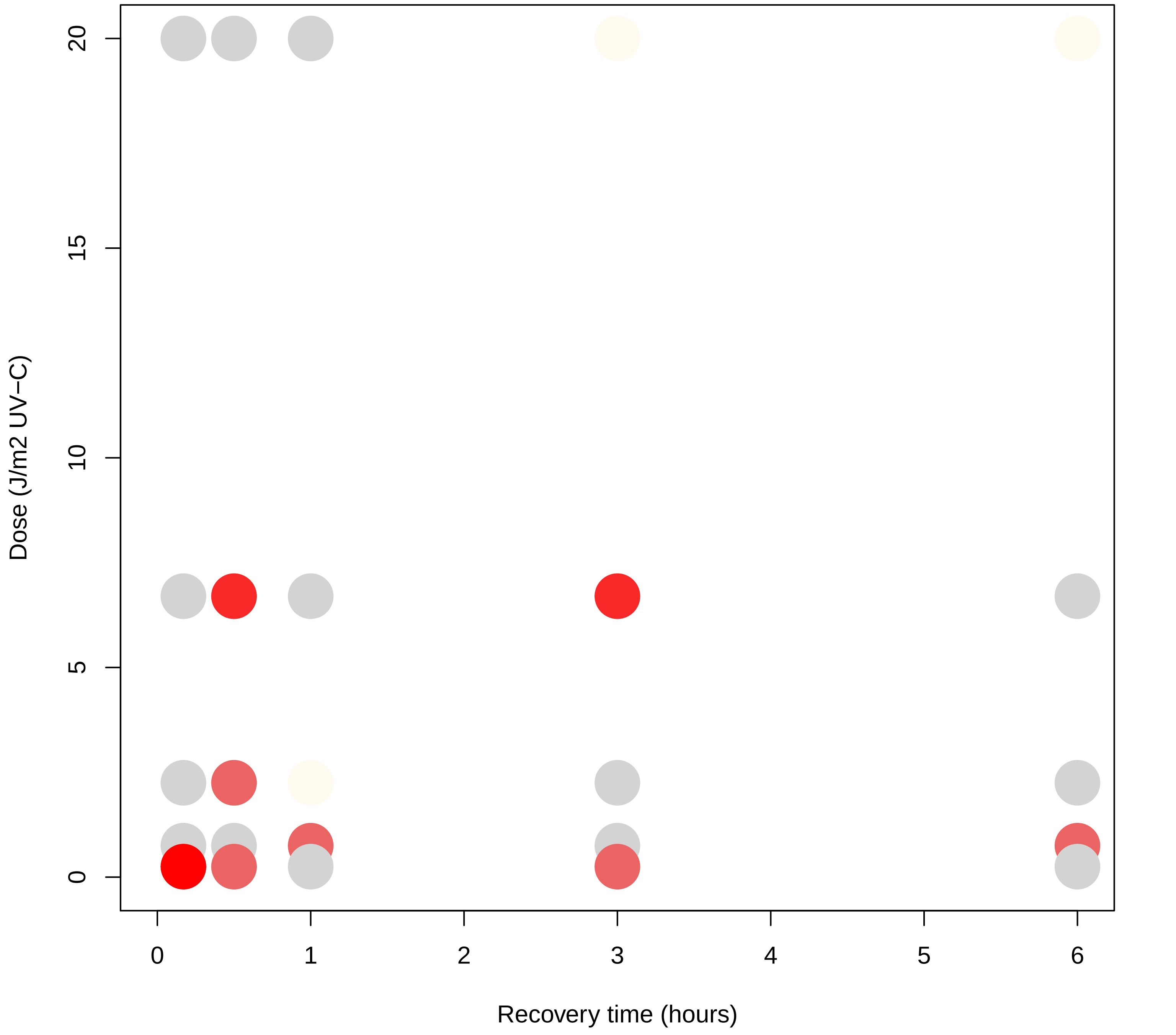

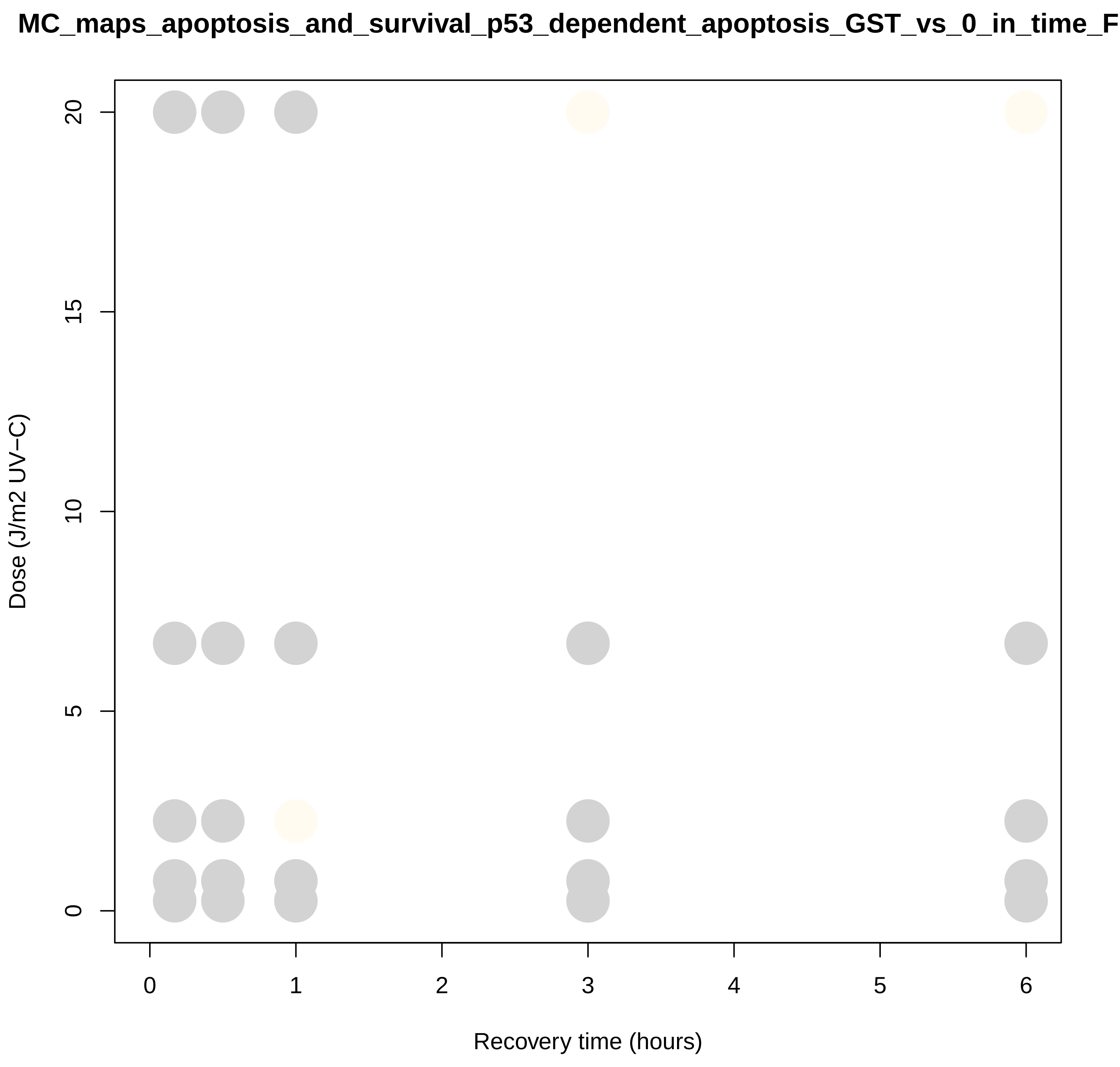

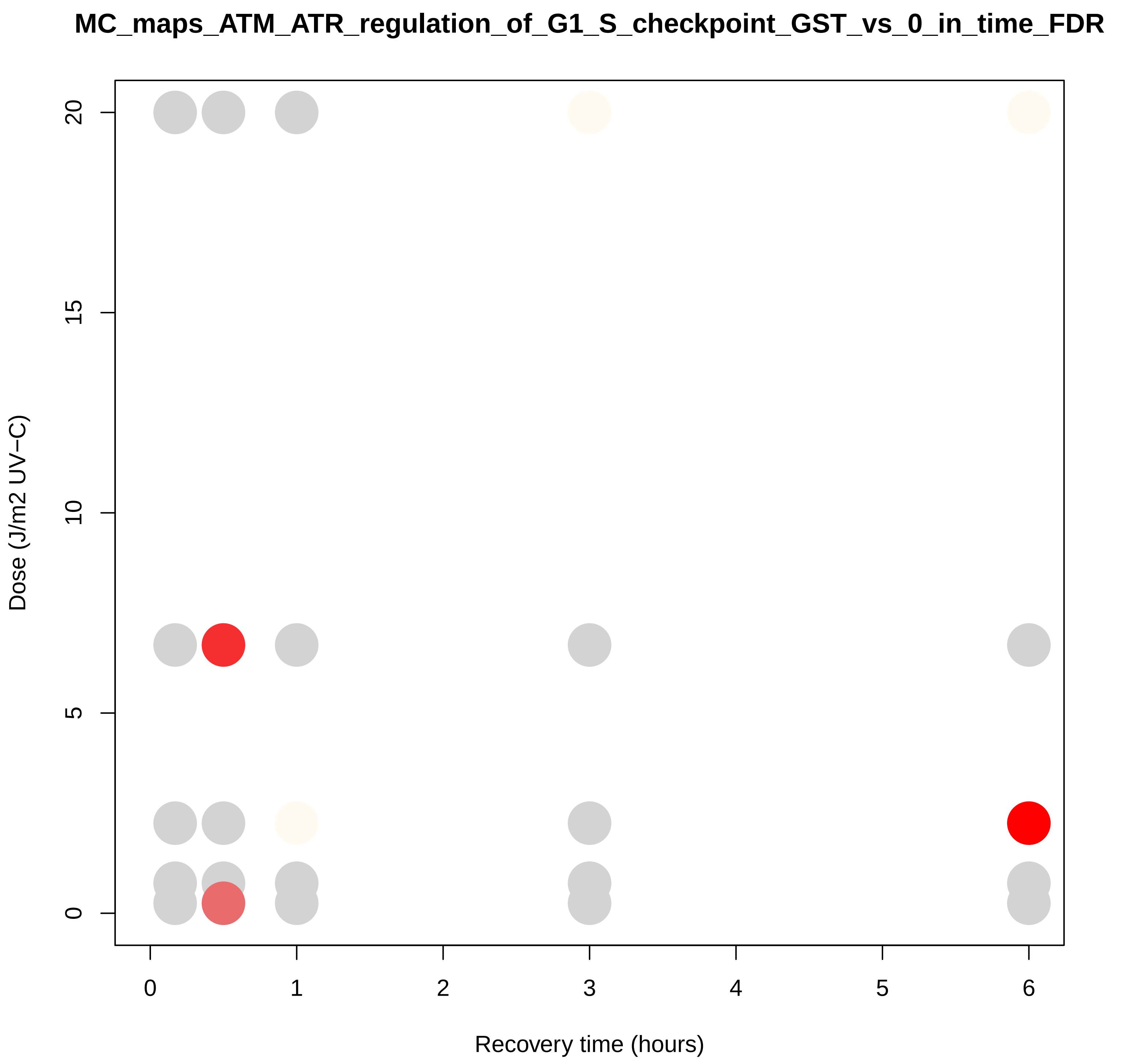

MC_maps_ATM_ATR_regulation_of_G1_S_checkpoint_GST_vs_0_in_time_FDR
Dose (J/m2 UV−C)
Recovery time (hours)
0
5
10
15
20
0
1
2
3
4
5
6

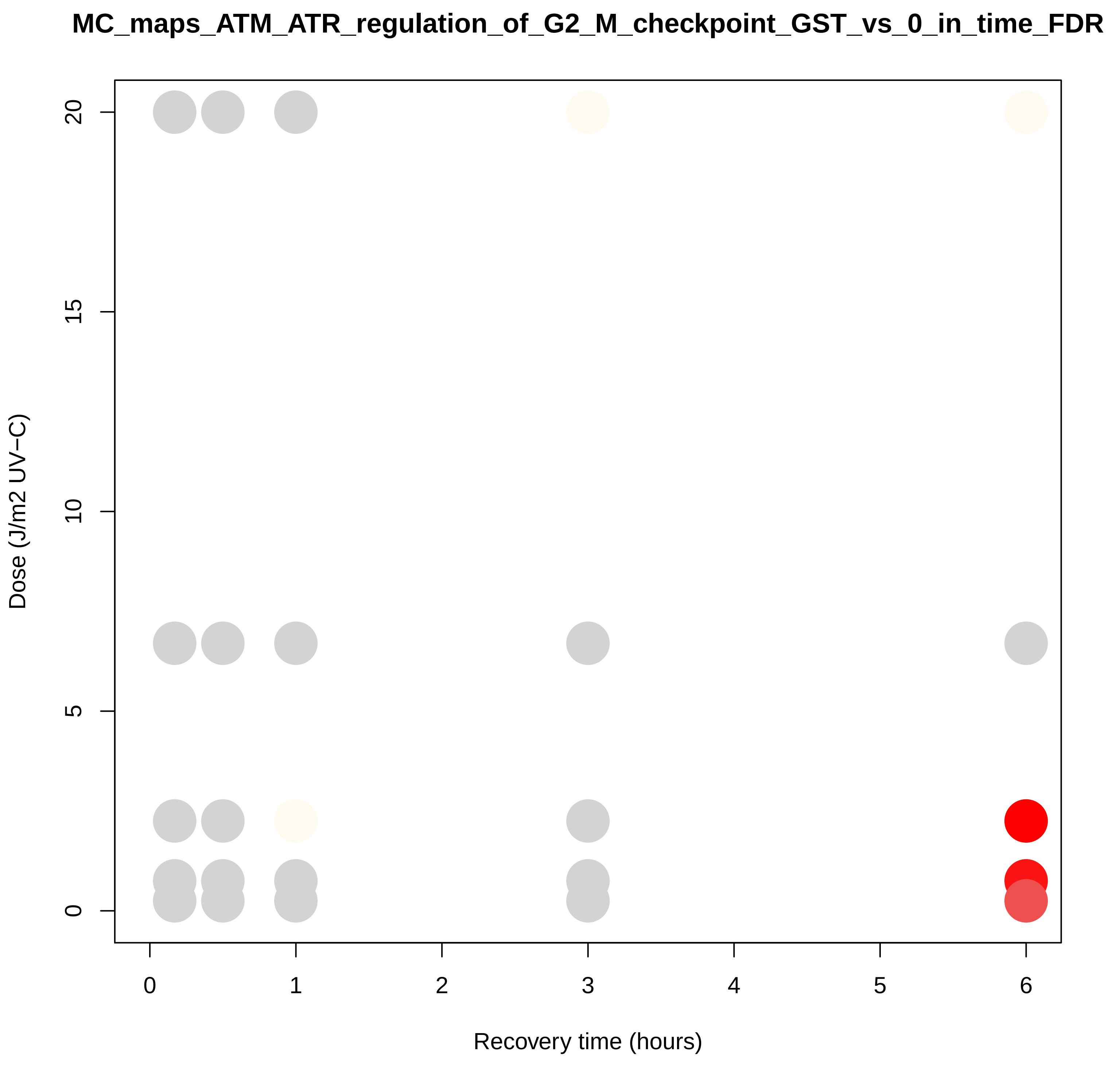

MC_maps_ATM_ATR_regulation_of_G2_M_checkpoint_GST_vs_0_in_time_FDR
Dose (J/m2 UV−C)
0
5
10
15
20
0
1
2
3
4
5
6
Recovery time (hours)

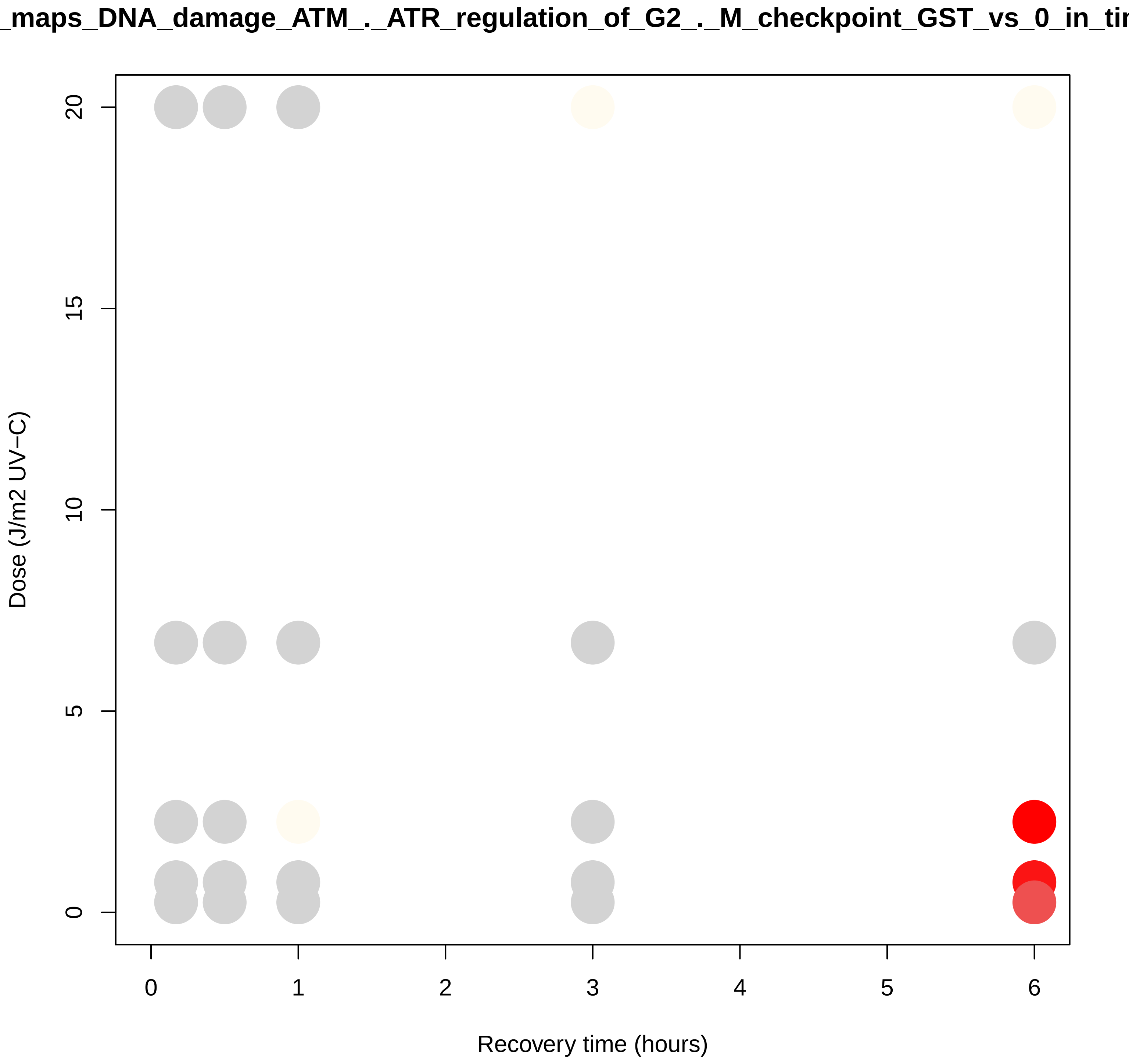
_maps_DNA_damage_ATM_._ATR_regulation_of_G2_._M_checkpoint_GST_vs_0_in_tir
Dose (J/m2 UV−C)
0
5
10
15
20
0
1
2
3
4
5
6
Recovery time (hours)

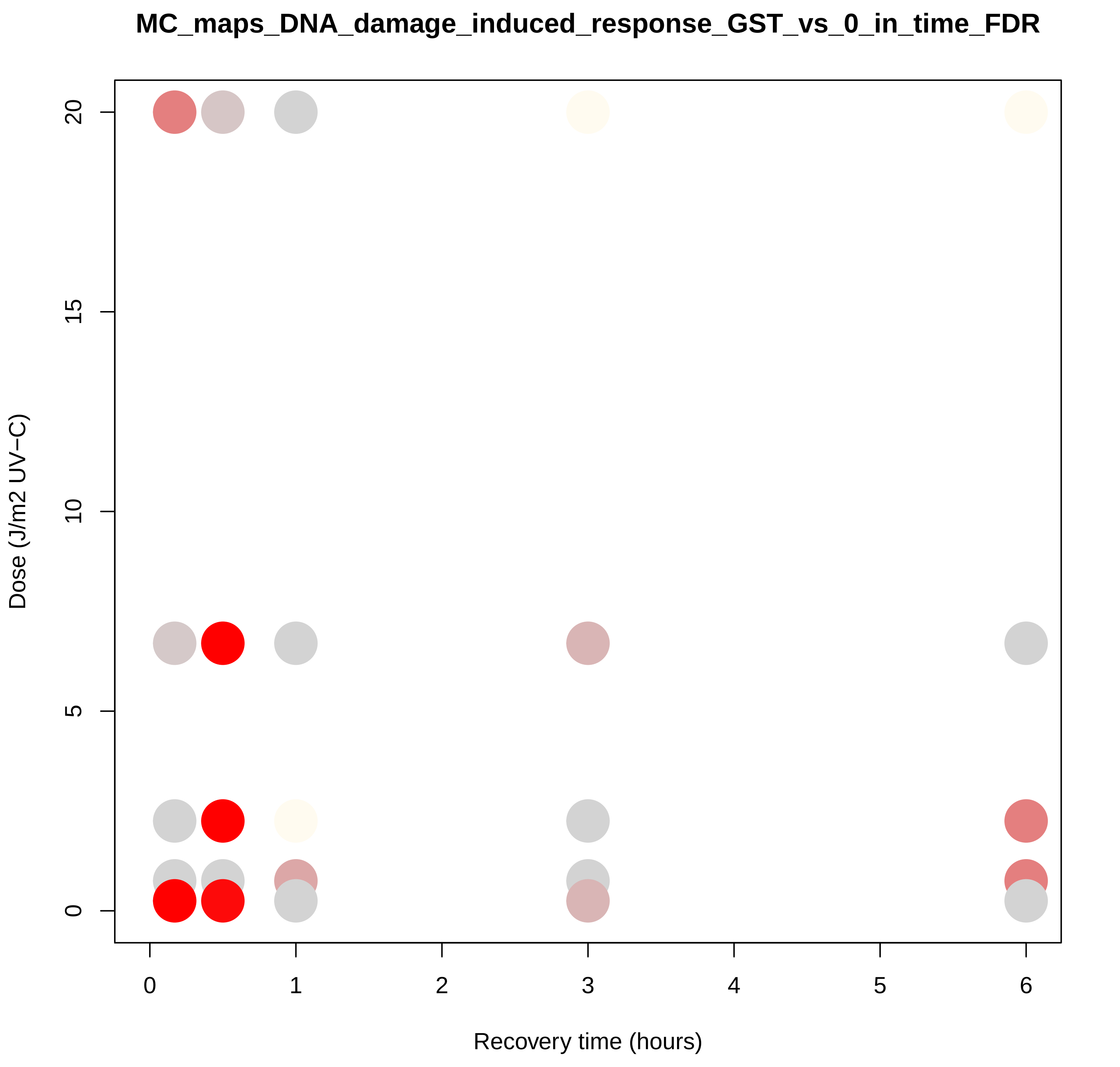
MC_maps_DNA_damage_induced_response_GST_vs_0_in_time_FDR
Dose (J/m2 UV−C)
0
5
10
15
20
0
1
2
3
4
5
6
Recovery time (hours)

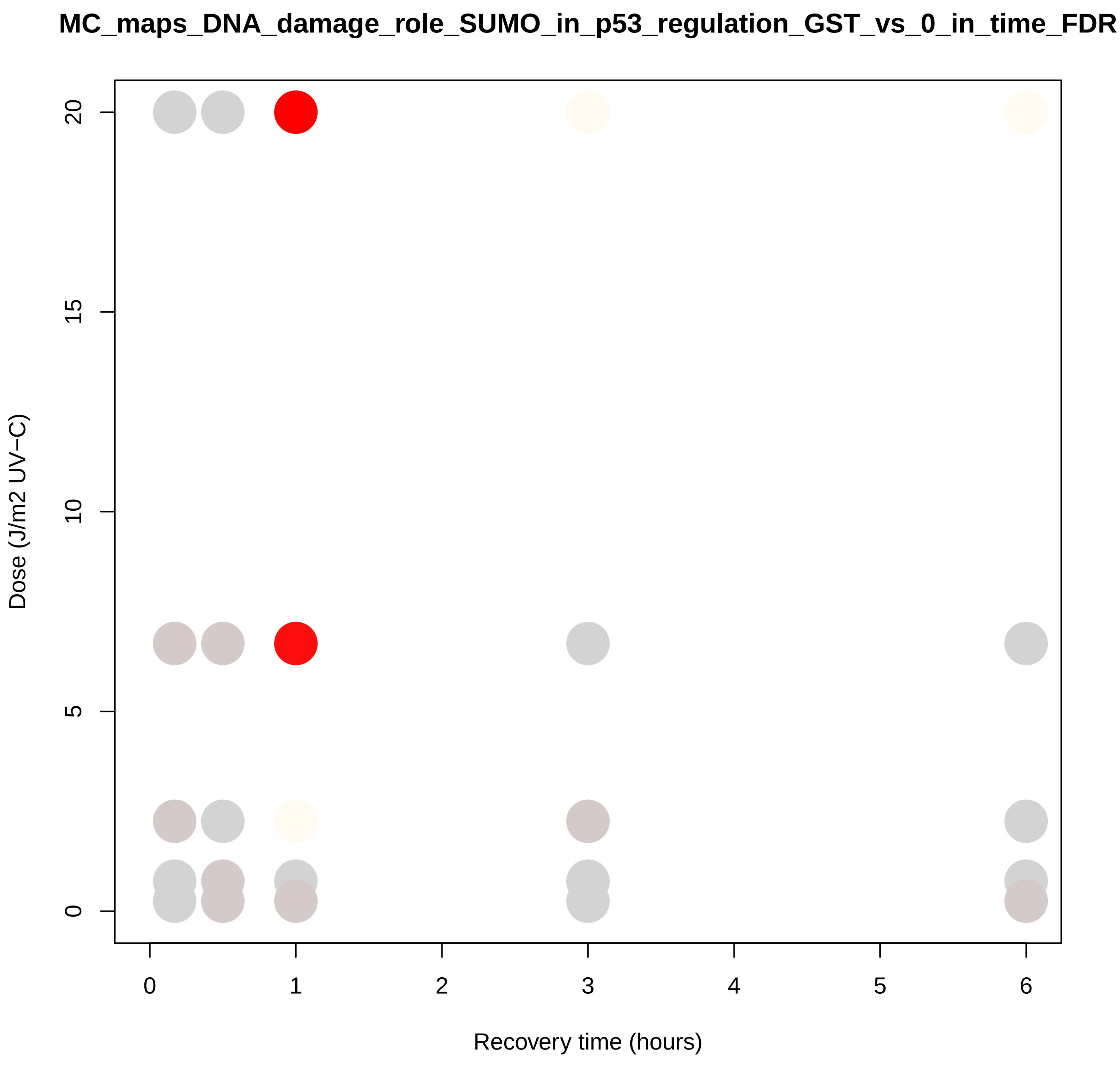

MC_maps_DNA_damage_role_SUMO_in_p53_regulation_GST_vs_0_in_time_FDR
Dose (J/m2 UV−C)
Recovery time (hours)
0
5
10
15
20
0
1
2
3
4
5
6

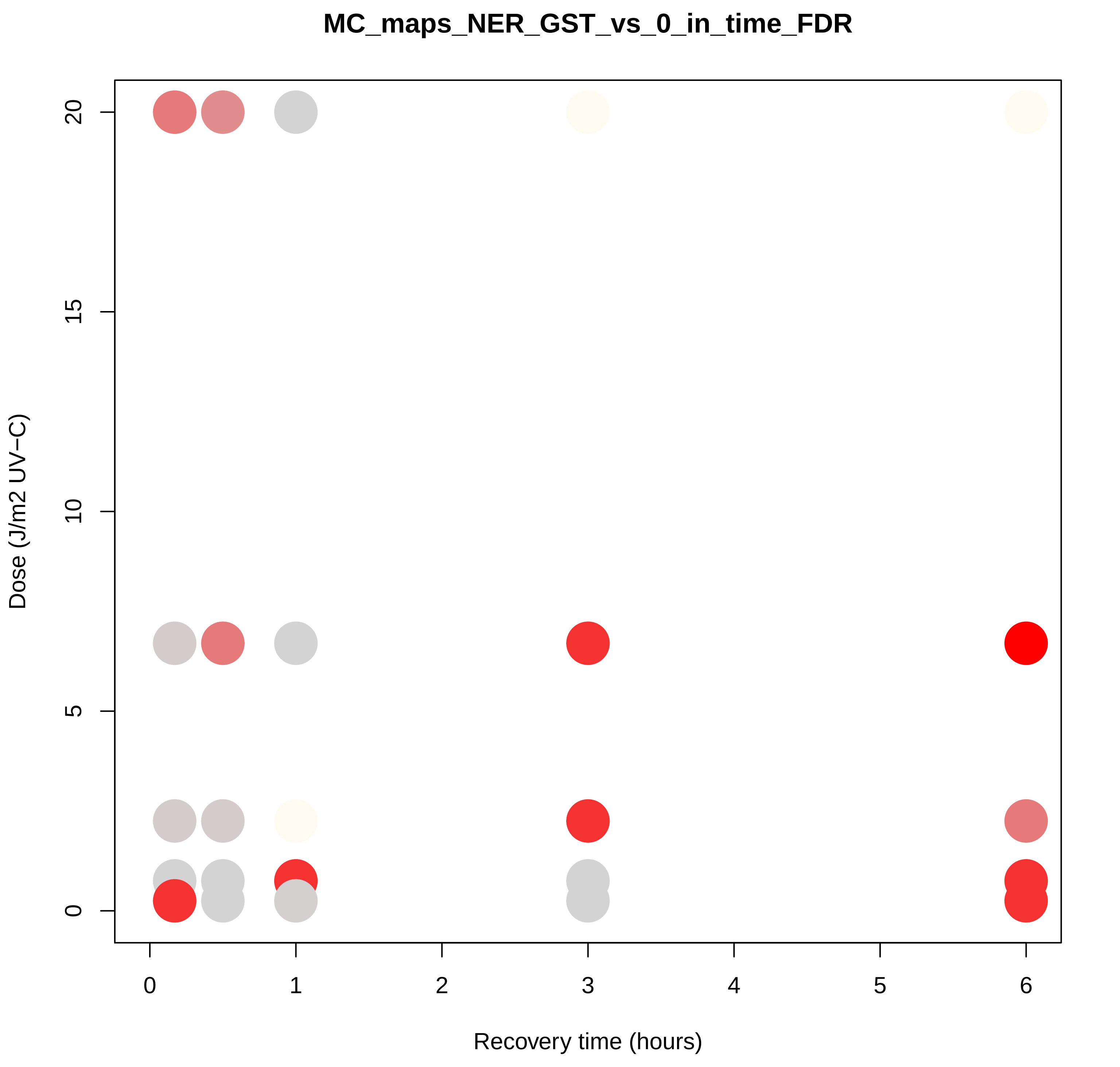
MC_maps_NER_GST_vs_0_in_time_FDR
Dose (J/m2 UV−C)
Recovery time (hours)
0
5
10
15
20
0
1
2
3
4
5
6

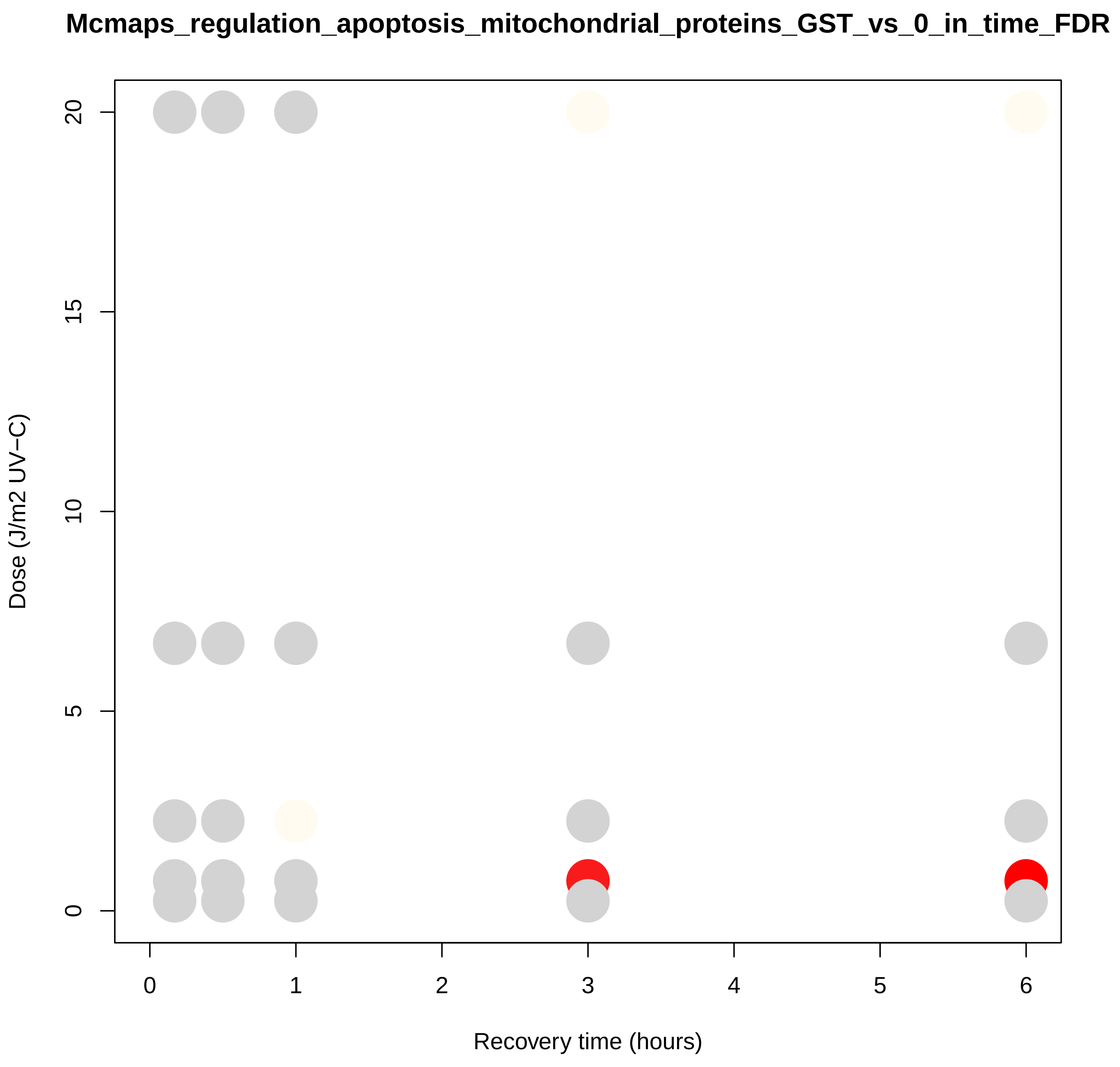
Mcmaps_regulation_apoptosis_mitochondrial_proteins_GST_vs_0_in_time_FDR
Dose (J/m2 UV−C)
0
5
10
15
20
0
1
2
3
4
5
6
Recovery time (hours)

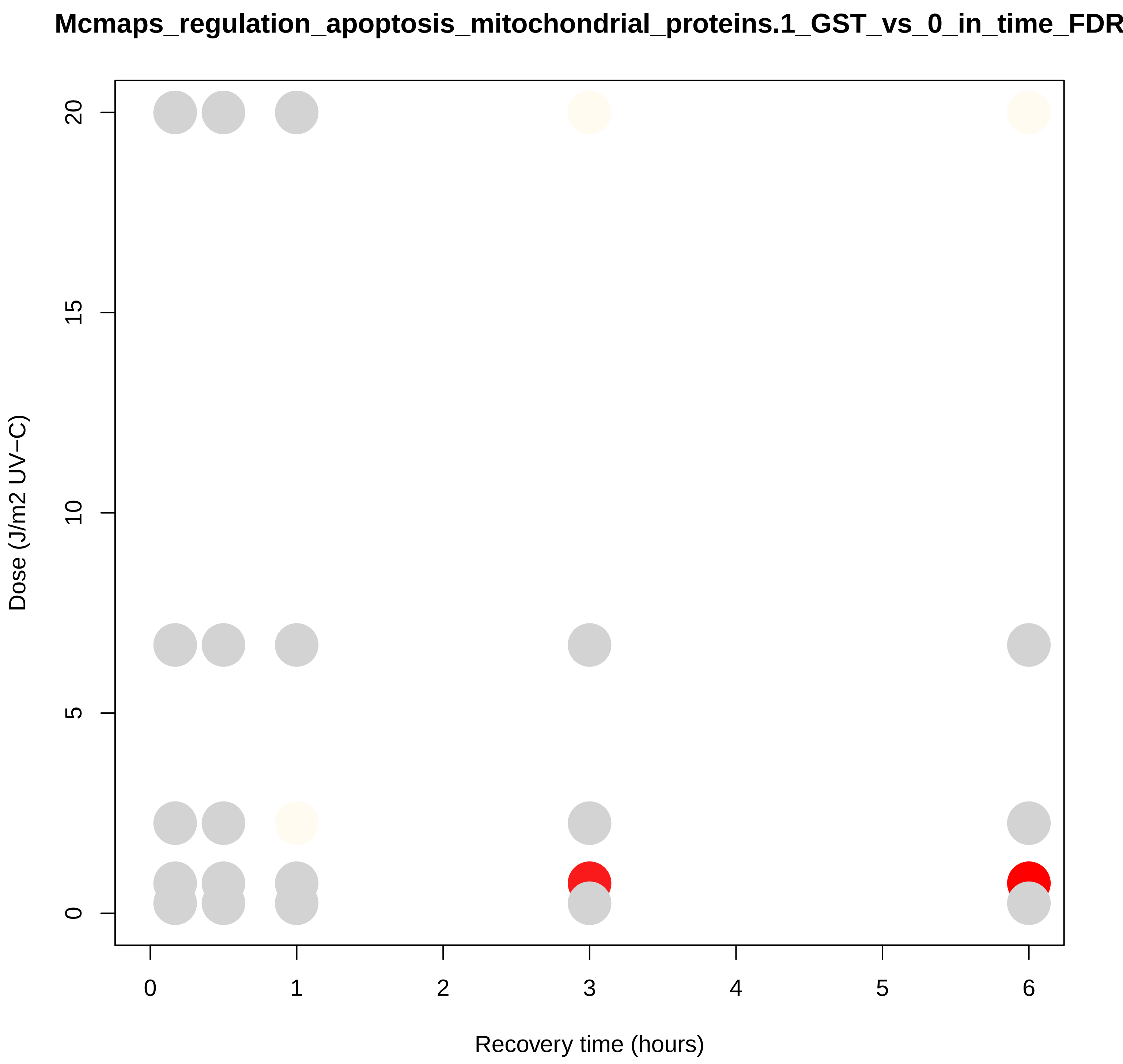

Mcmaps_regulation_apoptosis_mitochondrial_proteins.1_GST_vs_0_in_time_FDR
Dose (J/m2 UV−C)
Recovery time (hours)
0
1
2
3
4
5
6
0
5
10
15
20

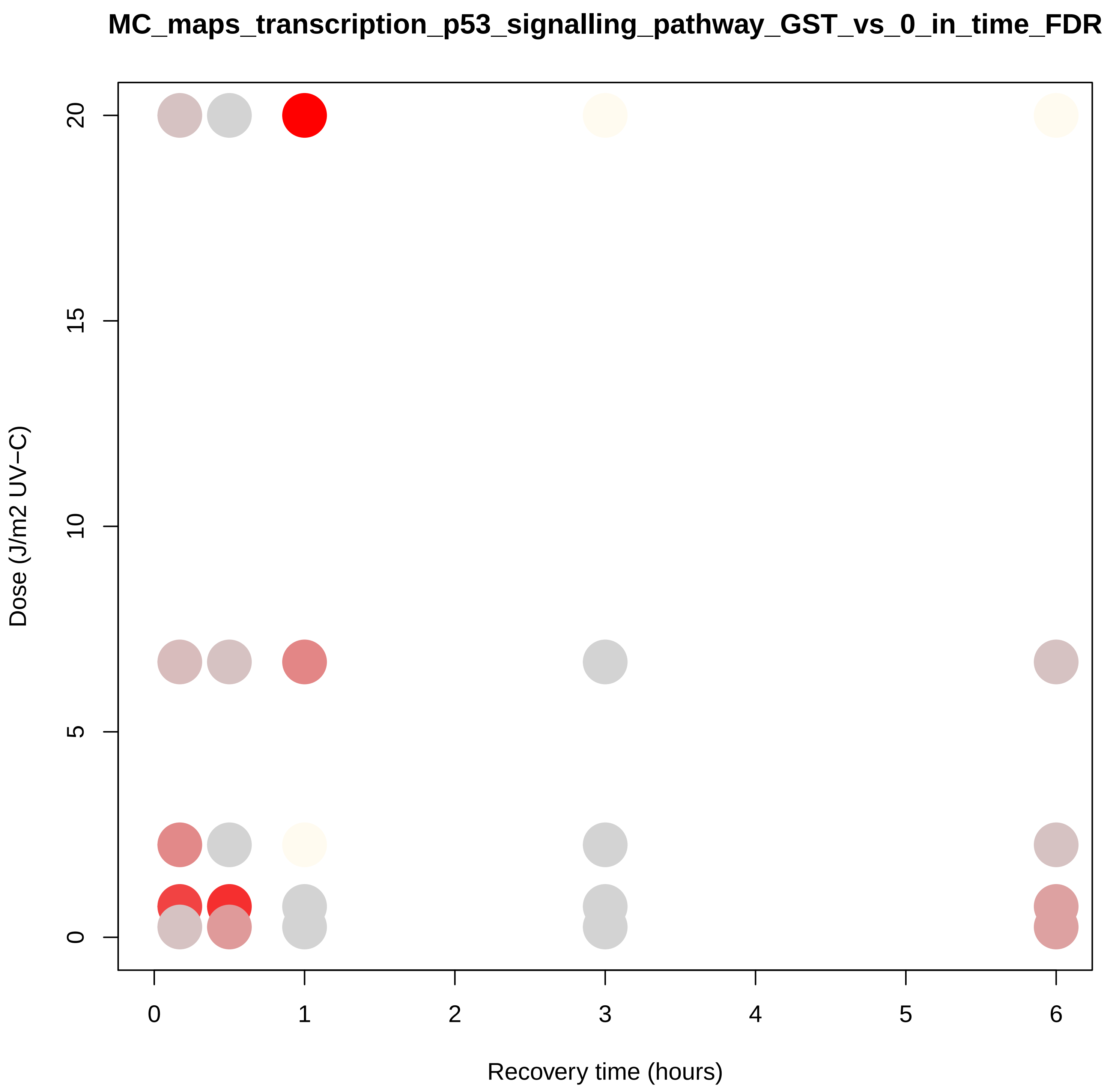
MC_maps_transcription_p53_signalling_pathway_GST_vs_0_in_time_FDR
Dose (J/m2 UV−C)
Recovery time (hours)
0
5
10
15
20
0
1
2
3
4
5
6

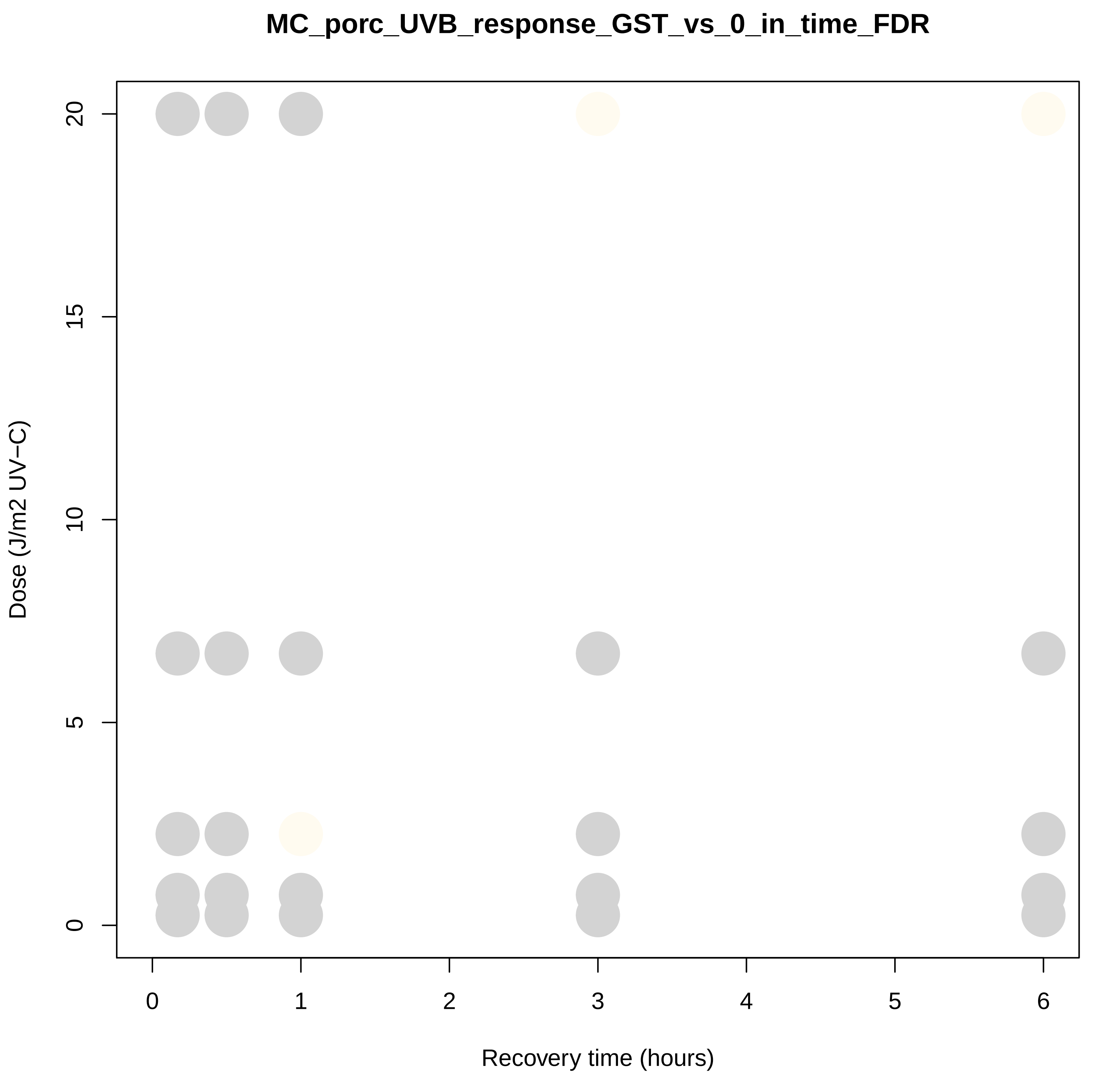
MC_porc_UVB_response_GST_vs_0_in_time_FDR
Dose (J/m2 UV−C)
Recovery time (hours)
0
5
10
15
20
0
1
2
3
4
5
6

ss_DNA_damage_response._signal_transduction_by_p53._cell_cycle_arrest_GST_vs_(

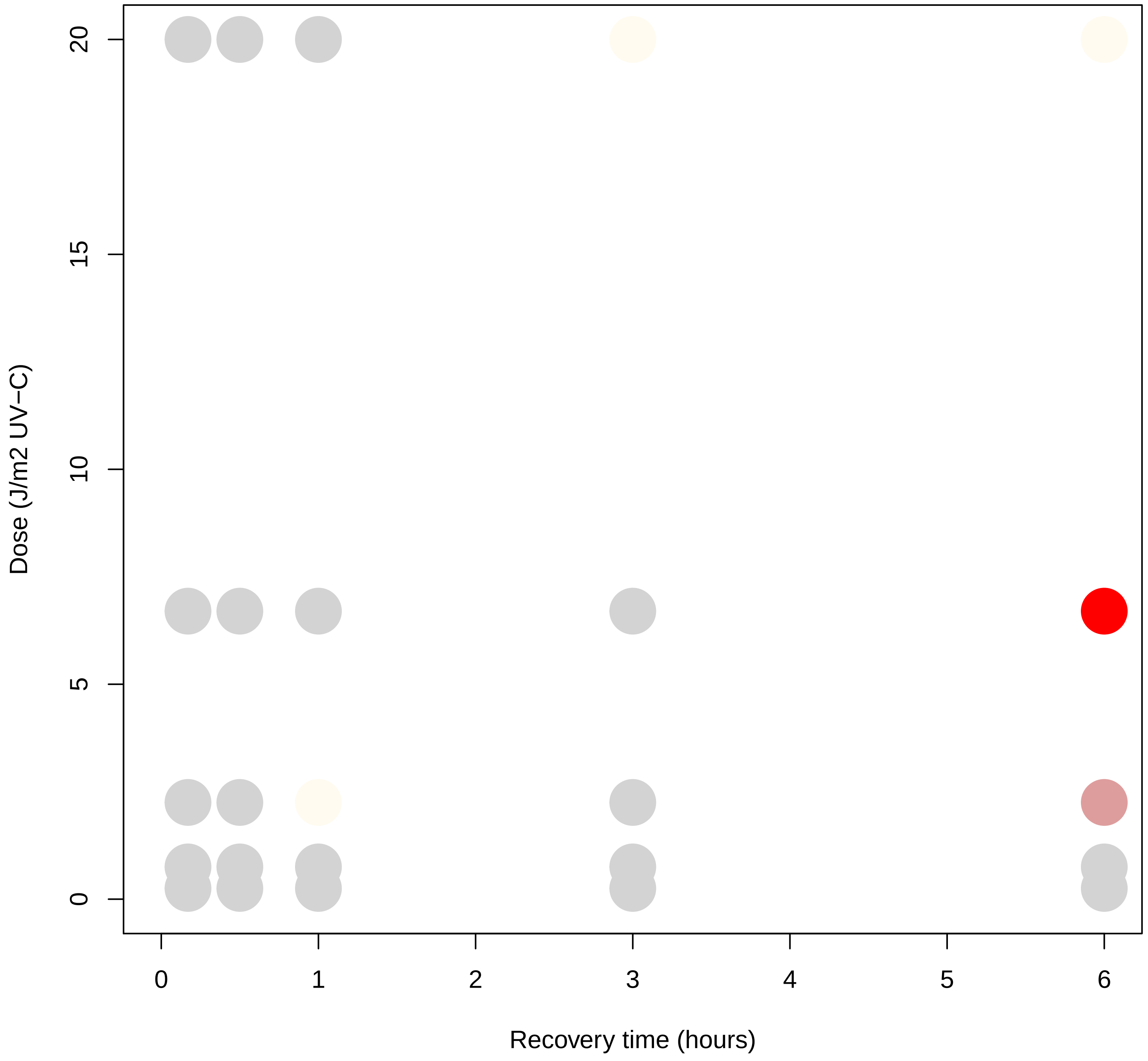

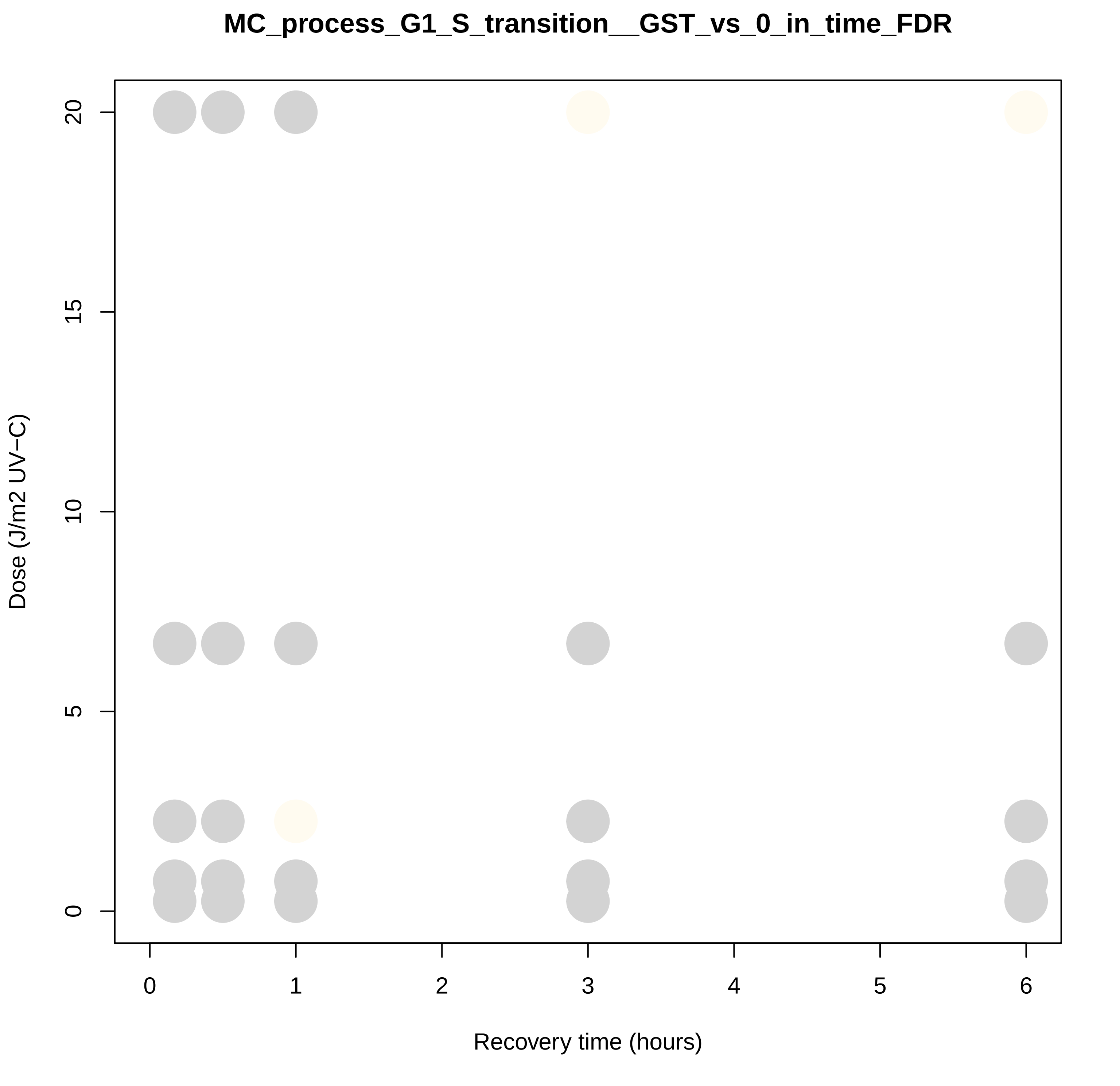

MC_process_G1_S_transition__GST_vs_0_in_time_FDR
Dose (J/m2 UV−C)
0
5
10
15
20
Recovery time (hours)
0
1
2
3
4
5
6

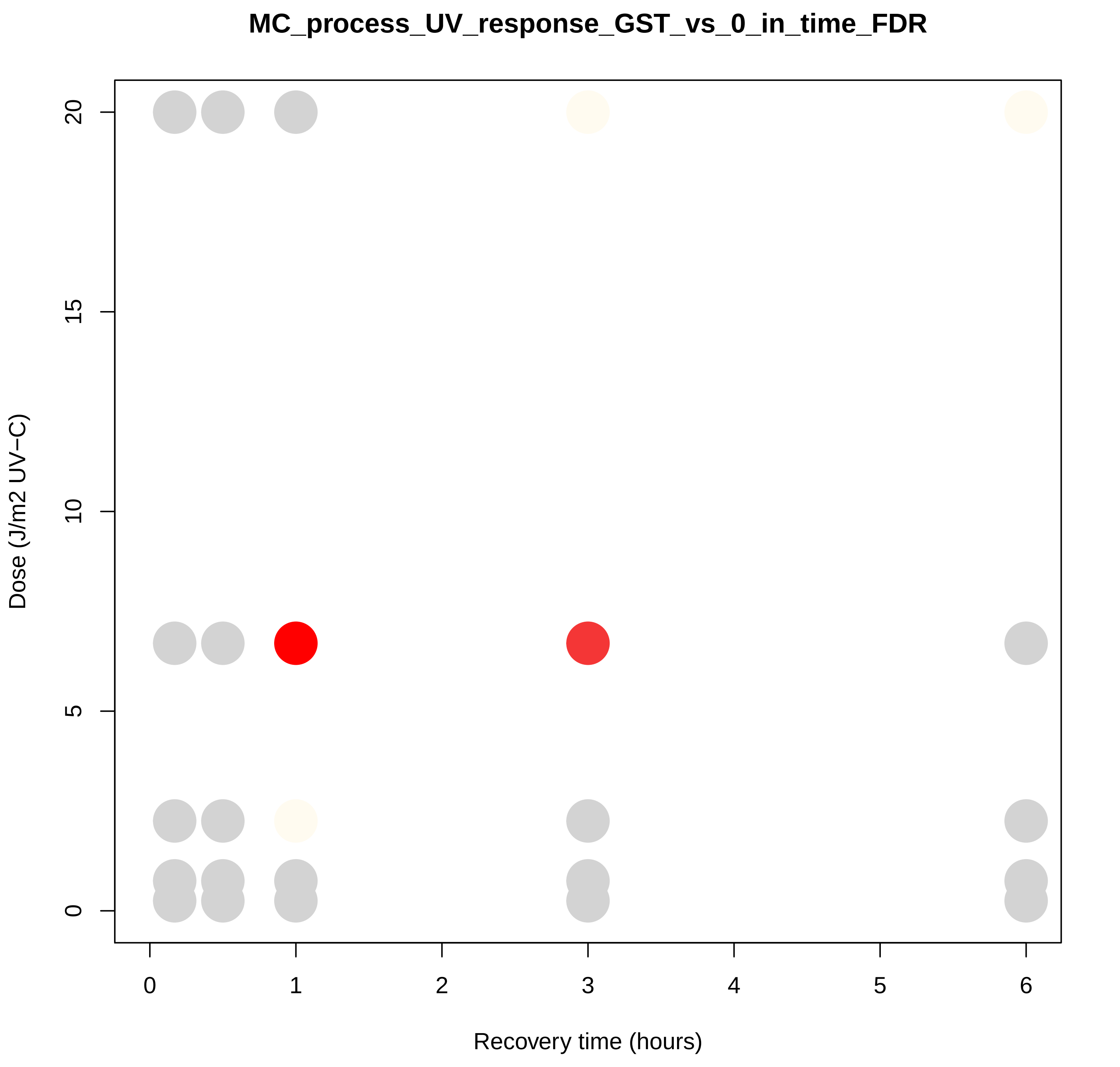

MC_process_UV_response_GST_vs_0_in_time_FDR
Dose (J/m2 UV−C)
Recovery time (hours)
0
5
10
15
20
0
1
2
3
4
5
6

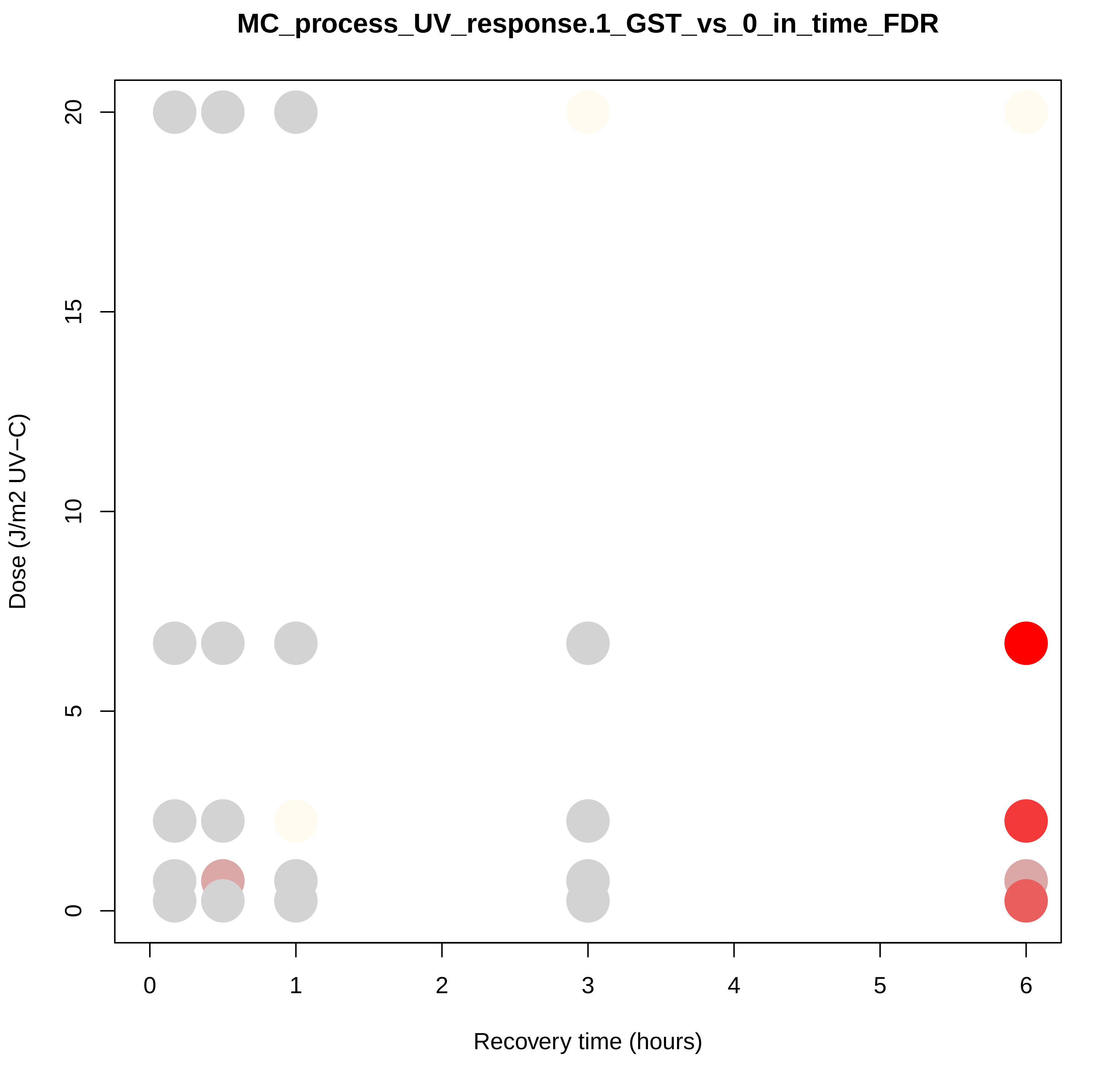
MC_process_UV_response.1_GST_vs_0_in_time_FDR
Dose (J/m2 UV−C)
Recovery time (hours)
0
5
10
15
20
0
1
2
3
4
5
6

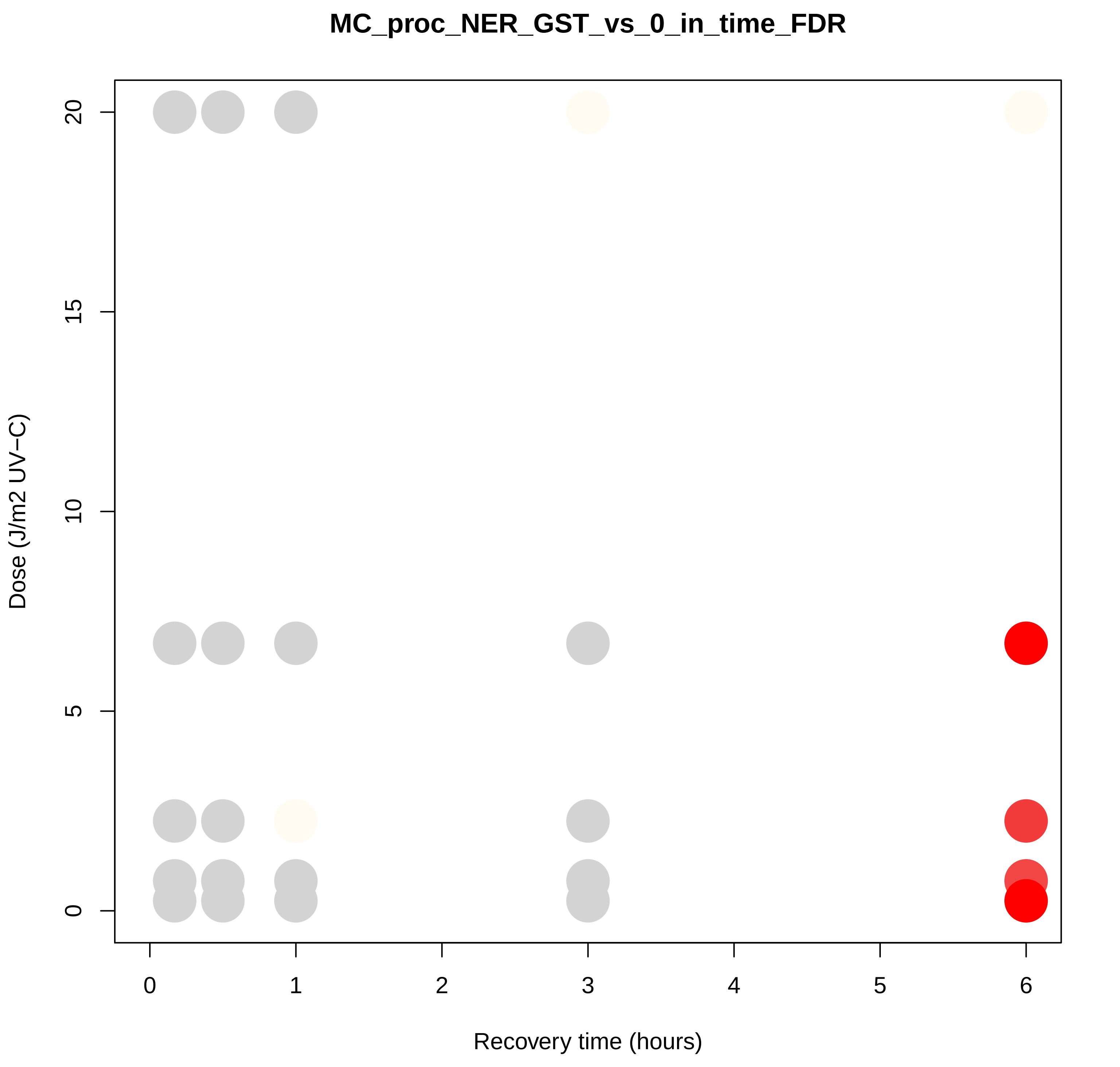

MC_proc_NER_GST_vs_0_in_time_FDR
Dose (J/m2 UV−C)
0
5
10
15
20
Recovery time (hours)
0
1
2
3
4
5
6

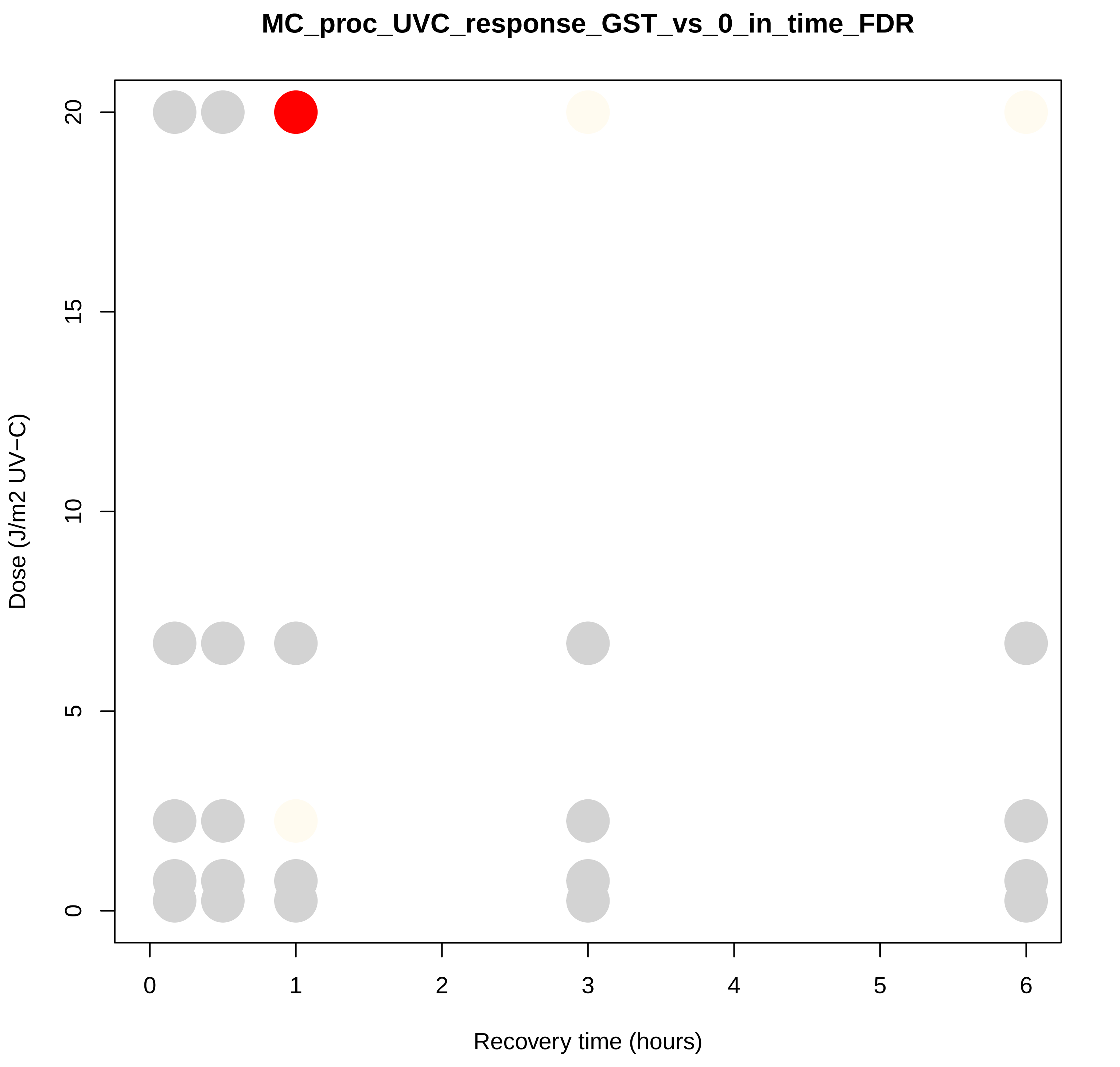
MC_proc_UVC_response_GST_vs_0_in_time_FDR
Dose (J/m2 UV−C)
0
5
10
15
20
0
1
2
3
4
5
6
Recovery time (hours)

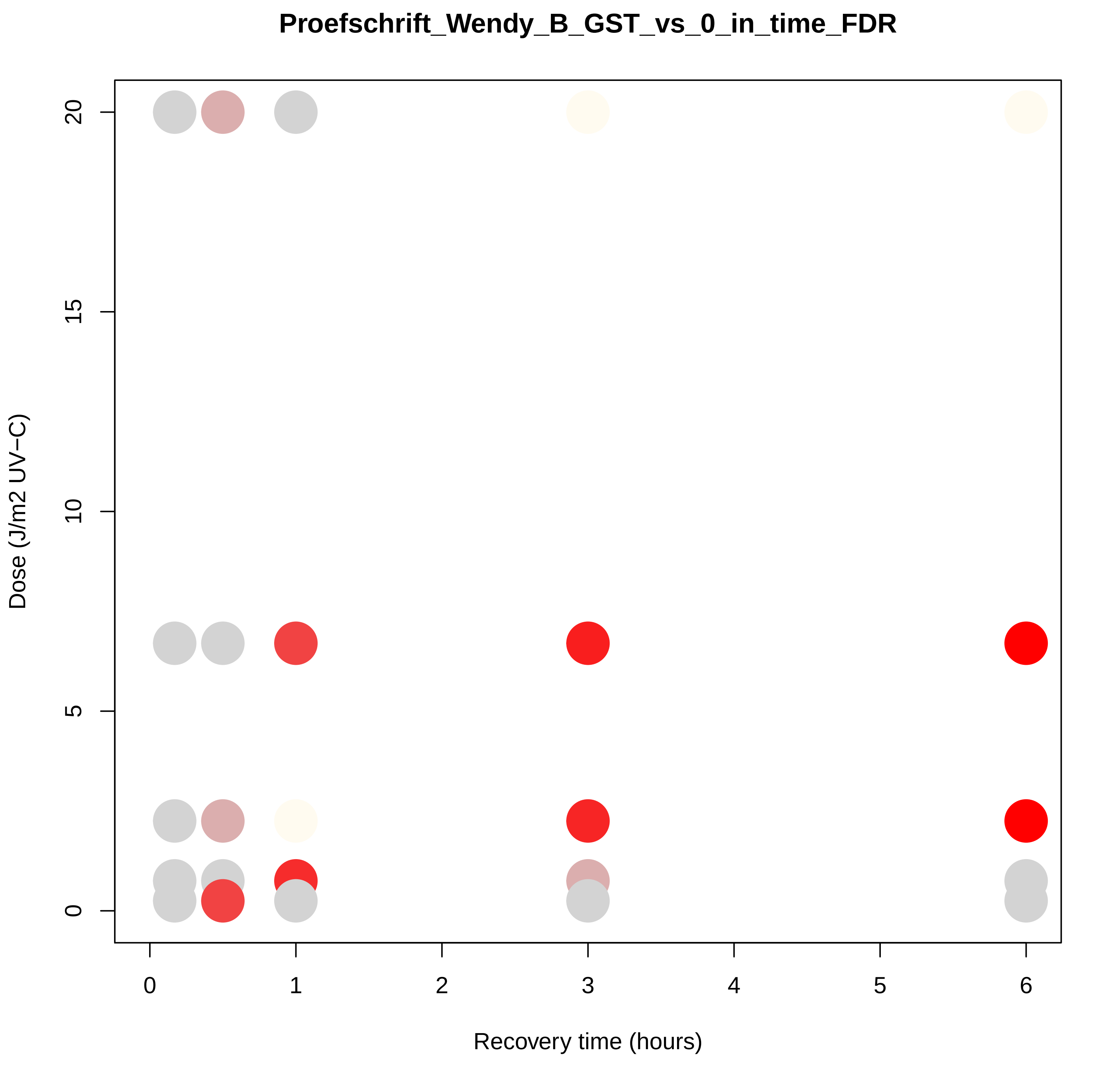
Proefschrift_Wendy_B_GST_vs_0_in_time_FDR
Dose (J/m2 UV−C)
0
5
10
15
20
0
1
2
3
4
5
6
Recovery time (hours)

Riley_S1_p53_regulated_genes_GST_vs_0_in_time_FDR

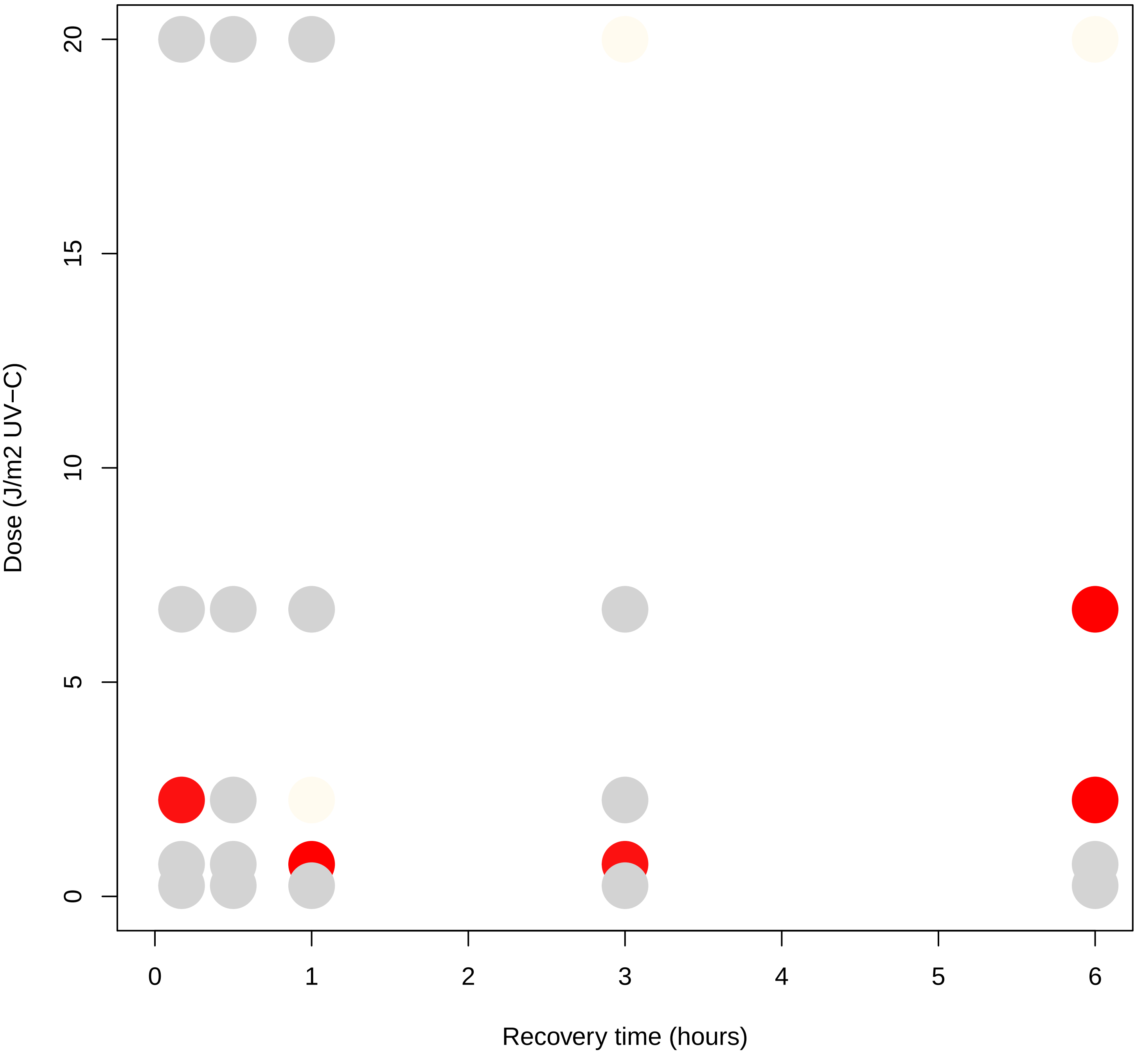

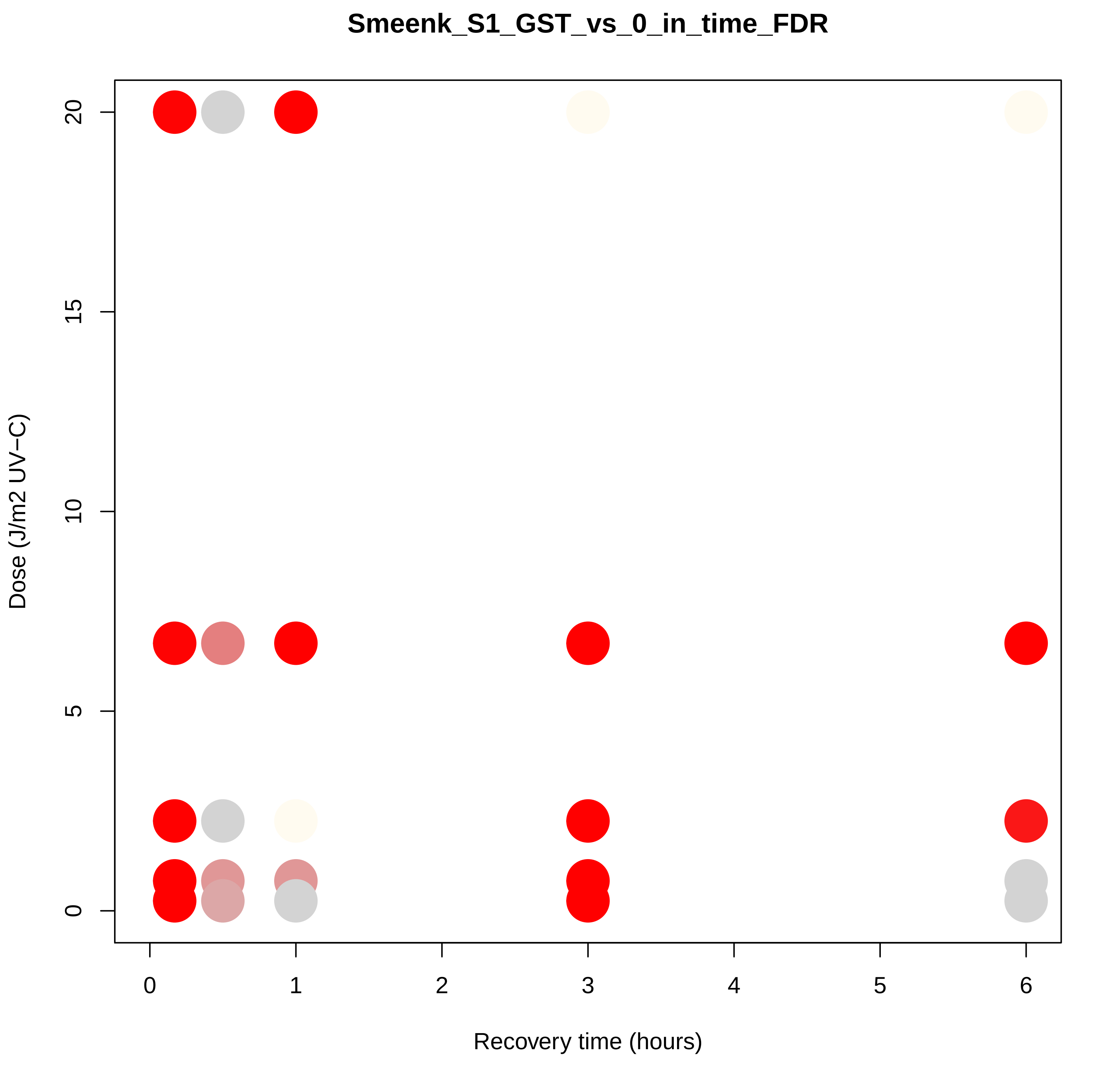

Smeenk_S1_GST_vs_0_in_time_FDR
Dose (J/m2 UV−C)
0
5
10
15
20
0
1
2
3
4
5
6
Recovery time (hours)

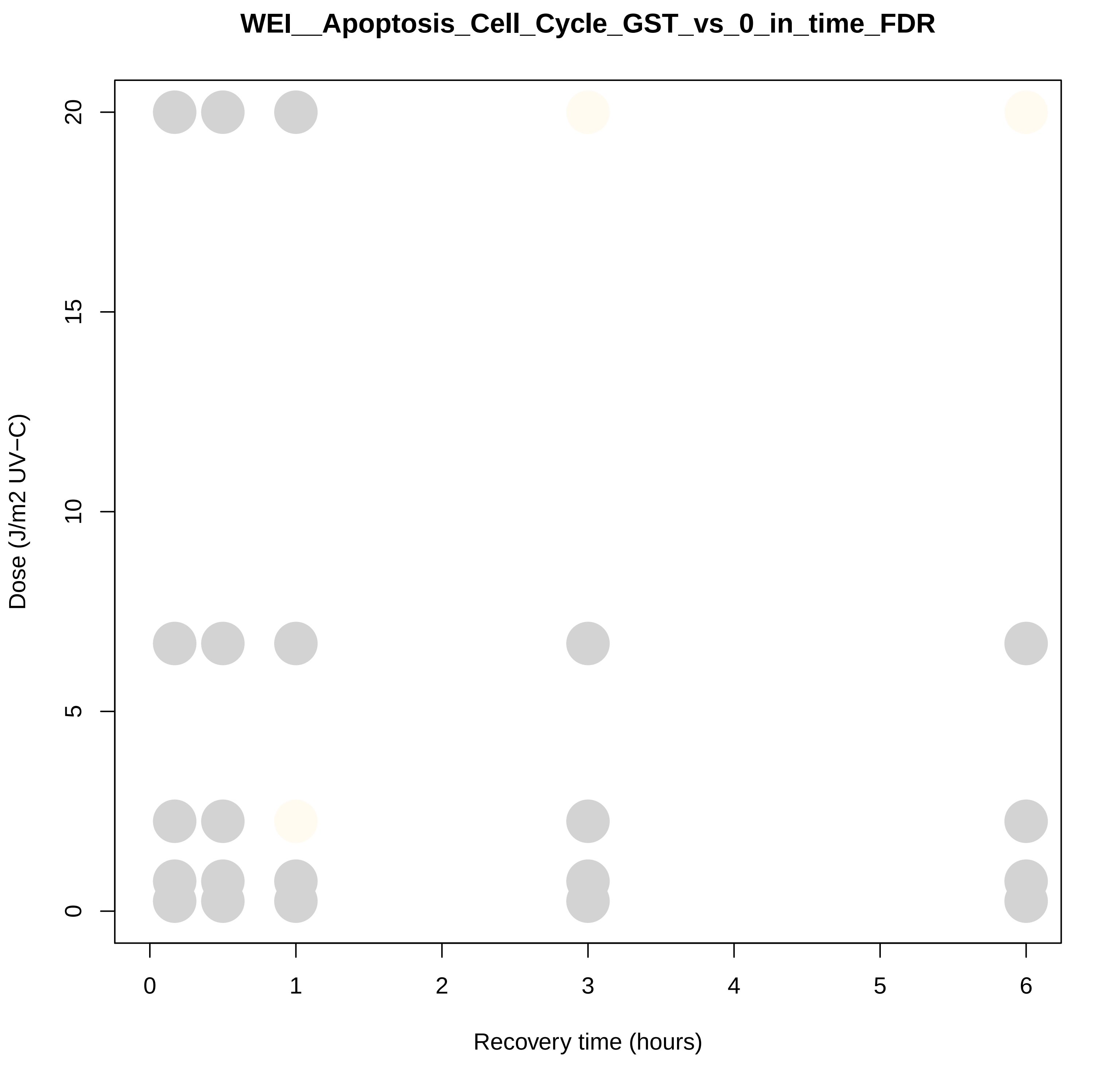
WEI__Apoptosis_Cell_Cycle_GST_vs_0_in_time_FDR
Dose (J/m2 UV−C)
0
5
10
15
20
0
1
2
3
4
5
6
Recovery time (hours)

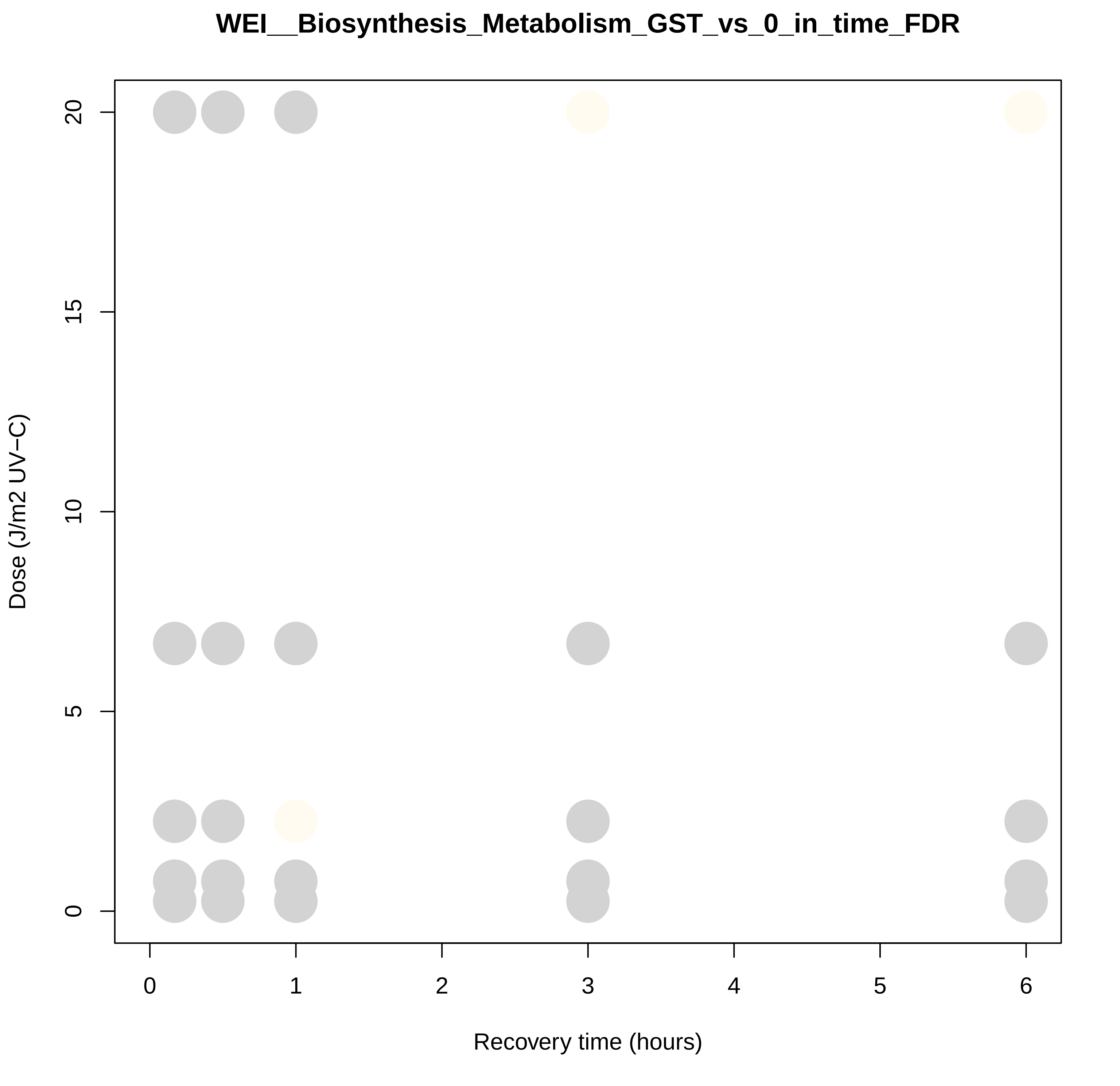
WEI__Biosynthesis_Metabolism_GST_vs_0_in_time_FDR
Dose (J/m2 UV−C)
Recovery time (hours)
0
5
10
15
20
0
1
2
3
4
5
6

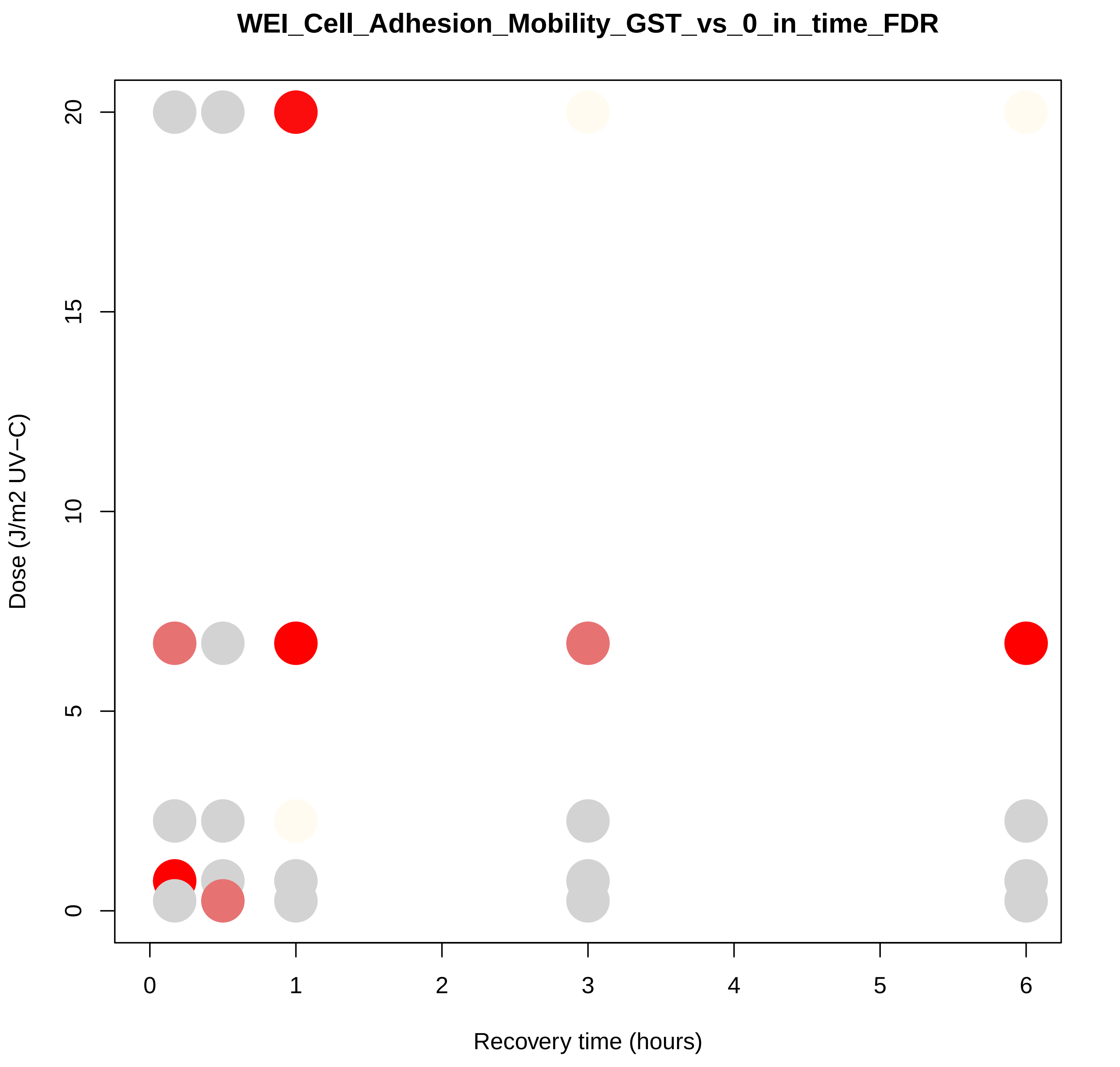
WEI_Cell_Adhesion_Mobility_GST_vs_0_in_time_FDR
Dose (J/m2 UV−C)
0
5
10
15
20
Recovery time (hours)
0
1
2
3
4
5
6

WEI_Cell_Growth_Differentiation_GST_vs_0_in_time_FDR

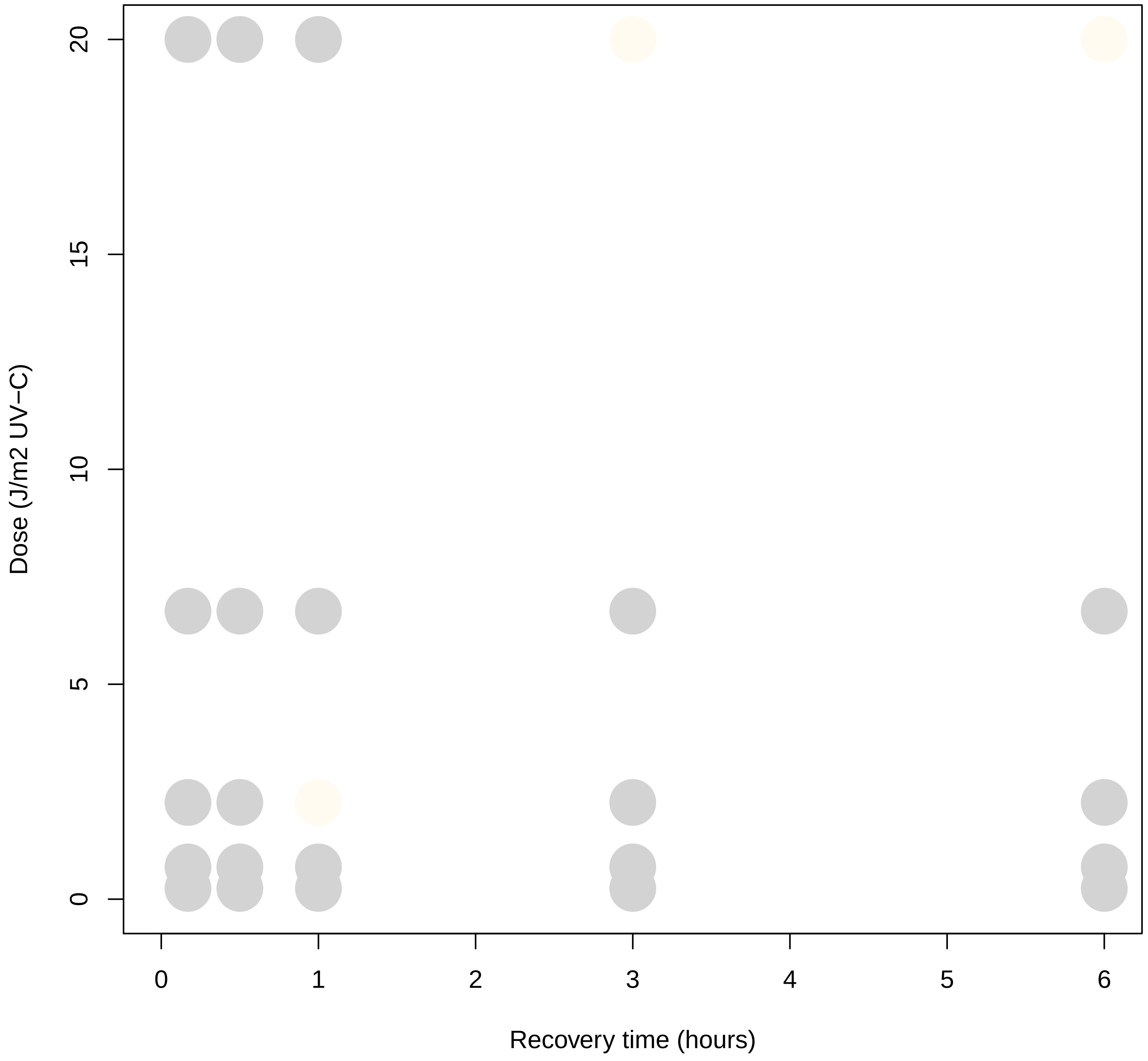

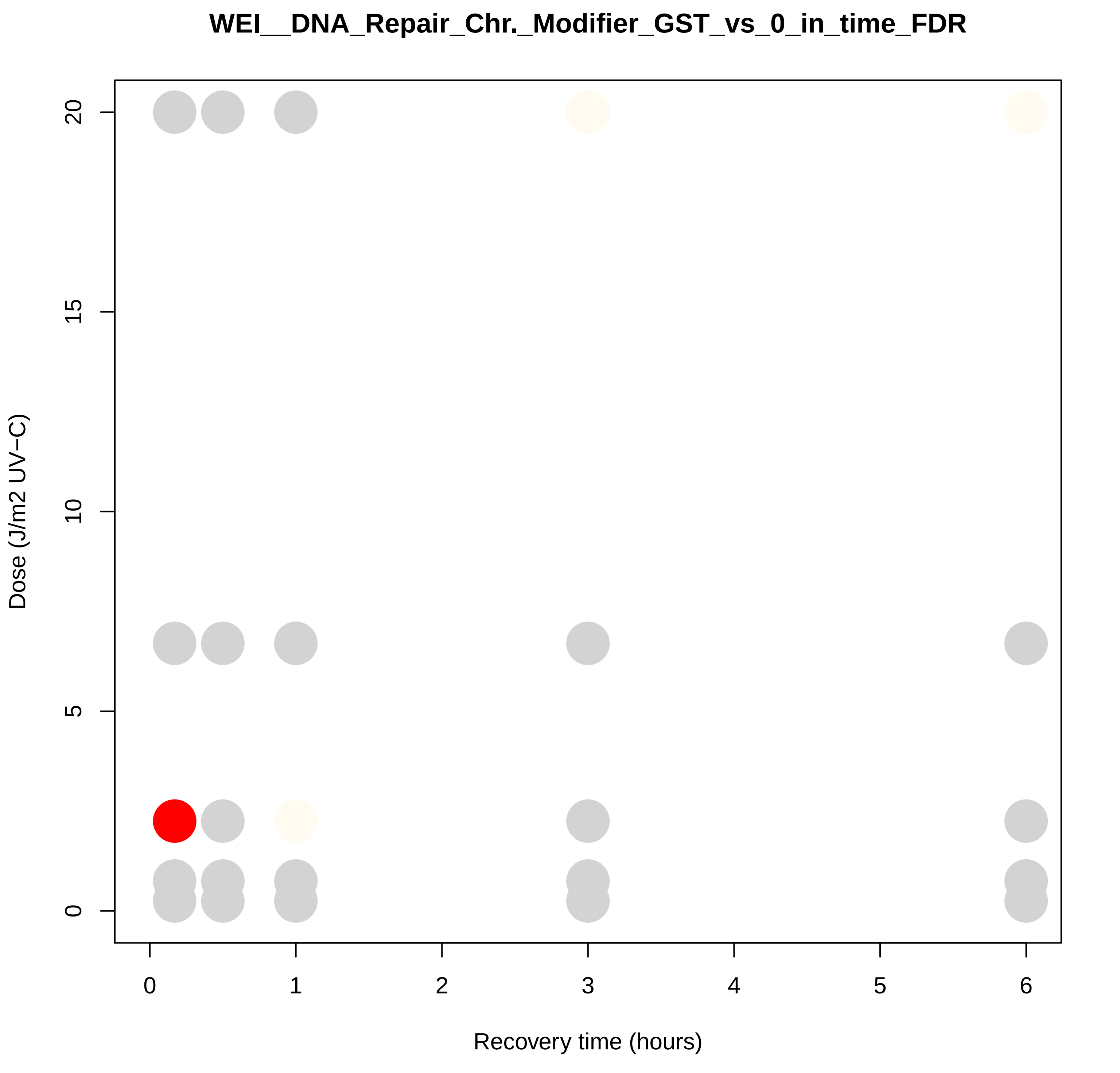
WEI__DNA_Repair_Chr._Modifier_GST_vs_0_in_time_FDR
Dose (J/m2 UV−C)
Recovery time (hours)
0
5
10
15
20
0
1
2
3
4
5
6

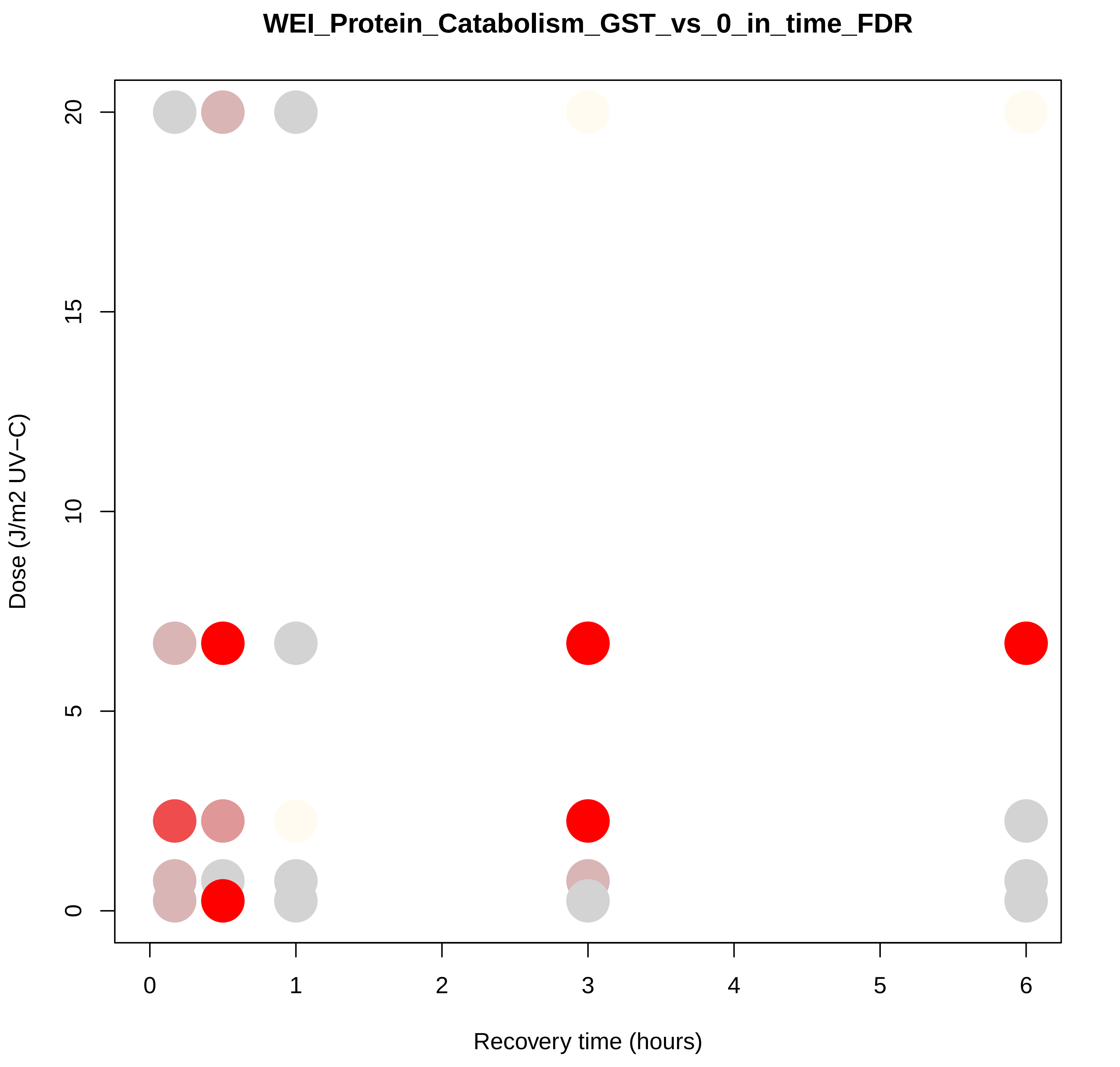
WEI_Protein_Catabolism_GST_vs_0_in_time_FDR
Dose (J/m2 UV−C)
Recovery time (hours)
0
5
10
15
20
0
1
2
3
4
5
6

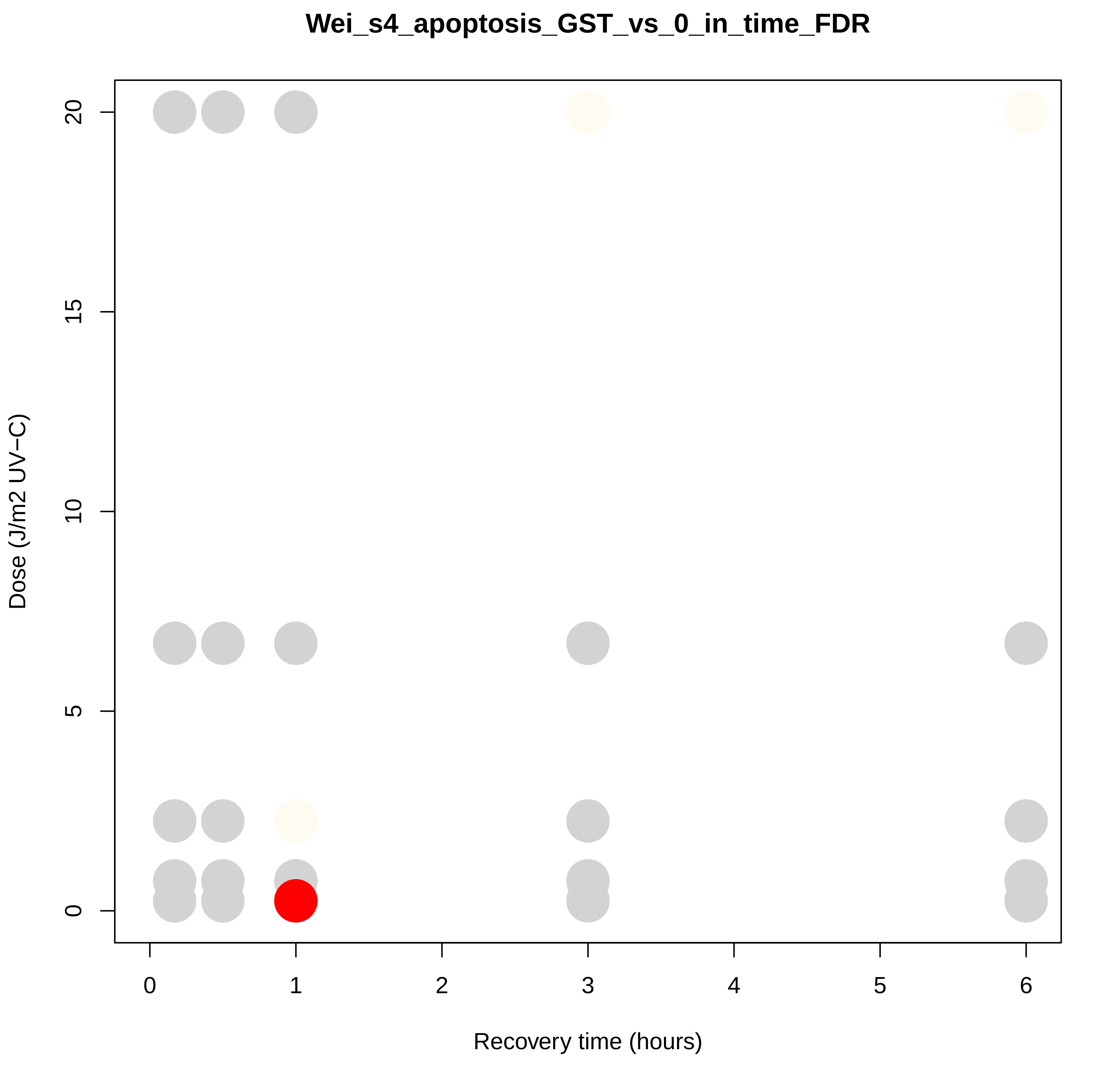
Wei_s4_apoptosis_GST_vs_0_in_time_FDR
Dose (J/m2 UV−C)
0
5
10
15
20
Recovery time (hours)
0
1
2
3
4
5
6

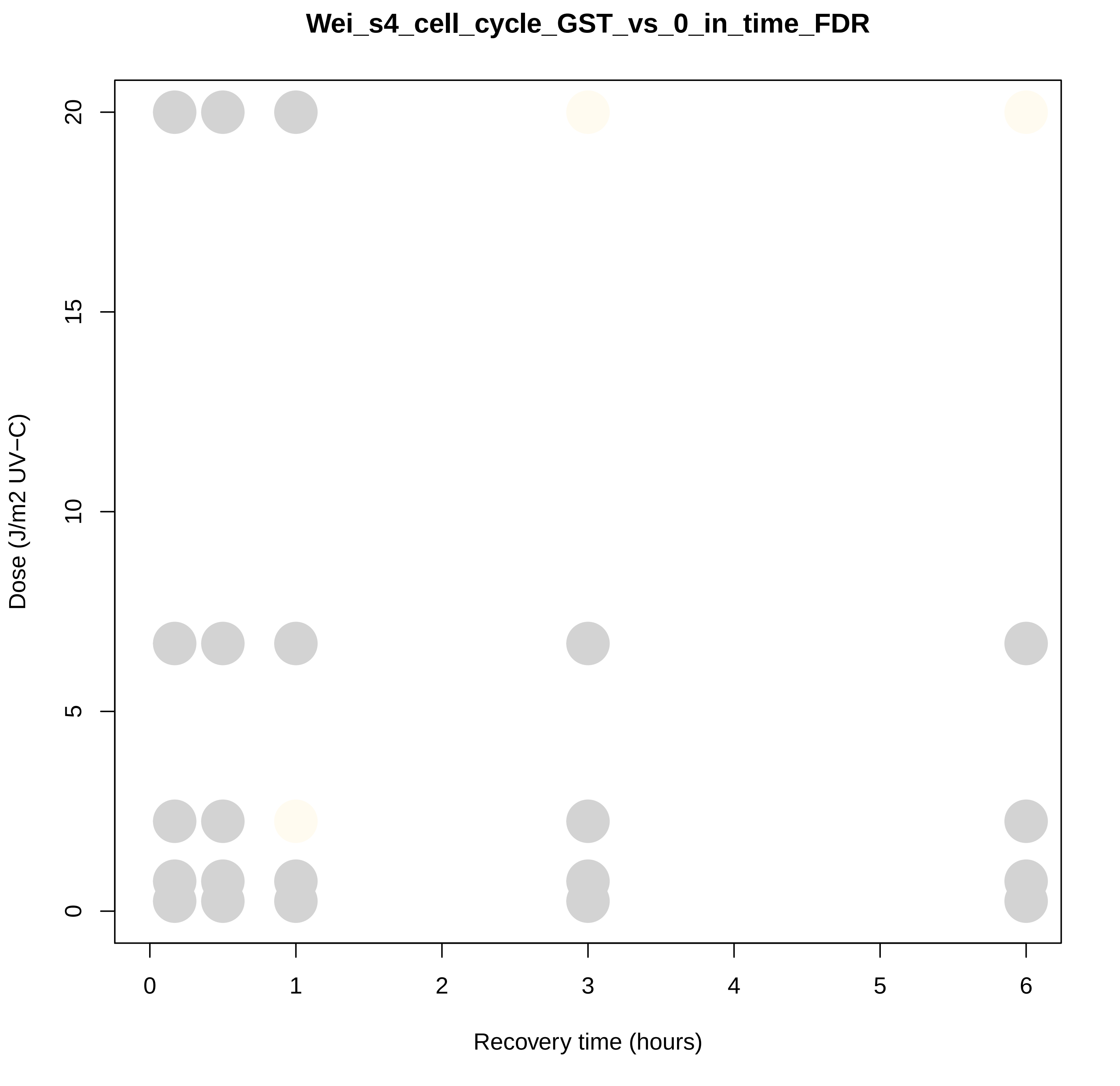

Wei_s4_cell_cycle_GST_vs_0_in_time_FDR
Dose (J/m2 UV−C)
0
5
10
15
20
Recovery time (hours)
0
1
2
3
4
5
6

Wei_s4_Cell_growth_GST_vs_0_in_time_FDR

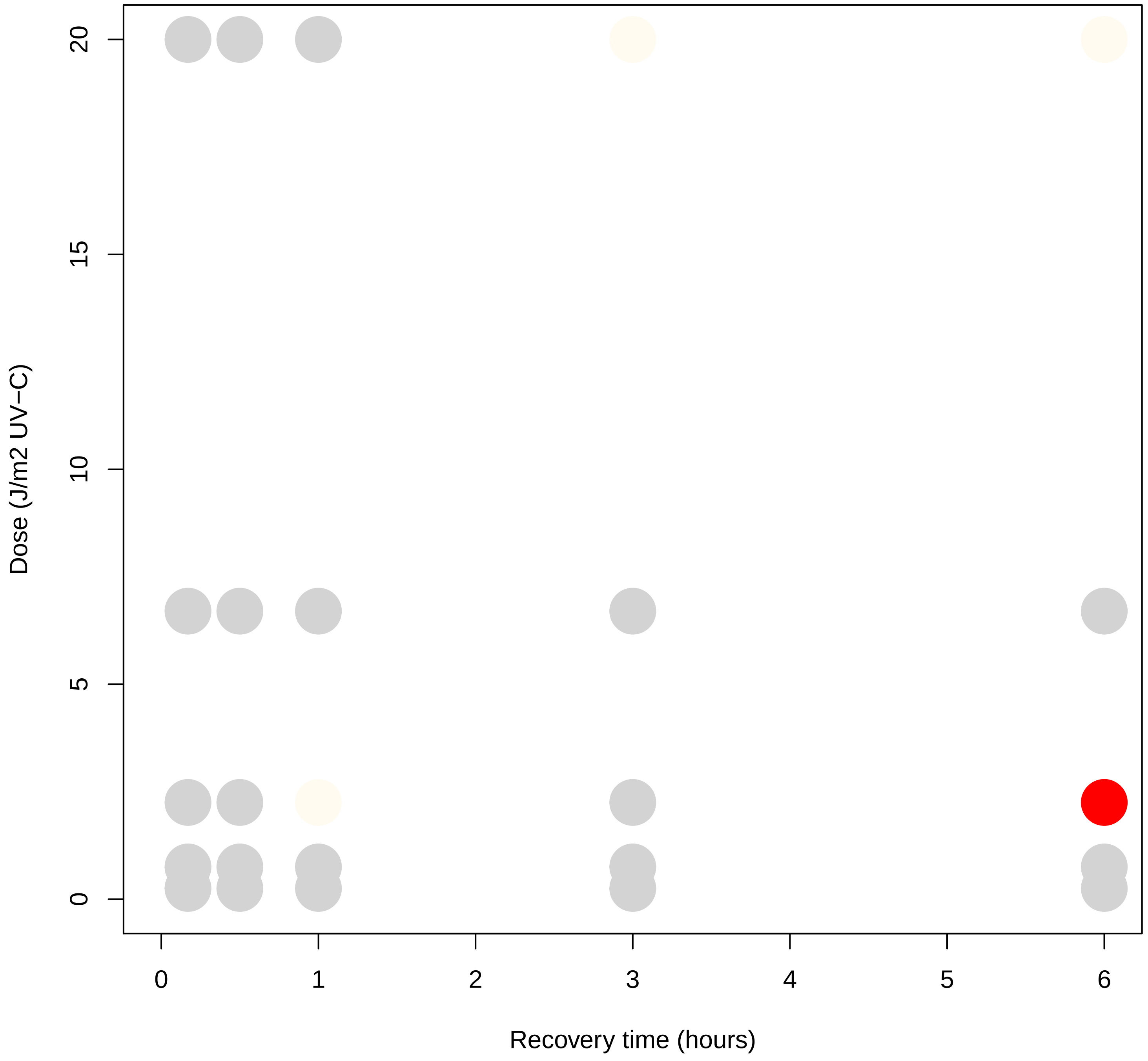

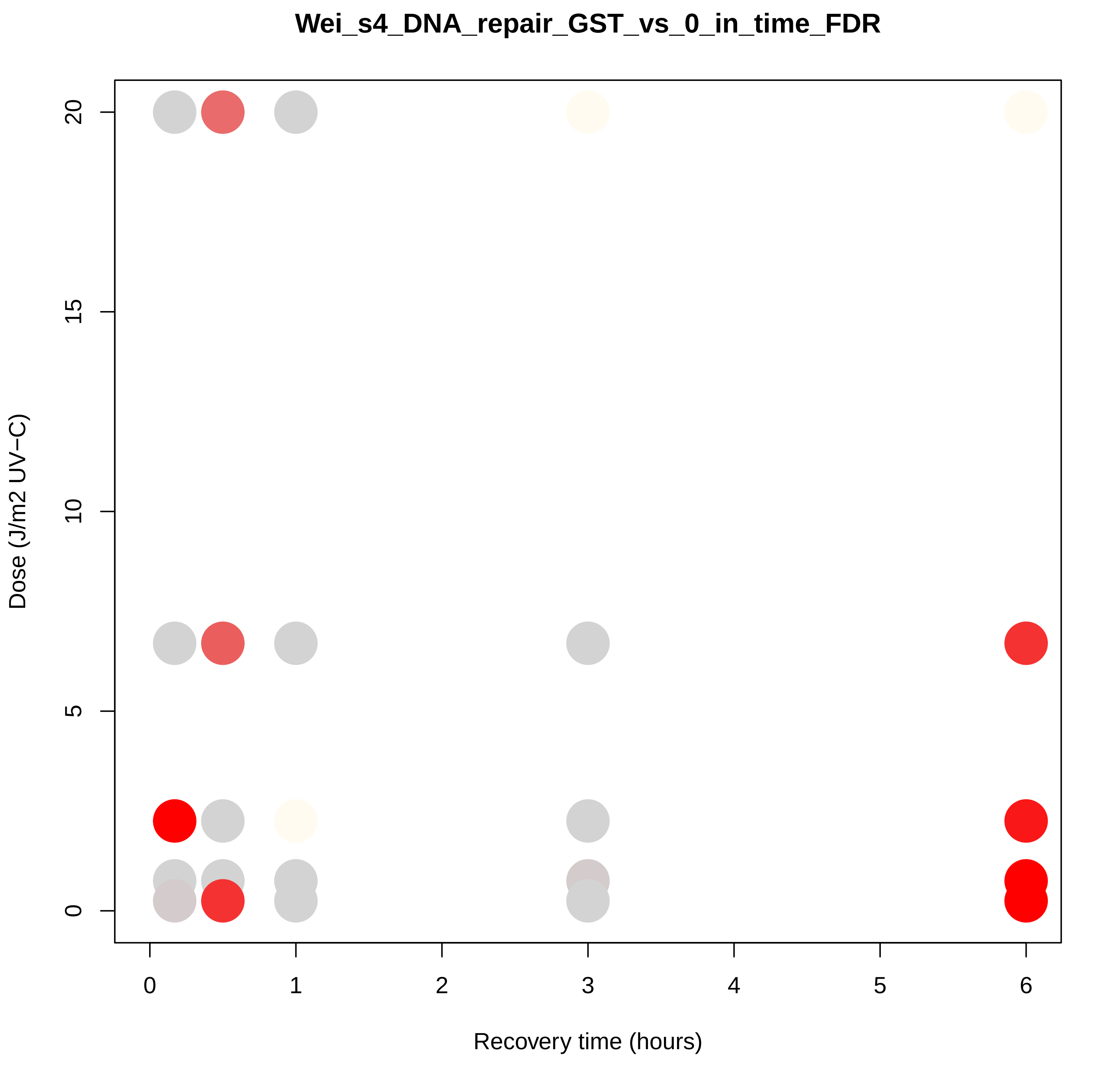
Wei_s4_DNA_repair_GST_vs_0_in_time_FDR
Dose (J/m2 UV−C)
0
5
10
15
20
0
1
2
3
4
5
6
Recovery time (hours)

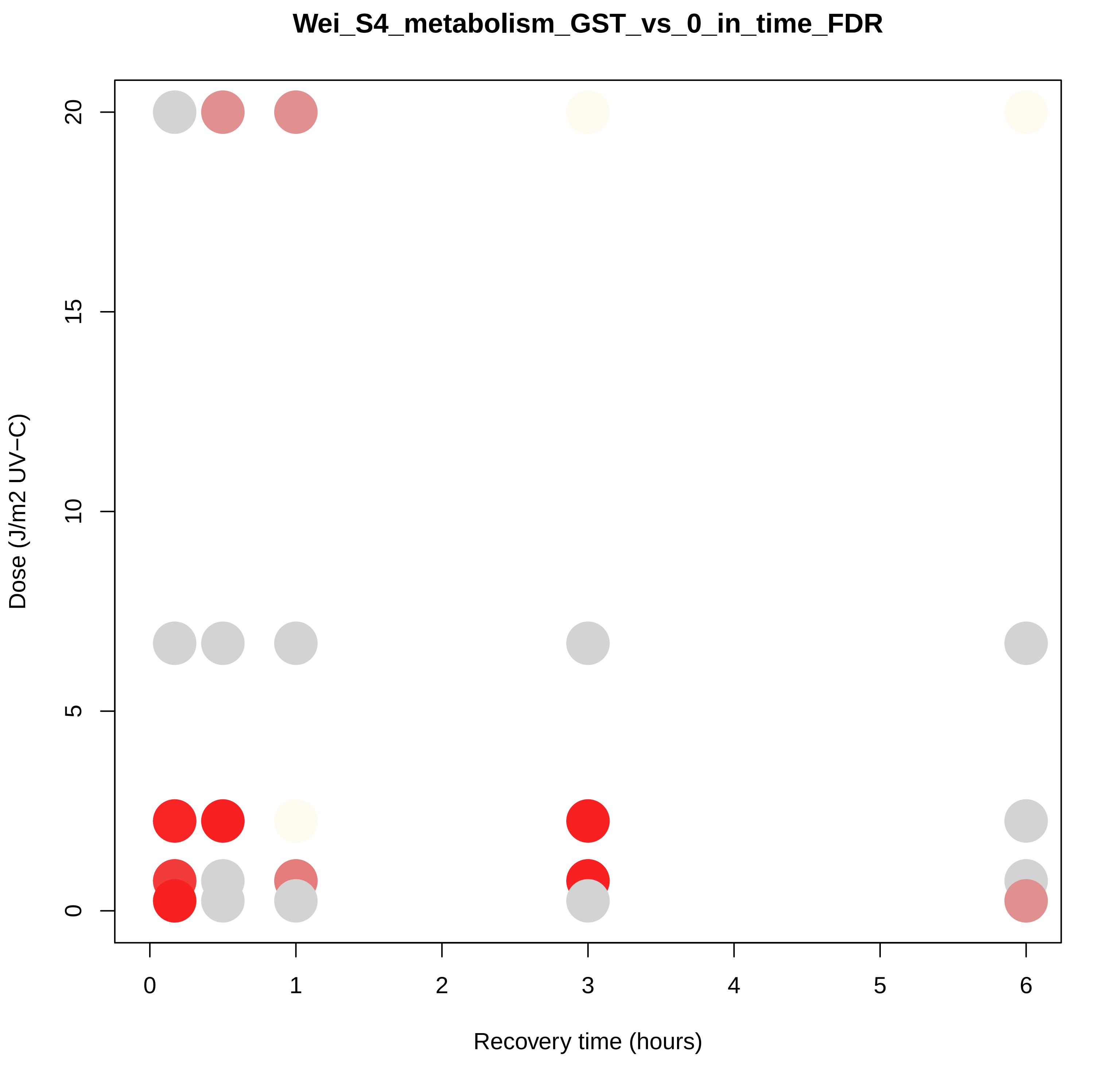

Wei_S4_metabolism_GST_vs_0_in_time_FDR
Dose (J/m2 UV−C)
0
5
10
15
20
0
1
2
3
4
5
6
Recovery time (hours)

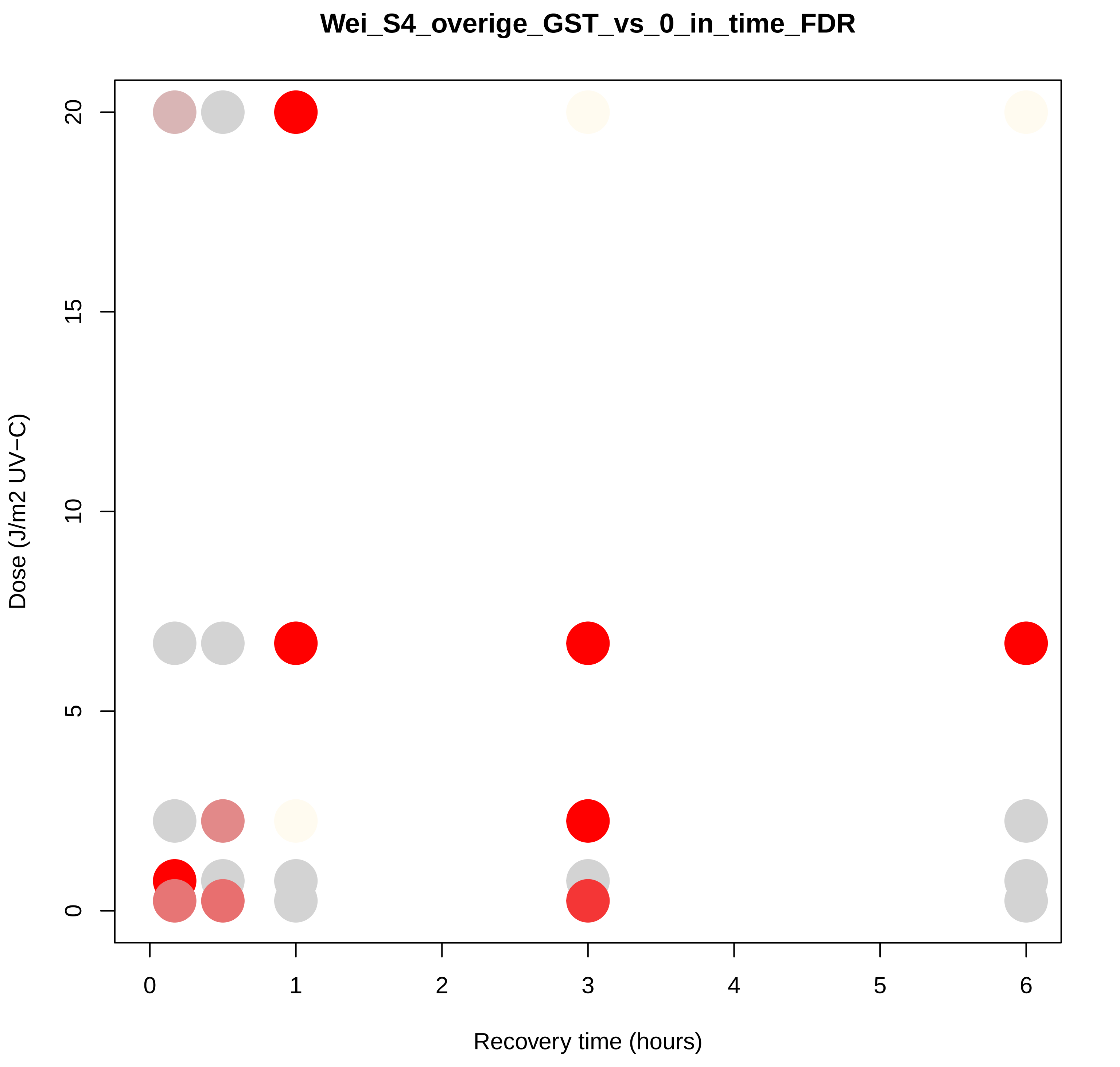
Wei_S4_overige_GST_vs_0_in_time_FDR
Dose (J/m2 UV−C)
0
5
10
15
20
Recovery time (hours)
0
1
2
3
4
5
6

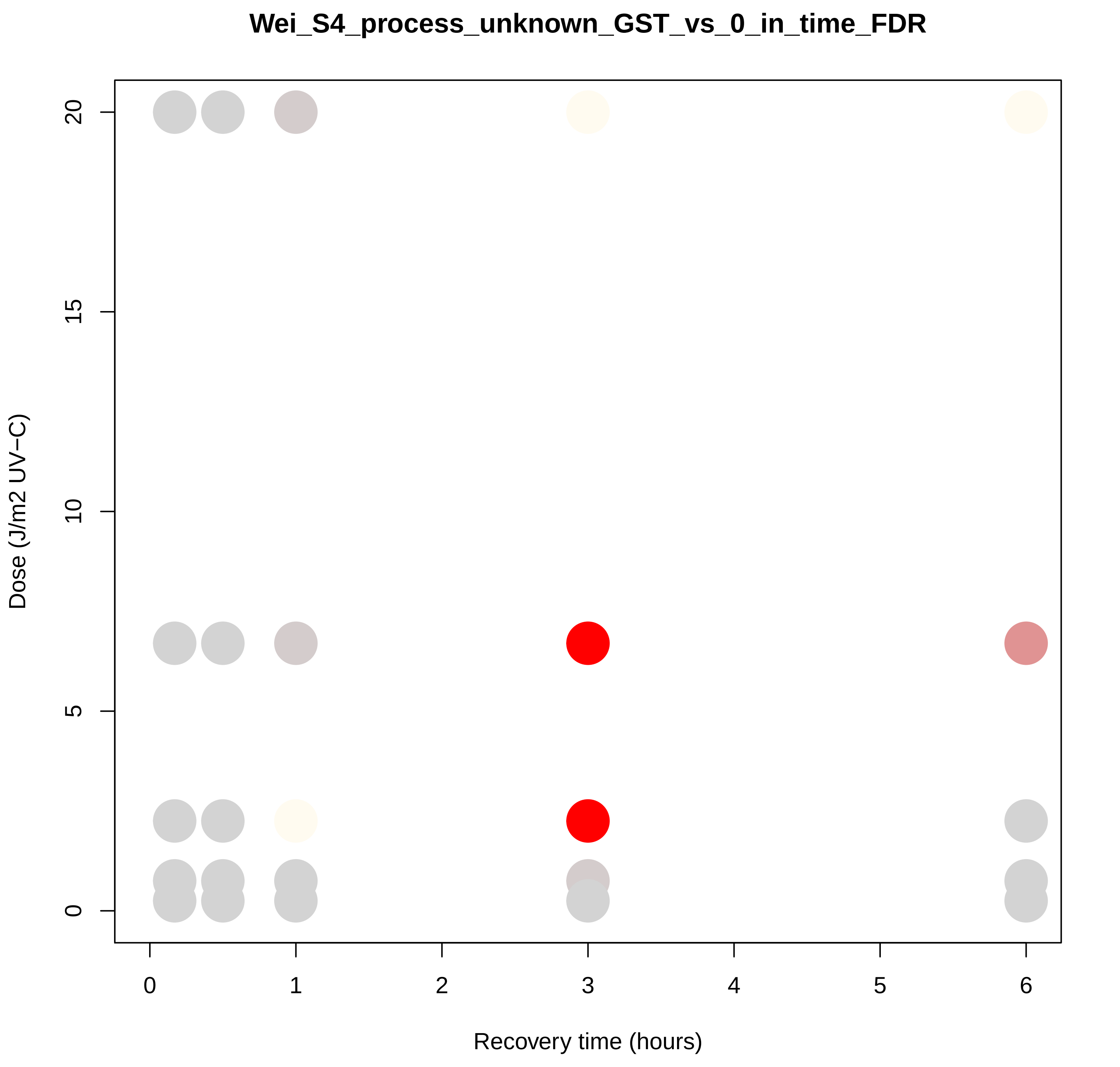
Wei_S4_process_unknown_GST_vs_0_in_time_FDR
Dose (J/m2 UV−C)
0
5
10
15
20
Recovery time (hours)
0
1
2
3
4
5
6

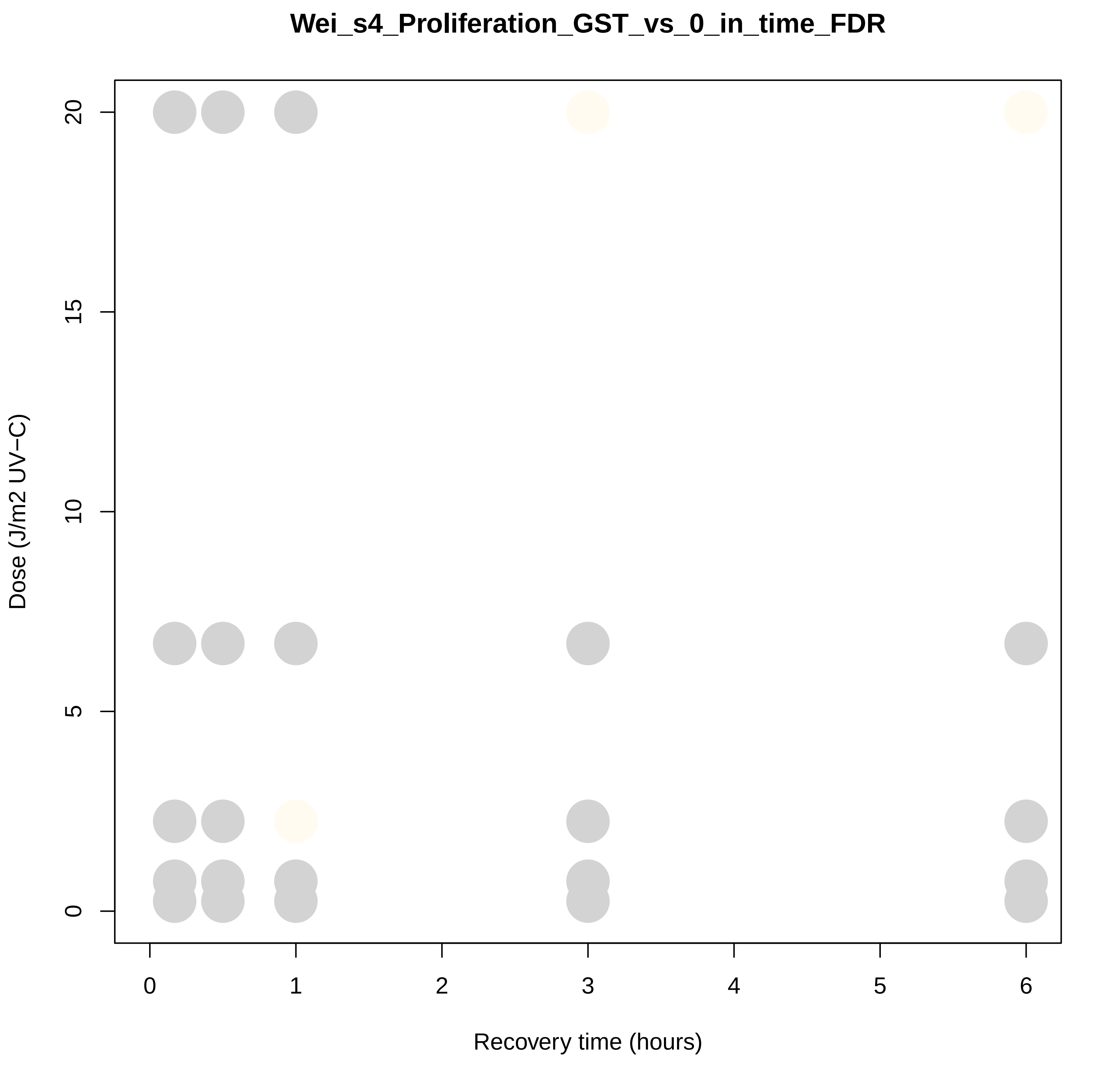
Wei_s4_Proliferation_GST_vs_0_in_time_FDR
Dose (J/m2 UV−C)
0
5
10
15
20
Recovery time (hours)
0
1
2
3
4
5
6

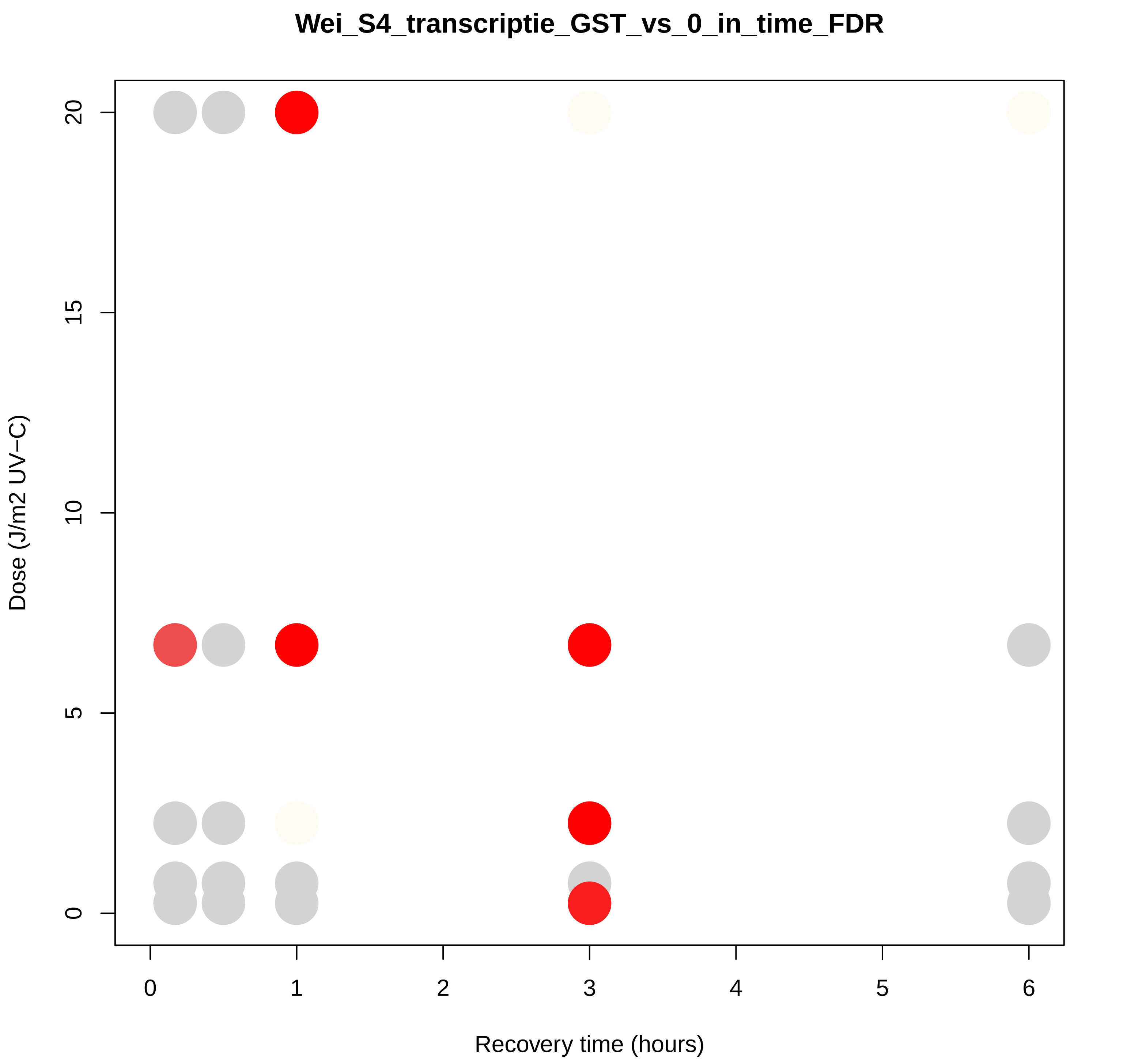
Wei_S4_transcriptie_GST_vs_0_in_time_FDR
Dose (J/m2 UV−C)
0
5
10
15
20
0
1
2
3
4
5
6
Recovery time (hours)

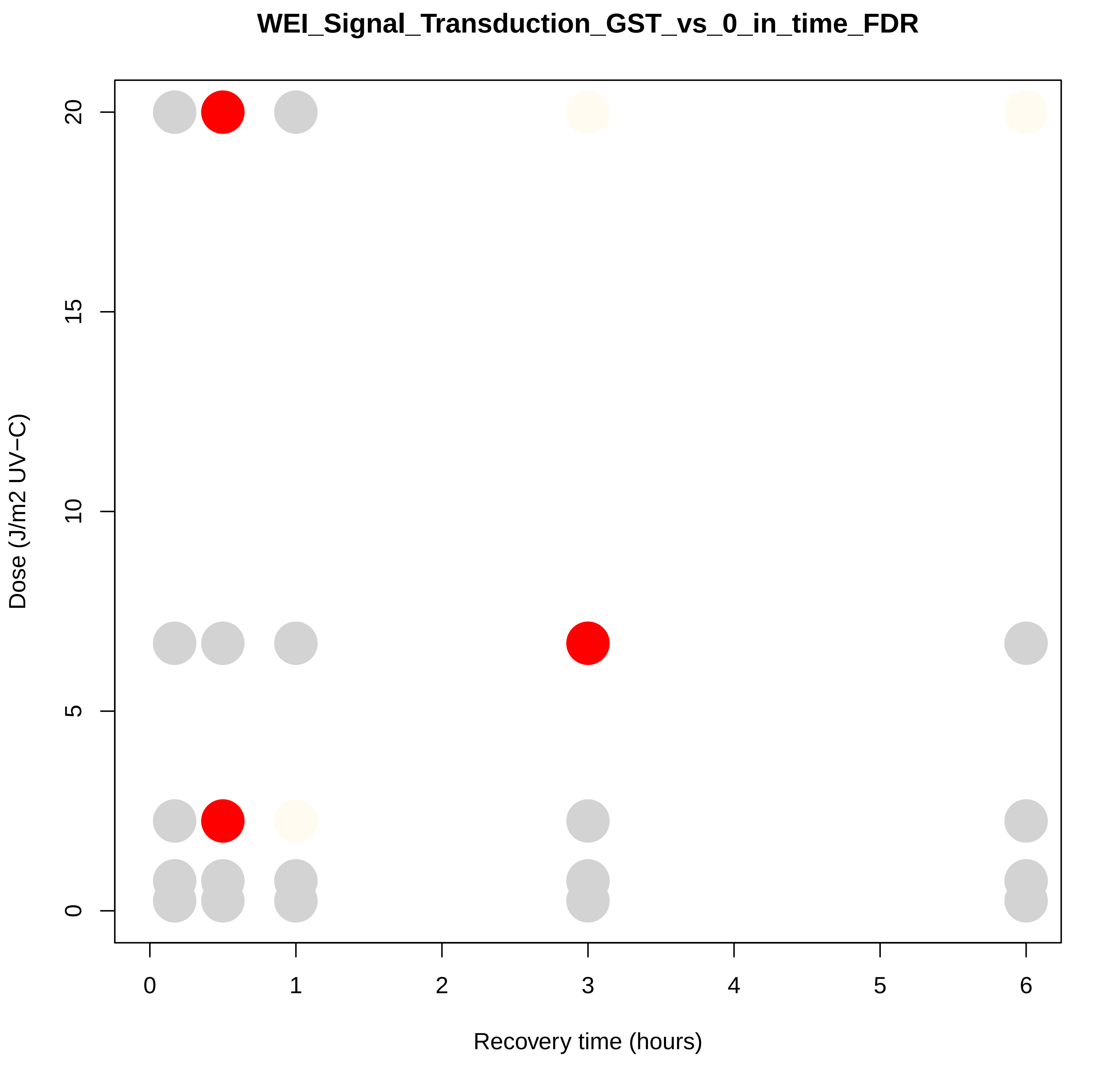
WEI_Signal_Transduction_GST_vs_0_in_time_FDR
Dose (J/m2 UV−C)
0
5
10
15
20
Recovery time (hours)
0
1
2
3
4
5
6

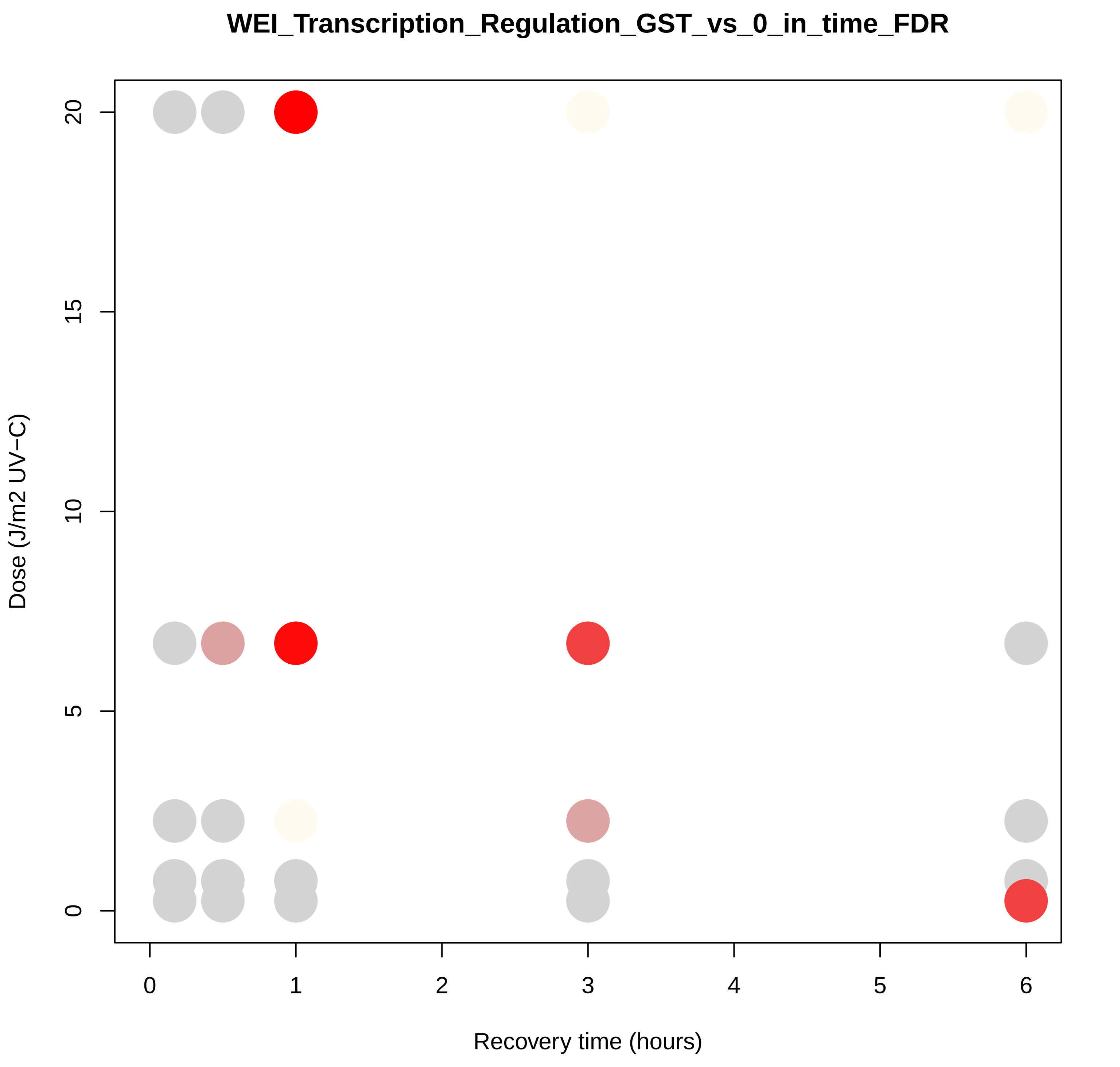
WEI_Transcription_Regulation_GST_vs_0_in_time_FDR
Dose (J/m2 UV−C)
Recovery time (hours)
0
5
10
15
20
0
1
2
3
4
5
6

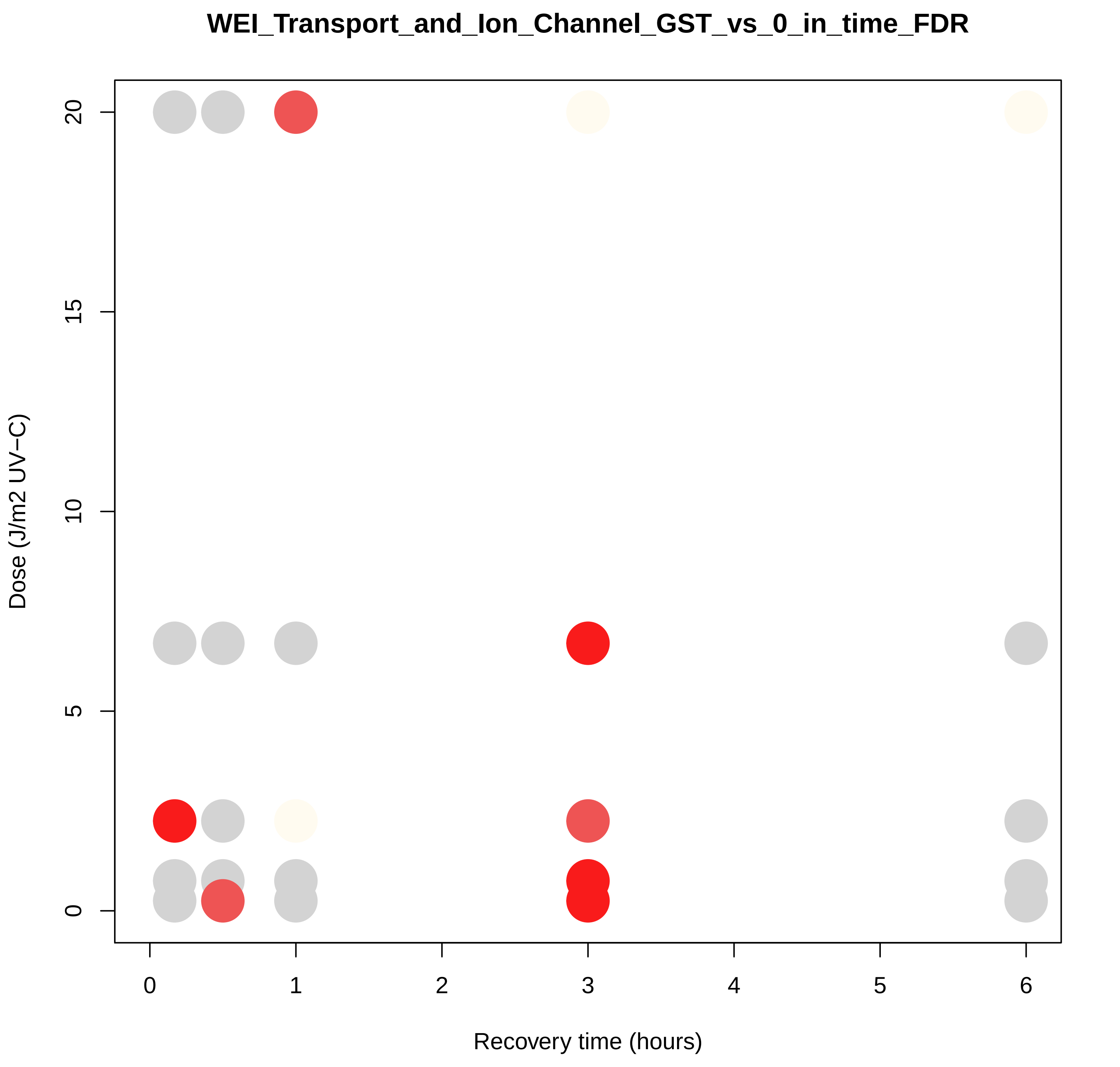

WEI_Transport_and_Ion_Channel_GST_vs_0_in_time_FDR
Dose (J/m2 UV−C)
Recovery time (hours)
0
5
10
15
20
0
1
2
3
4
5
6

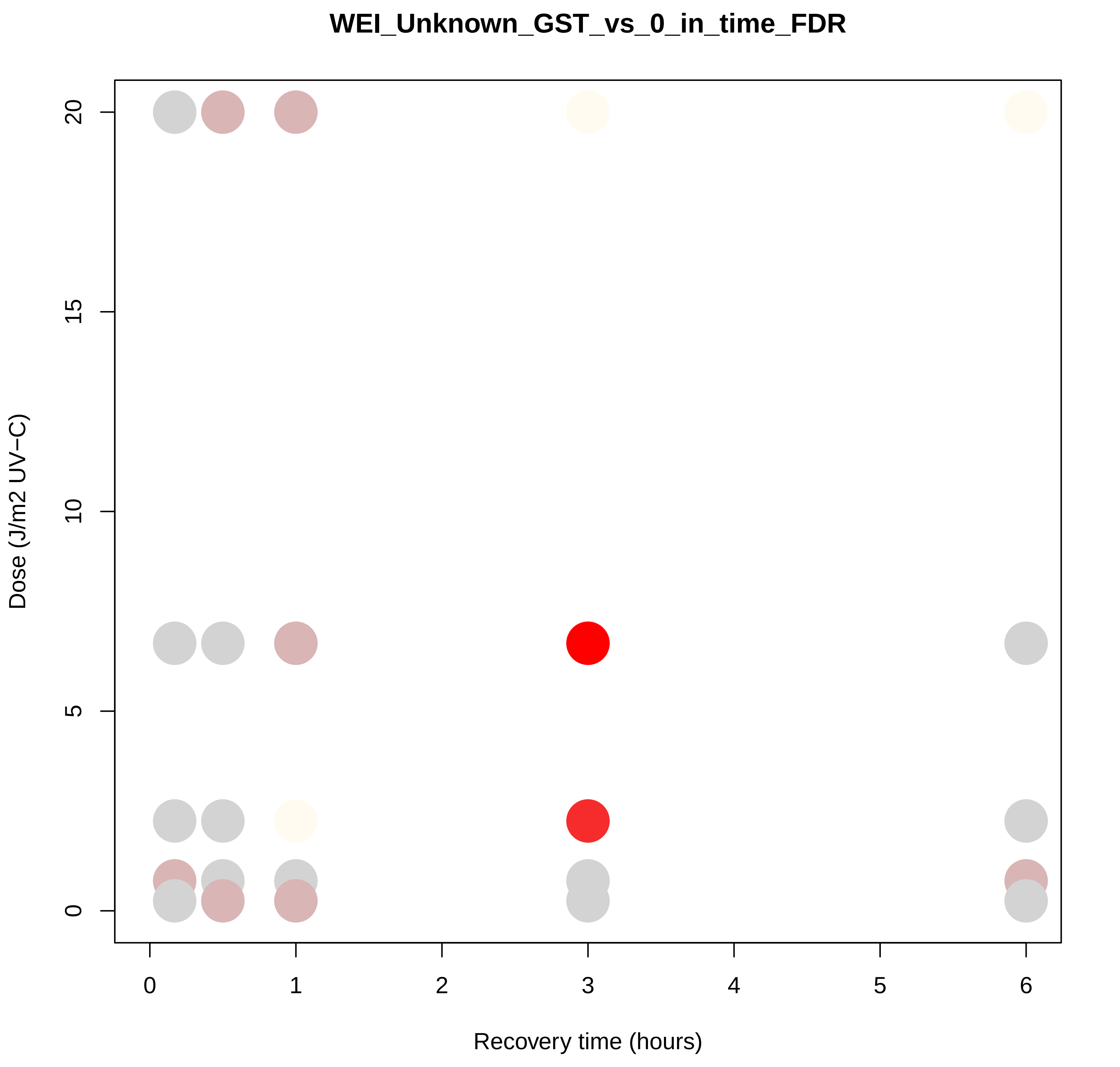

WEI_Unknown_GST_vs_0_in_time_FDR
Dose (J/m2 UV−C)
Recovery time (hours)
0
5
10
15
20
0
1
2
3
4
5
6

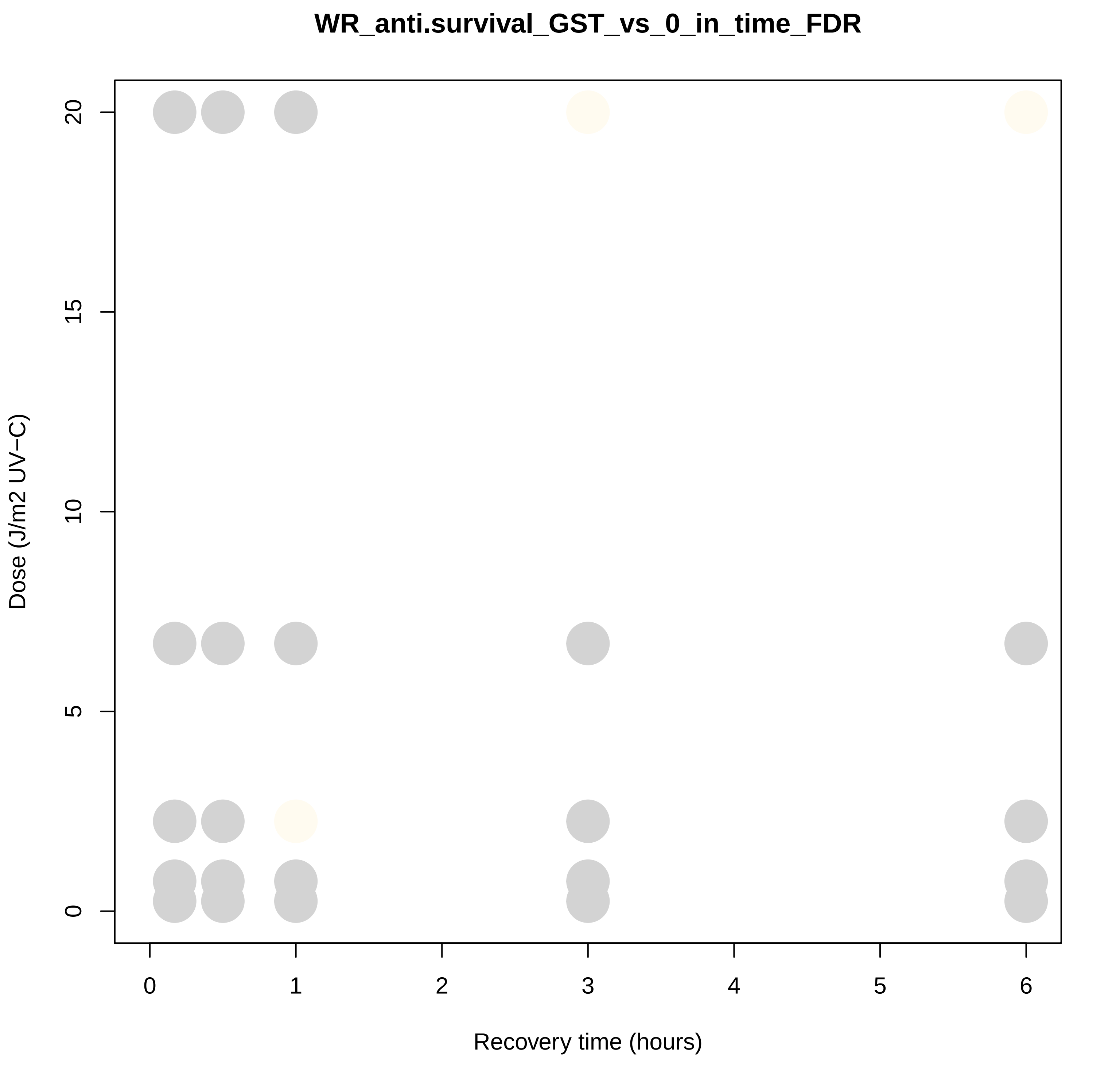

WR_anti.survival_GST_vs_0_in_time_FDR
Dose (J/m2 UV−C)
0
5
10
15
20
0
1
2
3
4
5
6
Recovery time (hours)

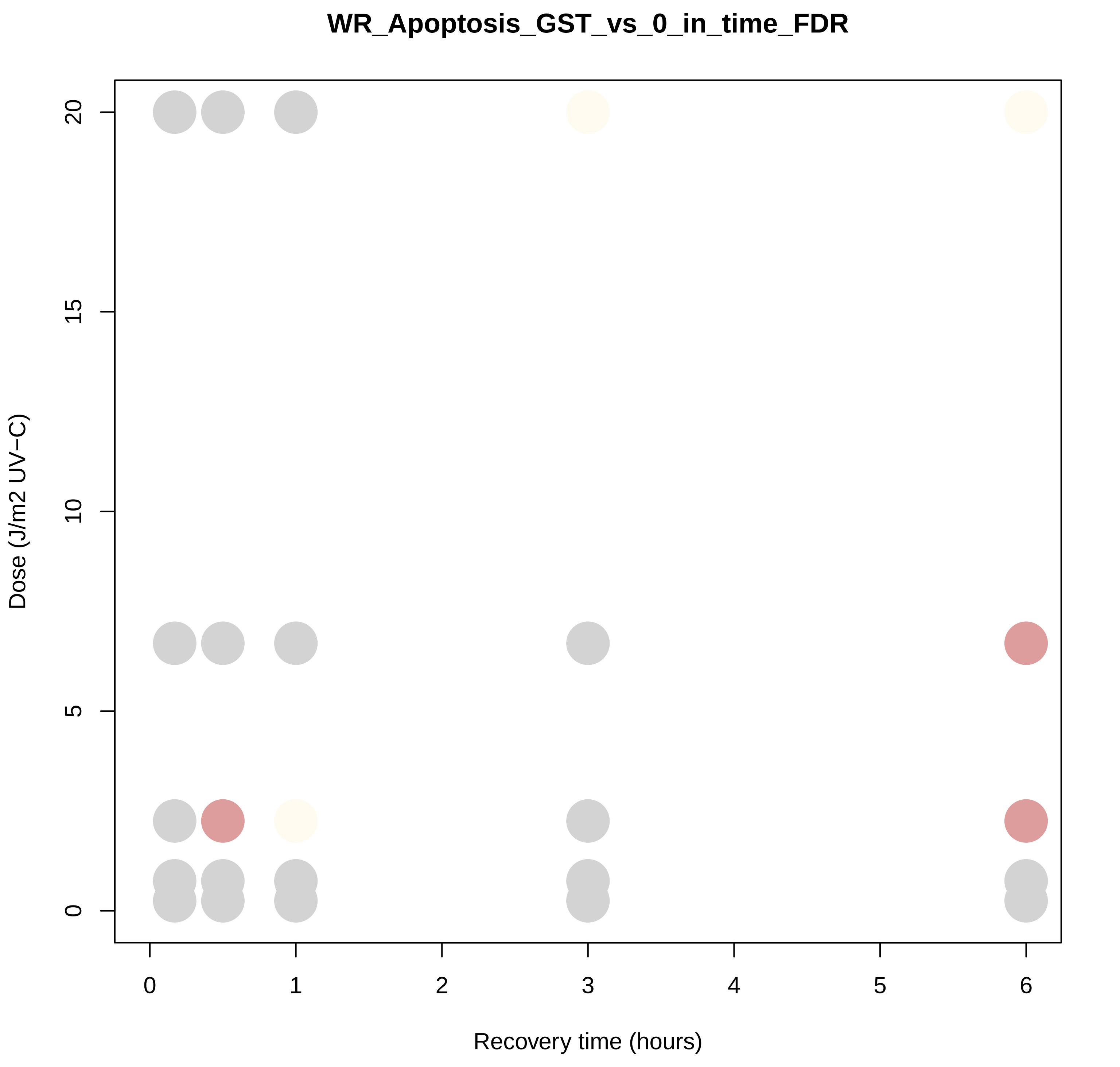

WR_Apoptosis_GST_vs_0_in_time_FDR
Dose (J/m2 UV−C)
0
5
10
15
20
0
1
2
3
4
5
6
Recovery time (hours)

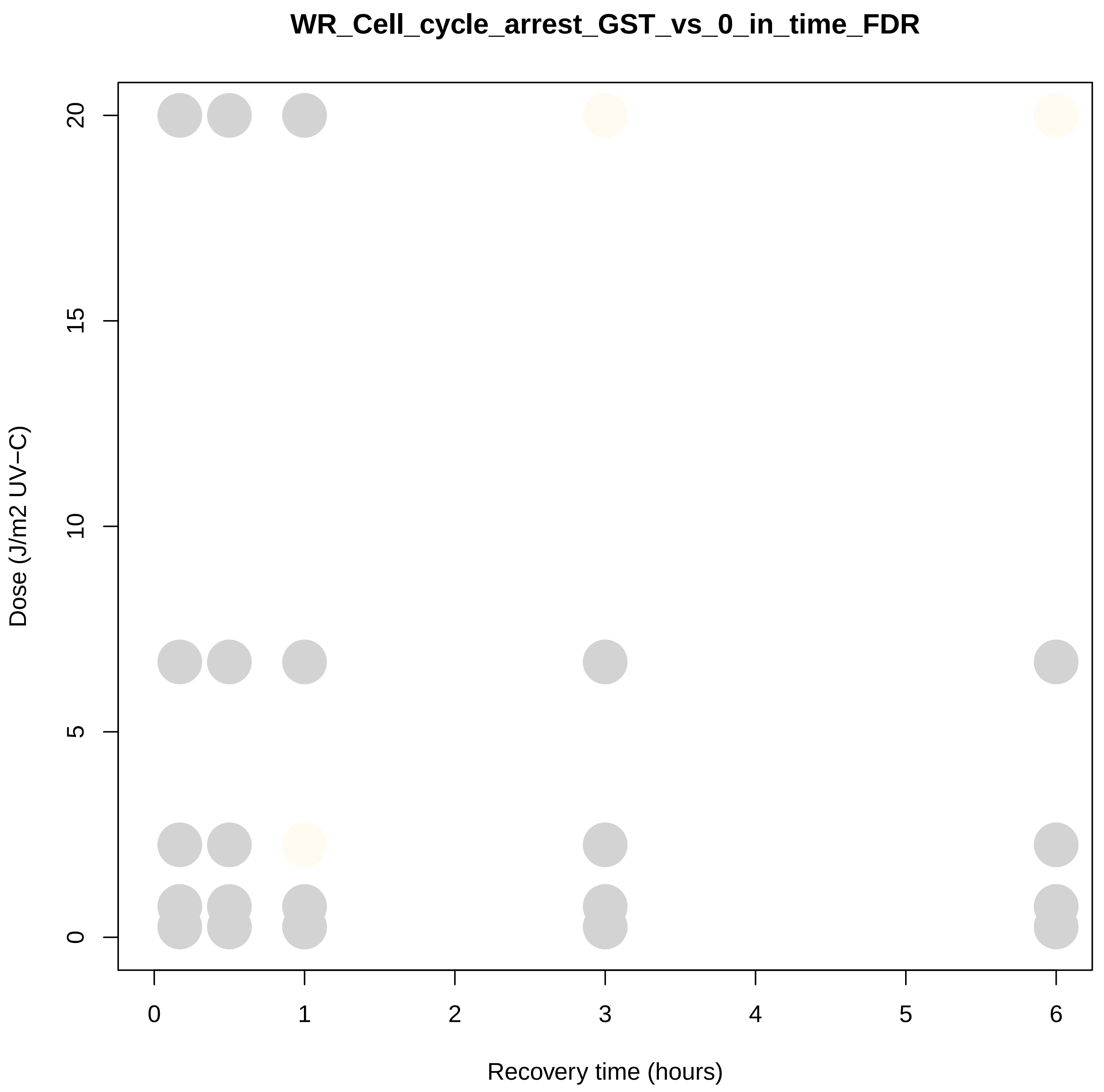
WR_Cell_cycle_arrest_GST_vs_0_in_time_FDR
Dose (J/m2 UV−C)
0
5
10
15
20
0
1
2
3
4
5
6
Recovery time (hours)

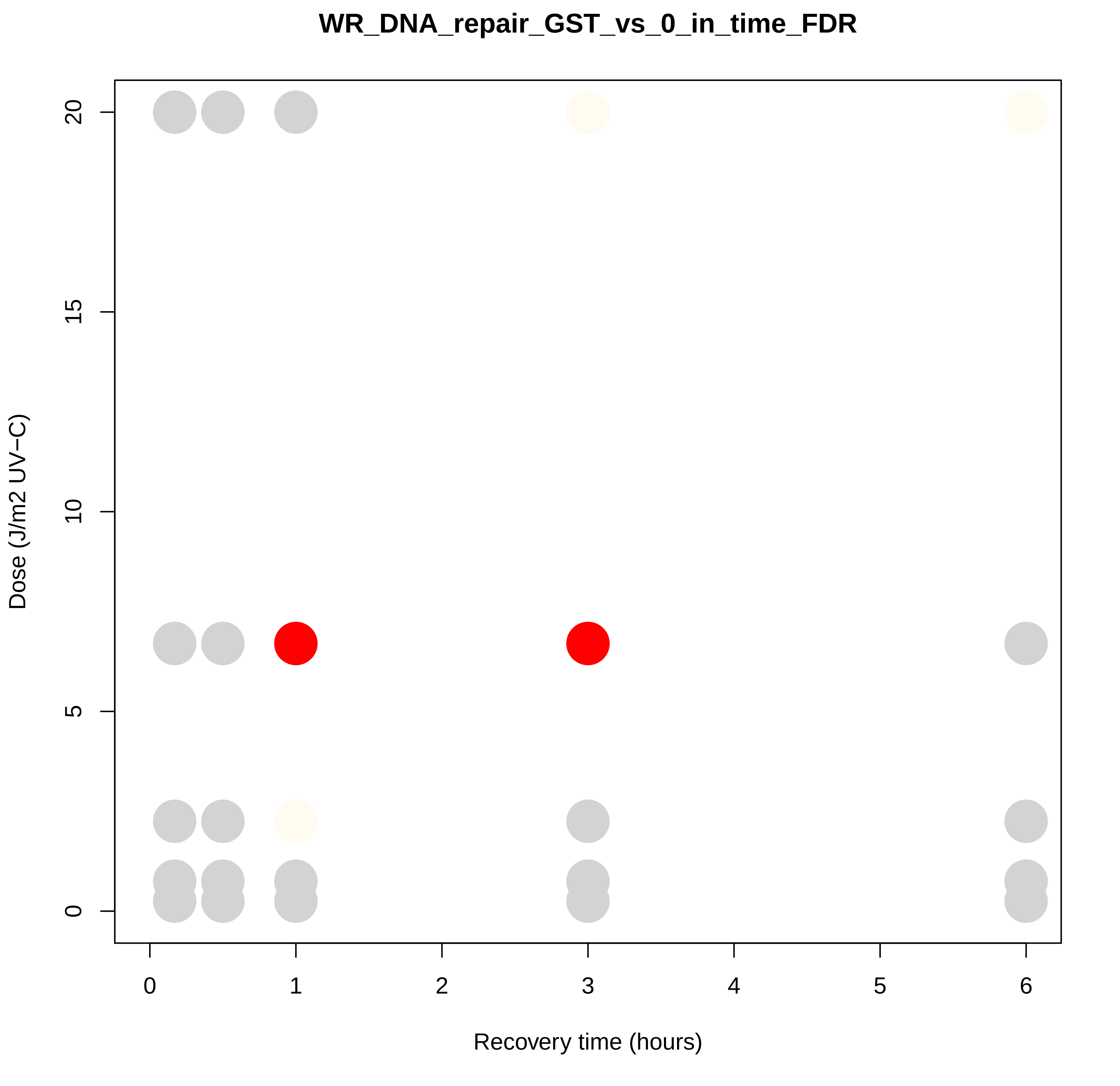
WR_DNA_repair_GST_vs_0_in_time_FDR
Dose (J/m2 UV−C)
0
5
10
15
20
0
1
2
3
4
5
6
Recovery time (hours)

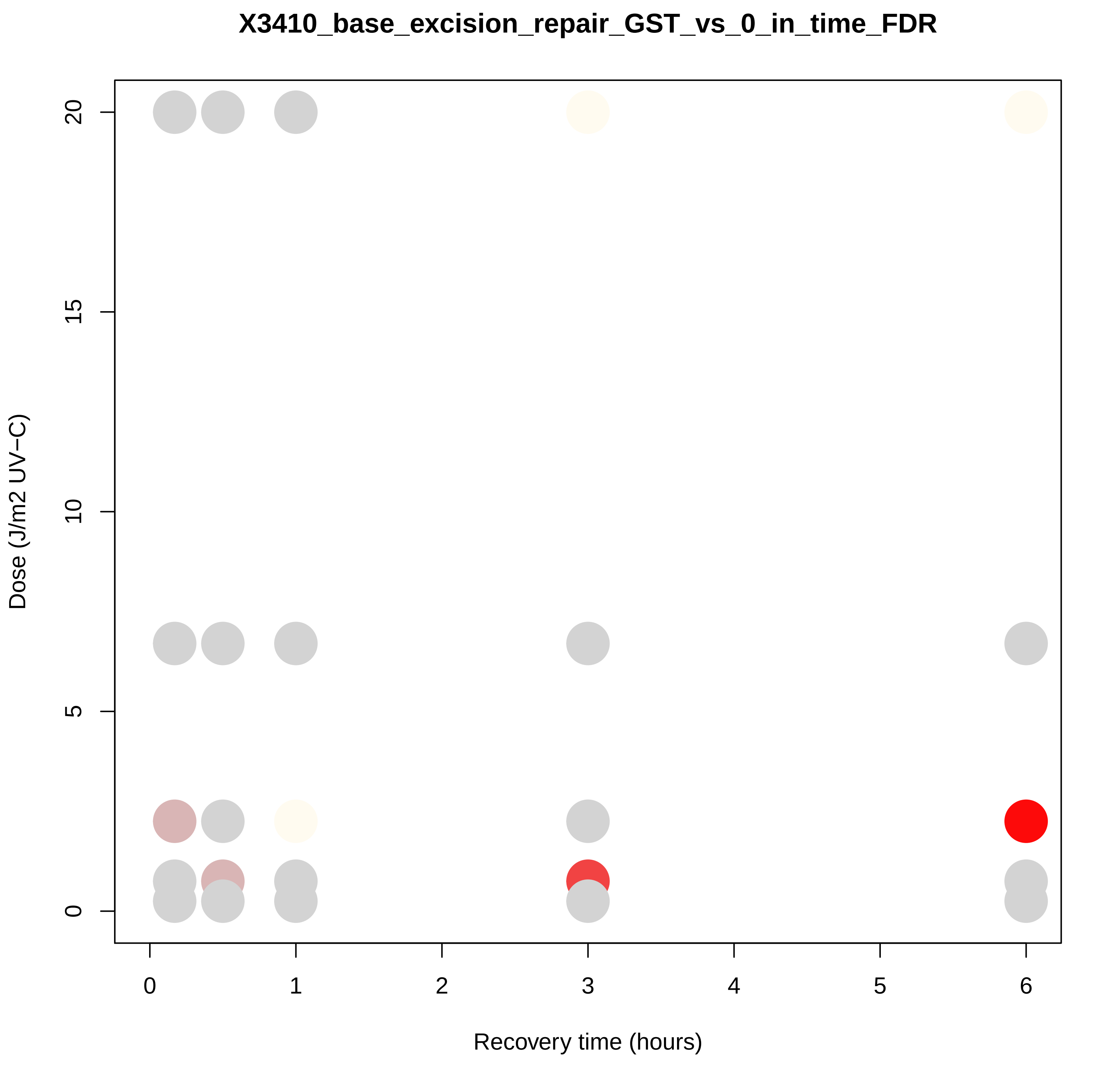

X3410_base_excision_repair_GST_vs_0_in_time_FDR
Dose (J/m2 UV−C)
0
5
10
15
20
Recovery time (hours)
0
1
2
3
4
5
6

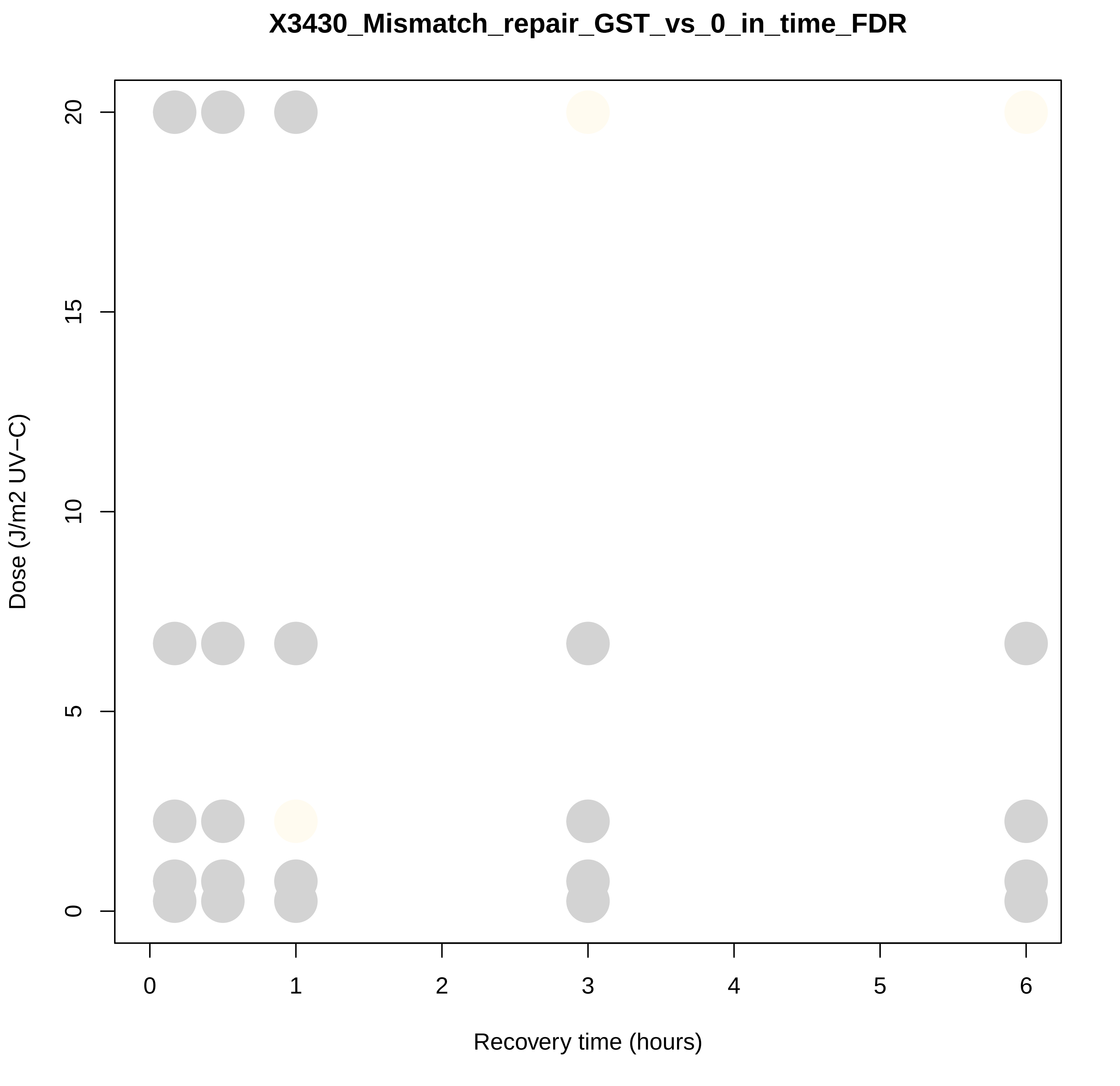
X3430_Mismatch_repair_GST_vs_0_in_time_FDR
Dose (J/m2 UV−C)
Recovery time (hours)
0
5
10
15
20
0
1
2
3
4
5
6

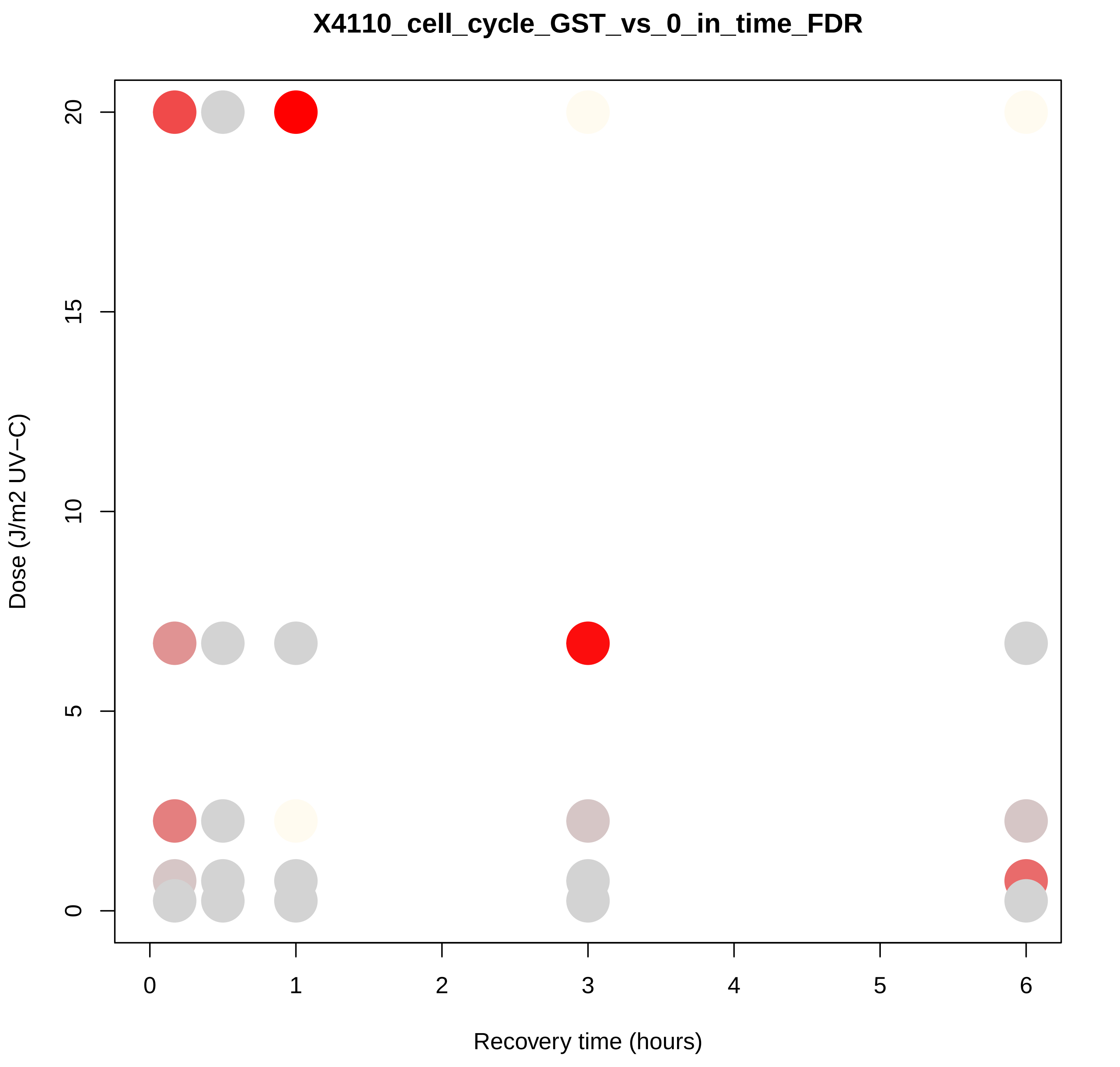

X4110_cell_cycle_GST_vs_0_in_time_FDR
Dose (J/m2 UV−C)
Recovery time (hours)
0
5
10
15
20
0
1
2
3
4
5
6

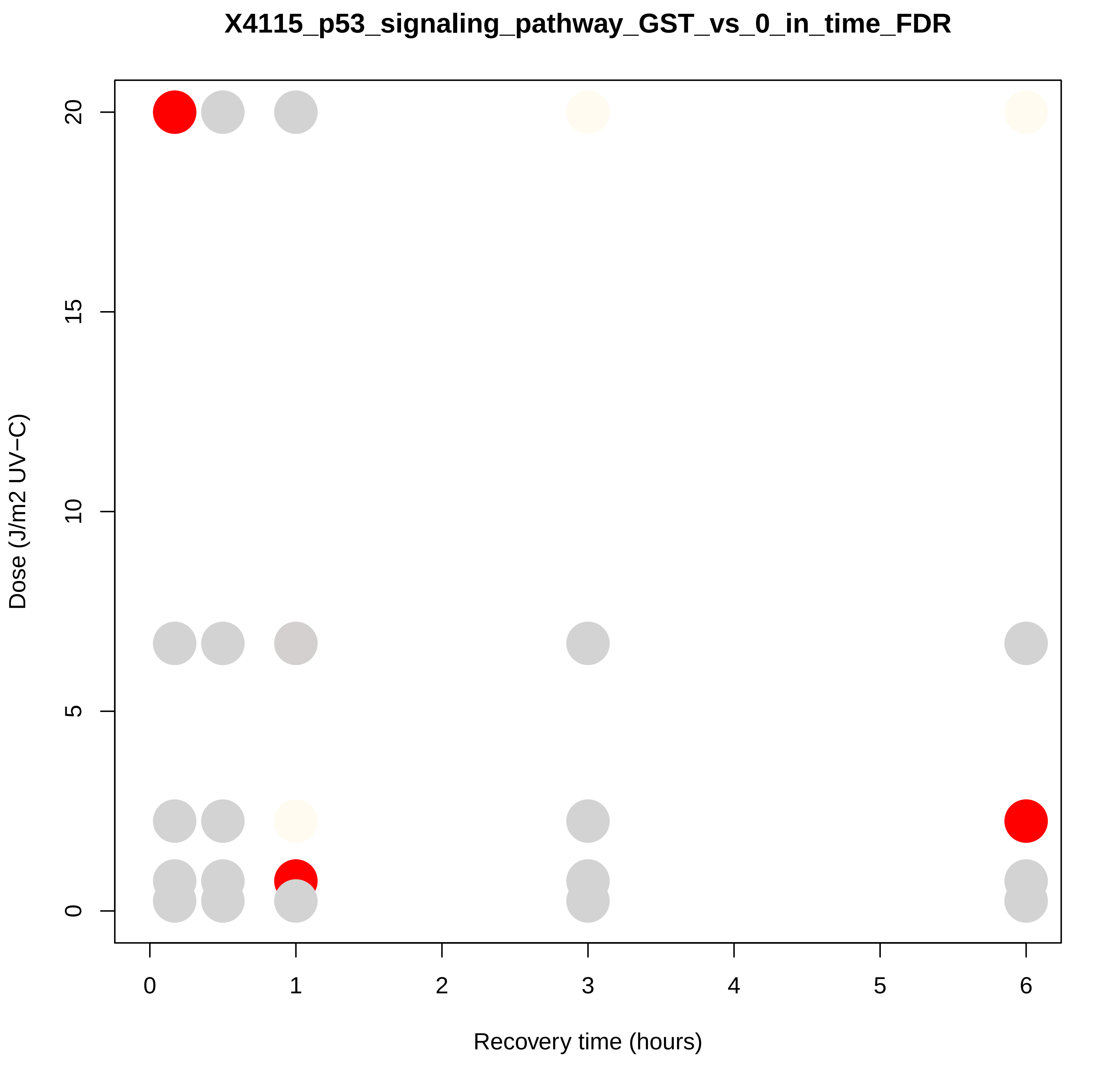
X4115_p53_signaling_pathway_GST_vs_0_in_time_FDR
Dose (J/m2 UV−C)
0
5
10
15
20
0
1
2
3
4
5
6
Recovery time (hours)

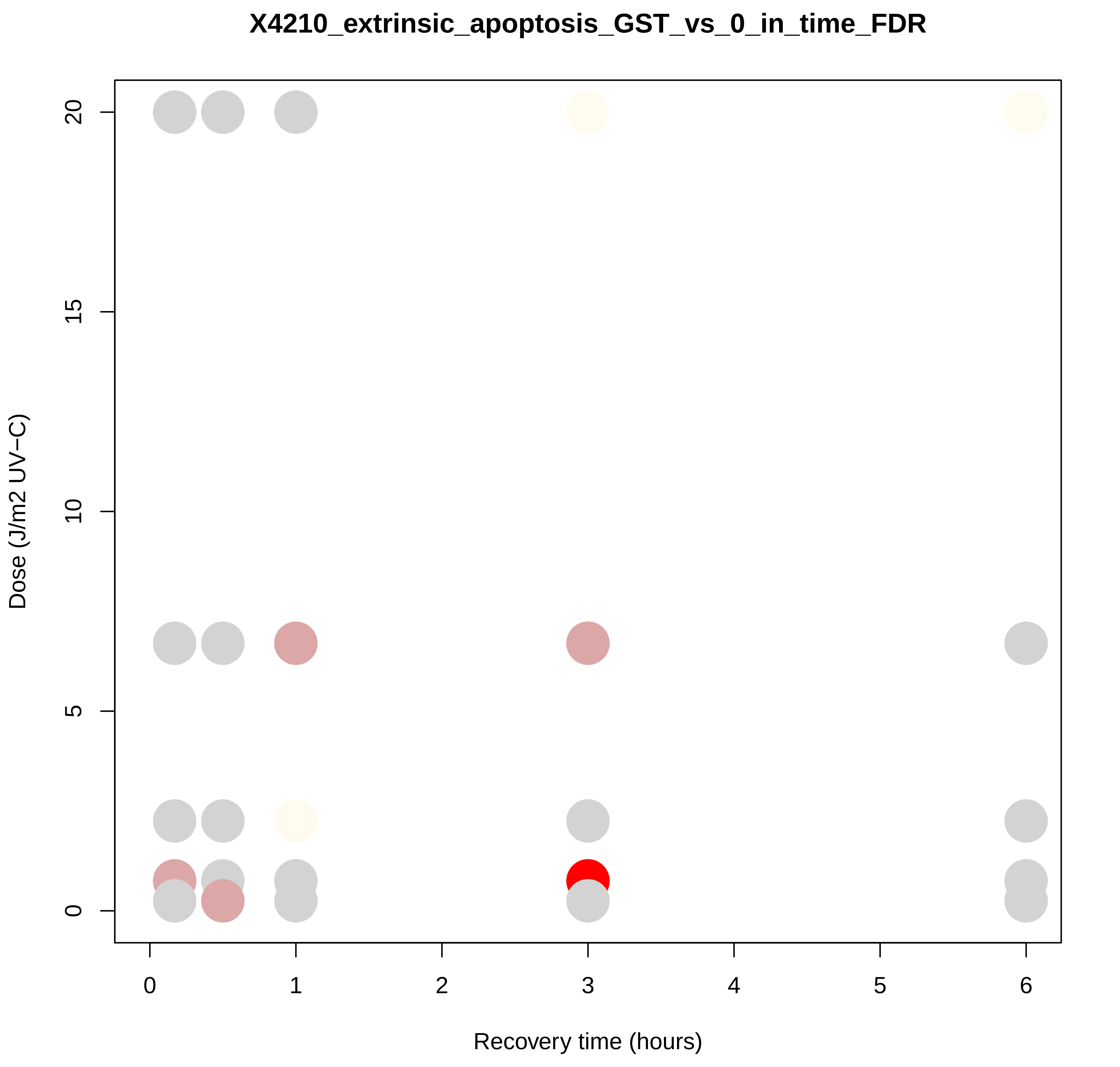
X4210_extrinsic_apoptosis_GST_vs_0_in_time_FDR
Dose (J/m2 UV−C)
Recovery time (hours)
0
5
10
15
20
0
1
2
3
4
5
6

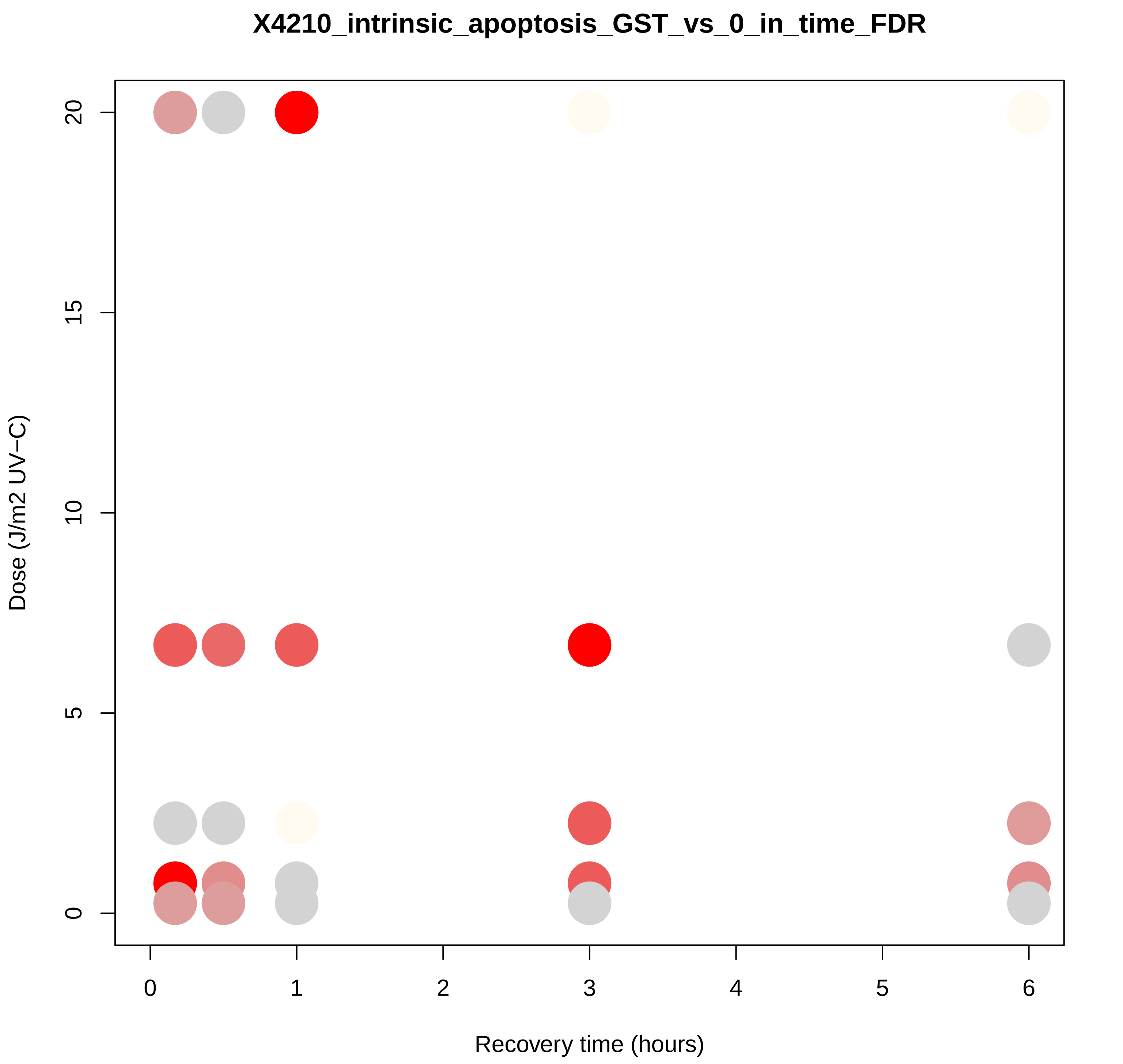

X4210_intrinsic_apoptosis_GST_vs_0_in_time_FDR
Dose (J/m2 UV−C)
Recovery time (hours)
0
5
10
15
20
0
1
2
3
4
5
6
