# Supplementary material for: A Range Finding Protocol to Support Design for Transcriptomics Experimentation: Examples of In-Vitro and In-Vivo Murine UV Exposure
Source: PLoS One. 2014 May 13;9(5):e97089. doi: 10.1371/journal.pone.0097089 (PMC4019648; doi:10.1371/journal.pone.0097089)
Supplement: Figure S4 — Cellular process specific responses in the in-vivo experiment design space. The potential sweet spots in the in-vivo range-finding experiment diagrams for all 64 tested gene sets (same set up as Figure 4A). (PDF) [file pone.0097089.s004.pdf]

Figure S4. Cellular process specific responses in the *in-vivo* experiment design space

The potential sweet spots in the *in-vivo* range-finding experiment diagrams for all 64 tested gene sets (same set up as Figure 4A).

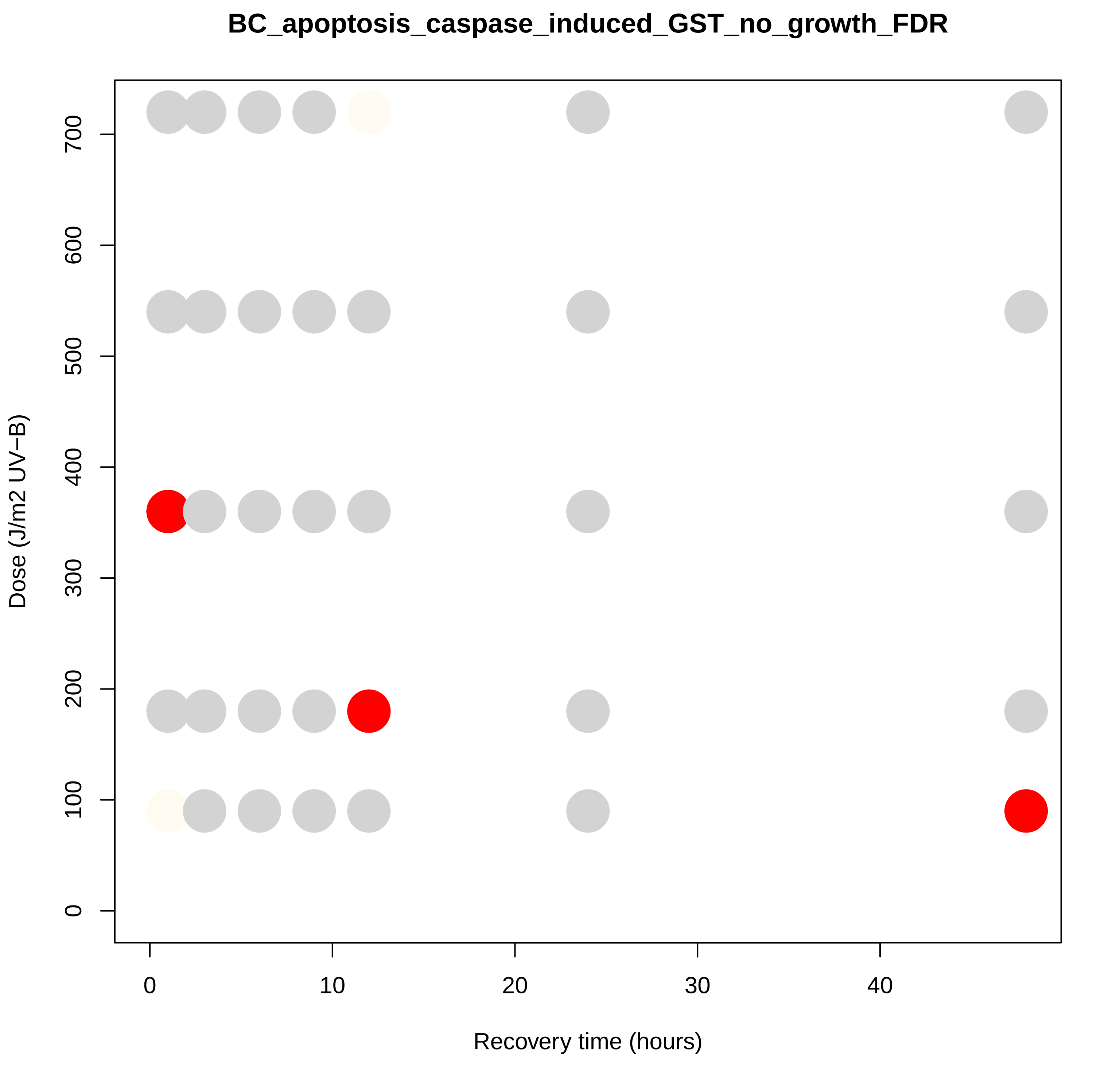
BC_apoptosis_caspase_induced_GST_no_growth_FDR
Dose (J/m2 UV−B)
0
100
200
300
400
500
600
700
Recovery time (hours)
0
10
20
30
40

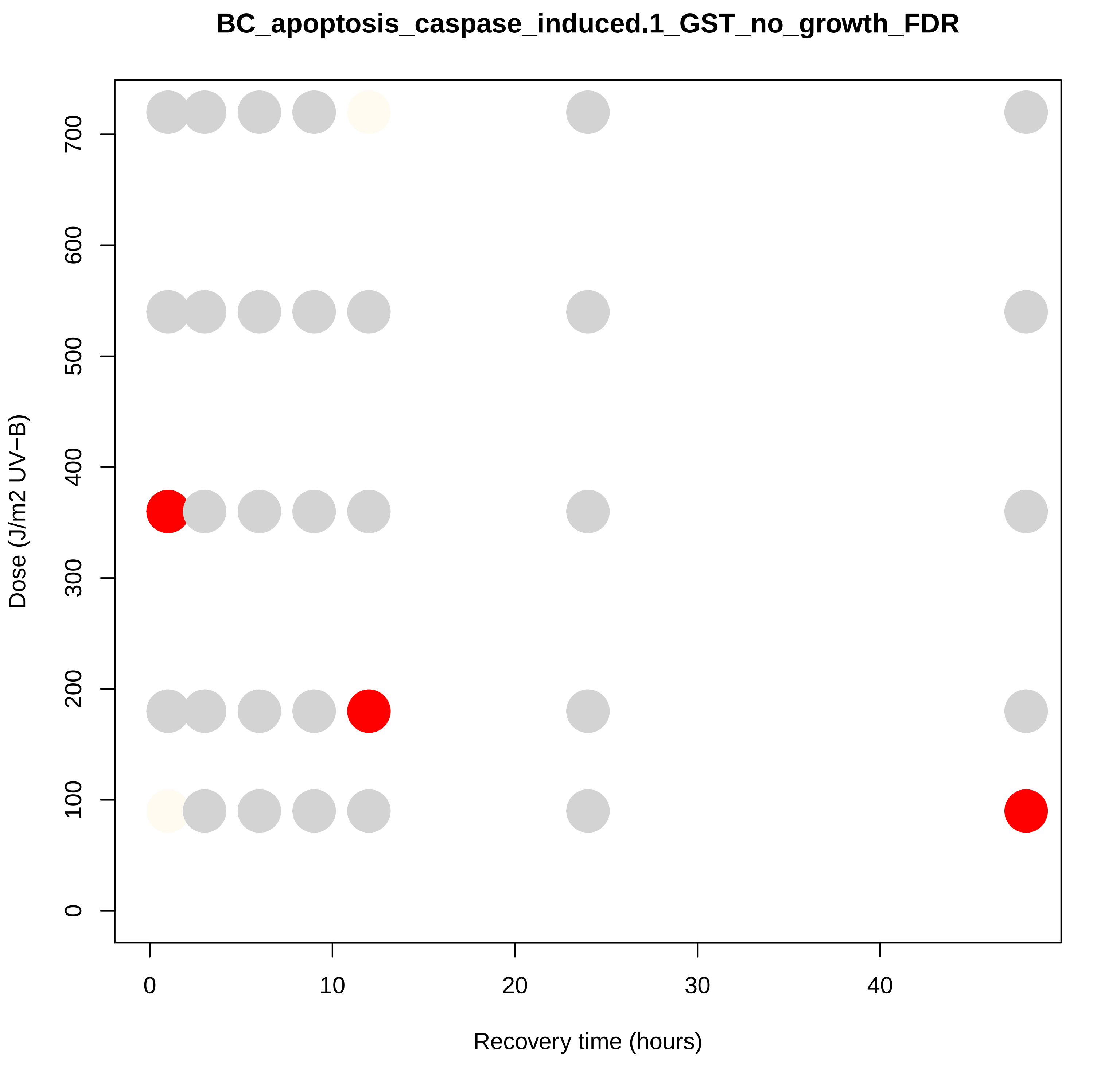
BC_apoptosis_caspase_induced.1_GST_no_growth_FDR
Dose (J/m2 UV−B)
0
100
200
300
400
500
600
700
0
10
20
30
40
Recovery time (hours)

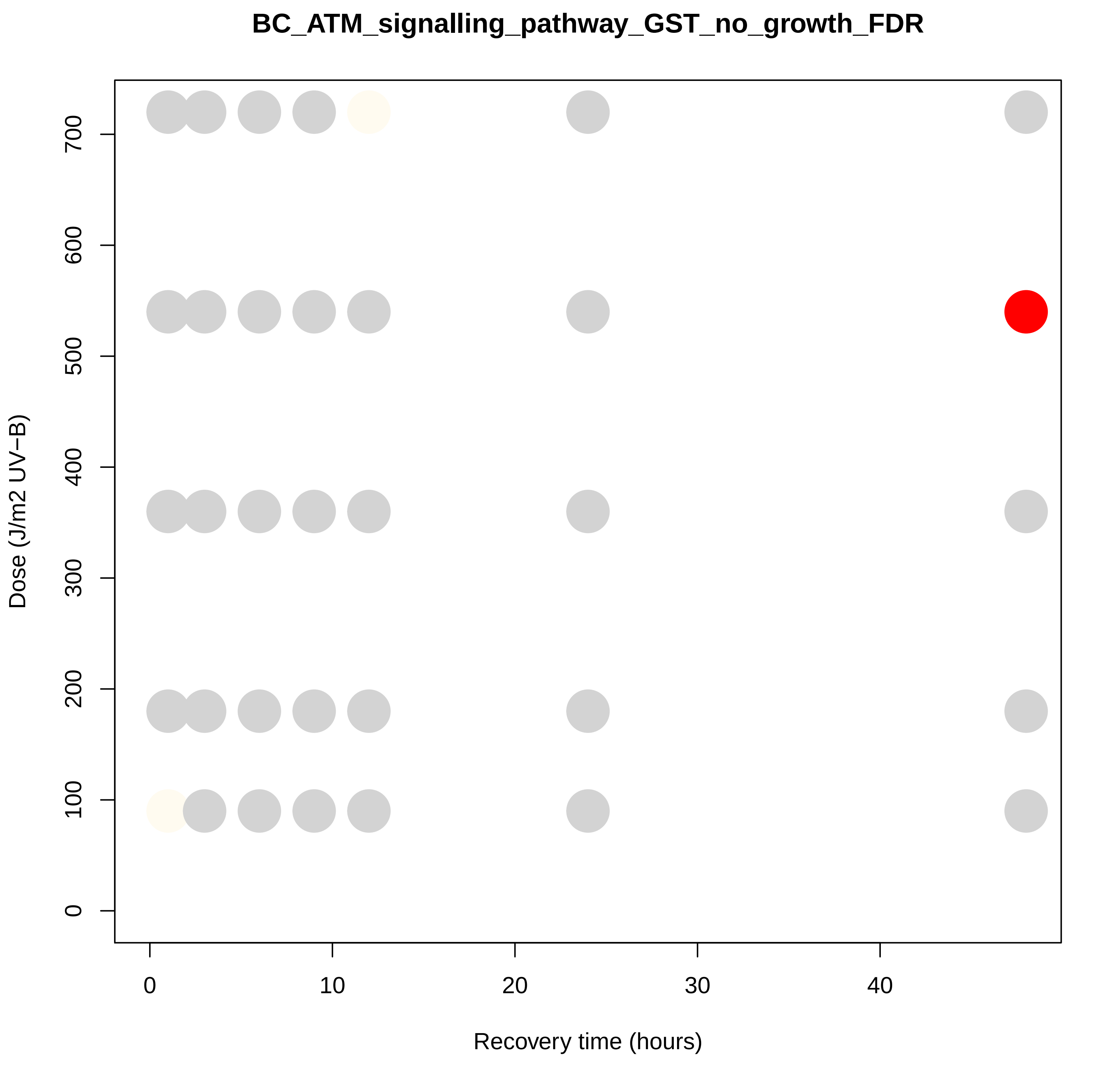

BC_ATM_signalling_pathway_GST_no_growth_FDR
Dose (J/m2 UV−B)
0
100
200
300
400
500
600
700
0
10
20
30
40
Recovery time (hours)

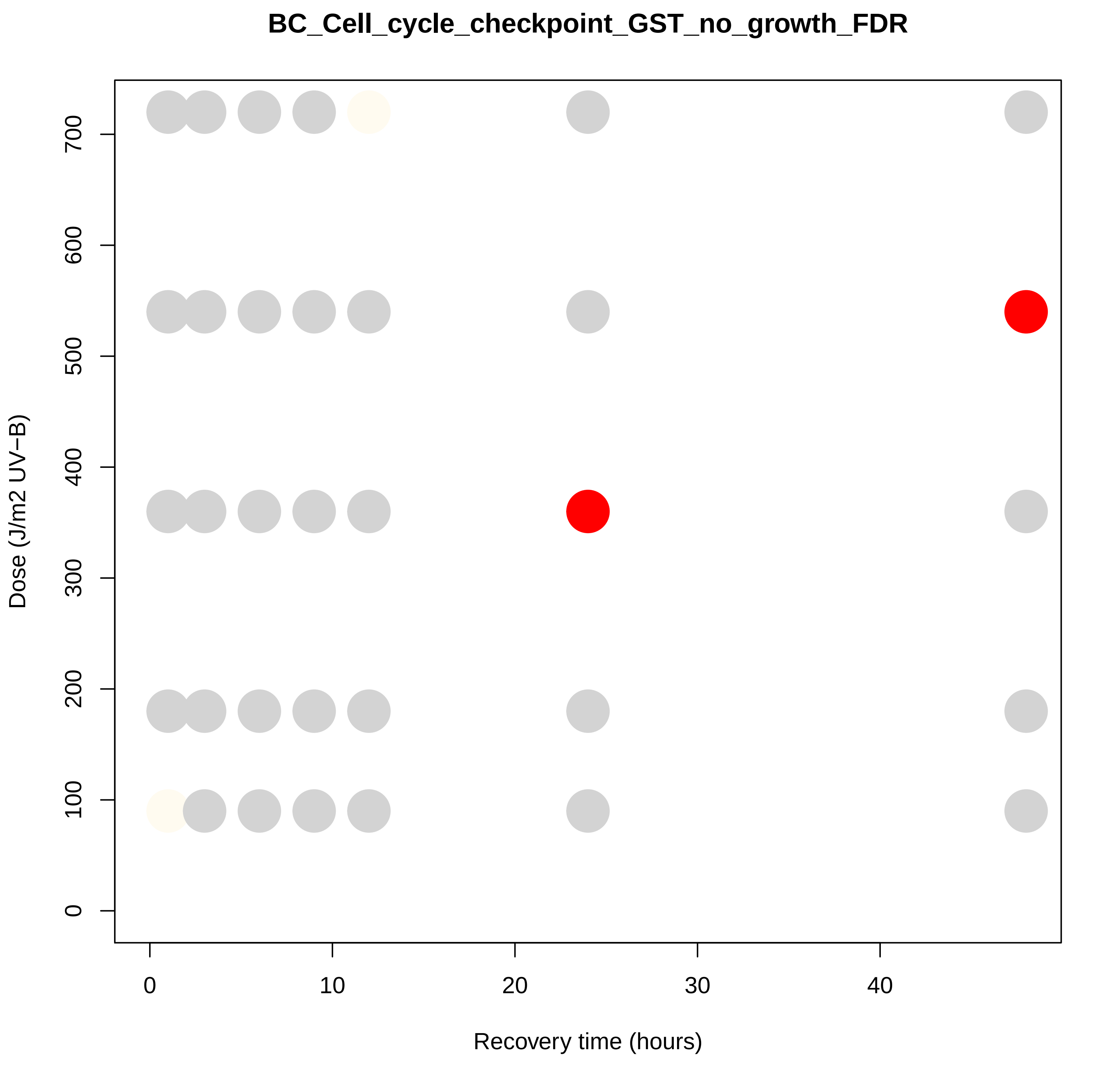
BC_Cell_cycle_checkpoint_GST_no_growth_FDR
Dose (J/m2 UV−B)
0
100
200
300
400
500
600
700
Recovery time (hours)
0
10
20
30
40

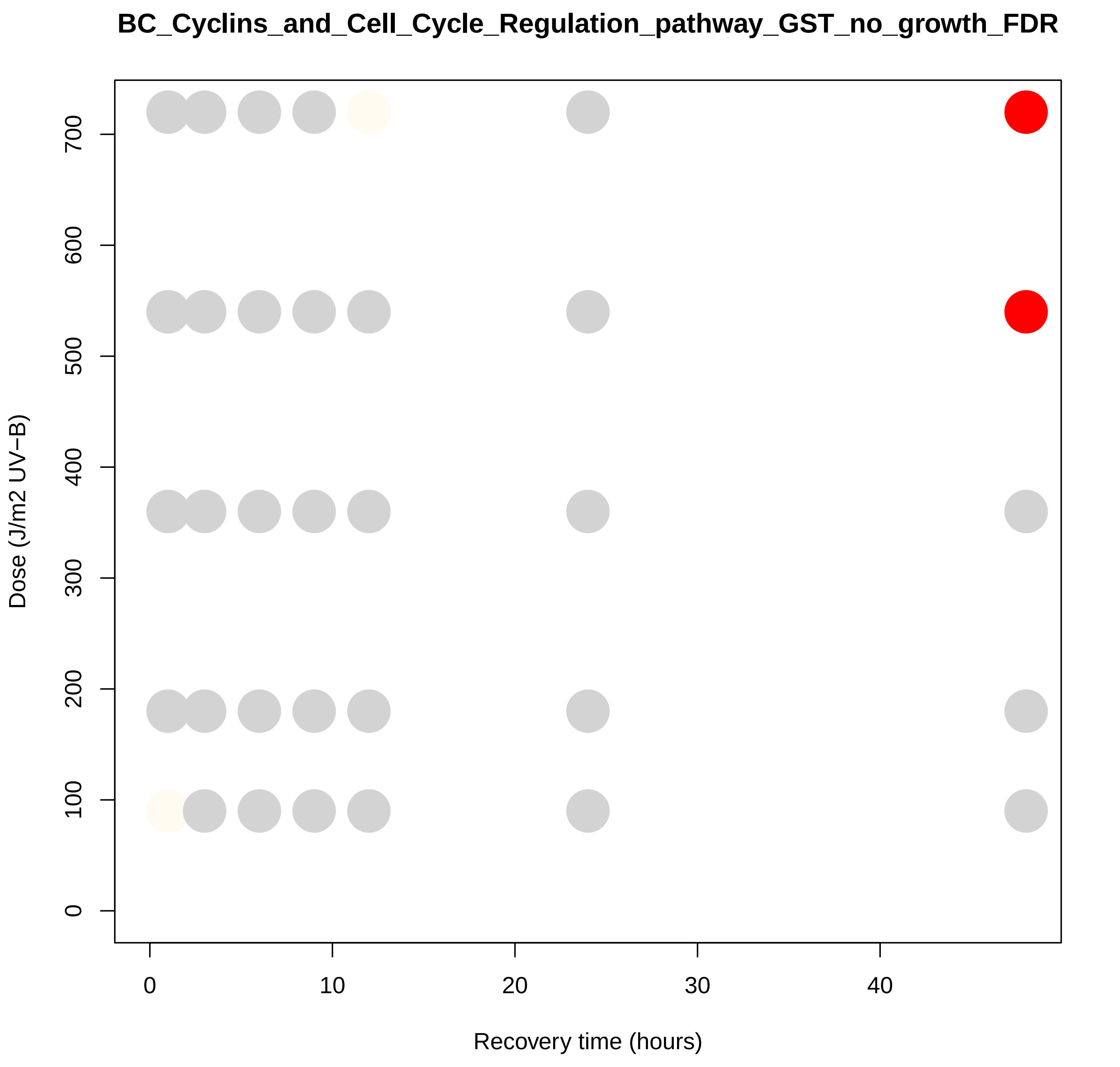
BC_Cyclins_and_Cell_Cycle_Regulation_pathway_GST_no_growth_FDR
Dose (J/m2 UV−B)
0
100
200
300
400
500
600
700
0
10
20
30
40
Recovery time (hours)

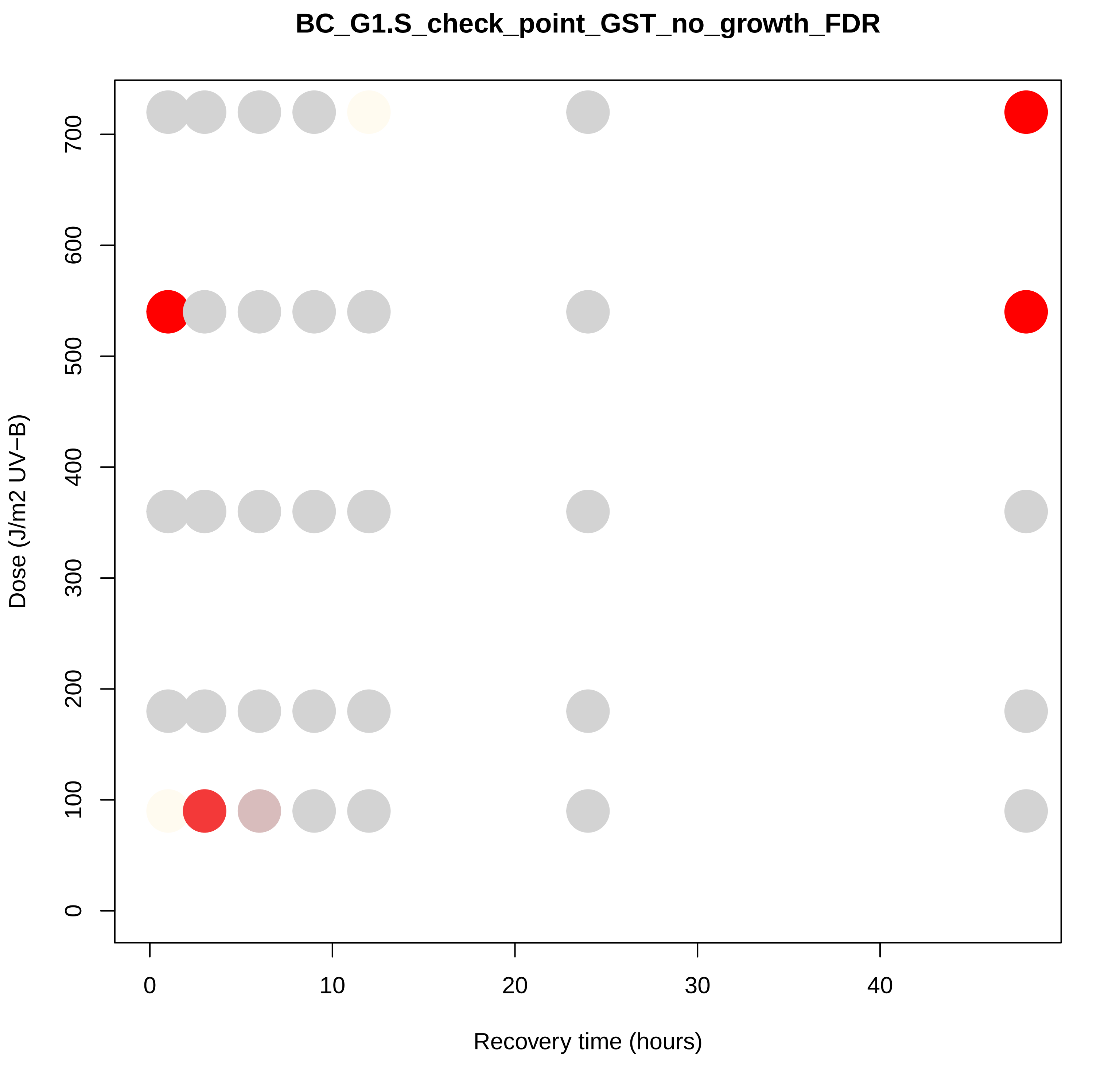

BC_G1.S_check_point_GST_no_growth_FDR
Dose (J/m2 UV−B)
Recovery time (hours)
0
100
200
300
400
500
600
700
0
10
20
30
40

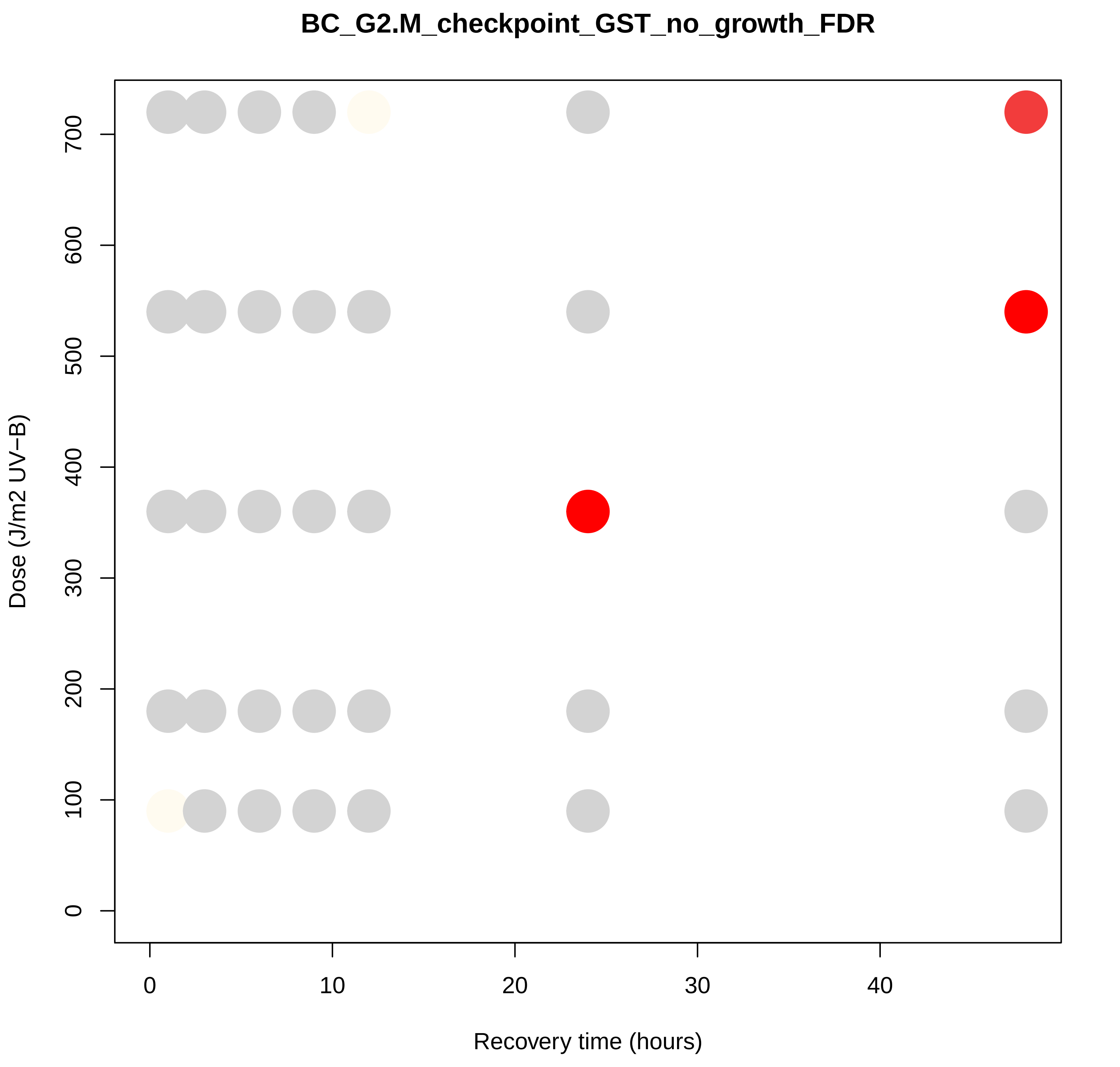
BC_G2.M_checkpoint_GST_no_growth_FDR
Dose (J/m2 UV−B)
Recovery time (hours)
0
100
200
300
400
500
600
700
0
10
20
30
40

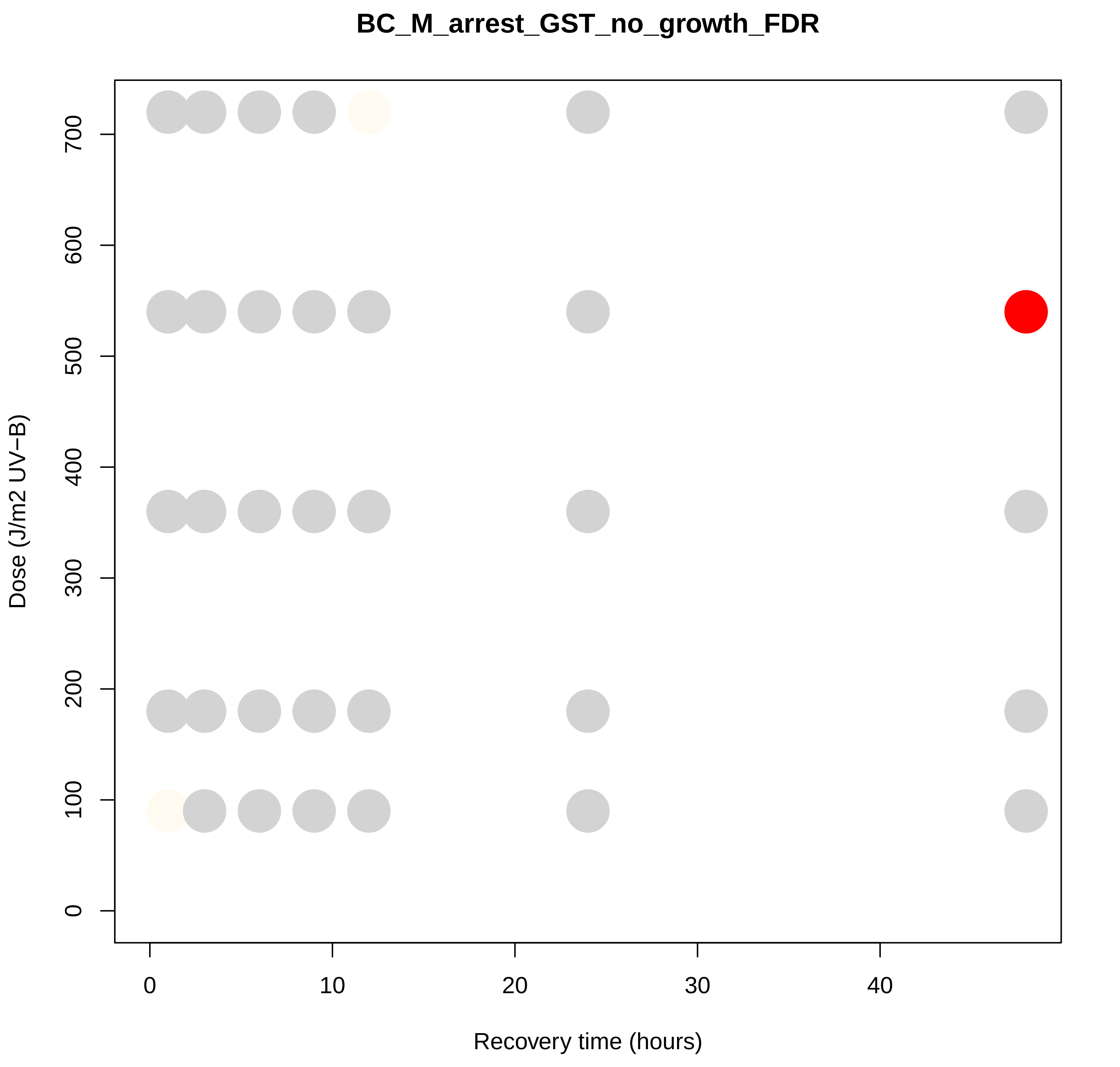
BC_M_arrest_GST_no_growth_FDR
Dose (J/m2 UV−B)
0
100
200
300
400
500
600
700
0
10
20
30
40
Recovery time (hours)

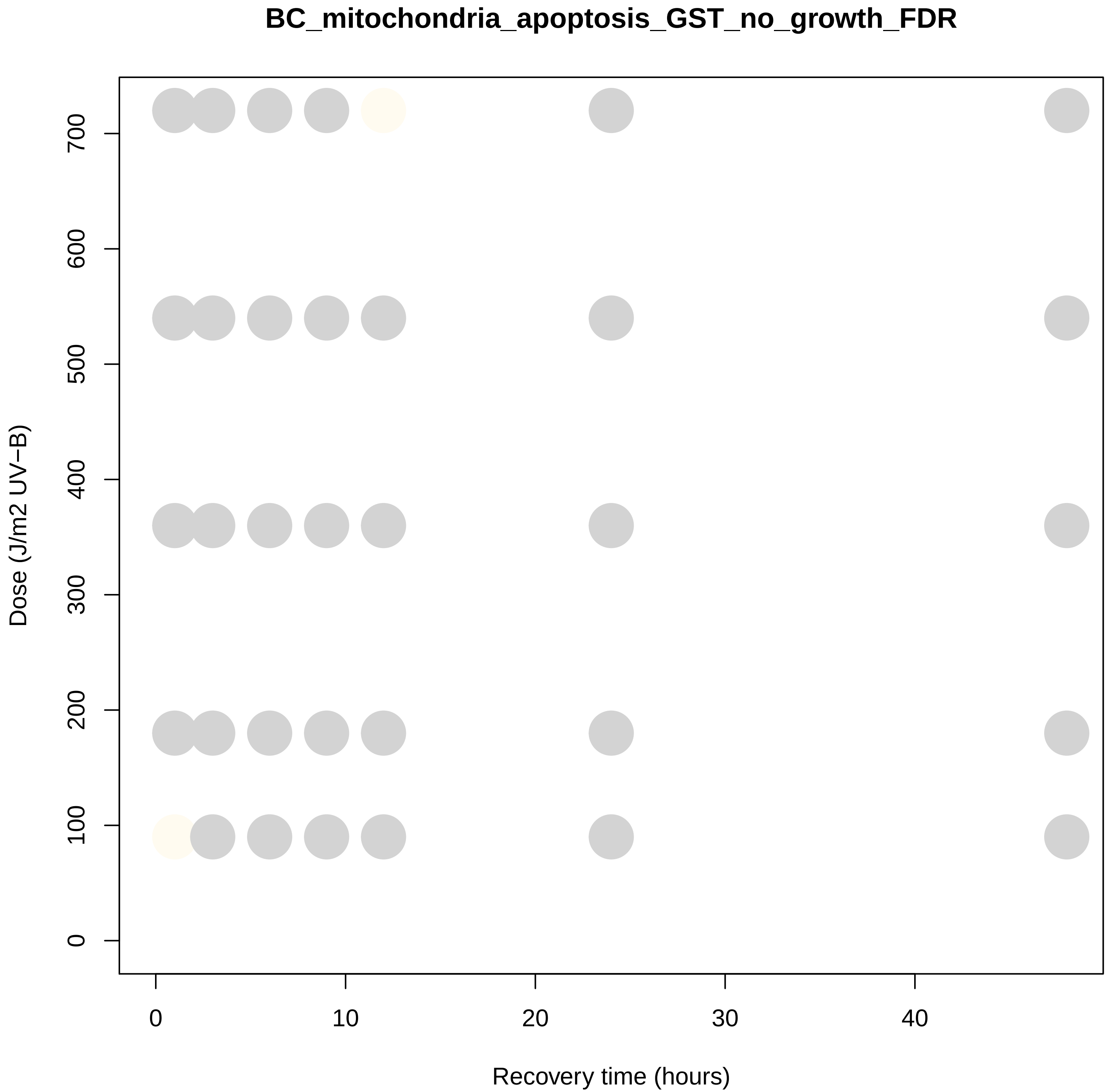

BC_mitochondria_apoptosis_GST_no_growth_FDR
Dose (J/m2 UV−B)
0
100
200
300
400
500
600
700
Recovery time (hours)
0
10
20
30
40

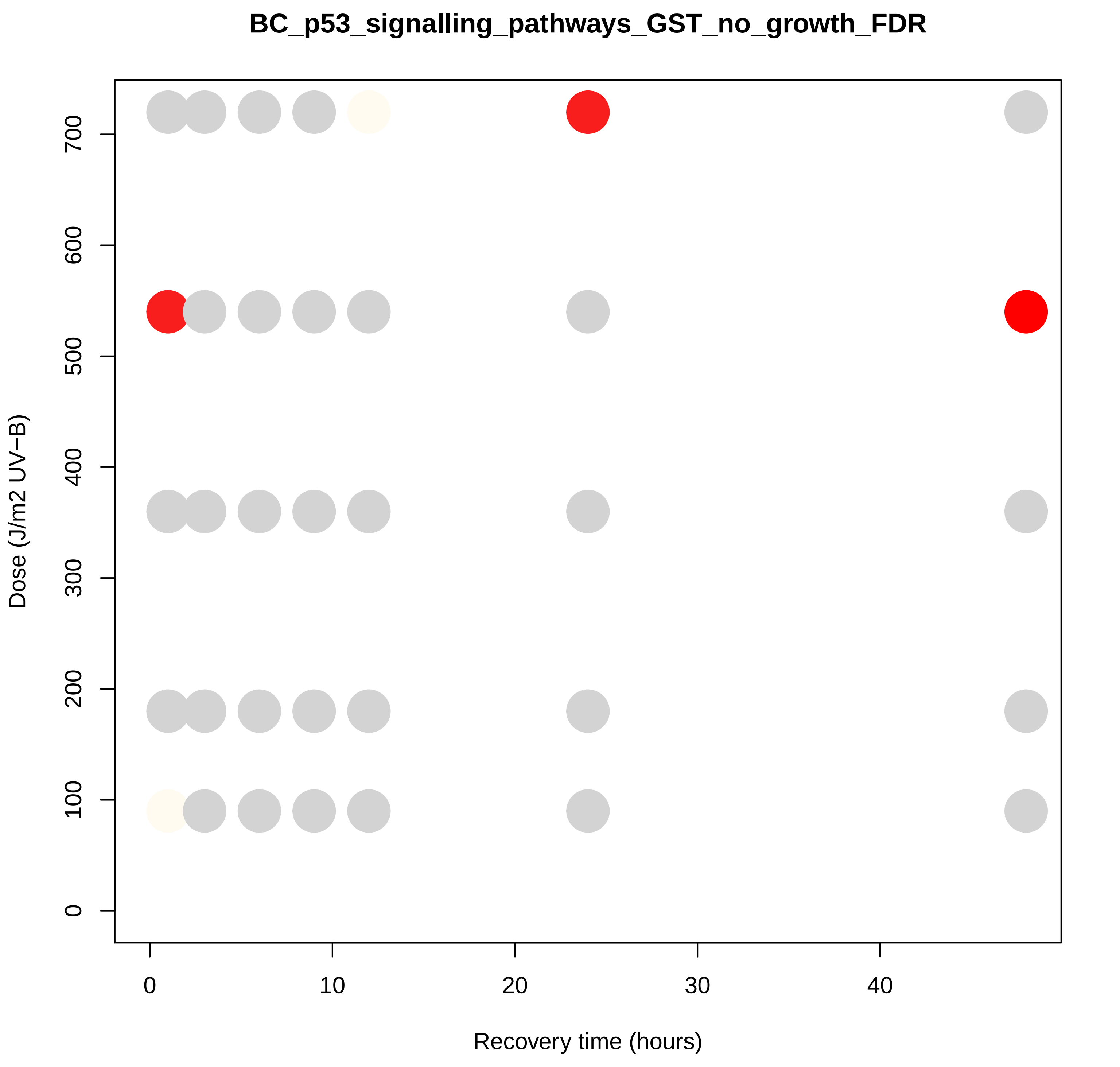

BC_p53_signalling_pathways_GST_no_growth_FDR
Dose (J/m2 UV−B)
0
100
200
300
400
500
600
700
Recovery time (hours)
0
10
20
30
40

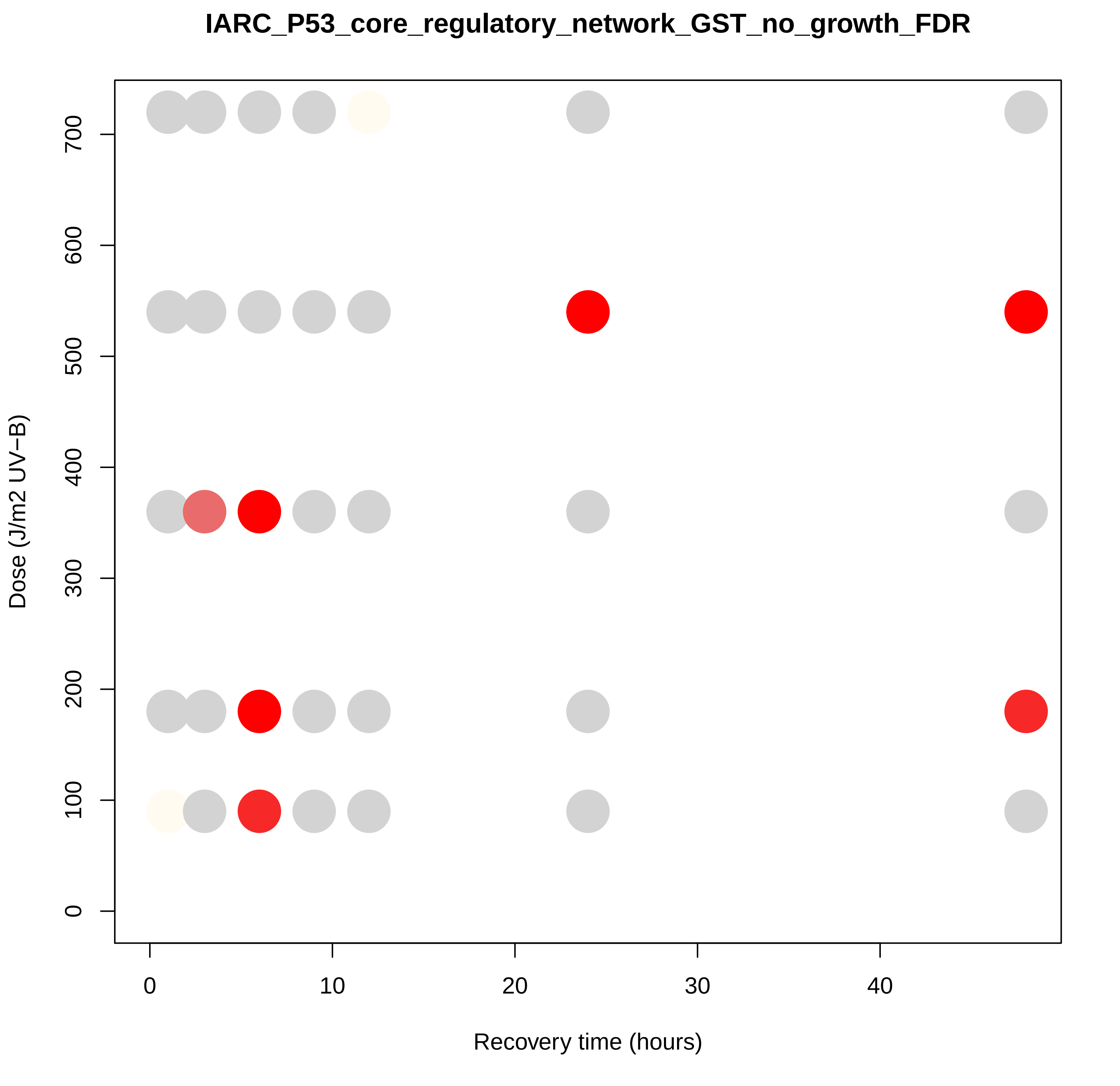

IARC_P53_core_regulatory_network_GST_no_growth_FDR
Dose (J/m2 UV−B)
0
100
200
300
400
500
600
700
Recovery time (hours)
0
10
20
30
40

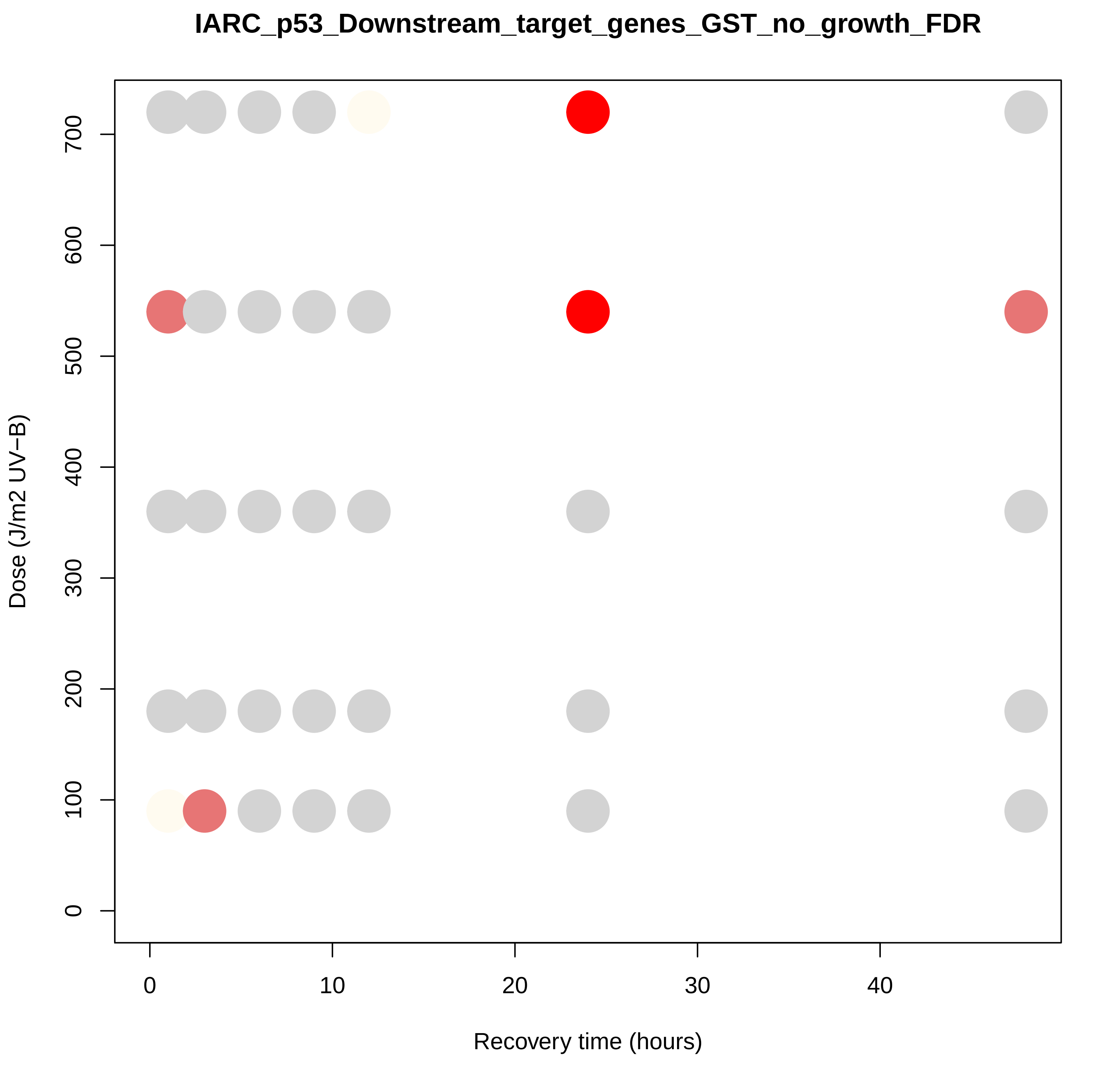
IARC_p53_Downstream_target_genes_GST_no_growth_FDR
Dose (J/m2 UV−B)
0
100
200
300
400
500
600
700
Recovery time (hours)
0
10
20
30
40

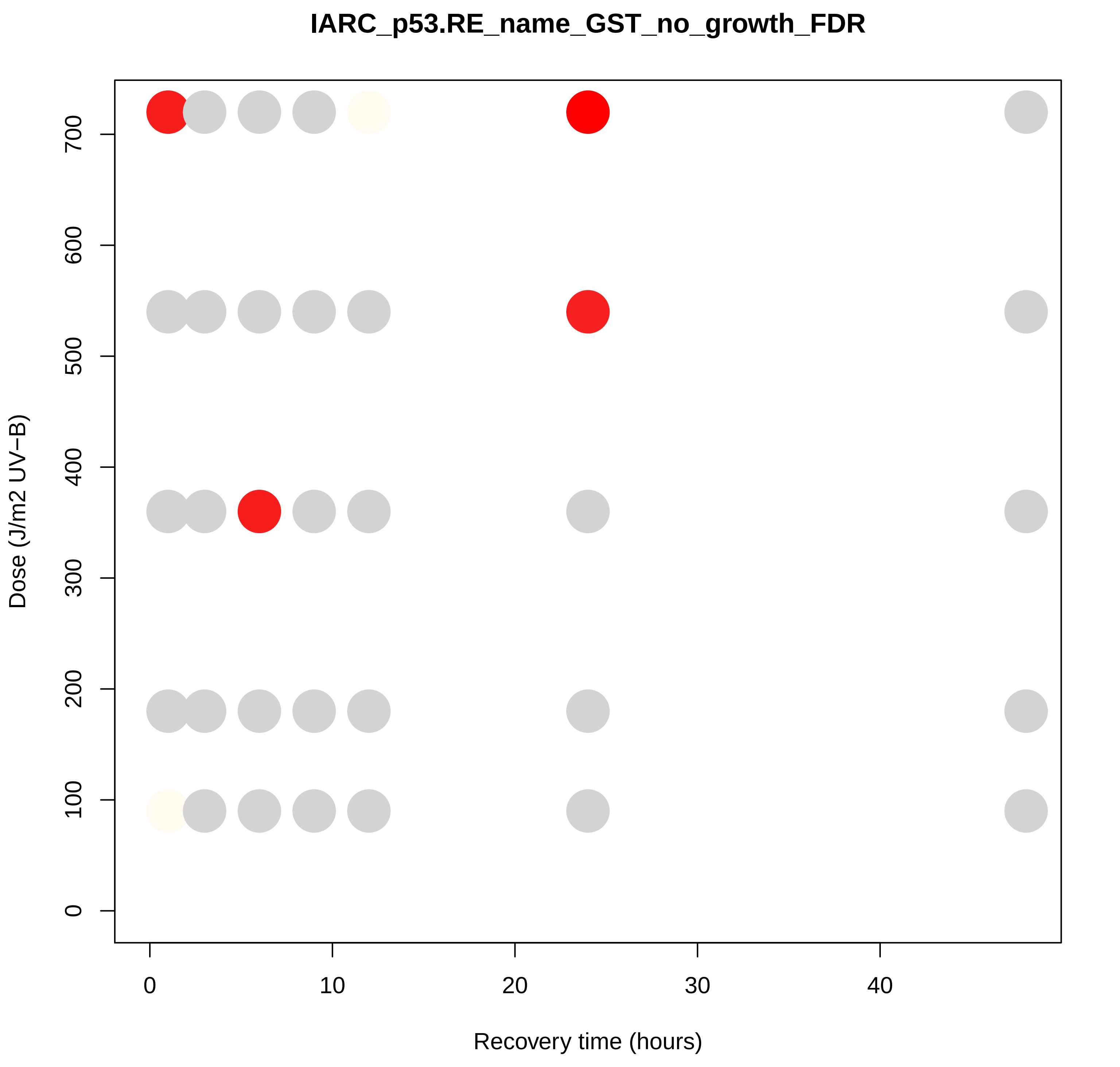

IARC_p53.RE_name_GST_no_growth_FDR
Dose (J/m2 UV−B)
0
100
200
300
400
500
600
700
Recovery time (hours)
0
10
20
30
40

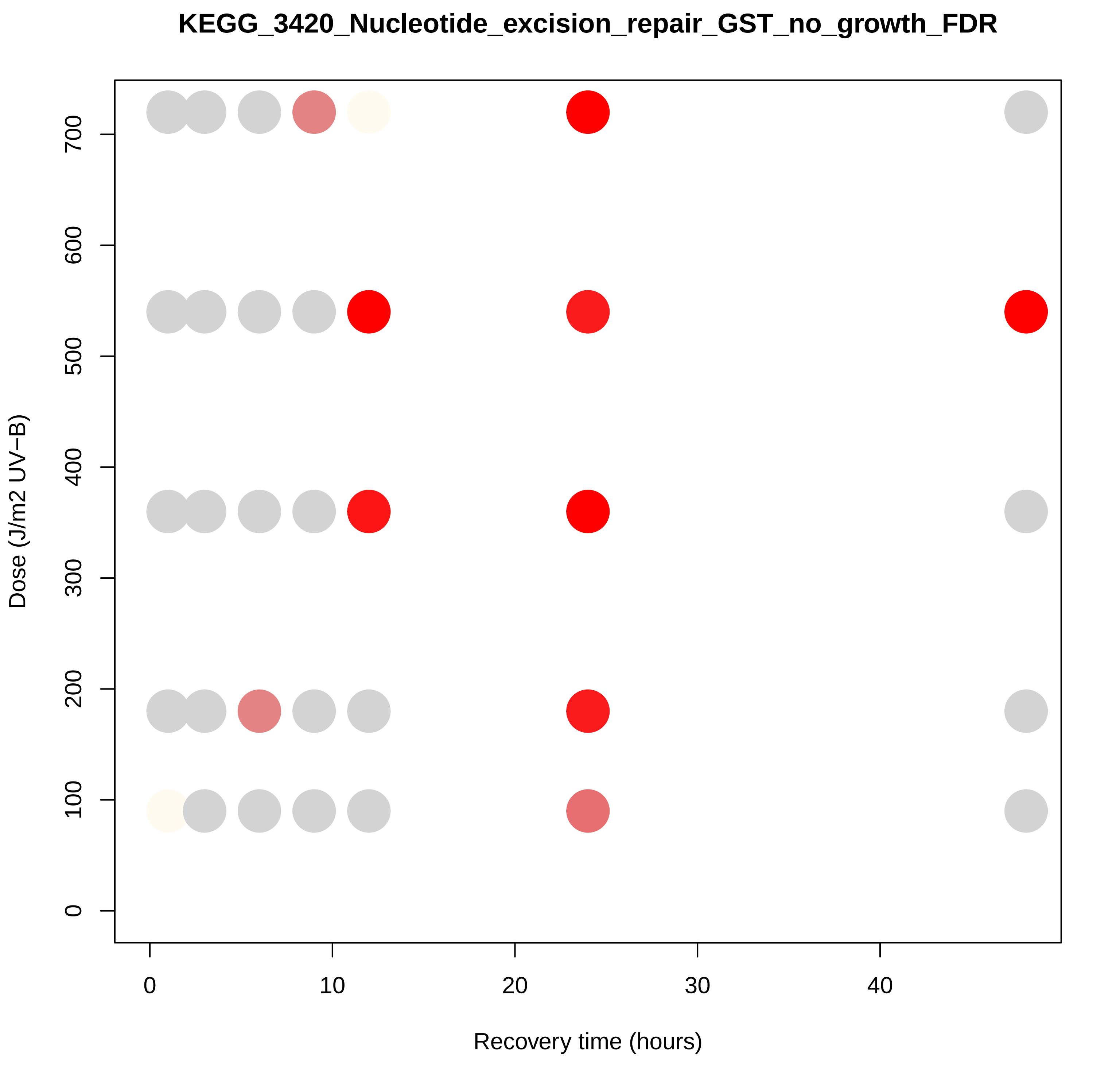
KEGG_3420_Nucleotide_excision_repair_GST_no_growth_FDR
Dose (J/m2 UV−B)
0
100
200
300
400
500
600
700
0
10
20
30
40
Recovery time (hours)

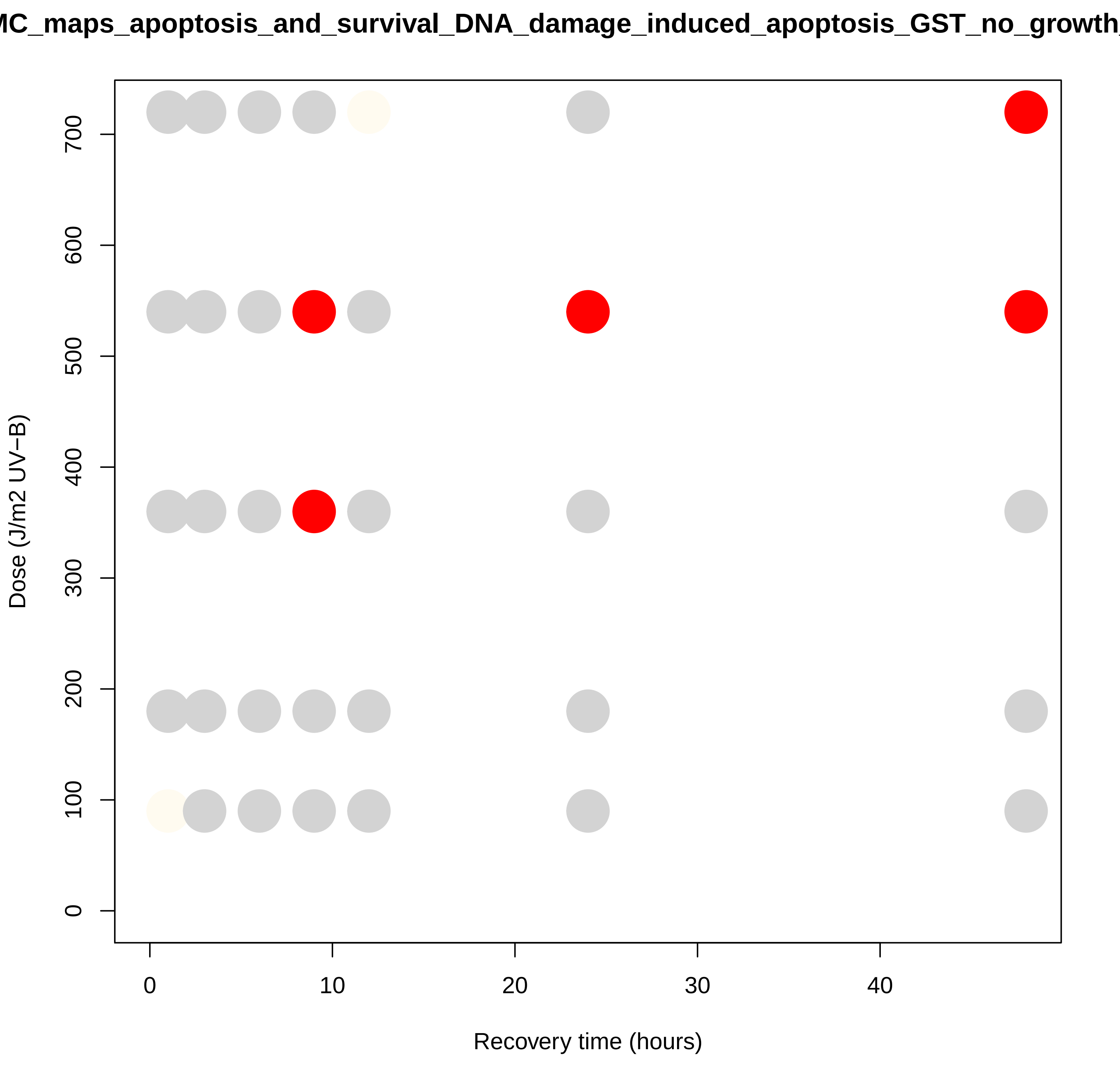
MC_maps_apoptosis_and_survival_DNA_damage_induced_apoptosis_GST_no_growth
Dose (J/m2 UV−B)
Recovery time (hours)
0
100
200
300
400
500
600
700
0
10
20
30
40

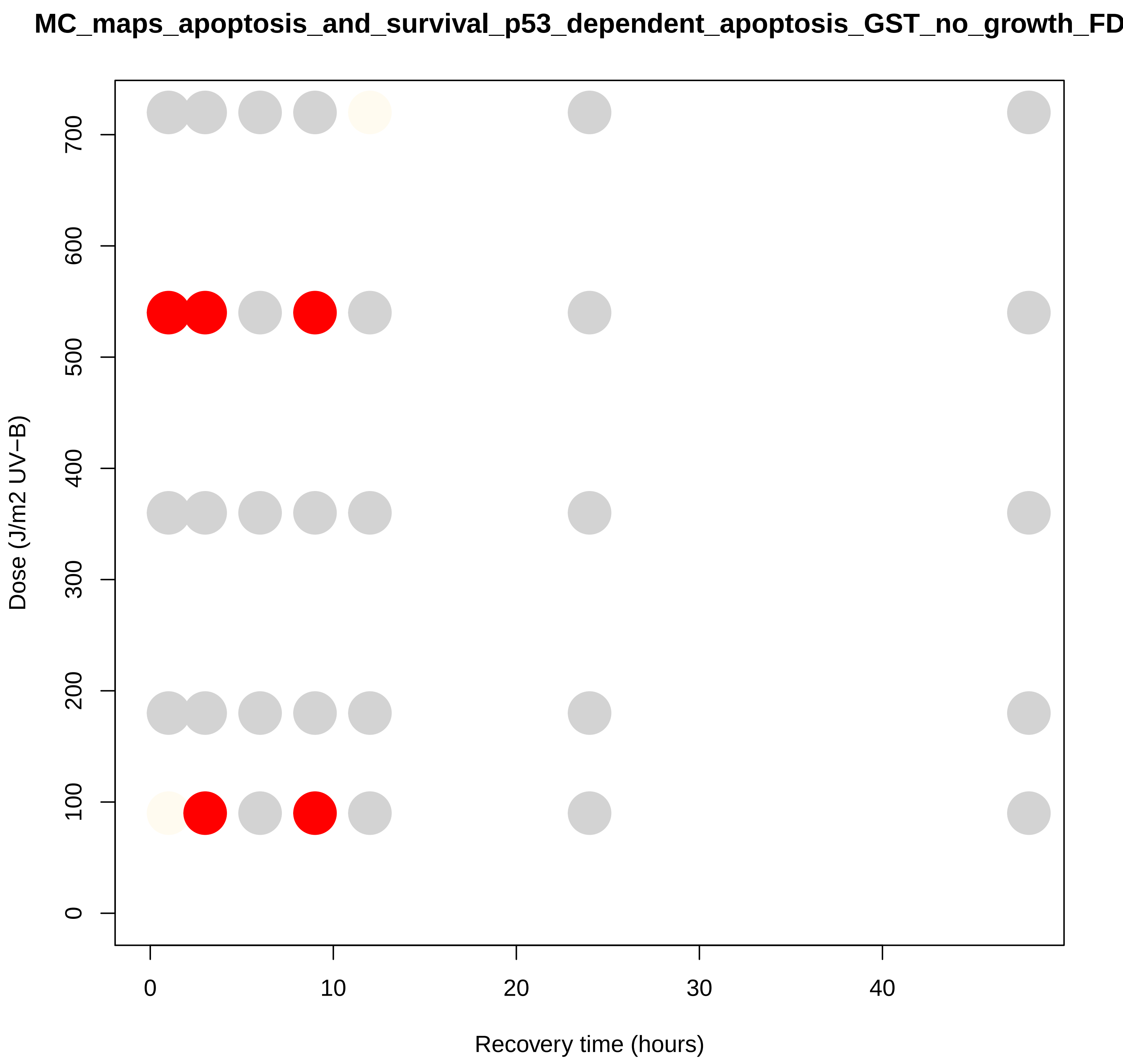

MC_maps_apoptosis_and_survival_p53_dependent_apoptosis_GST_no_growth_FD
Dose (J/m2 UV−B)
0
100
200
300
400
500
600
700
0
10
20
30
40
Recovery time (hours)

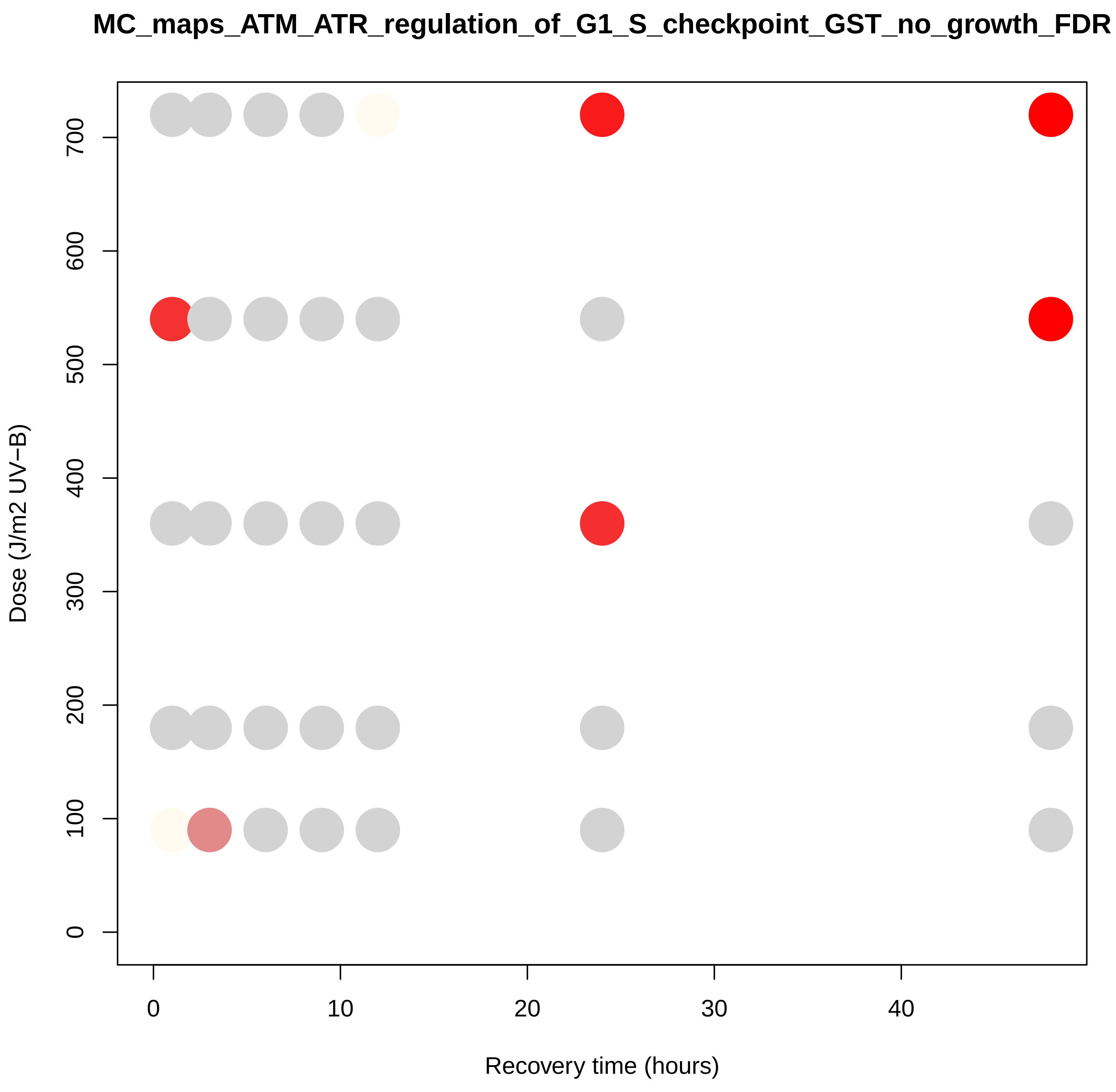

MC_maps_ATM_ATR_regulation_of_G1_S_checkpoint_GST_no_growth_FDR
Dose (J/m2 UV−B)
0
100
200
300
400
500
600
700
Recovery time (hours)
0
10
20
30
40

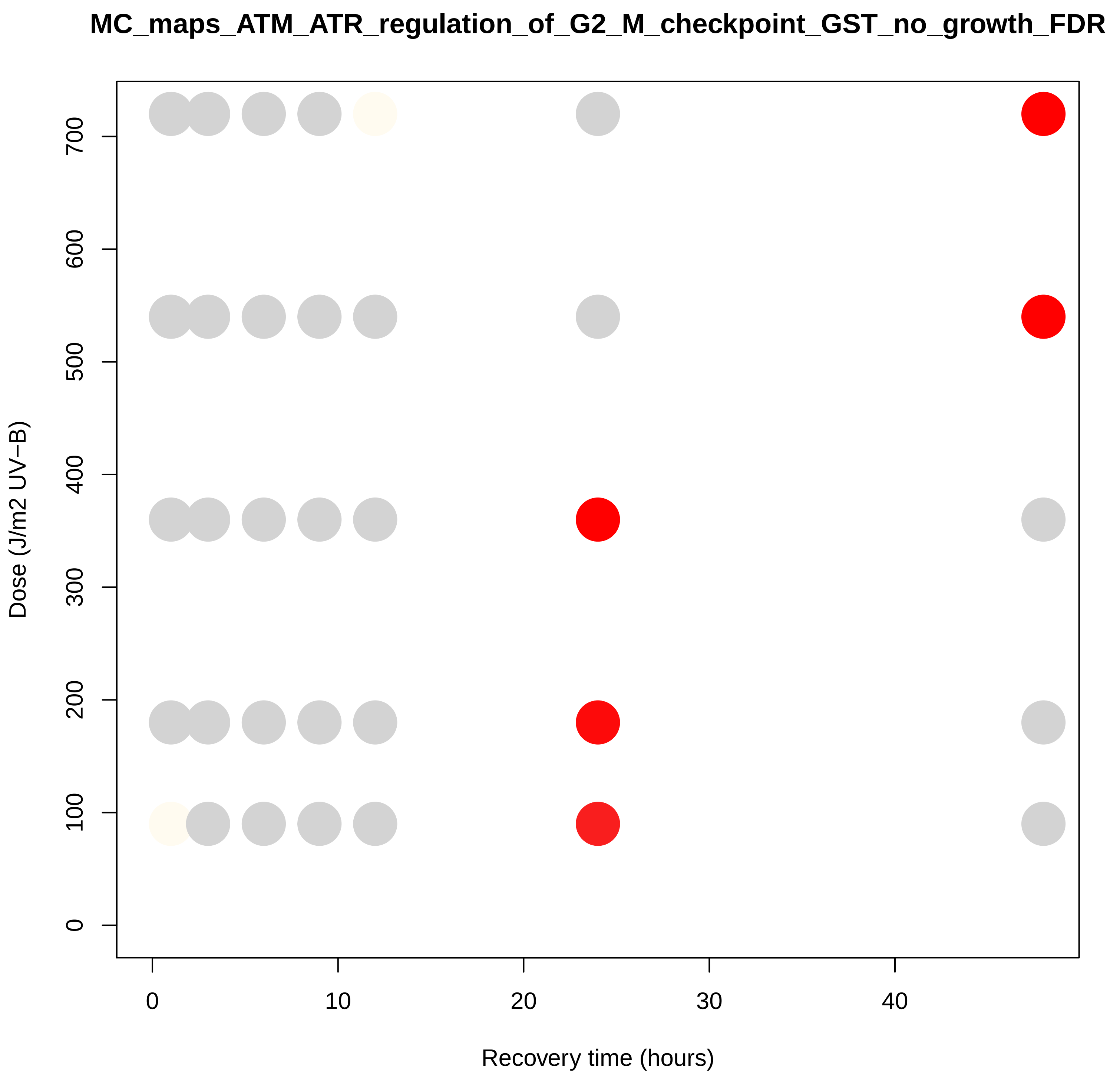

MC_maps_ATM_ATR_regulation_of_G2_M_checkpoint_GST_no_growth_FDR
Dose (J/m2 UV−B)
0
100
200
300
400
500
600
700
0
10
20
30
40
Recovery time (hours)

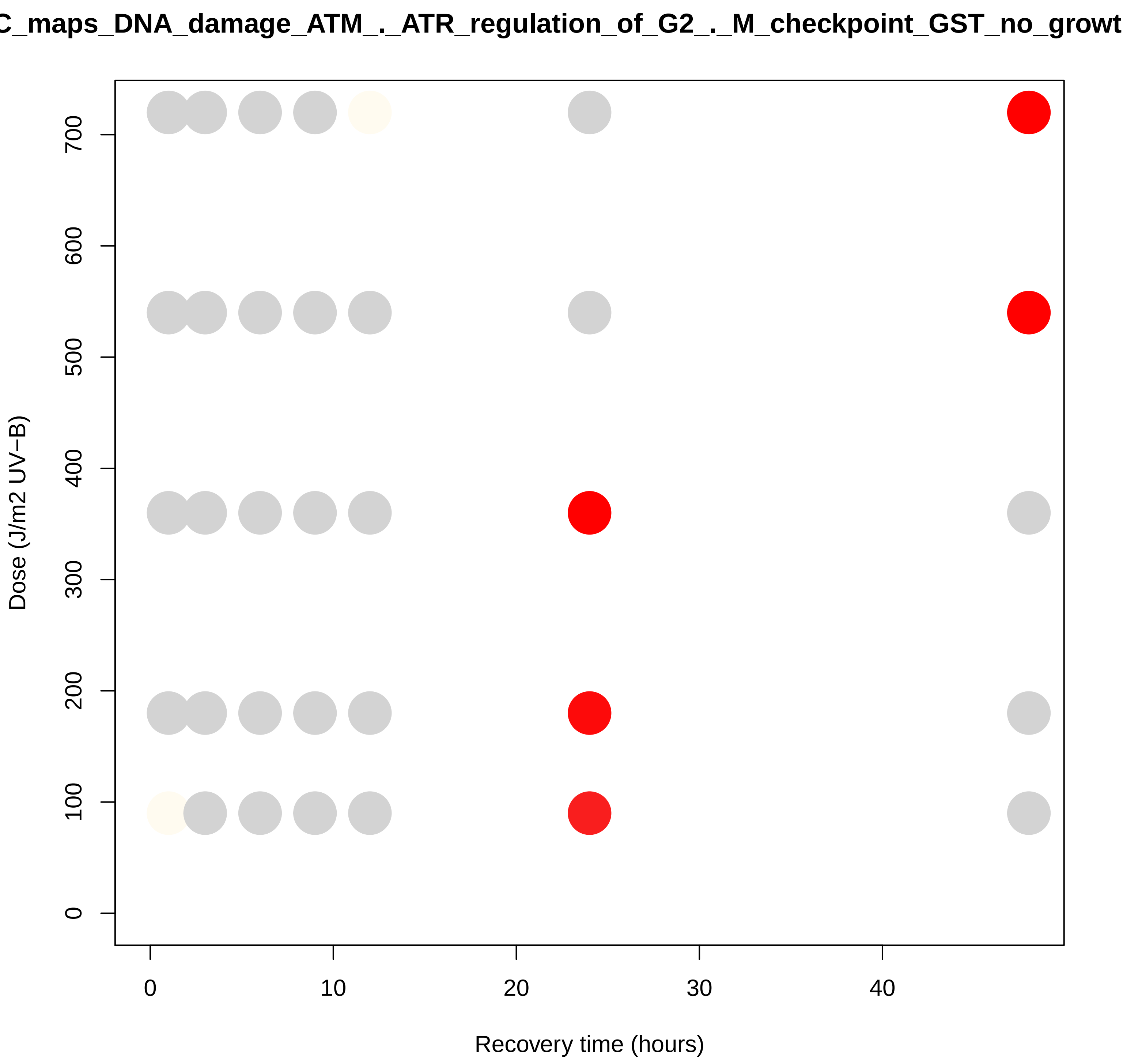

C_maps_DNA_damage_ATM_._ATR_regulation_of_G2_._M_checkpoint_GST_no_growt
Dose (J/m2 UV−B)
0
100
200
300
400
500
600
700
0
10
20
30
40
Recovery time (hours)

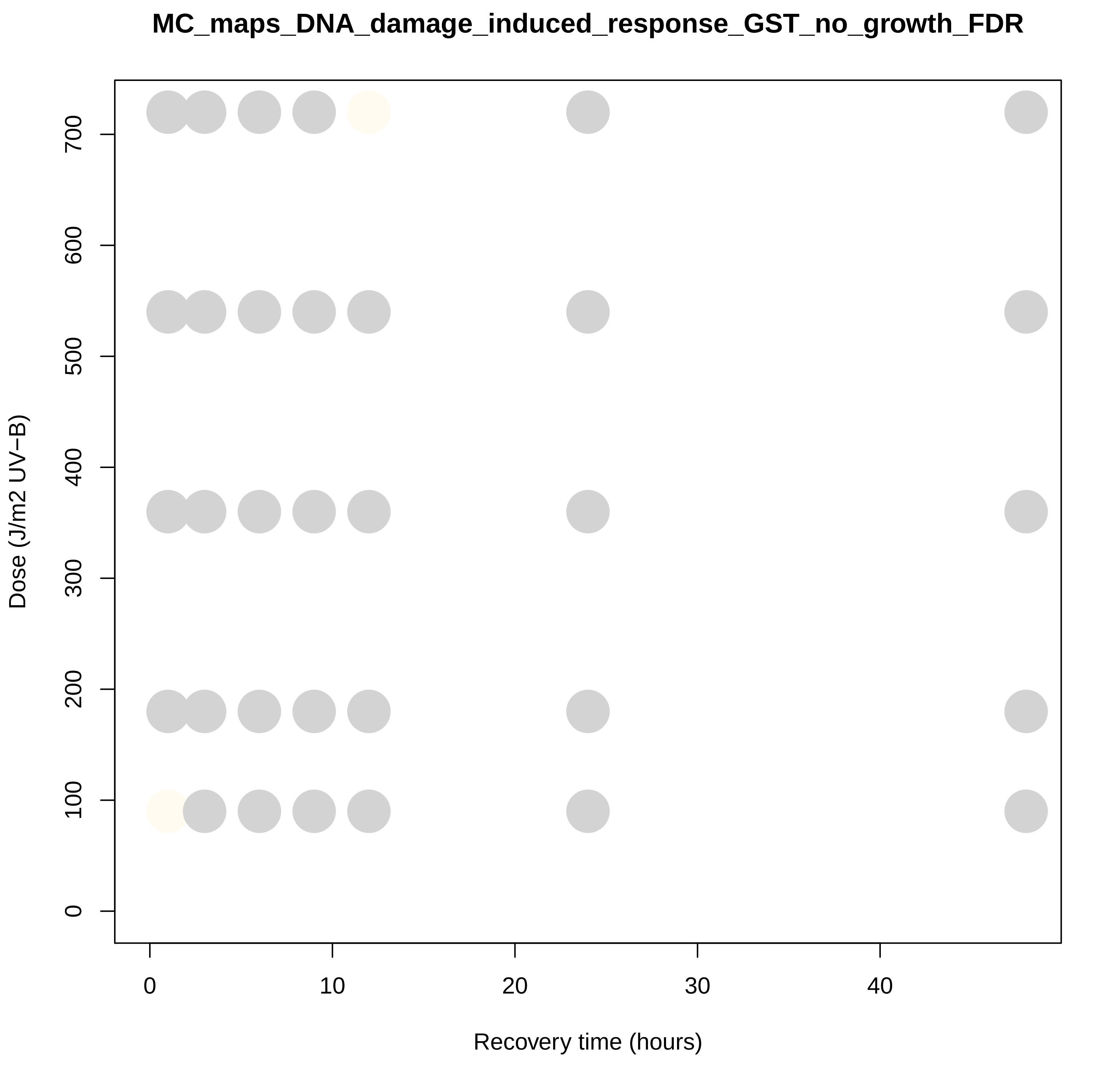
MC_maps_DNA_damage_induced_response_GST_no_growth_FDR
Dose (J/m2 UV−B)
0
100
200
300
400
500
600
700
0
10
20
30
40
Recovery time (hours)

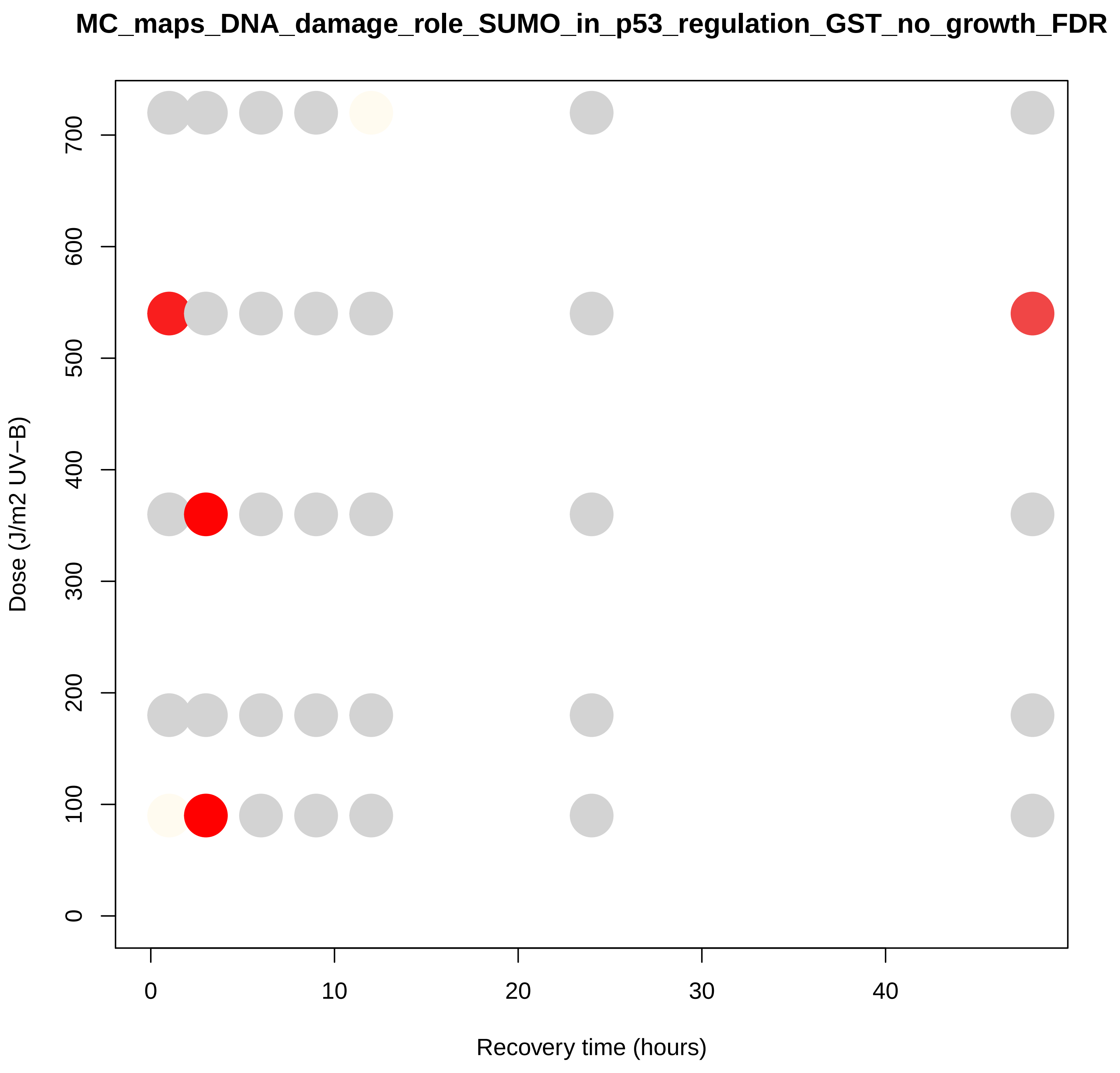

MC_maps_DNA_damage_role_SUMO_in_p53_regulation_GST_no_growth_FDR
Dose (J/m2 UV−B)
0
100
200
300
400
500
600
700
Recovery time (hours)
0
10
20
30
40

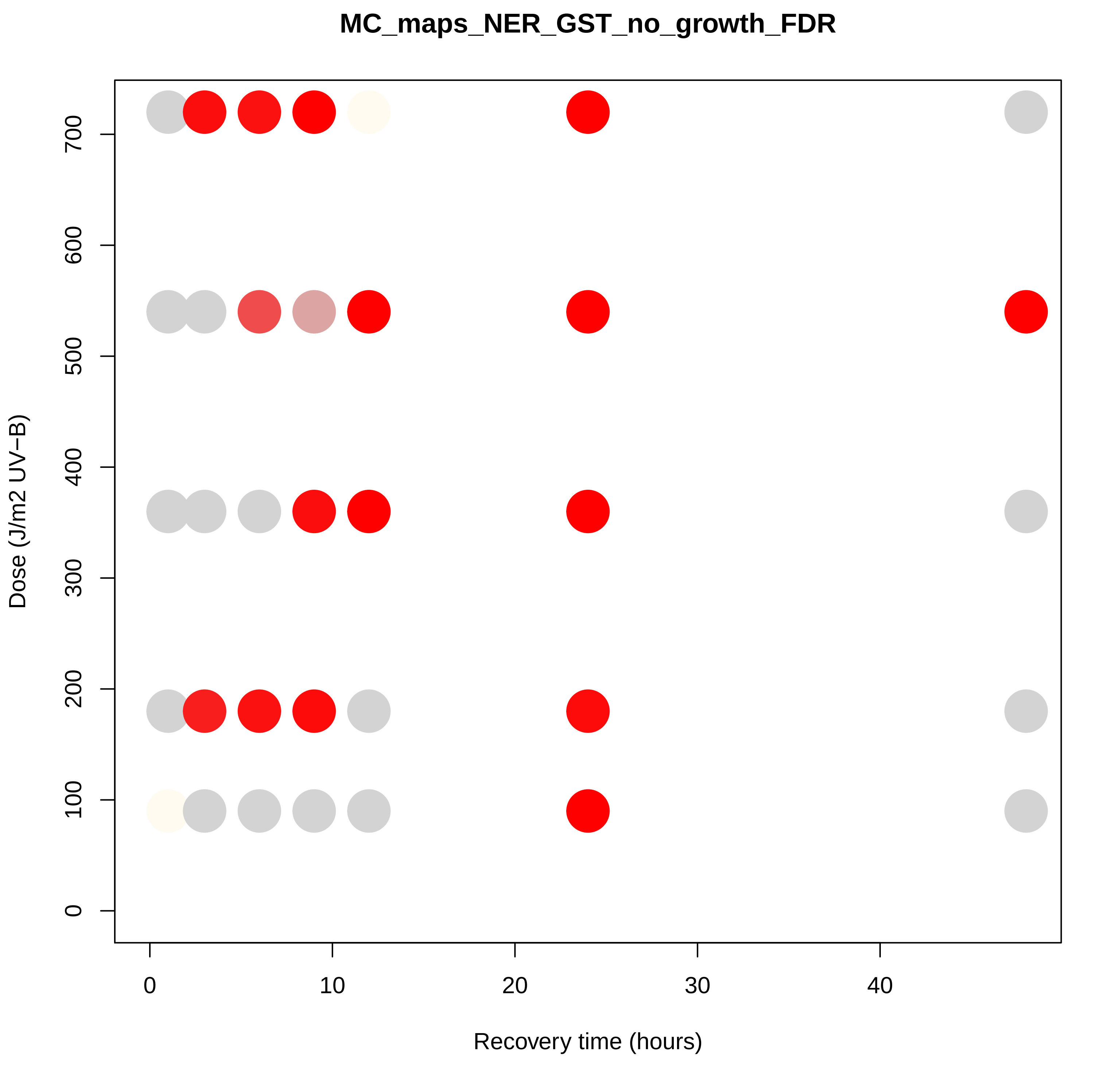
MC_maps_NER_GST_no_growth_FDR
Dose (J/m2 UV−B)
0
100
200
300
400
500
600
700
Recovery time (hours)
0
10
20
30
40

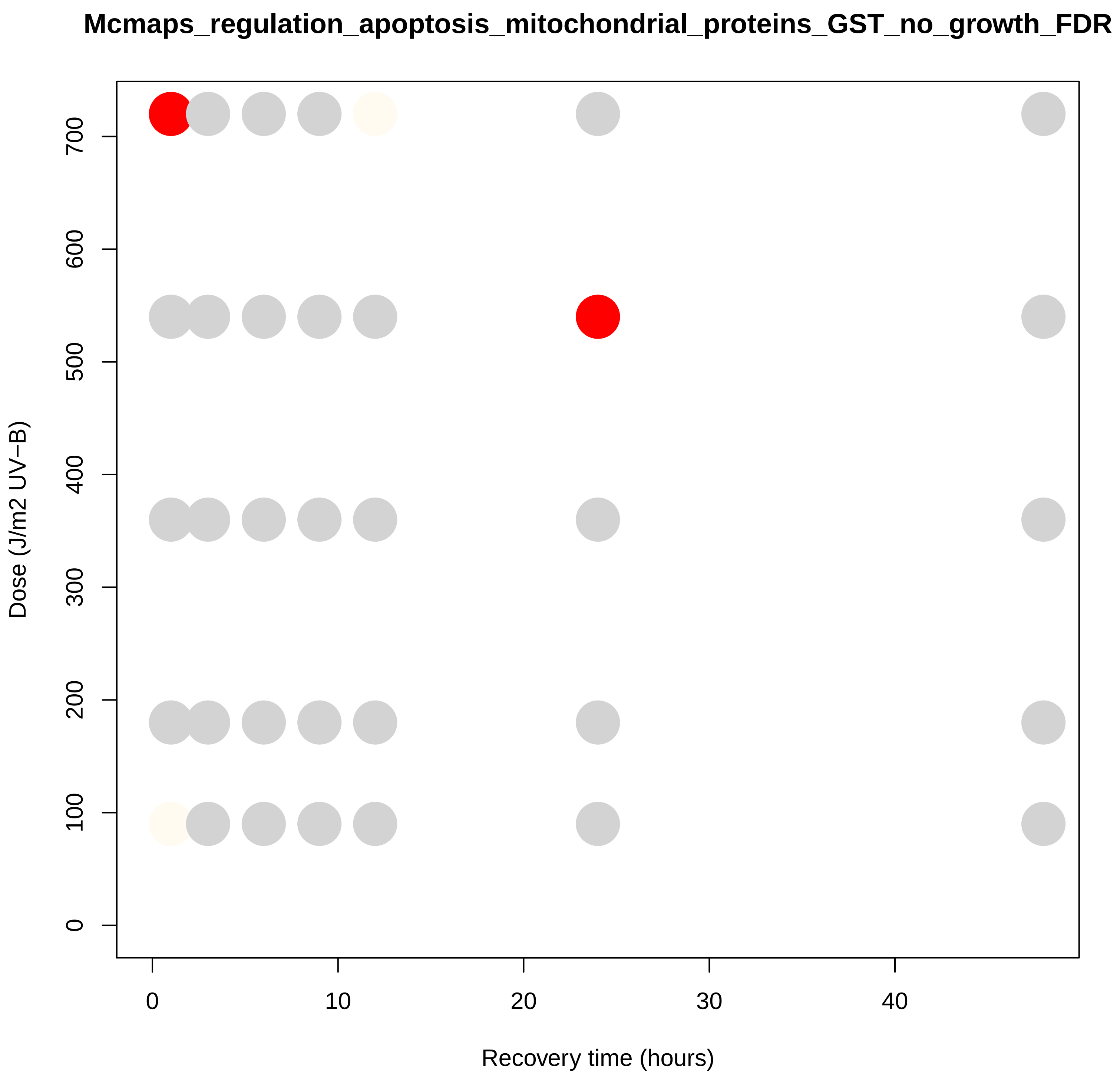

Mcmaps_regulation_apoptosis_mitochondrial_proteins_GST_no_growth_FDR
Dose (J/m2 UV−B)
Recovery time (hours)
0
100
200
300
400
500
600
700
0
10
20
30
40

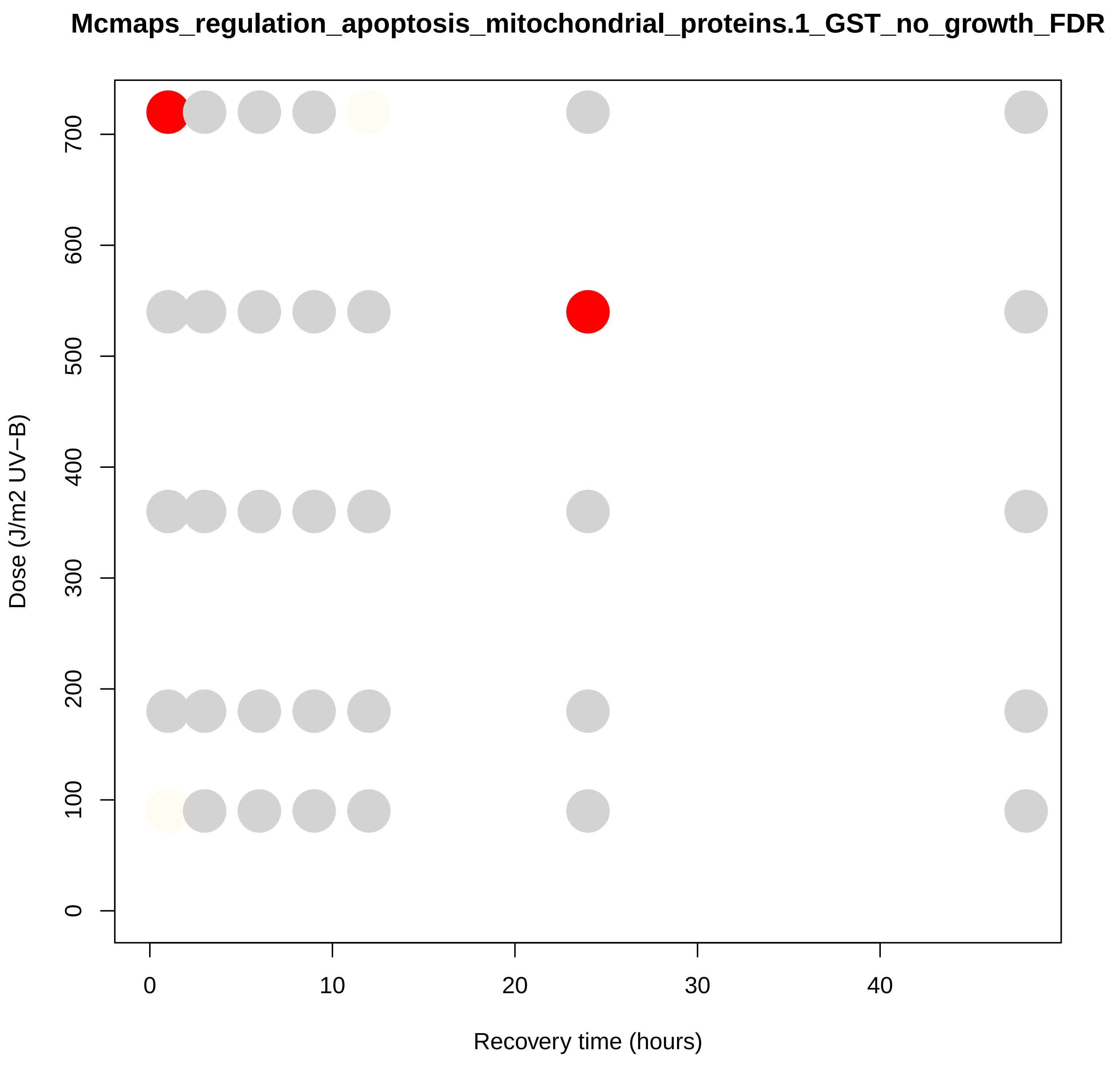

Mcmaps_regulation_apoptosis_mitochondrial_proteins.1_GST_no_growth_FDR
Dose (J/m2 UV−B)
0
100
200
300
400
500
600
700
0
10
20
30
40
Recovery time (hours)

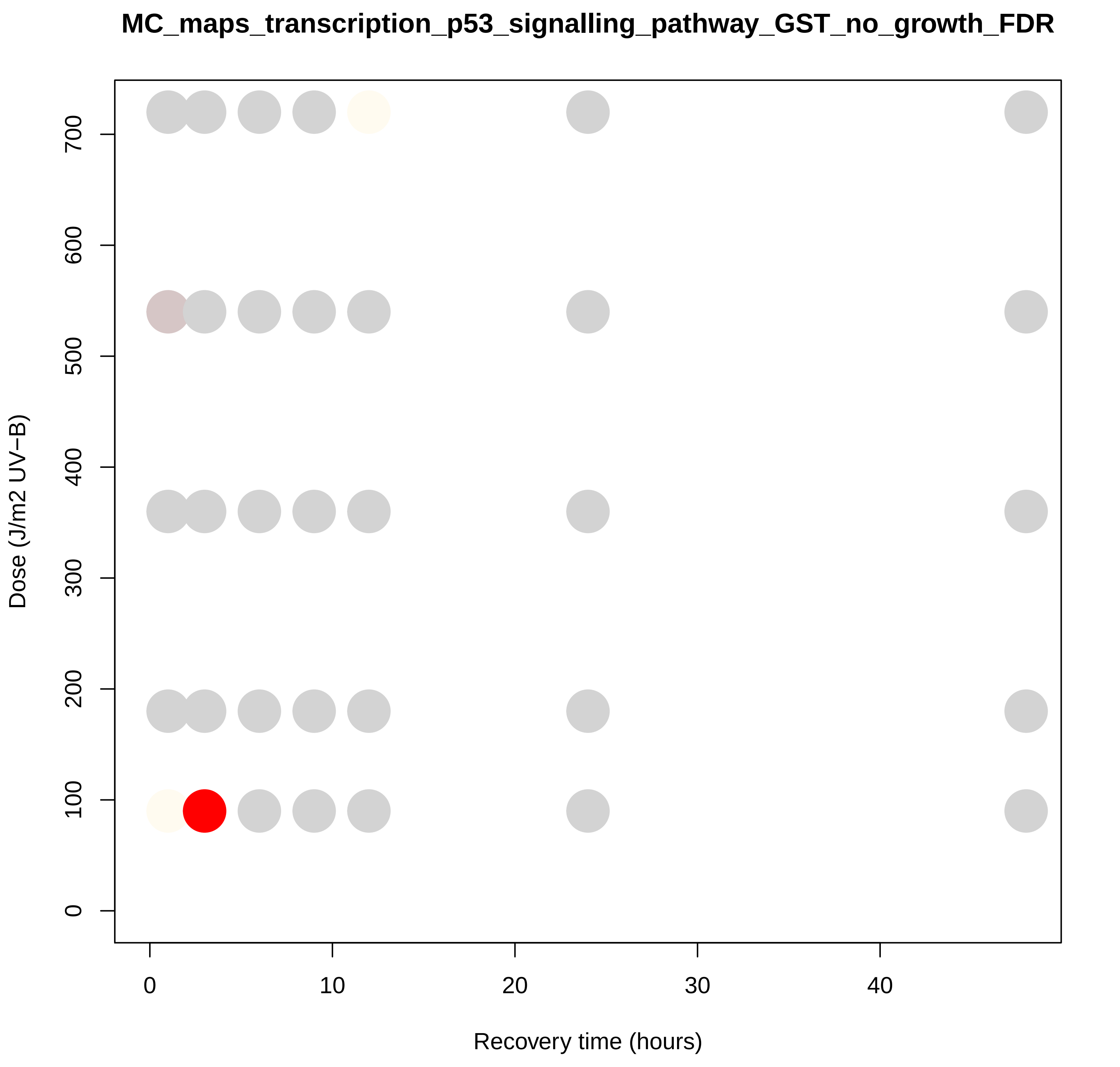

MC_maps_transcription_p53_signalling_pathway_GST_no_growth_FDR
Dose (J/m2 UV−B)
0
100
200
300
400
500
600
700
Recovery time (hours)
0
10
20
30
40

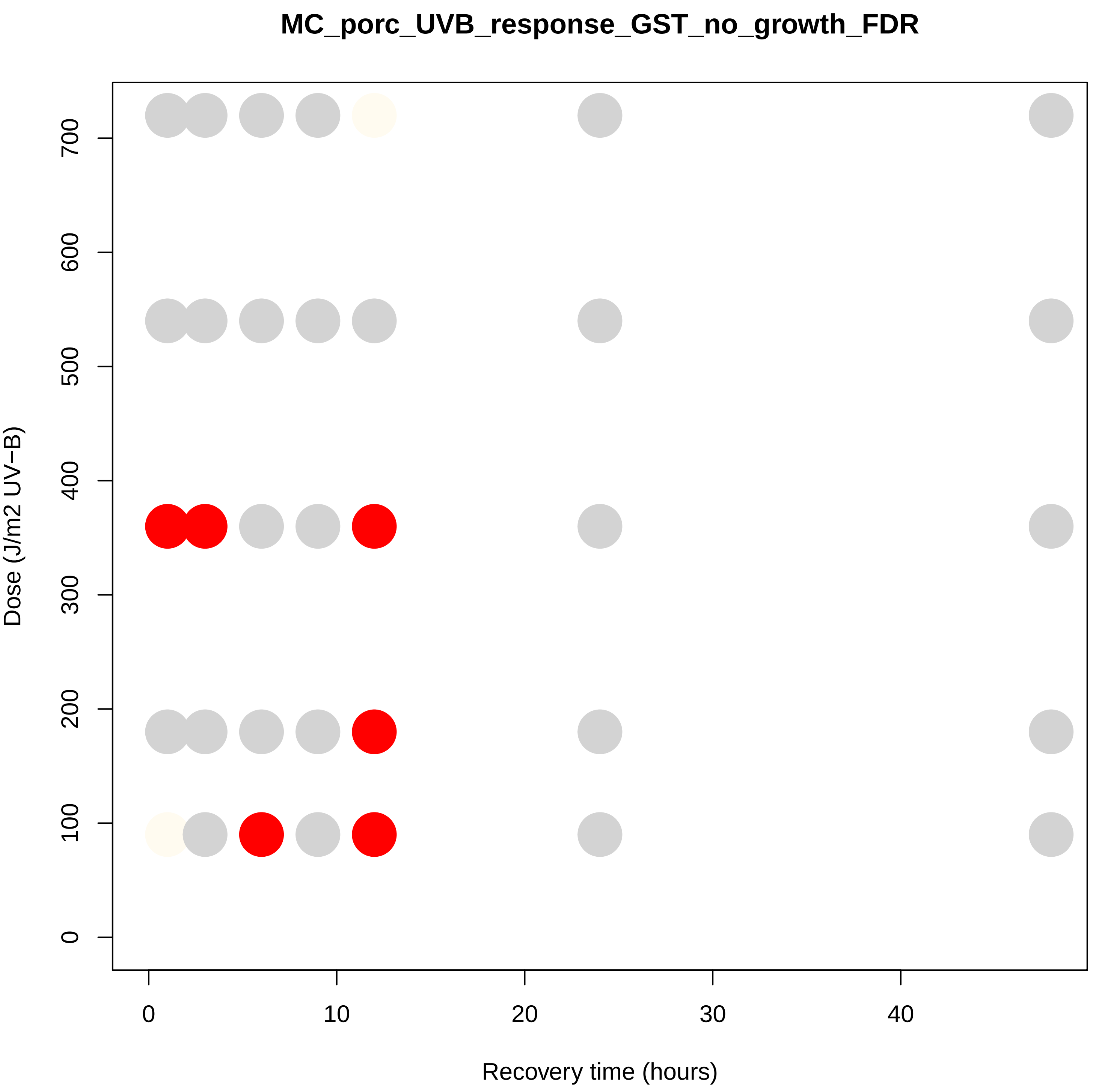
MC_porc_UVB_response_GST_no_growth_FDR
Dose (J/m2 UV−B)
0
100
200
300
400
500
600
700
Recovery time (hours)
0
10
20
30
40

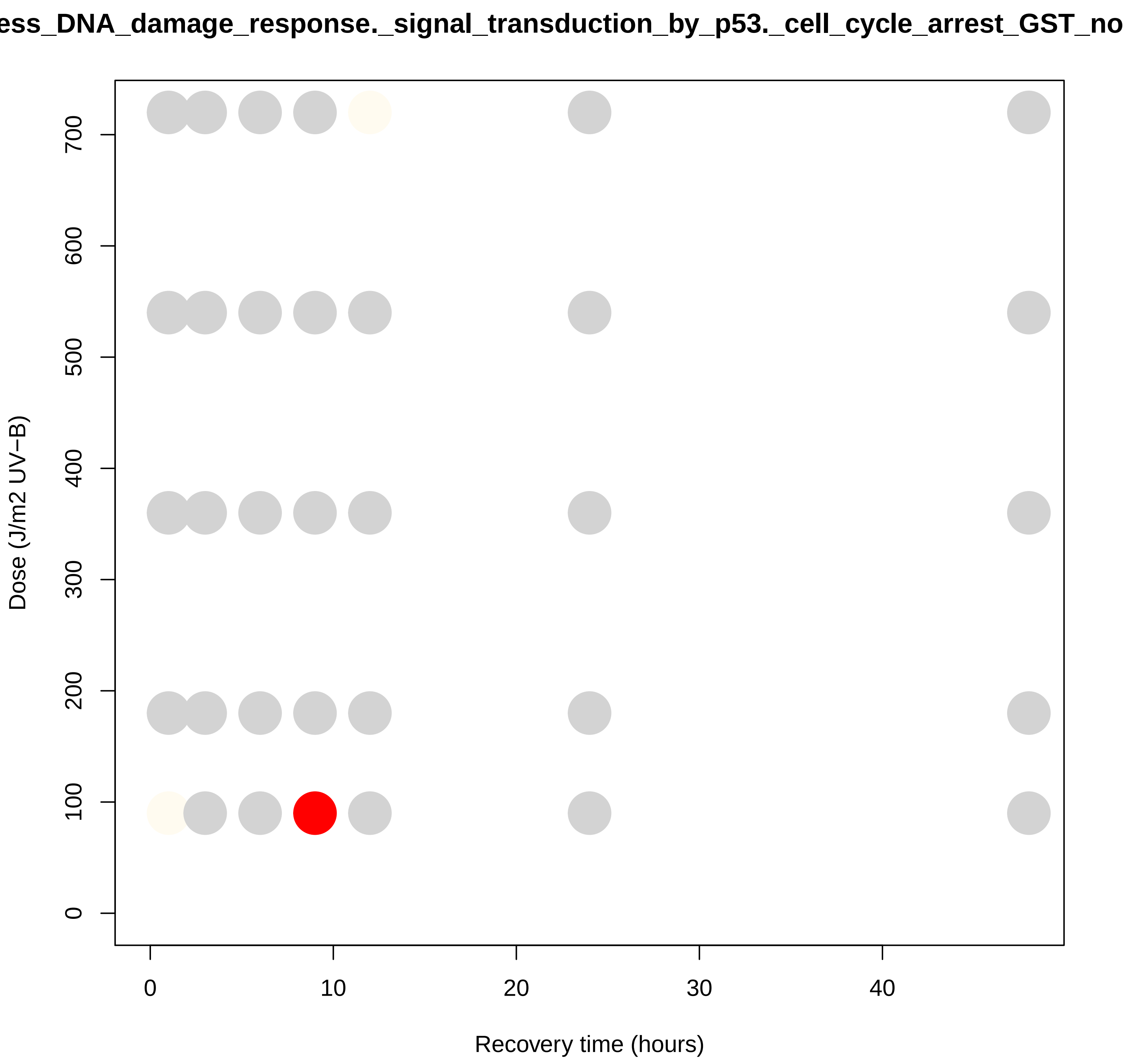

ess_DNA_damage_response._signal_transduction_by_p53._cell_cycle_arrest_GST_no
Dose (J/m2 UV−B)
0
100
200
300
400
500
600
700
0
10
20
30
40
Recovery time (hours)

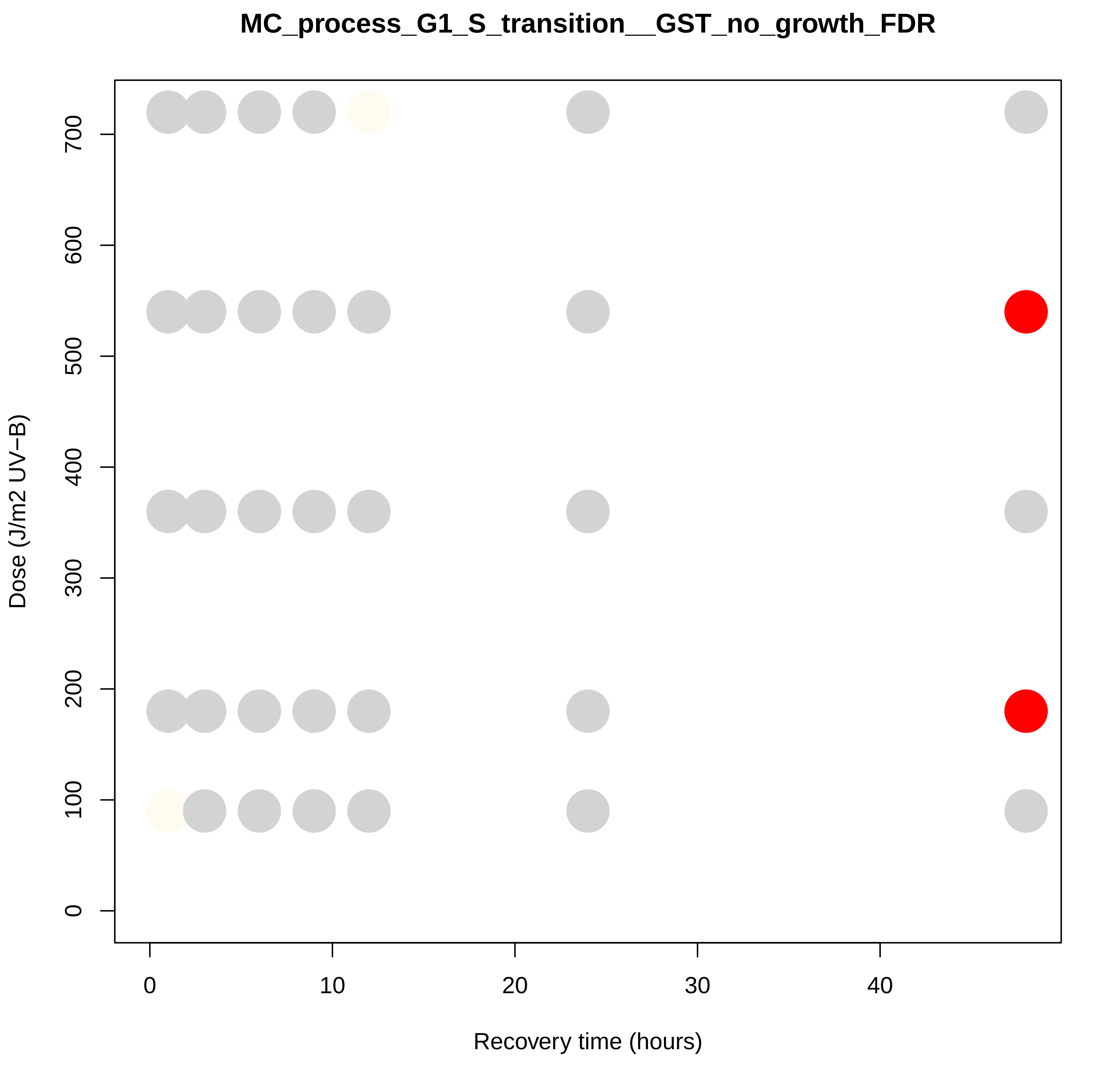
MC_process_G1_S_transition__GST_no_growth_FDR
Dose (J/m2 UV−B)
0
100
200
300
400
500
600
700
Recovery time (hours)
0
10
20
30
40

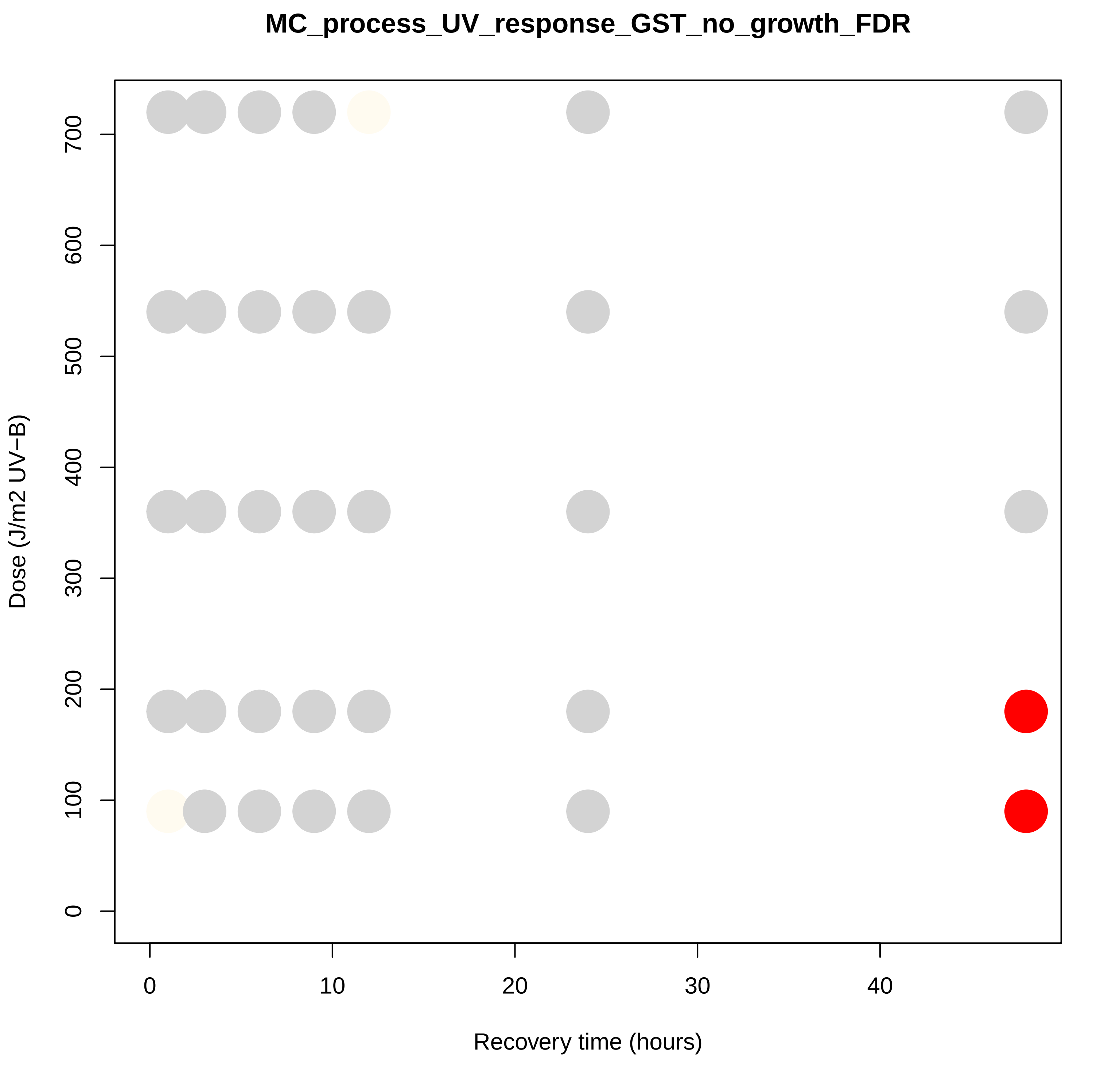
MC_process_UV_response_GST_no_growth_FDR
Dose (J/m2 UV−B)
0
100
200
300
400
500
600
700
0
10
20
30
40
Recovery time (hours)

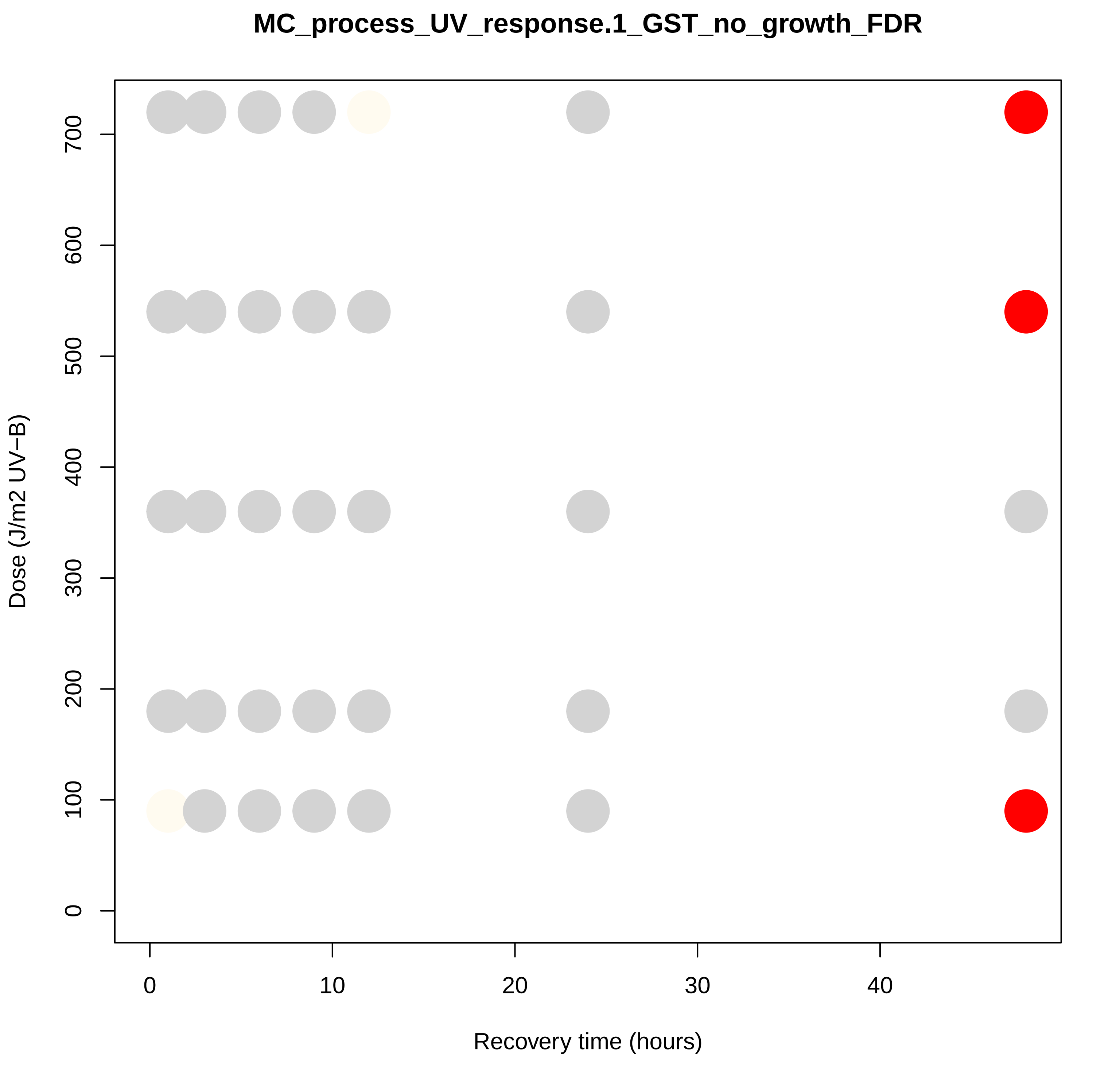
MC_process_UV_response.1_GST_no_growth_FDR
Dose (J/m2 UV−B)
Recovery time (hours)
0
100
200
300
400
500
600
700
0
10
20
30
40

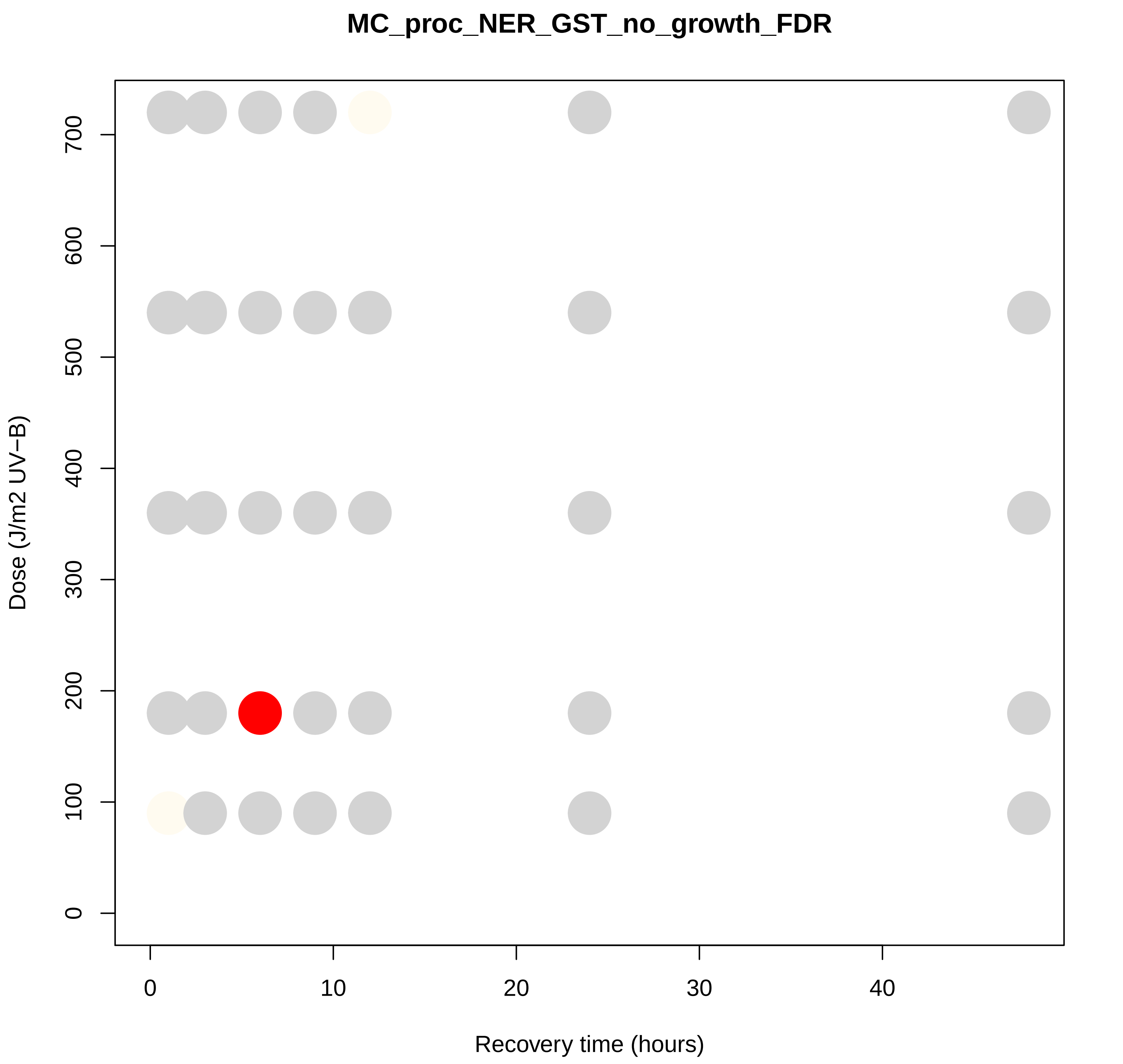

MC_proc_NER_GST_no_growth_FDR
Dose (J/m2 UV−B)
0
100
200
300
400
500
600
700
0
10
20
30
40
Recovery time (hours)

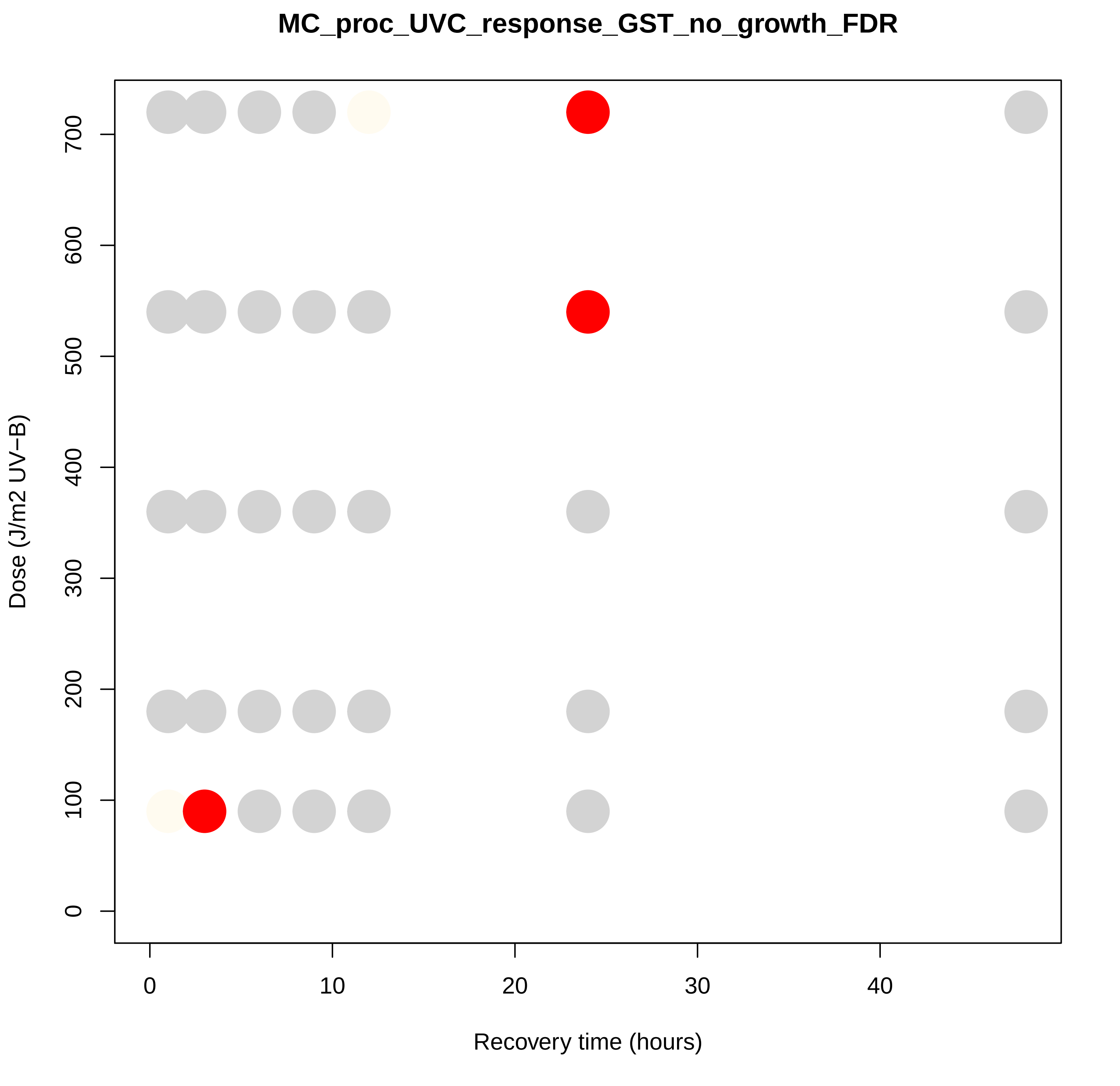
MC_proc_UVC_response_GST_no_growth_FDR
Dose (J/m2 UV−B)
0
100
200
300
400
500
600
700
0
10
20
30
40
Recovery time (hours)

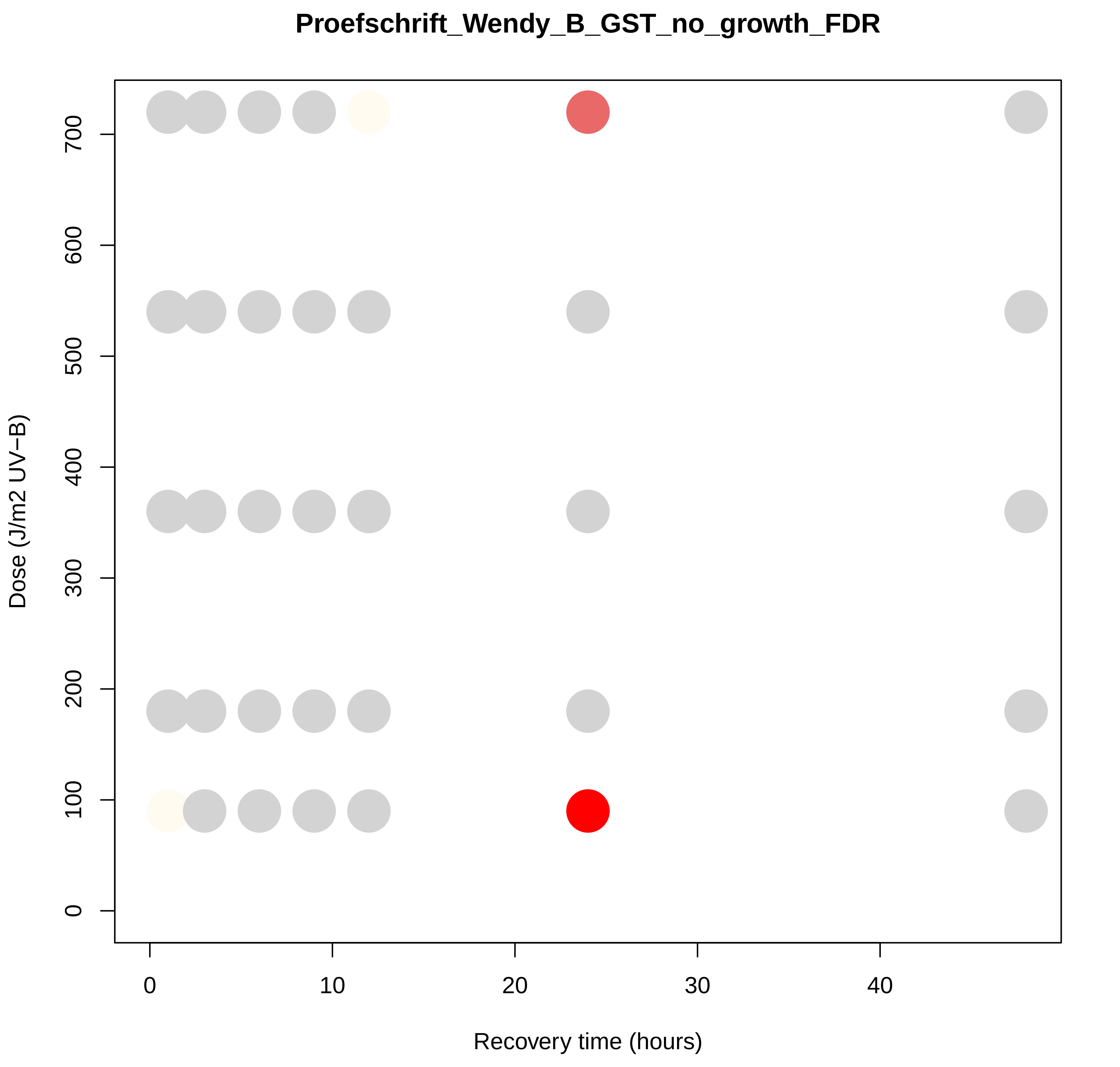
Proefschrift_Wendy_B_GST_no_growth_FDR
Dose (J/m2 UV−B)
0
100
200
300
400
500
600
700
Recovery time (hours)
0
10
20
30
40

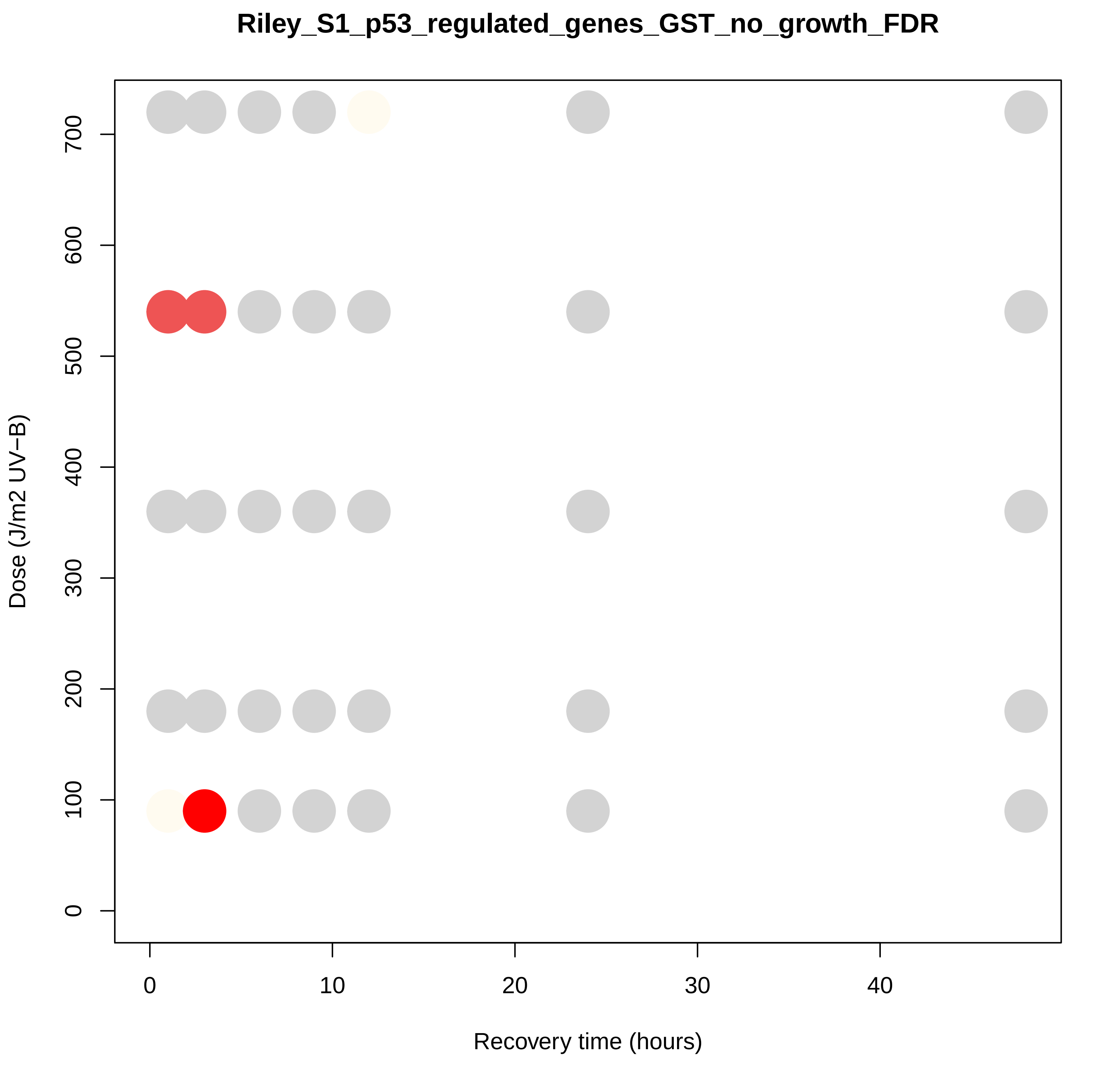
Riley_S1_p53_regulated_genes_GST_no_growth_FDR
Dose (J/m2 UV−B)
0
100
200
300
400
500
600
700
Recovery time (hours)
0
10
20
30
40

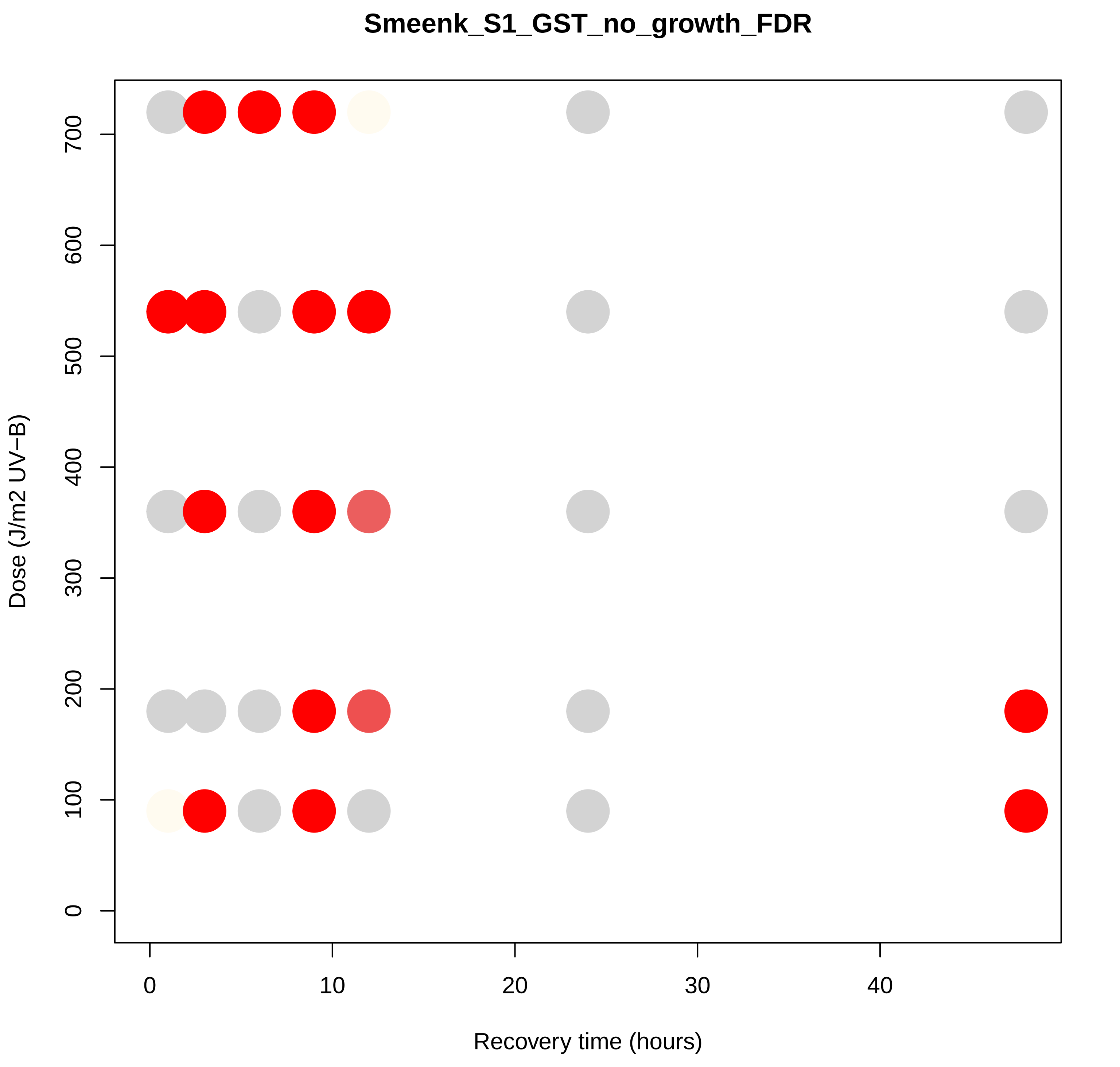
Smeenk_S1_GST_no_growth_FDR
Dose (J/m2 UV−B)
Recovery time (hours)
0
100
200
300
400
500
600
700
0
10
20
30
40

WEI__Apoptosis_Cell_Cycle_GST_no_growth_FDR

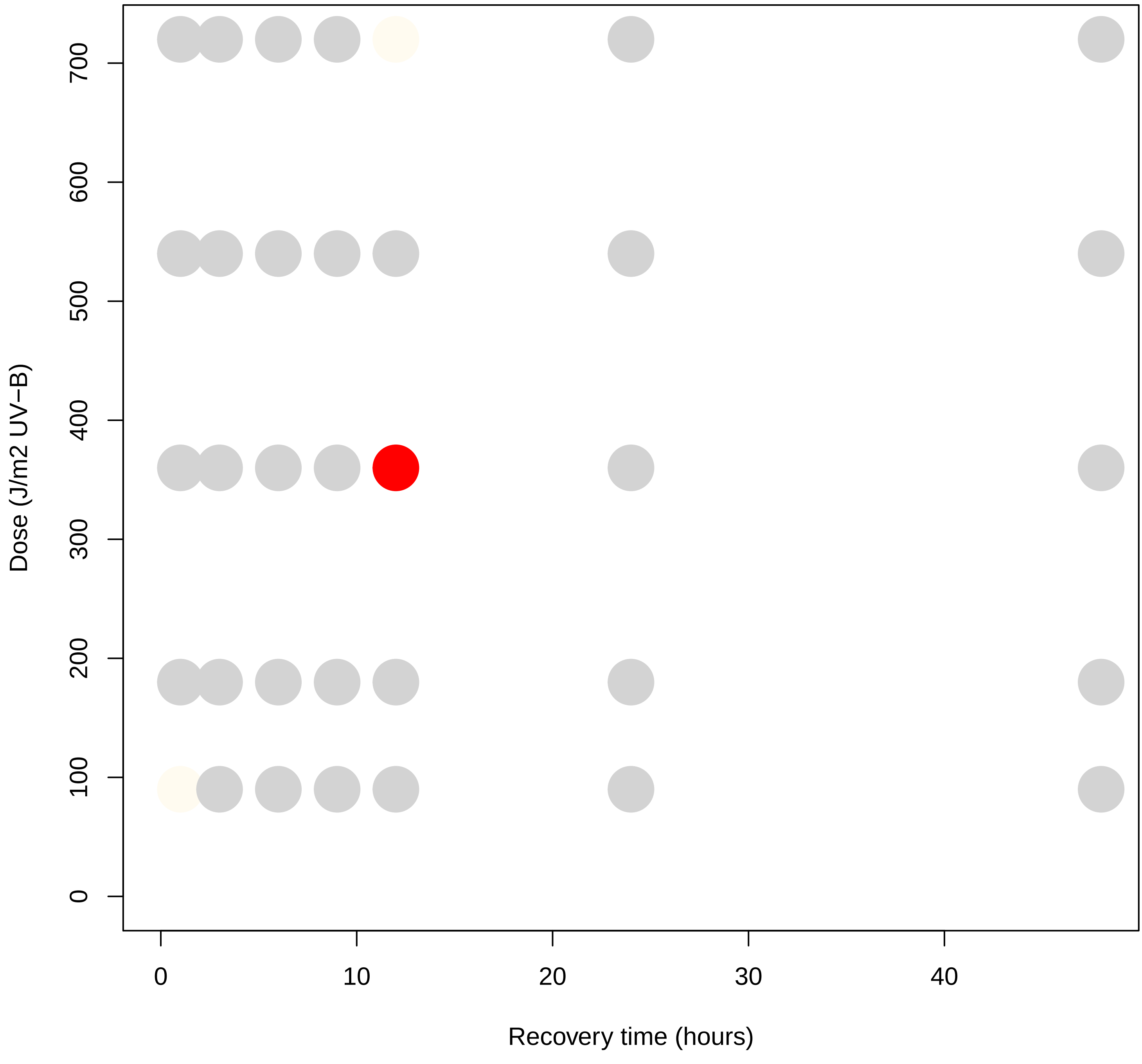

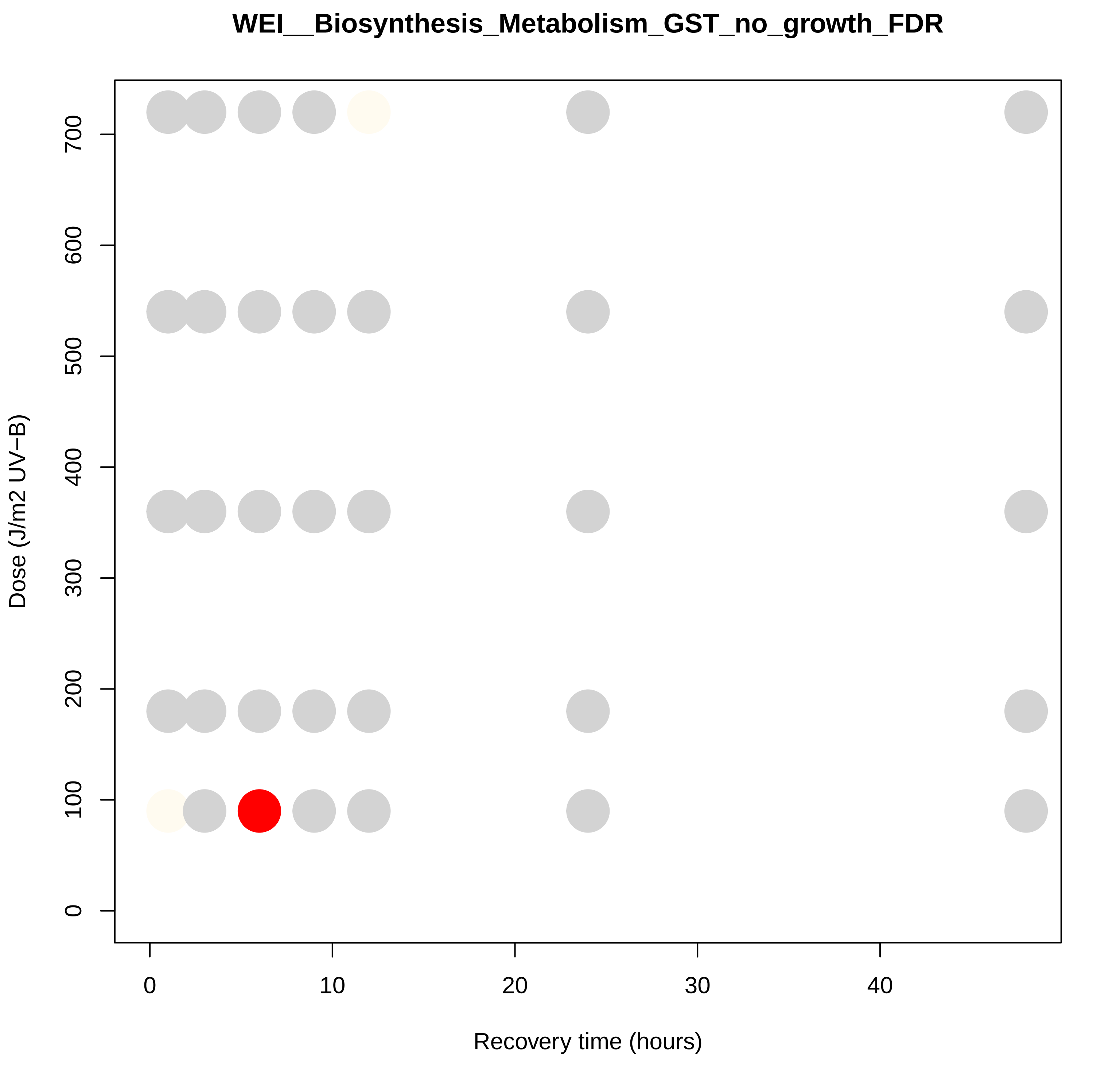

WEI__Biosynthesis_Metabolism_GST_no_growth_FDR
Dose (J/m2 UV−B)
Recovery time (hours)
0
100
200
300
400
500
600
700
0
10
20
30
40

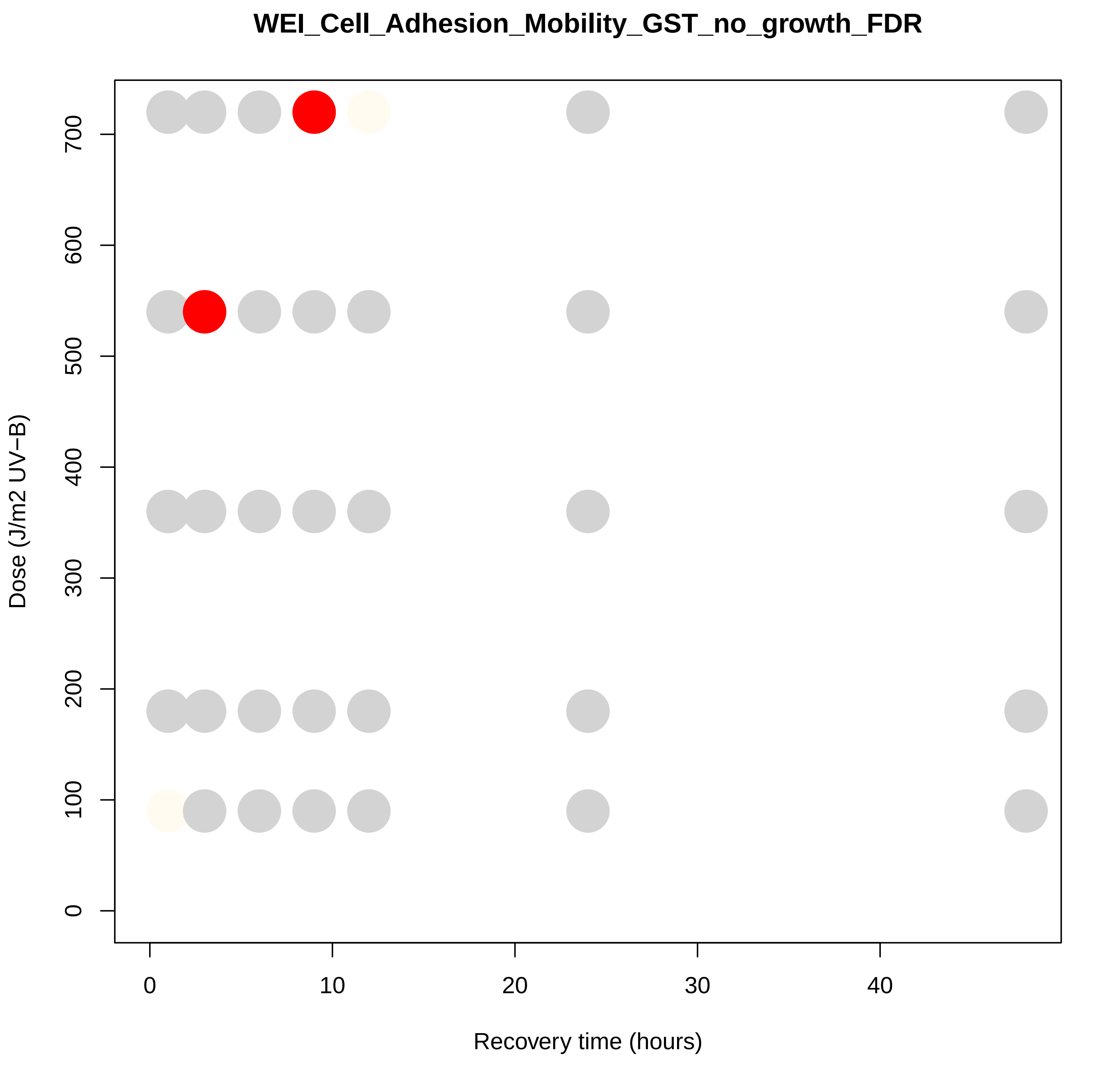
WEI_Cell_Adhesion_Mobility_GST_no_growth_FDR
Dose (J/m2 UV−B)
0
100
200
300
400
500
600
700
0
10
20
30
40
Recovery time (hours)

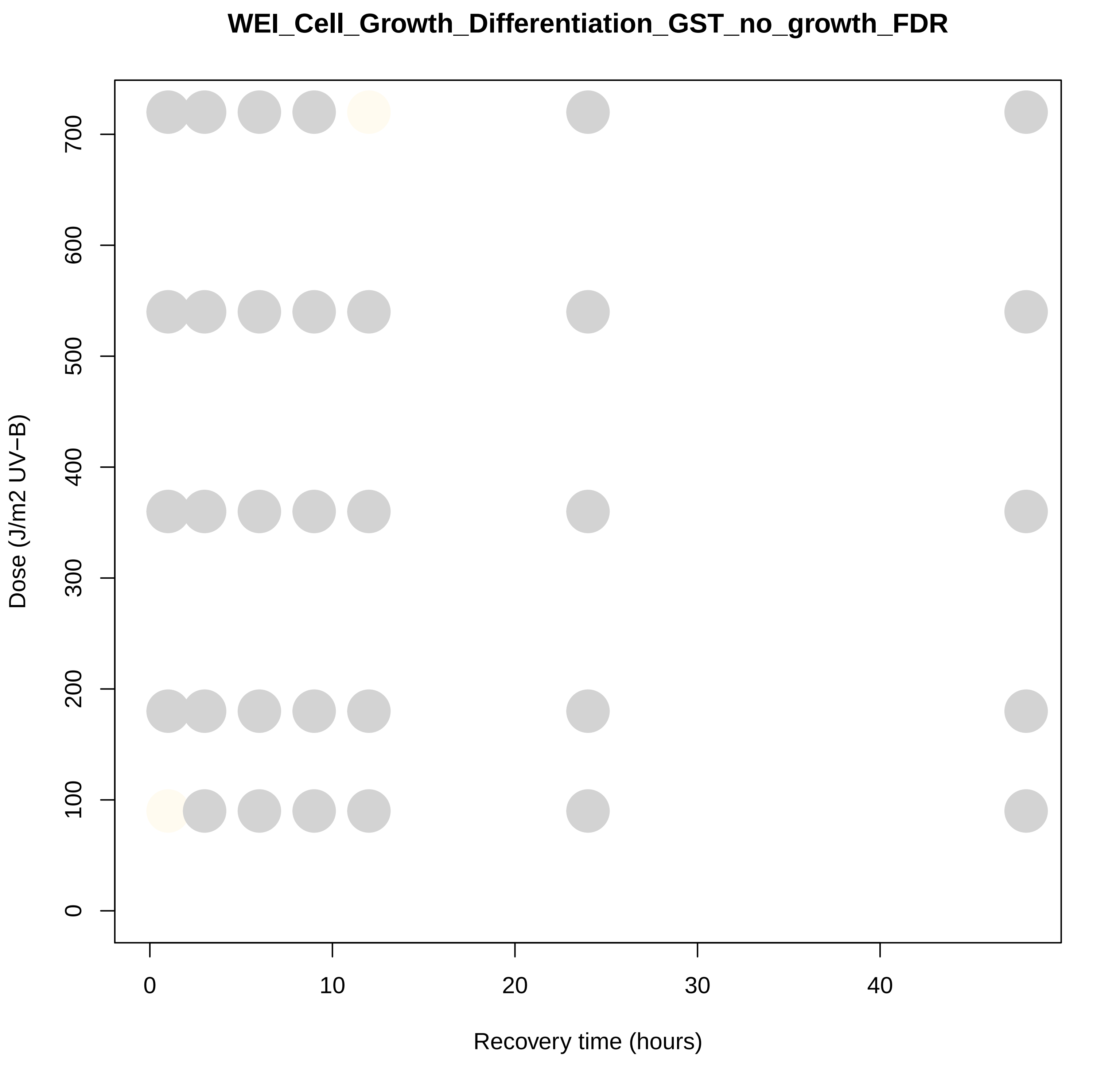
WEI_Cell_Growth_Differentiation_GST_no_growth_FDR
Dose (J/m2 UV−B)
0
100
200
300
400
500
600
700
0
10
20
30
40
Recovery time (hours)

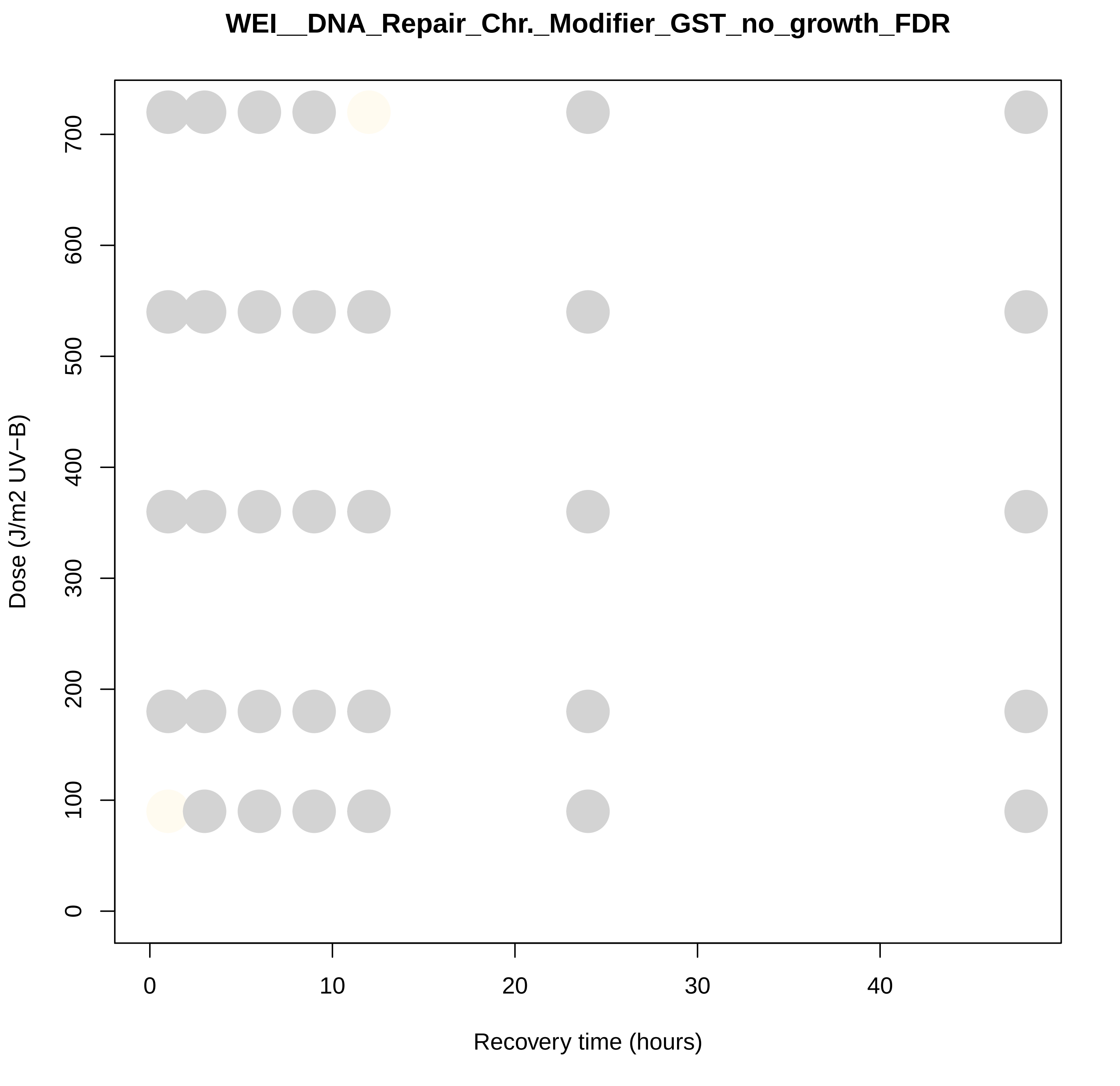
WEI__DNA_Repair_Chr._Modifier_GST_no_growth_FDR
Dose (J/m2 UV−B)
Recovery time (hours)
0
100
200
300
400
500
600
700
0
10
20
30
40

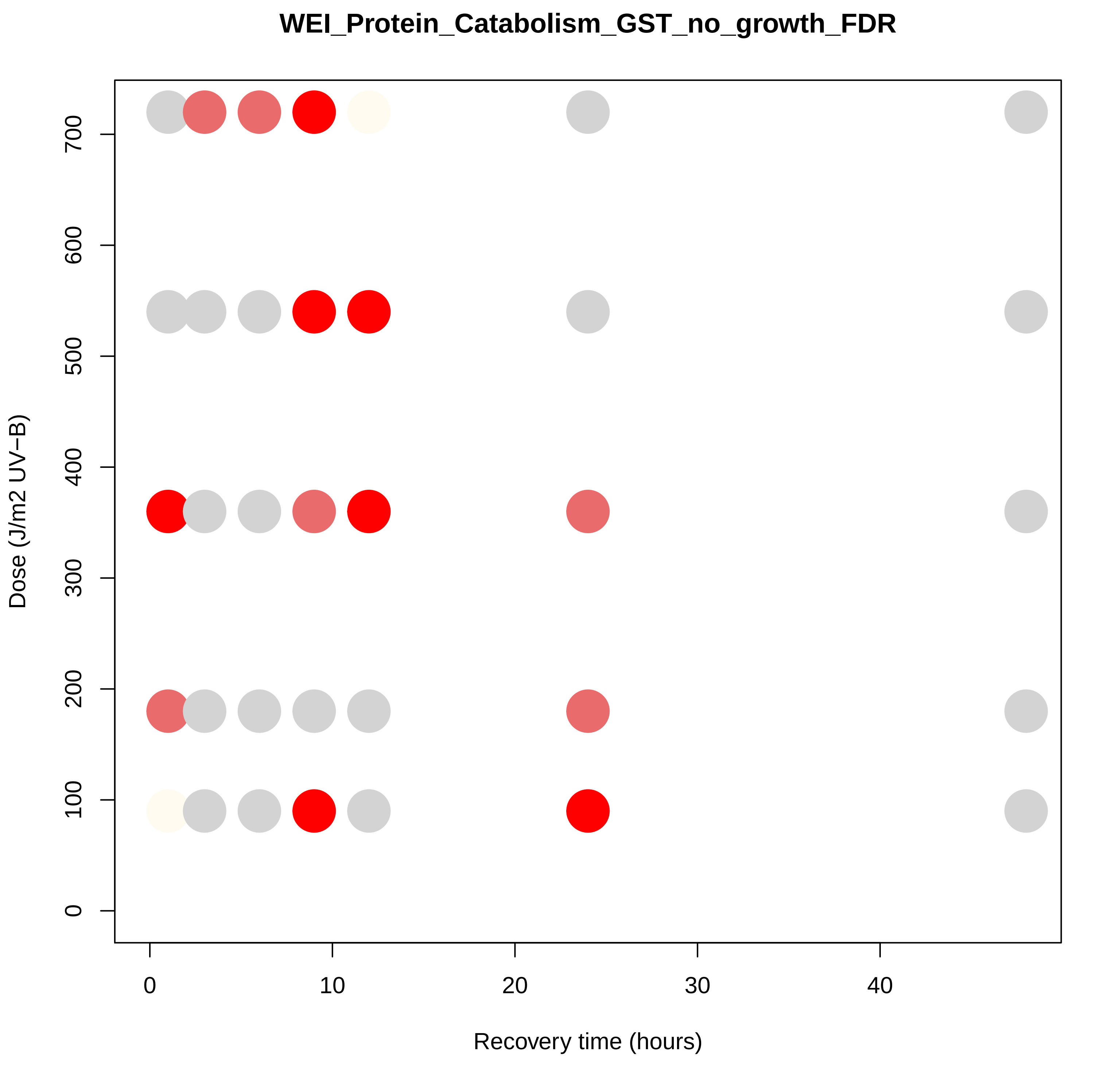

WEI_Protein_Catabolism_GST_no_growth_FDR
Dose (J/m2 UV−B)
0
100
200
300
400
500
600
700
0
10
20
30
40
Recovery time (hours)

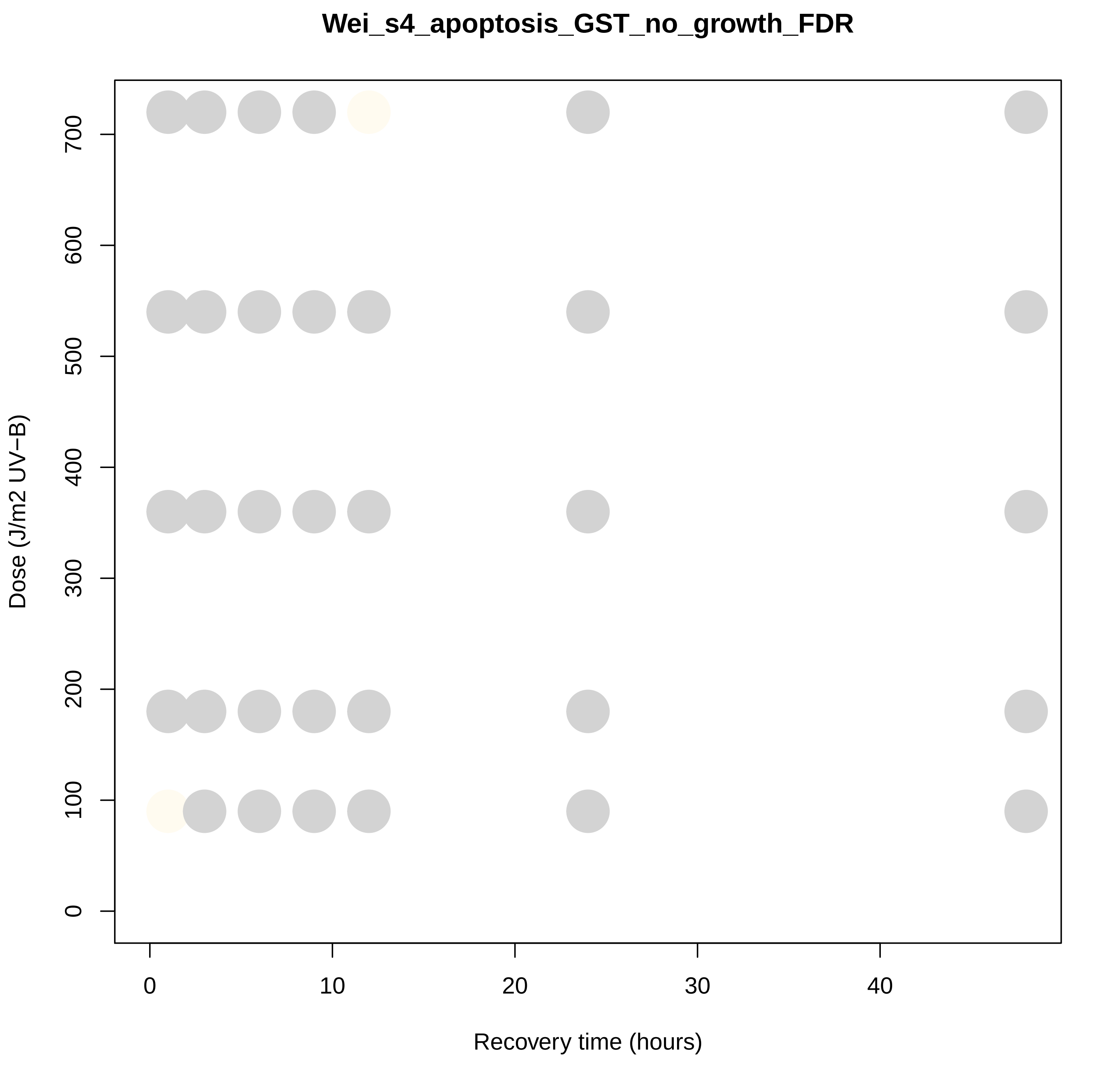

Wei_s4_apoptosis_GST_no_growth_FDR
Dose (J/m2 UV−B)
Recovery time (hours)
0
100
200
300
400
500
600
700
0
10
20
30
40

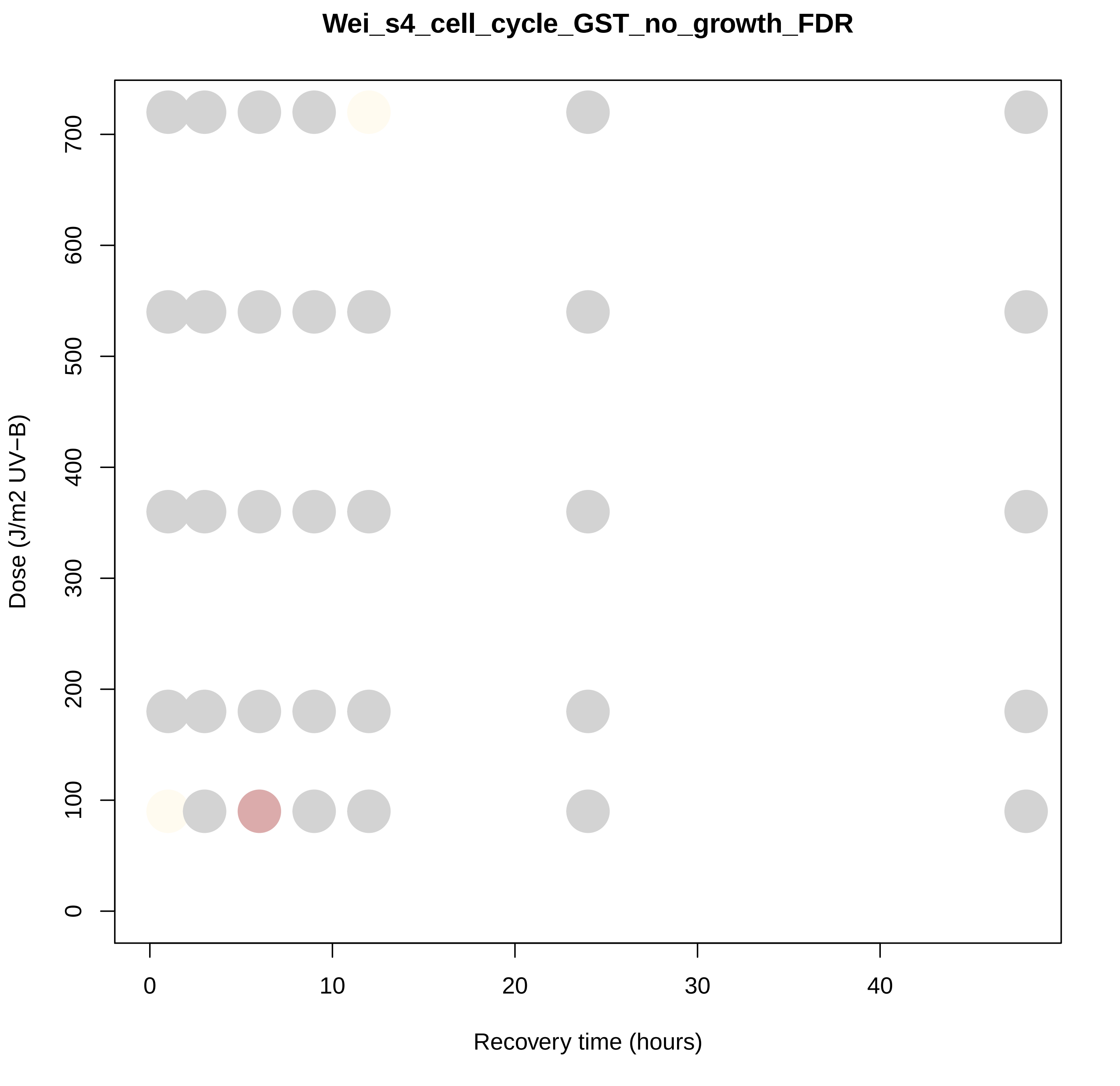

Wei_s4_cell_cycle_GST_no_growth_FDR
Dose (J/m2 UV−B)
0
100
200
300
400
500
600
700
0
10
20
30
40
Recovery time (hours)

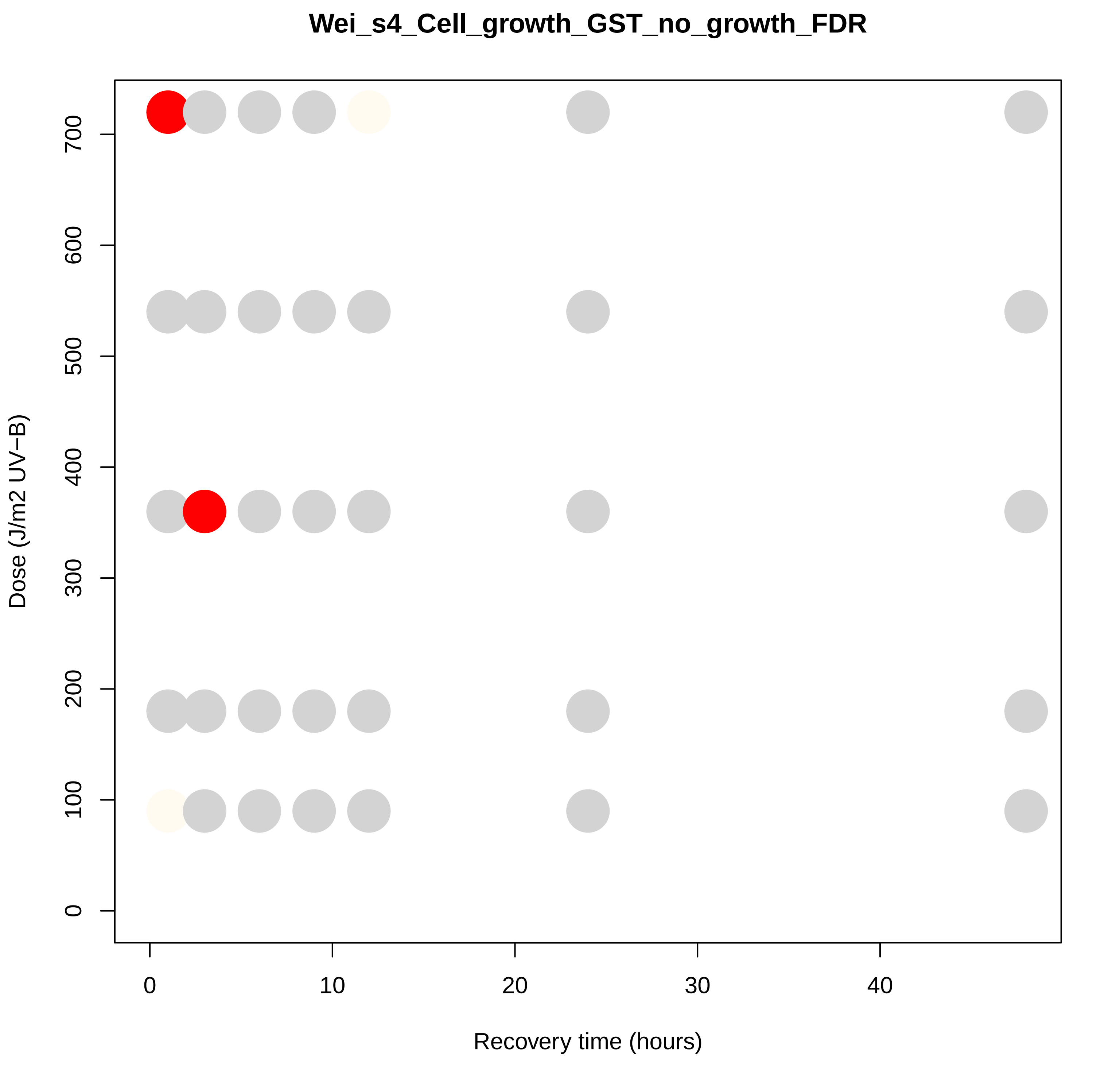
Wei_s4_Cell_growth_GST_no_growth_FDR
Dose (J/m2 UV−B)
0
100
200
300
400
500
600
700
Recovery time (hours)
0
10
20
30
40

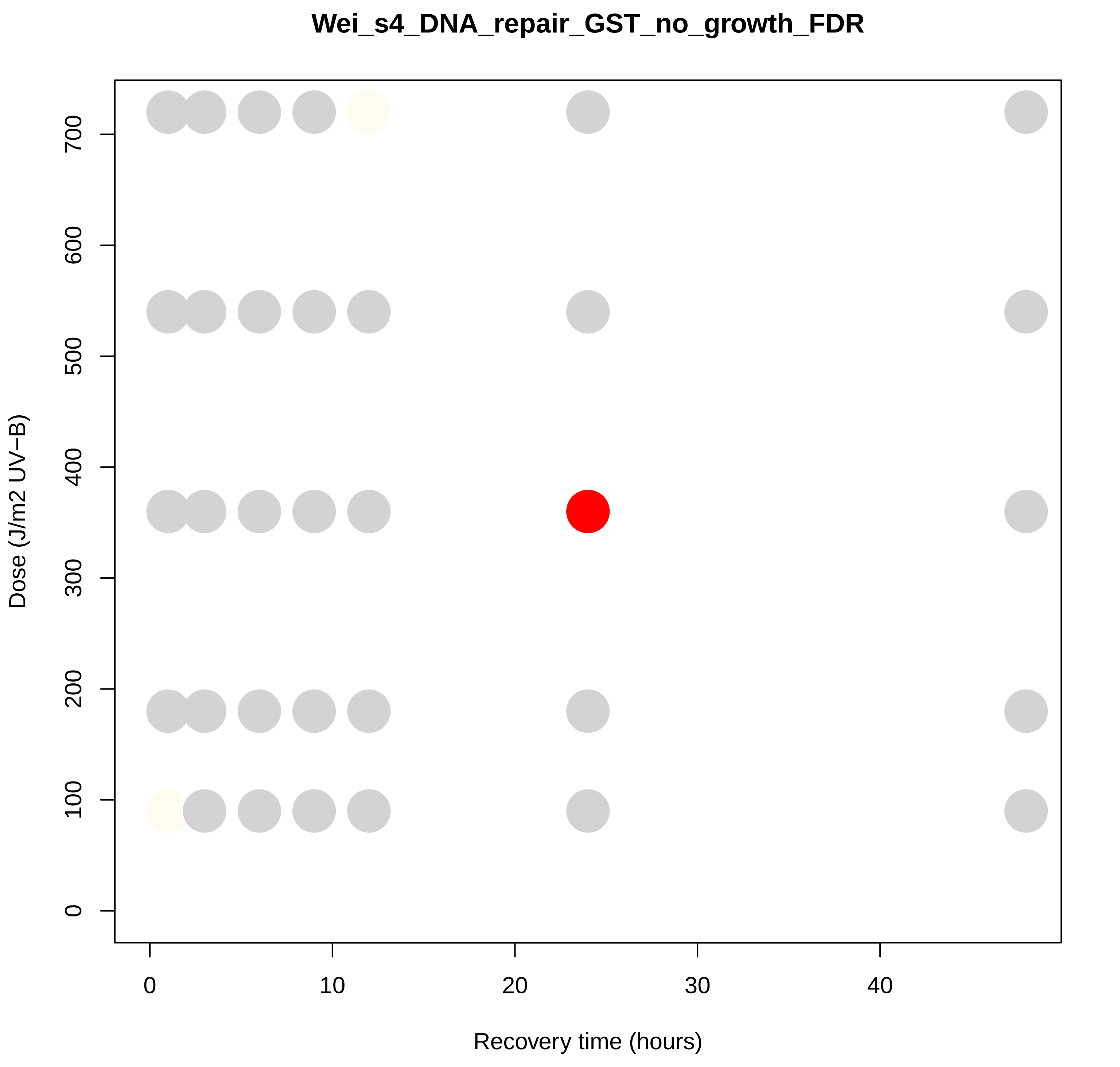
Wei_s4_DNA_repair_GST_no_growth_FDR
Dose (J/m2 UV−B)
Recovery time (hours)
0
100
200
300
400
500
600
700
0
10
20
30
40

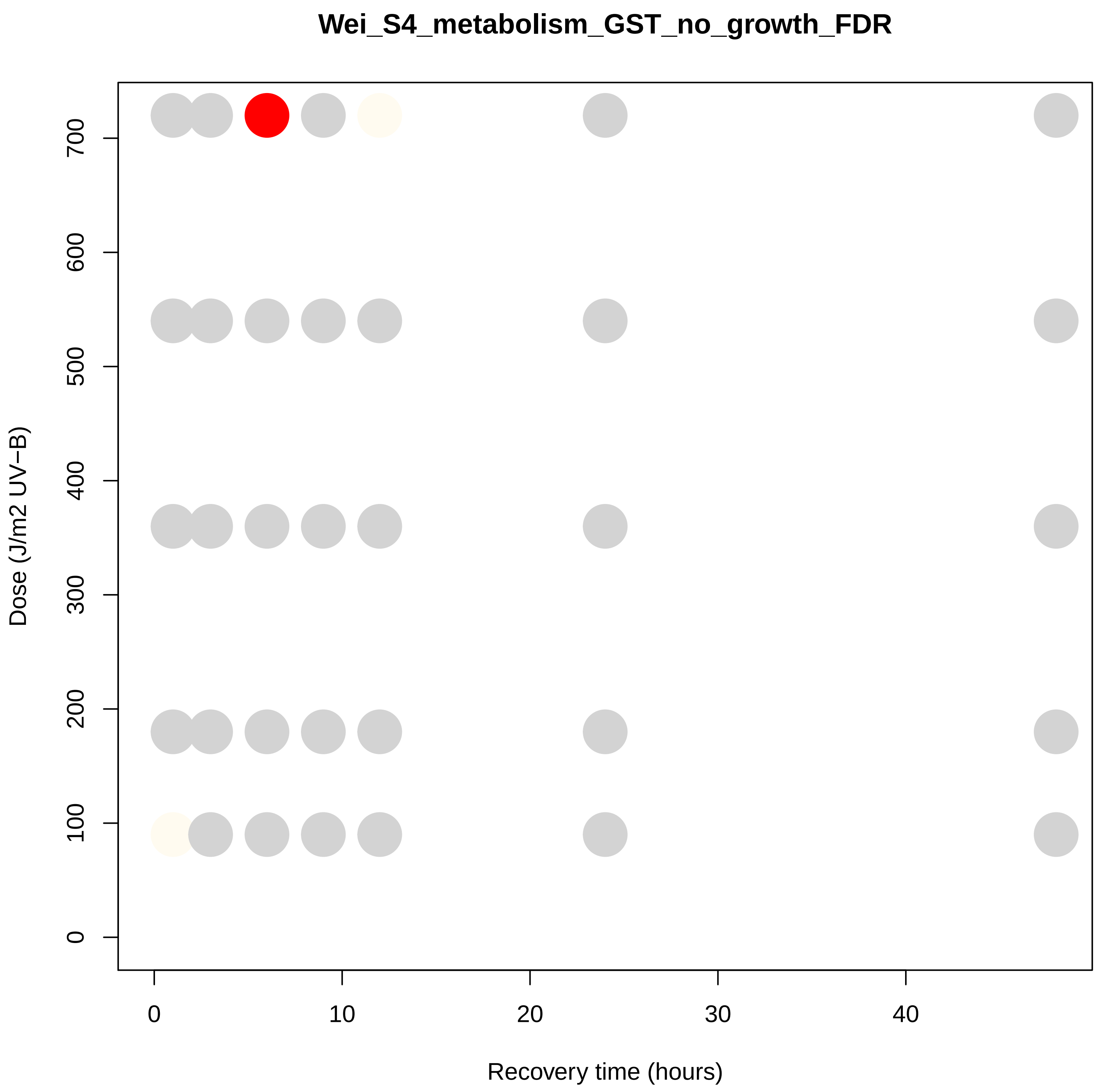
Wei_S4_metabolism_GST_no_growth_FDR
Dose (J/m2 UV−B)
0
100
200
300
400
500
600
700
Recovery time (hours)
0
10
20
30
40

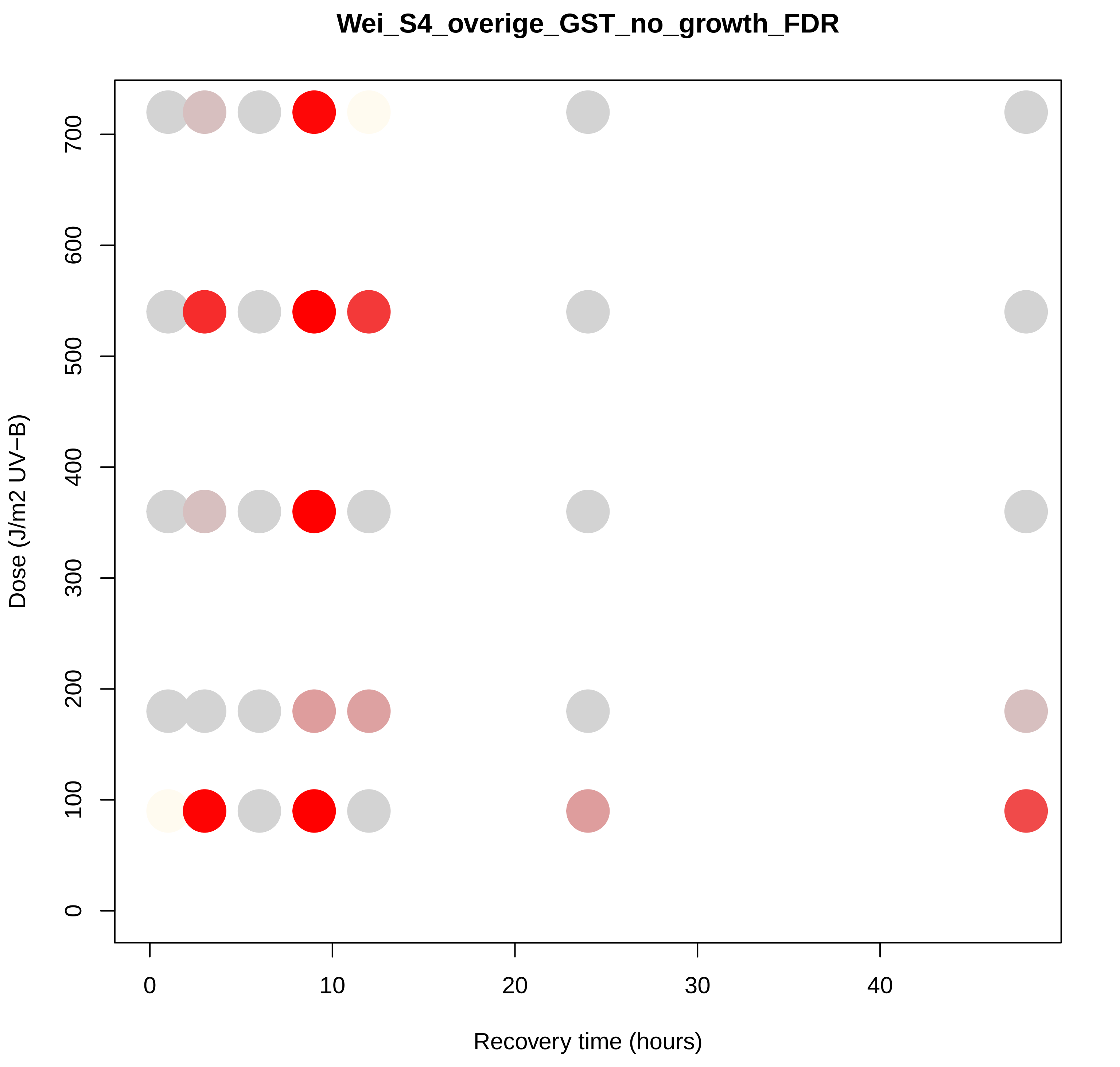
Wei_S4_overige_GST_no_growth_FDR
Dose (J/m2 UV−B)
0
100
200
300
400
500
600
700
Recovery time (hours)
0
10
20
30
40

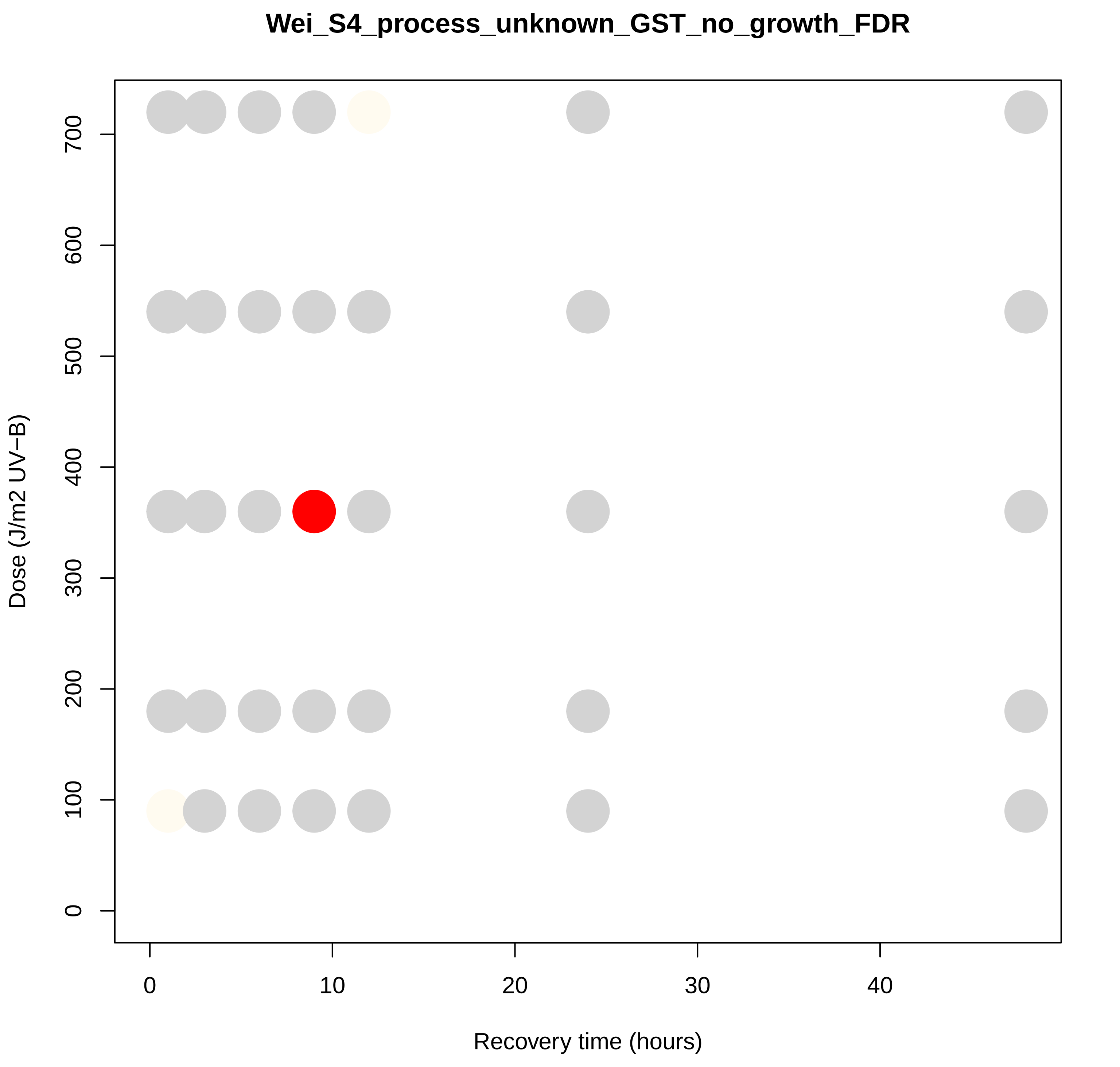

Wei_S4_process_unknown_GST_no_growth_FDR
Dose (J/m2 UV−B)
0
100
200
300
400
500
600
700
0
10
20
30
40
Recovery time (hours)

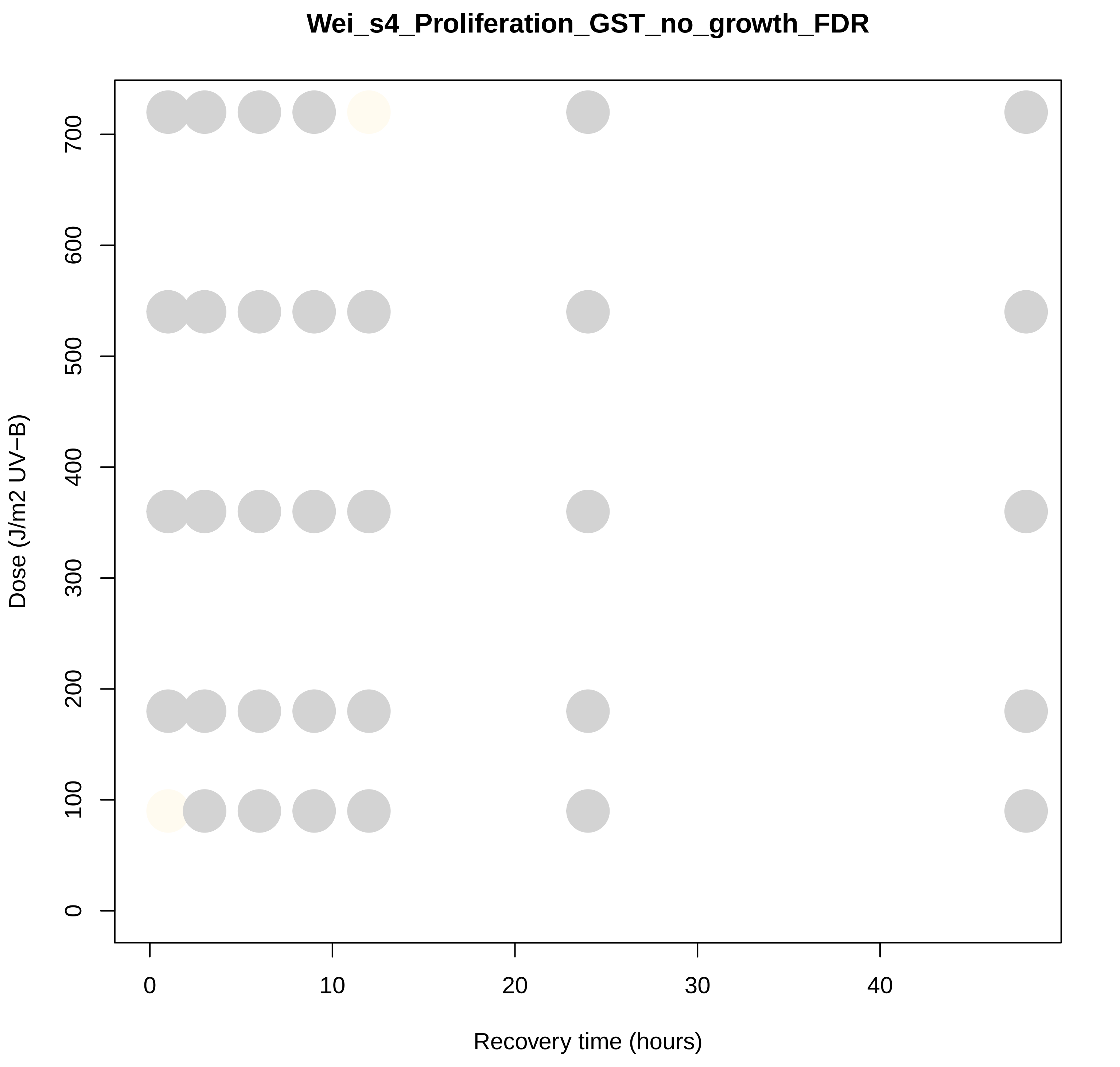
Wei_s4_Proliferation_GST_no_growth_FDR
Dose (J/m2 UV−B)
0
100
200
300
400
500
600
700
Recovery time (hours)
0
10
20
30
40

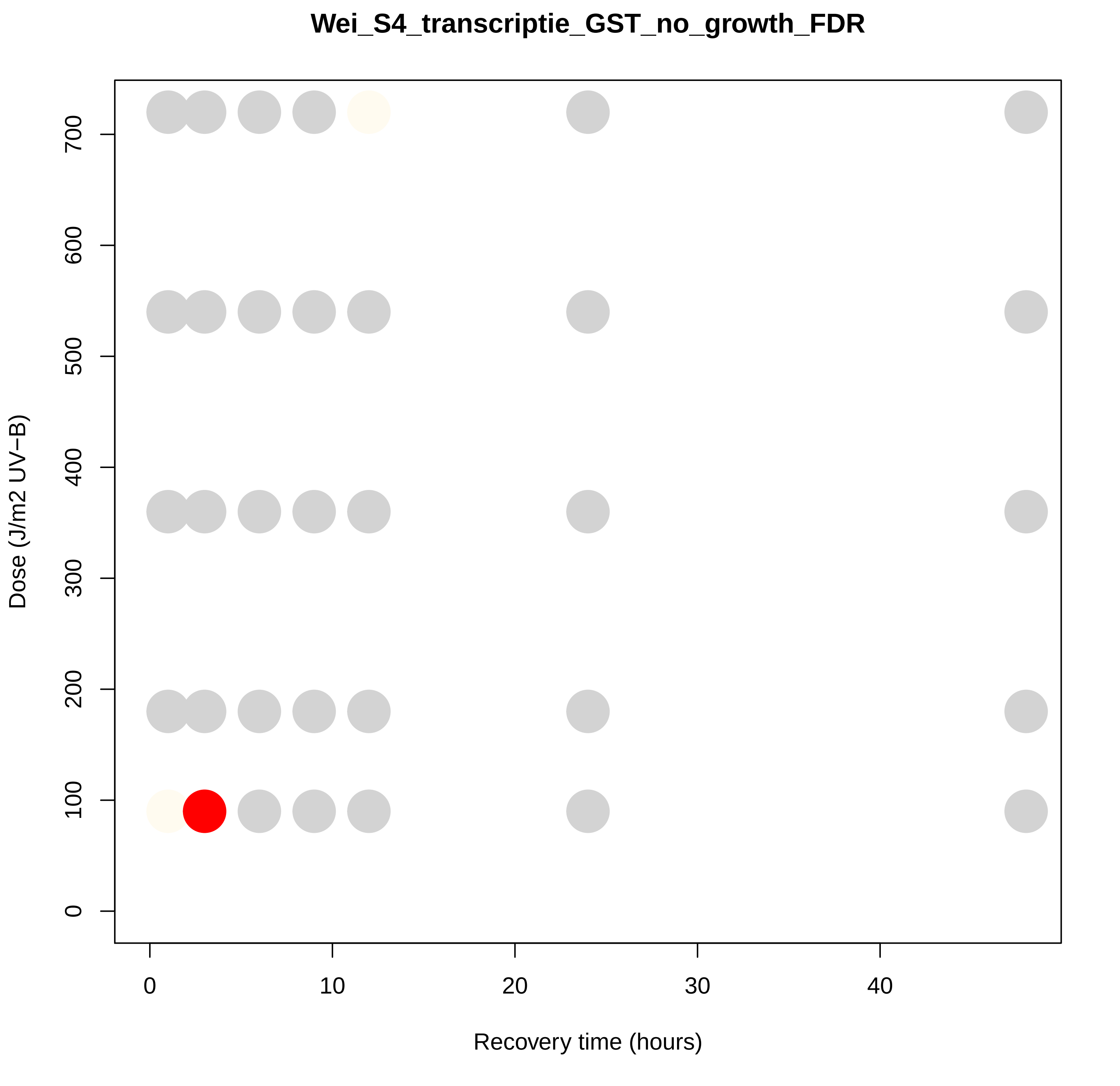
Wei_S4_transcriptie_GST_no_growth_FDR
Dose (J/m2 UV−B)
0
100
200
300
400
500
600
700
0
10
20
30
40
Recovery time (hours)

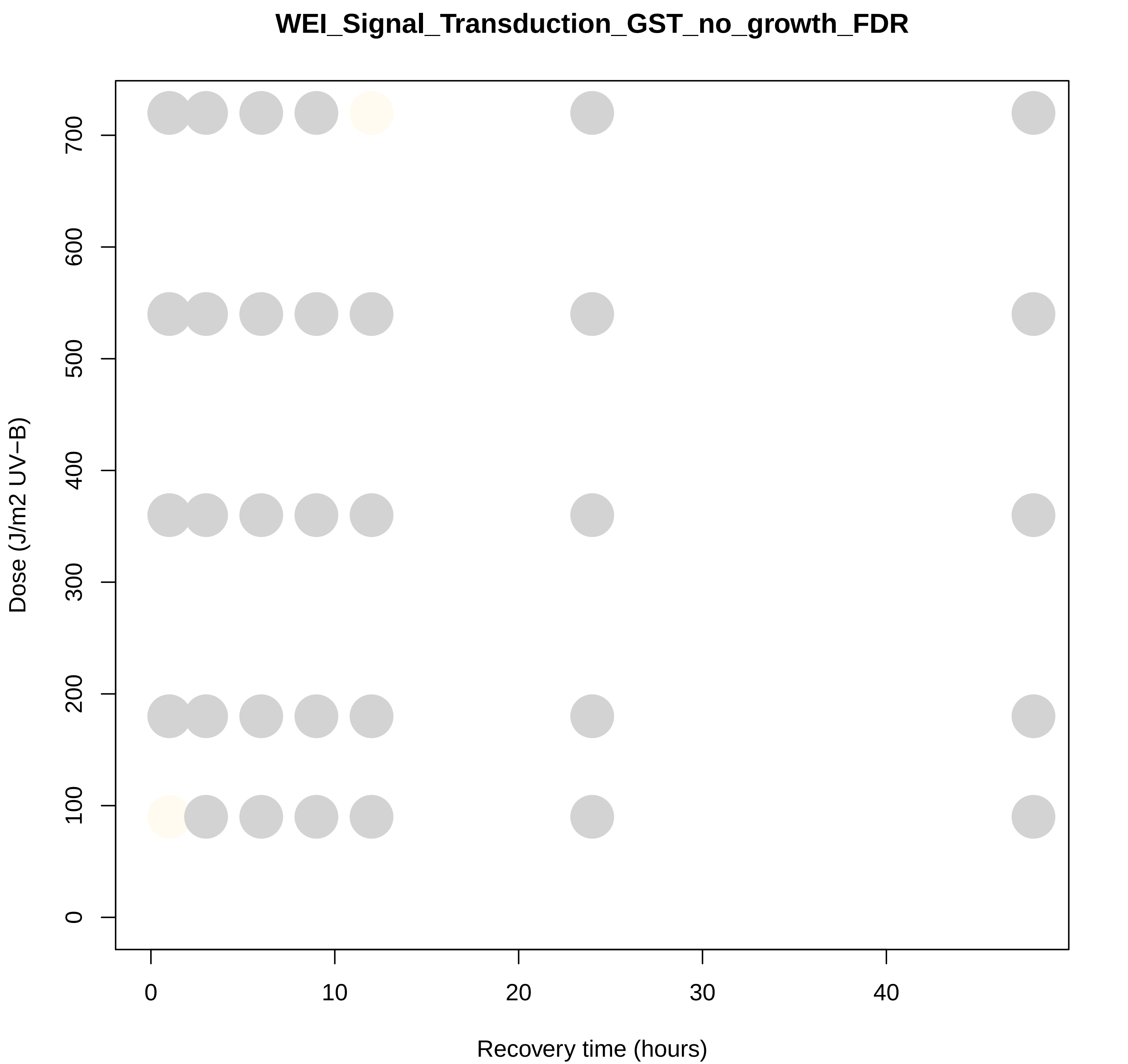
WEI_Signal_Transduction_GST_no_growth_FDR
Dose (J/m2 UV−B)
0
100
200
300
400
500
600
700
Recovery time (hours)
0
10
20
30
40

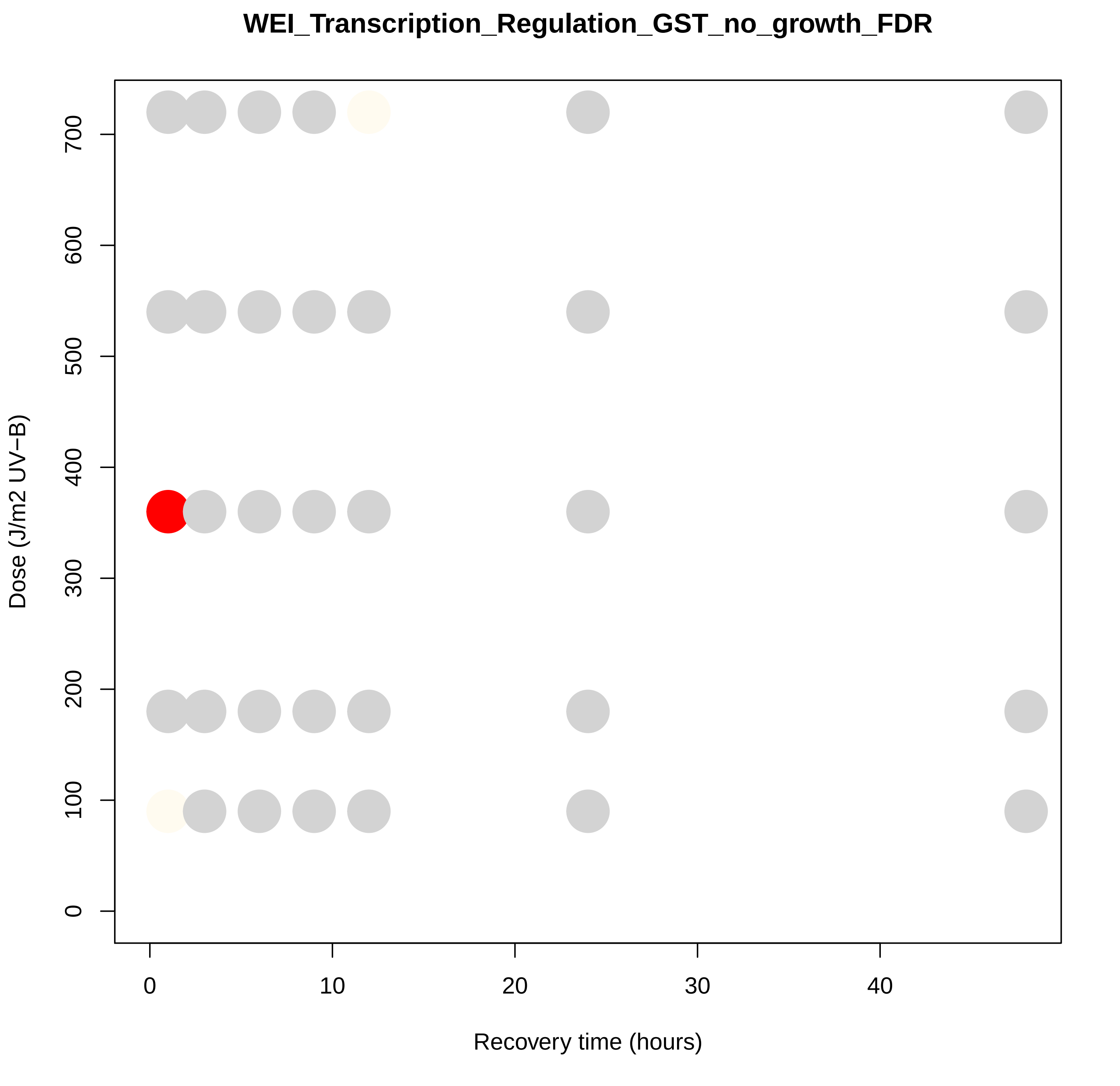
WEI_Transcription_Regulation_GST_no_growth_FDR
Dose (J/m2 UV−B)
Recovery time (hours)
0
100
200
300
400
500
600
700
0
10
20
30
40

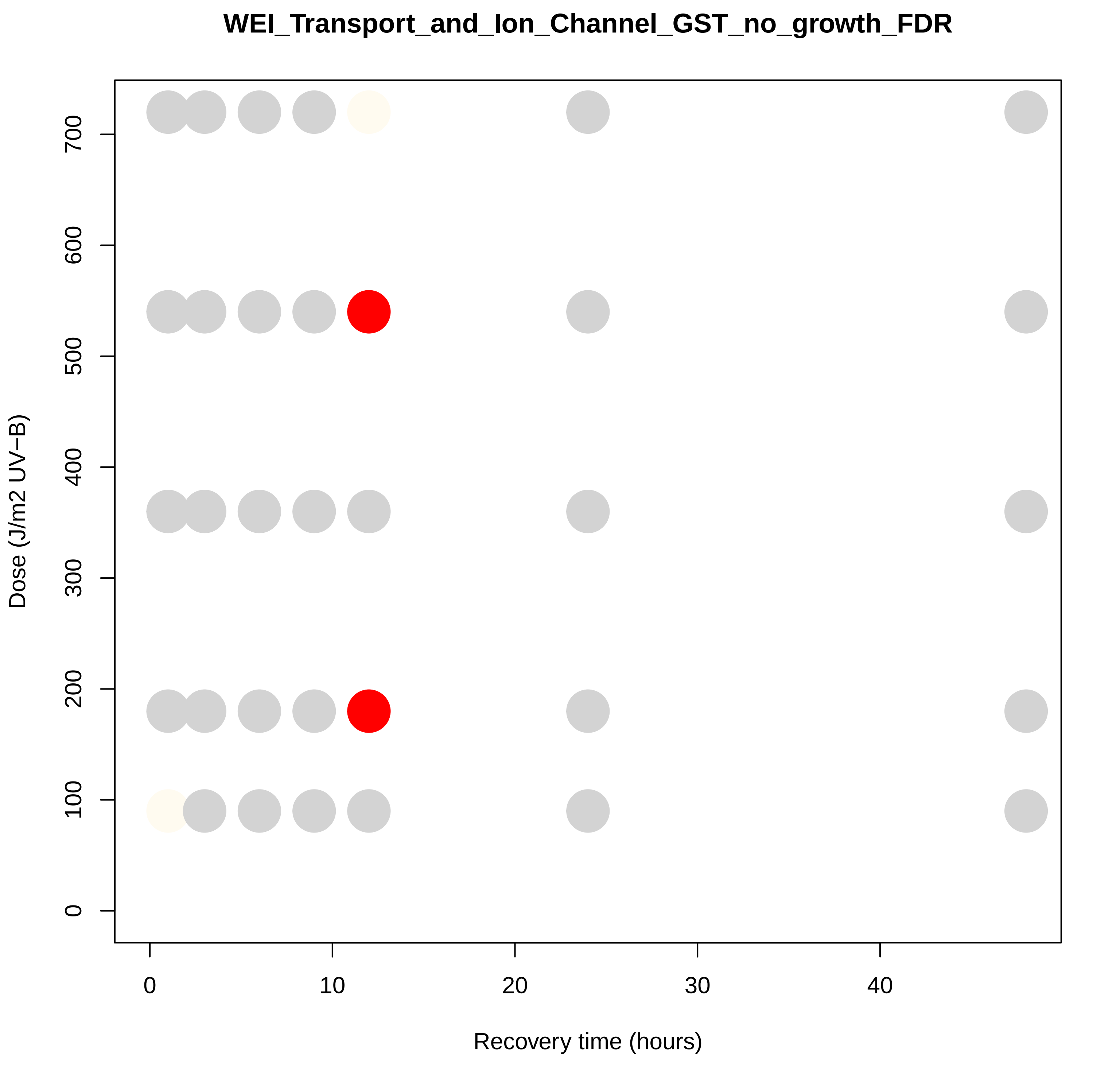

WEI_Transport_and_Ion_Channel_GST_no_growth_FDR
Dose (J/m2 UV−B)
Recovery time (hours)
0
100
200
300
400
500
600
700
0
10
20
30
40

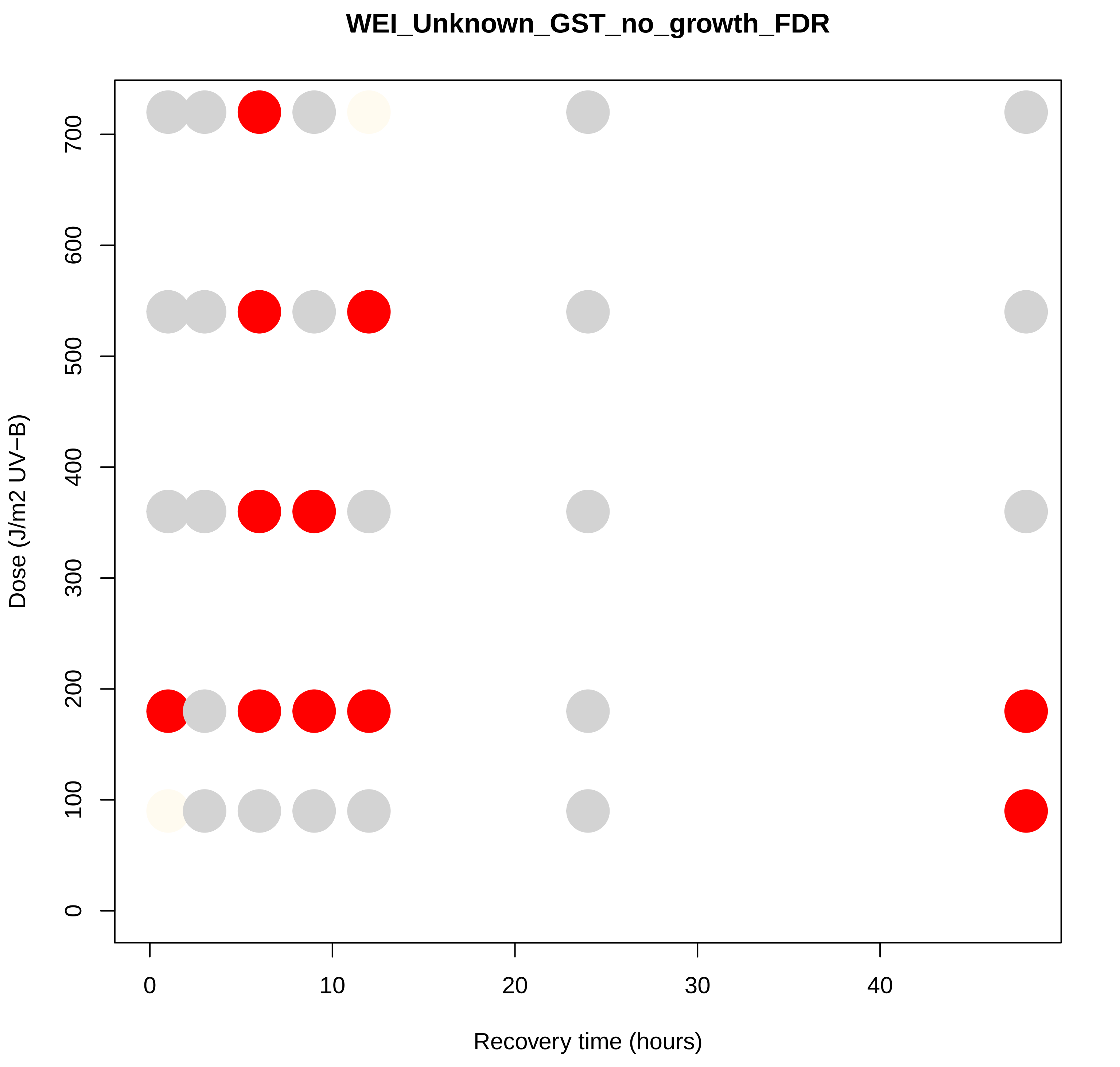

WEI_Unknown_GST_no_growth_FDR
Dose (J/m2 UV−B)
0
100
200
300
400
500
600
700
0
10
20
30
40
Recovery time (hours)

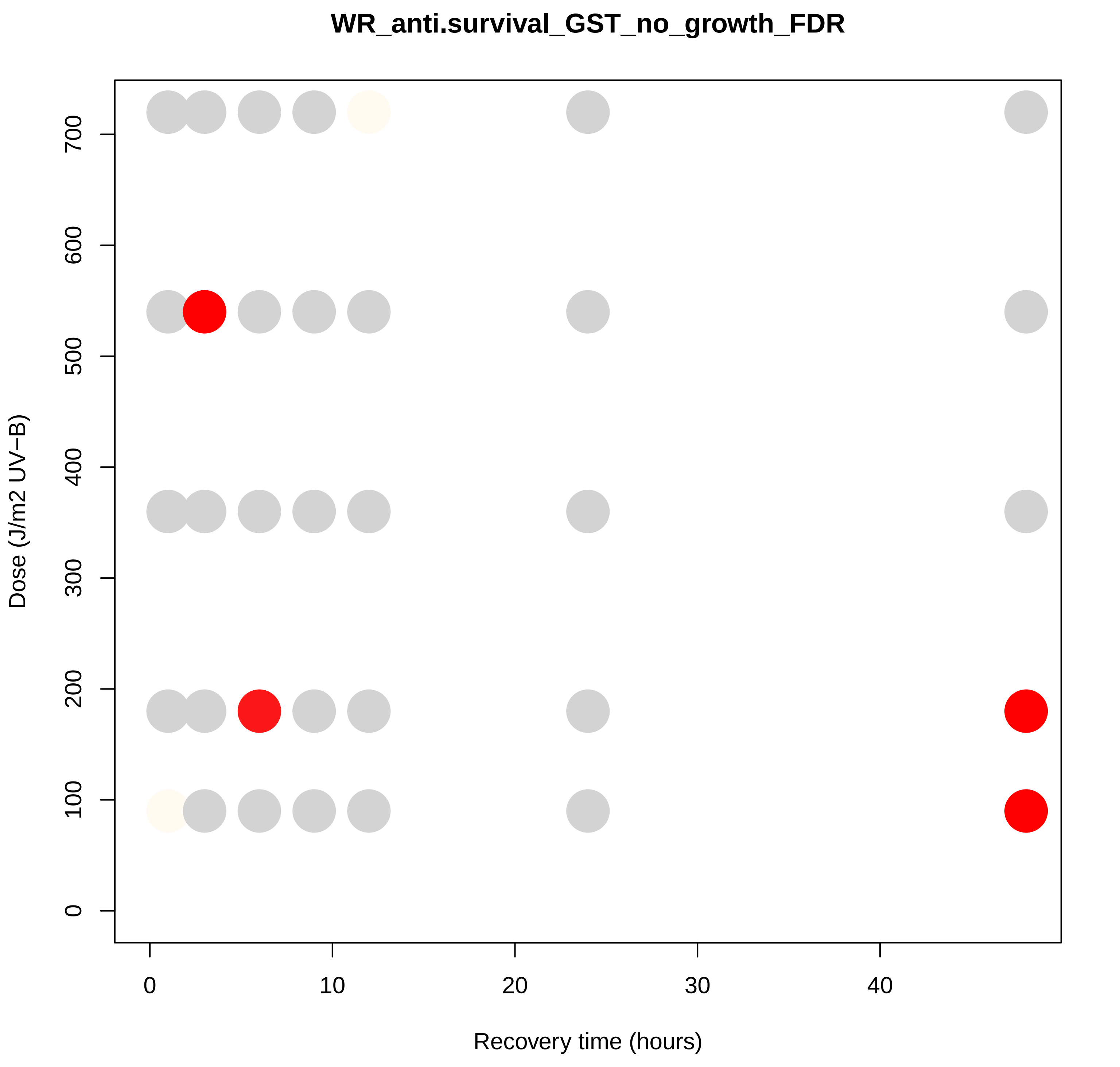
WR_anti.survival_GST_no_growth_FDR
Dose (J/m2 UV−B)
0
100
200
300
400
500
600
700
Recovery time (hours)
0
10
20
30
40

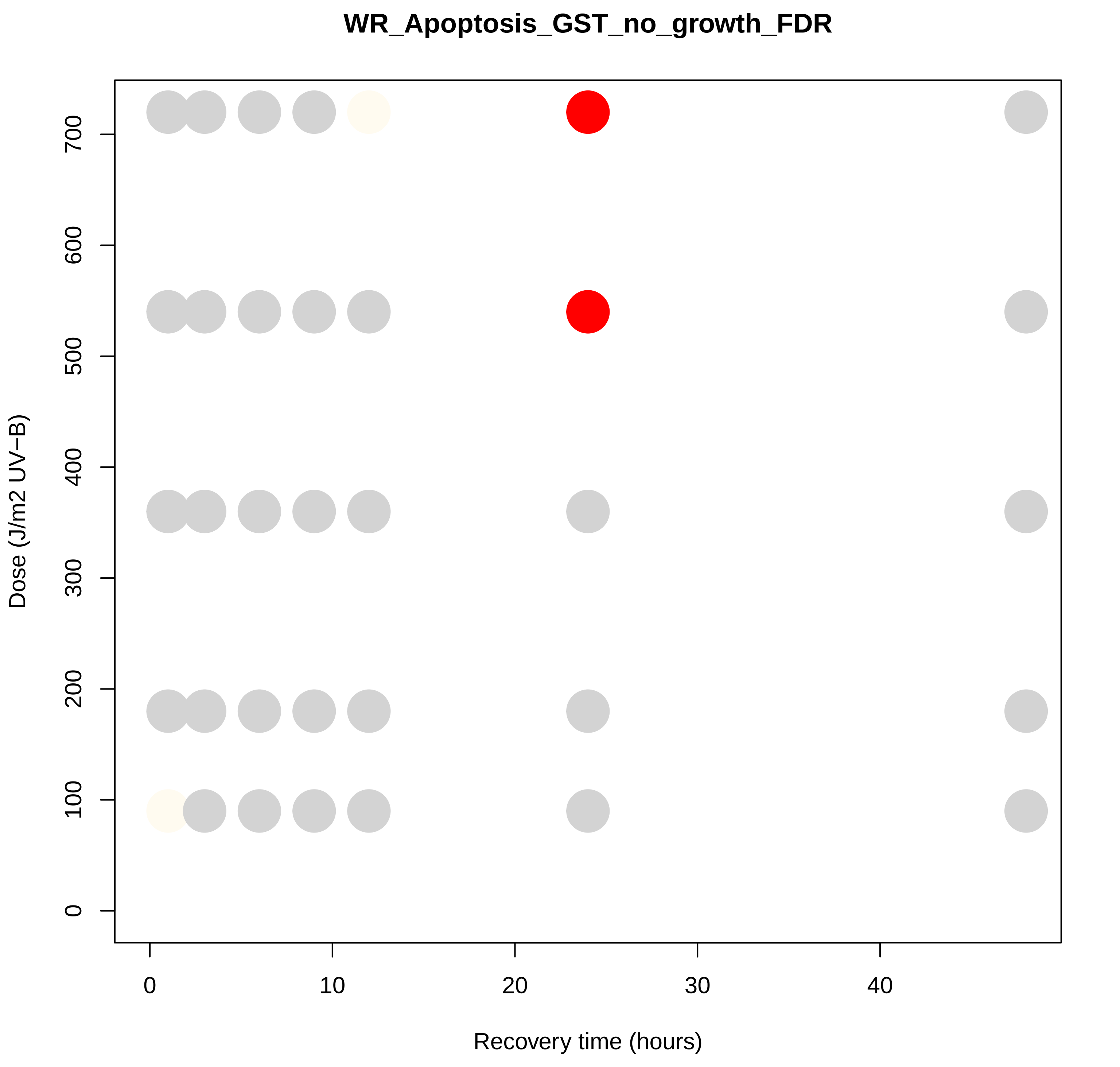
WR_Apoptosis_GST_no_growth_FDR
Dose (J/m2 UV−B)
Recovery time (hours)
0
100
200
300
400
500
600
700
0
10
20
30
40

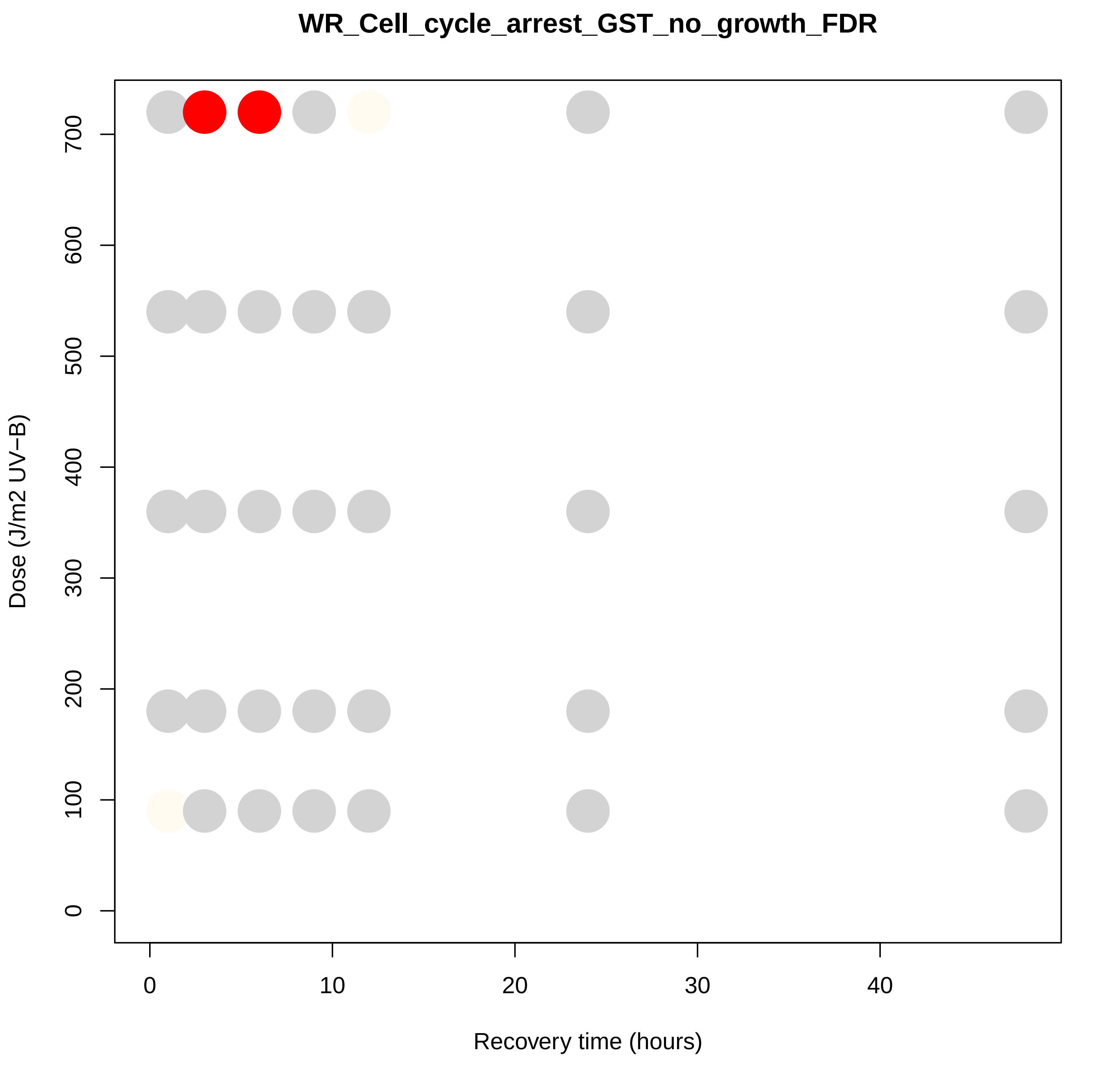

WR_Cell_cycle_arrest_GST_no_growth_FDR
Dose (J/m2 UV−B)
0
100
200
300
400
500
600
700
0
10
20
30
40
Recovery time (hours)

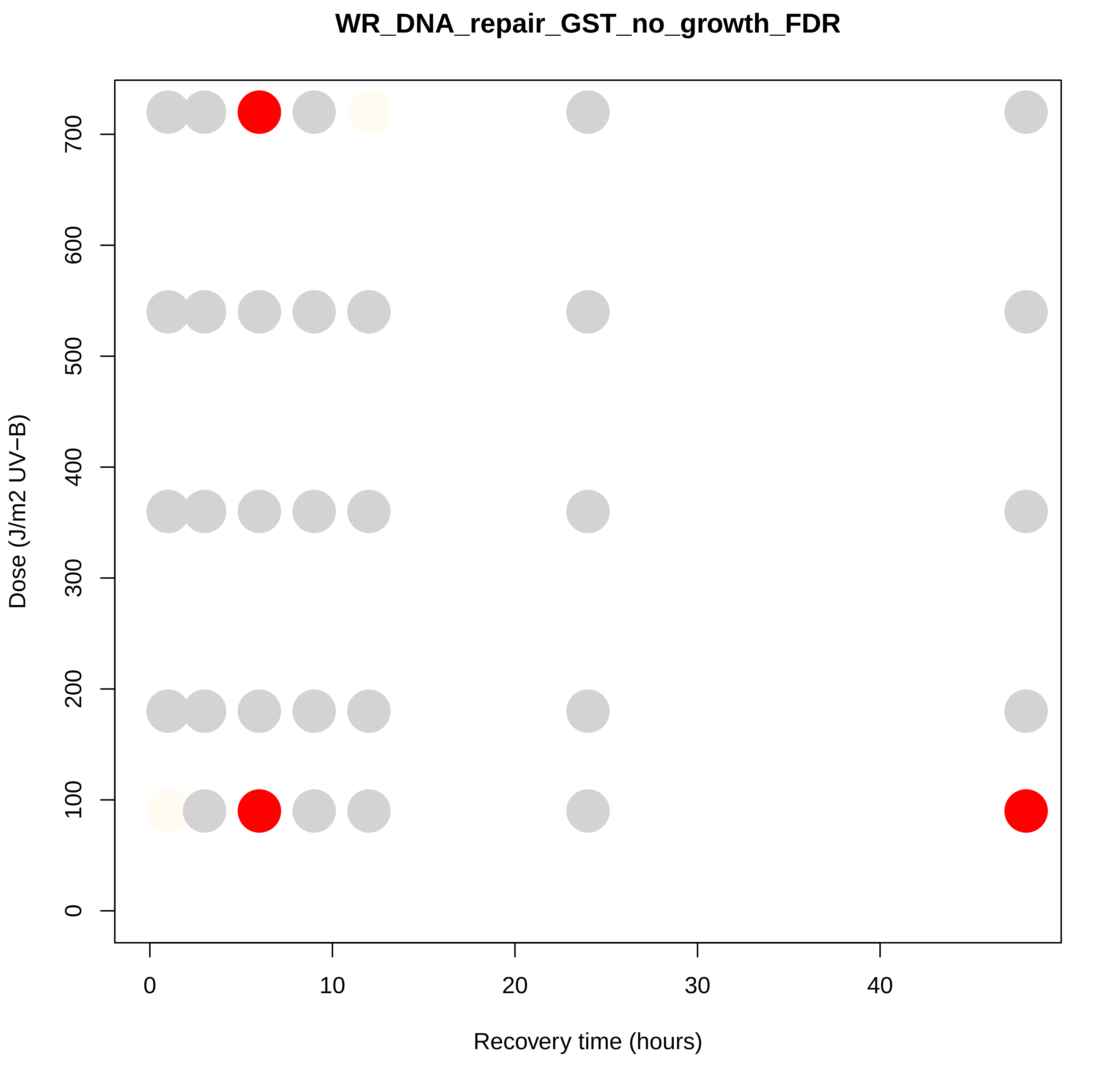
WR_DNA_repair_GST_no_growth_FDR
Dose (J/m2 UV−B)
0
100
200
300
400
500
600
700
0
10
20
30
40
Recovery time (hours)

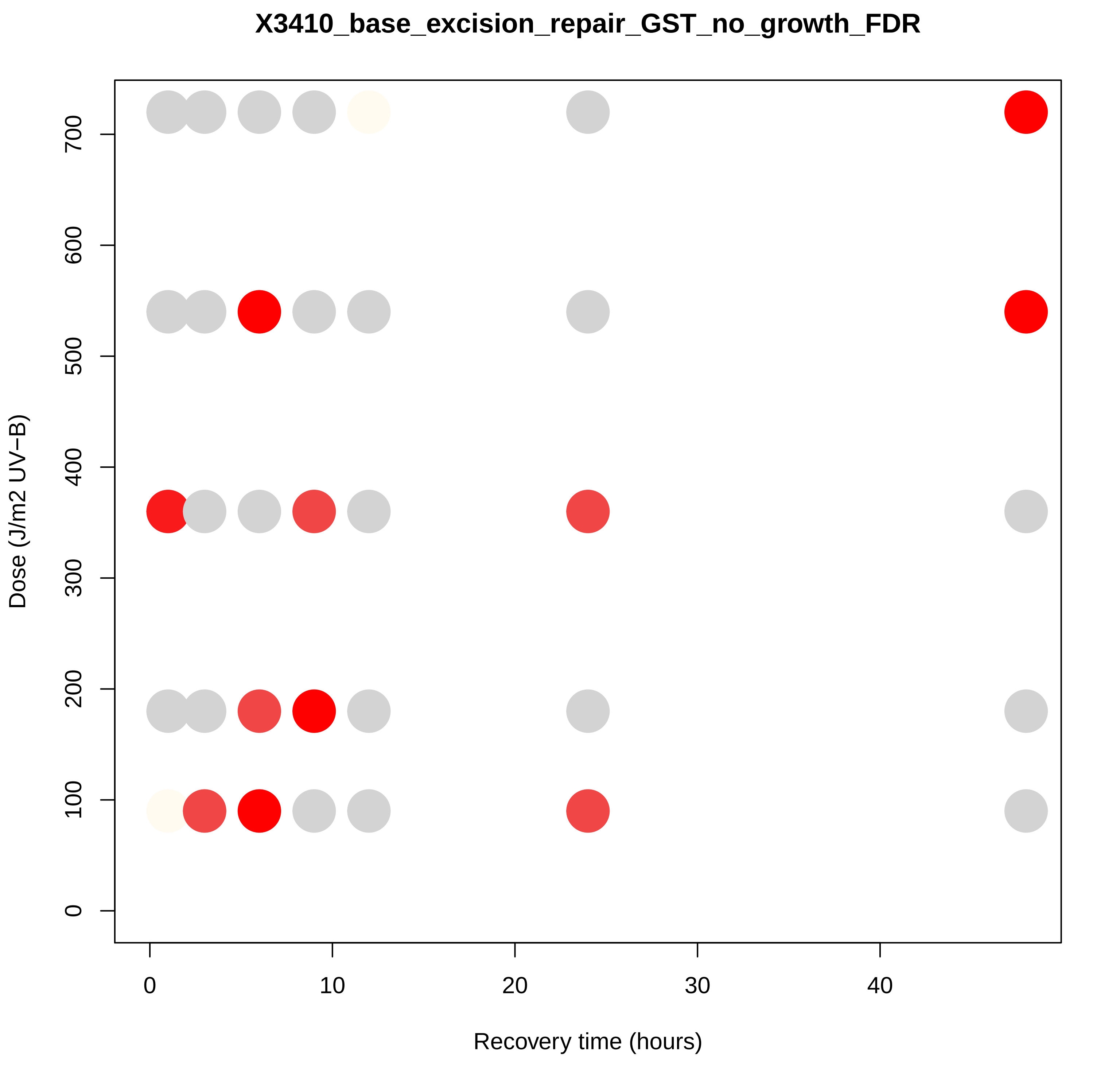
X3410_base_excision_repair_GST_no_growth_FDR
Dose (J/m2 UV−B)
0
100
200
300
400
500
600
700
0
10
20
30
40
Recovery time (hours)

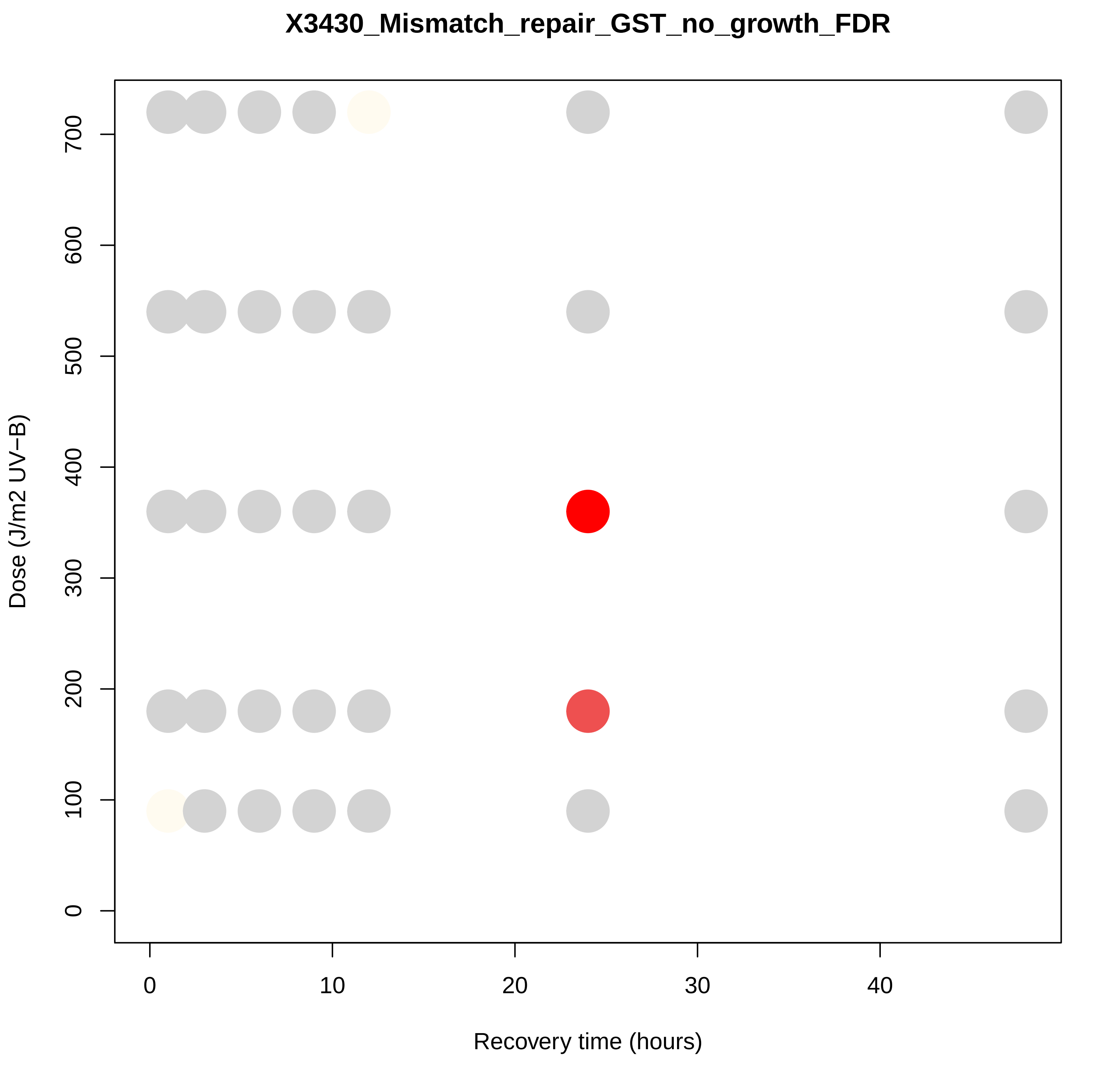

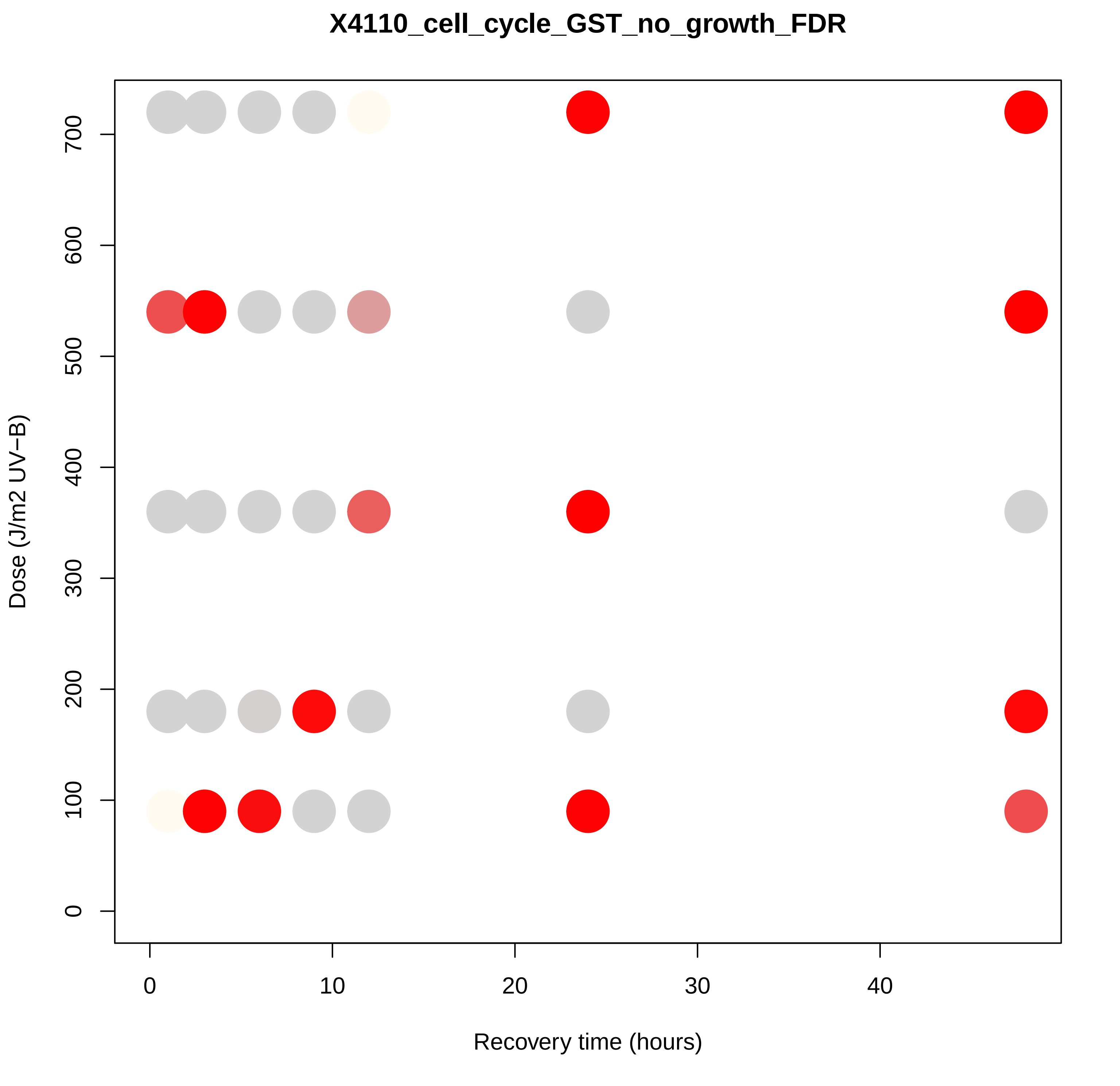
X4110_cell_cycle_GST_no_growth_FDR
Dose (J/m2 UV−B)
Recovery time (hours)
0
100
200
300
400
500
600
700
0
10
20
30
40

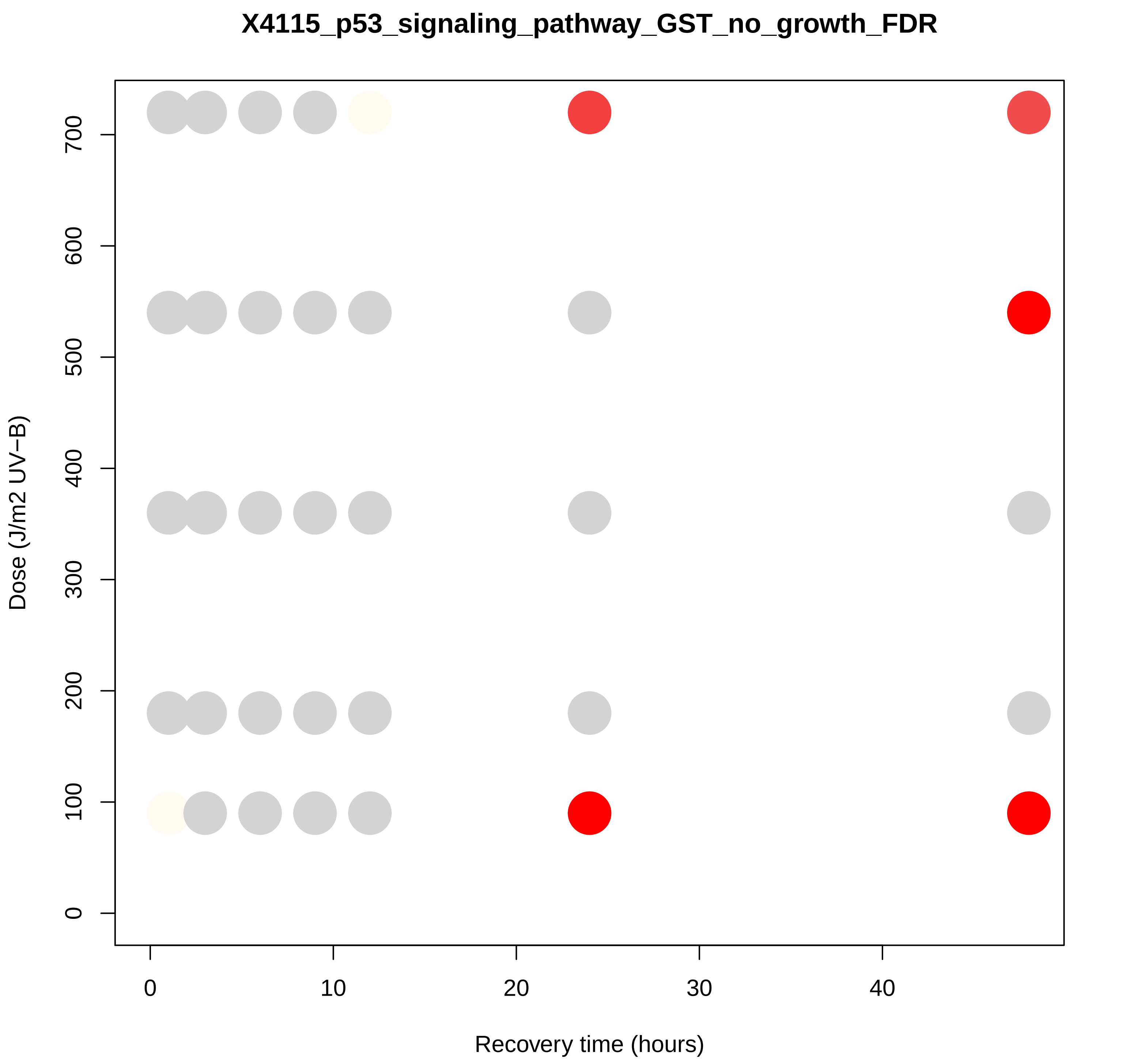
X4115_p53_signaling_pathway_GST_no_growth_FDR
Dose (J/m2 UV−B)
0
100
200
300
400
500
600
700
Recovery time (hours)
0
10
20
30
40

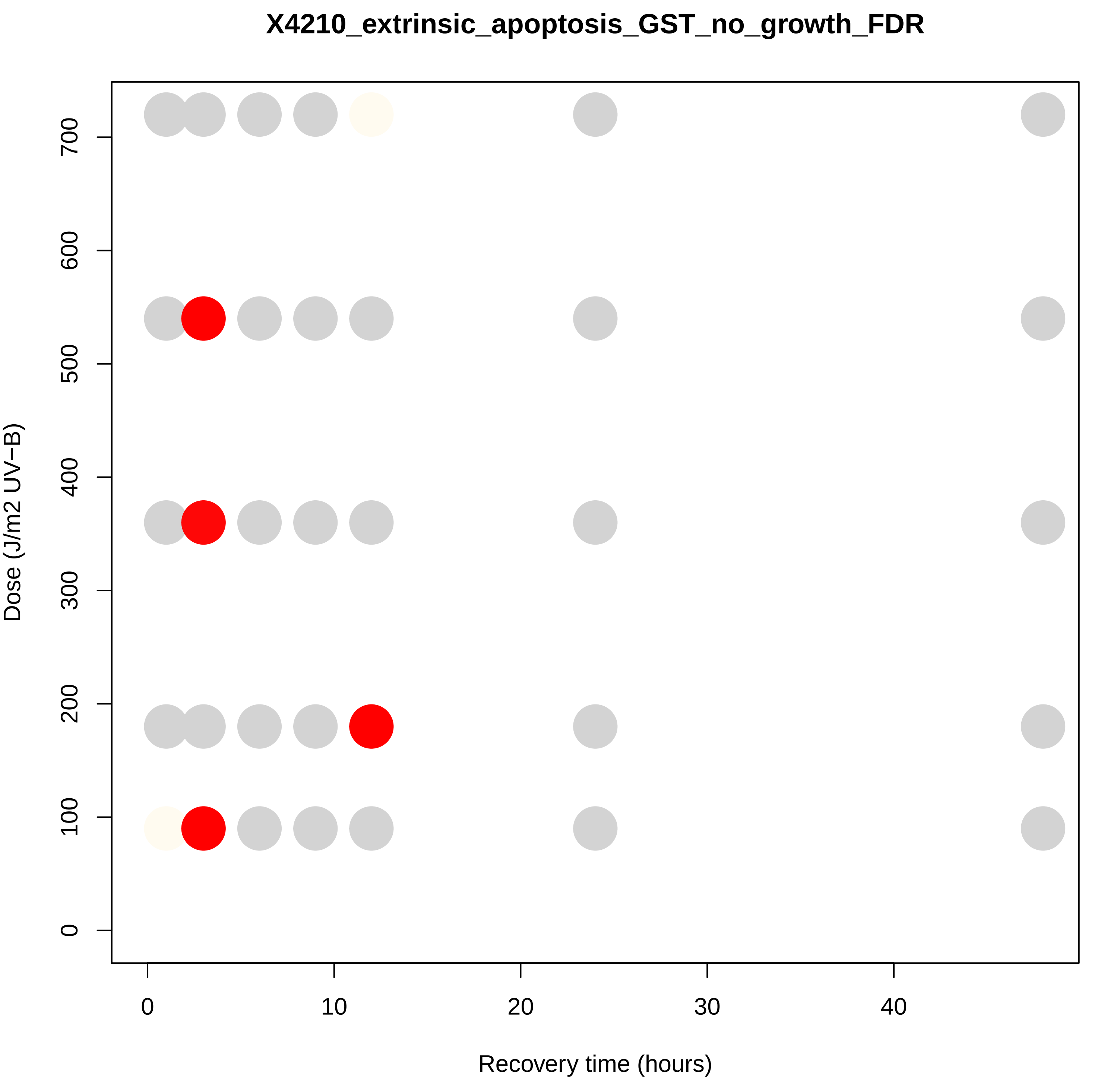
X4210_extrinsic_apoptosis_GST_no_growth_FDR
Dose (J/m2 UV−B)
0
100
200
300
400
500
600
700
Recovery time (hours)
0
10
20
30
40

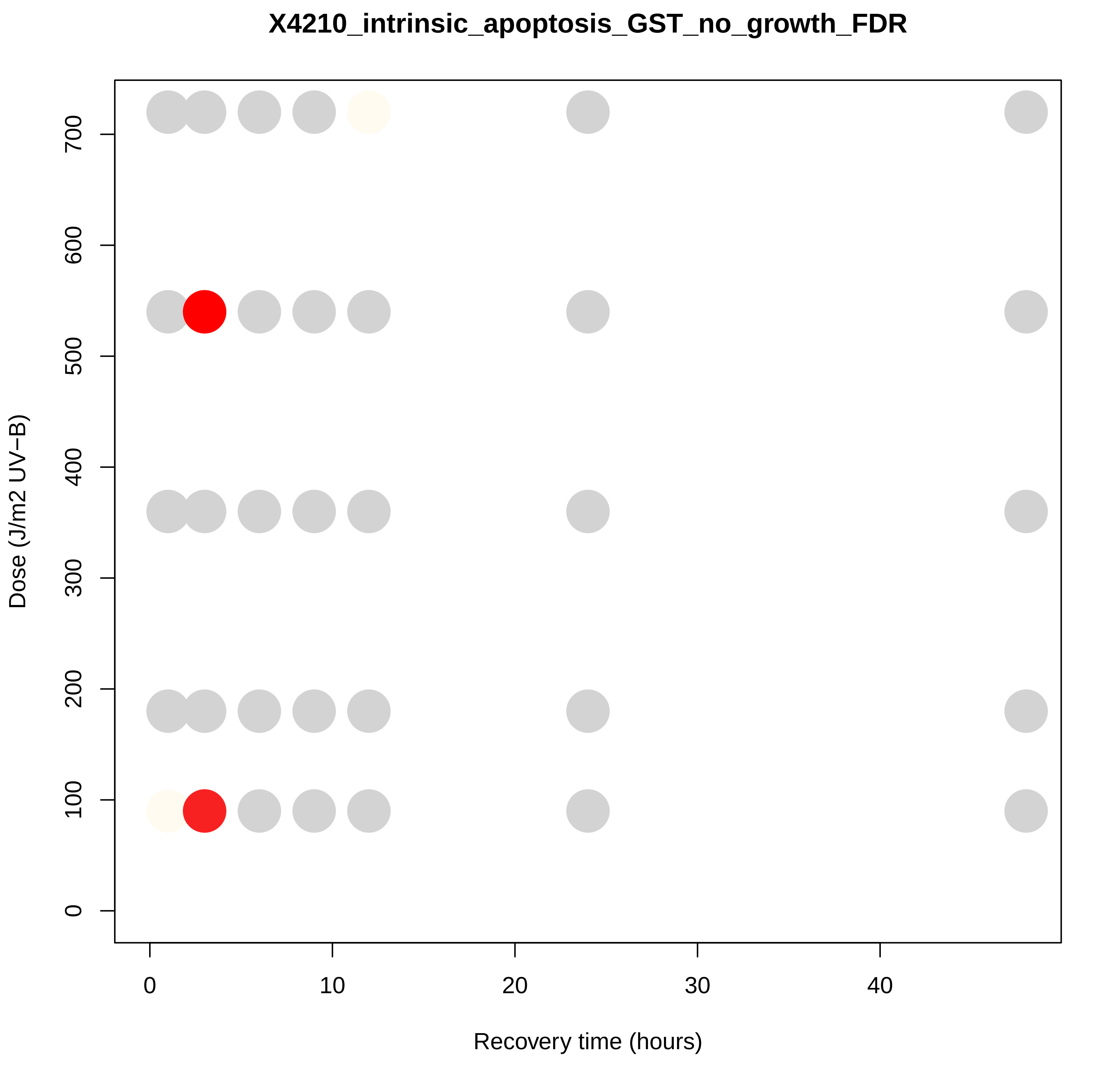
X4210_intrinsic_apoptosis_GST_no_growth_FDR
Dose (J/m2 UV−B)
0
100
200
300
400
500
600
700
0
10
20
30
40
Recovery time (hours)
