# Supplementary material for: A Range Finding Protocol to Support Design for Transcriptomics Experimentation: Examples of In-Vitro and In-Vivo Murine UV Exposure
Source: PLoS One. 2014 May 13;9(5):e97089. doi: 10.1371/journal.pone.0097089 (PMC4019648; doi:10.1371/journal.pone.0097089)
Supplement: Table S1 — RNA isolation metrics. Detailed RNA sample information for the in-vitro experiment (A) and the in-vivo experiment (B). (PDF) [file pone.0097089.s005.pdf]

Table S1. RNA isolation metrics

Detailed RNA sample information for the *in-vitro* experiment (A) and the *in-vivo* experiment (B).

A

In-vitro MEF exposure

Sample	Dose UV-C(J/m2)	Recovery time (hours)	rRNA (ng/µl)	RIN value	mRNA ng/ul	Array
36	0	0	40.05	10	695.61	448431A12
25	0	0.17	38.78	9.6	606.97	448431A01
19	0	0.5	42.25	9.6	629.11	447355A07
10	0	1	40.7	10	636.16	447333A10
11	0	3	43.9	10	787.2	447333A11
37	0	6	51.77	10	616.65	448536A01
20	0	12	70.37	9.8	592.6	447355A08
3	0	24	139.75	10	572.56	447333A03
46	0	48	231.13	9.5	412.83	448536A10
26	0.25	0.5	38.8	9.6	545.39	448431A02
45	0.25	1	45.85	9.9	770.15	448536A09
27	0.25	3	47.41	9.9	668.3	448431A03
43	0.25	6	39.04	10	573.75	448536A07
15	0.25	10	39.27	9.9	504.25	447355A03
4	0.25	12	74	10	571.87	447333A04
22	0.25	24	121.06	10	605.48	447355A10
5	0.25	48	263.89	10	622.86	447333A05
6	0.75	0.17	35.6	9.8	546.19	447333A06
48	0.75	0.5	41.99	9.3	455.29	448536A12
21	0.75	1	43.97	9.9	587.26	447355A09
47	0.75	3	49.29	10	637.41	448536A11
16	0.75	6	42.23	10	663.57	447355A04
31	0.75	12	68.47	10	606.73	448431A07
12	0.75	24	125.27	10	831.93	447333A12
34	0.75	48	274.03	10	614.49	448431A10
9	2.25	0.17	25.2	10	307.97	447333A09
40	2.25	0.5	44.01	10	747.89	448536A04
17	2.25	3	48.18	9.8	763.07	447355A05
30	2.25	6	53.82	9.9	570.79	448431A06
39	2.25	12	68.38	10	575.98	448536A03
13	2.25	24	128.85	10	717.27	447355A01
35	2.25	48	232.55	9.1	656.41	448431A11
29	6.7	0.17	39.93	9.7	518.55	448431A05
7	6.7	0.5	39.38	9.9	585.01	447333A07
2	6.7	1	41.18	10	784.67	447333A02
42	6.7	3	44.09	9.4	651.1	448536A06
28	6.7	6	57.23	9.4	558.46	448431A04
24	6.7	12	54.41	10	607.17	447355A12
44	6.7	24	115.51	10	587.45	448536A08
18	6.7	48	184.63	10	671.64	447355A06
41	20	0.17	41.53	10	570.63	448536A05
14	20	0.5	42.18	10	633.77	447355A02
38	20	1	44.91	9.6	539.92	448536A02
1	20	3	36.84	10	394.29	447333A01
8	20	6	47.66	9.3	527.46	447333A08
32	20	12	33.04	9.4	721.5	448431A08
33	20	24	48.81	10	645.08	448431A09
23	20	48	51.51	10	747.69	447355A11

B

In-vivo skin exposure

Sample	Mouse	Recovery time (hours)	Dose UV-B (J/m2)	rRNA ng/ul	RIN	mRNA ng/ul	Array
46	5	0	0	234.07	8.1	307.52	527442A01
22	5	3	0	79.58	7.4	274.96	527372A01
1	5	9	0	105.96	7.5	403.6	526839A01
19	5	24	0	141.92	7.3	350.88	527334A10
34	5	48	0	72.46	6.9	238	527374A01
27	6	0	0	122.78	8.2	165.84	527372A06
11	6	1	0	136.79	8.1	283.12	527334A02
42	6	6	0	215.69	7.7	328.48	527374A09
9	6	12	0	248.96	8.2	233.04	526839A09
52	6	48	0	129.96	7.4	177.2	527442A07
4	7	0	0	178.19	8.6	126.32	526839A04
12	7	3	90	121.41	7.4	172	527334A03
43	7	9	90	115.7	8.0	292.4	527374A10
55	7	24	90	155.61	8.7	424.88	527442A10
30	7	48	90	104.54	8.2	277.84	527372A09
29	8	0	0	117.99	9.0	108.16	527372A08
58	8	1	90	62.86	7.7	37.52	
20	8	6	90	157.97	7.9	419.76	527334A11
49	8	12	90	115.99	7.3	304.4	527442A04
40	8	48	90	92	7.8	322.56	527374A07
58	9	0	0	102.34	8.7	41.36	
25	9	3	180	191.56	7.9	281.68	527372A04
17	9	9	180	190.63	7.7	426.08	527334A08
39	9	24	180	117.45	8.3	374	527374A06
53	9	48	180	151.17	7.9	379.52	527442A08
47	10	0	0	139.22	8.7	328.64	527442A02
38	10	1	180	111.57	8.2	464.88	527374A05
32	10	6	180	118.51	7.2	424.08	527372A11
21	10	12	180	84.21	6.8	317.04	527334A12
3	10	48	180	85.98	7.0	307.36	526839A03
10	11	0	0	179.86	9.0	325.2	527334A01
44	11	3	360	142.98	8.8	334	527374A11
23	11	9	360	162.81	7.5	350.32	527372A02
8	11	24	360	146.81	8.7	287.04	526839A08
50	11	48	360	167.58	8.0	384.96	527442A05
35	12	0	0	85.9	8.4	293.6	527374A02
54	12	1	360	111.46	7.8	301.76	527442A09
5	12	6	360	99.52	7.5	352.32	526839A05
28	12	12	360	111.96	7.6	391.44	527372A07
16	12	48	360	121.4	6.8	304.56	527334A07
6	13	0	0	119.79	8.1	172.32	526839A06
48	13	3	540	139.17	7.0	321.12	527442A03
45	13	9	540	108.84	7.0	444.16	527374A12
26	13	24	540	128.71	8.2	381.12	527372A05
13	13	48	540	235.67	8.7	318.64	527334A04
18	14	0	0	113.11	8.3	152.24	527334A09
2	14	1	540	327.78	8.9	267.84	526839A02
51	14	6	540	125.14	7.6	383.92	527442A06
41	14	12	540	103.24	7.7	384.16	527374A08
31	14	48	540	116.9	7.1	305.12	527372A10
14	15	0	0	172.74	8.5	210.72	527334A05
7	15	3	720	141.55	8.2	293.28	526839A07
56	15	9	720	99.31	8.6	417.68	527442A11
24	15	24	720	137.65	7.7	412.8	527372A03
37	15	48	720	197.32	8.0	380.32	527374A04
36	16	0	0	178.48	8.3	335.12	527374A03
33	16	1	720	126.91	7.9	405.84	527372A12
57	16	6	720	125.67	7.6	383.52	527442A12
58	16	12	720	63.31	7.0	47.12	
15	16	48	720	209.9	7.4	274.32	527334A06
