# Supplementary material for: A Range Finding Protocol to Support Design for Transcriptomics Experimentation: Examples of In-Vitro and In-Vivo Murine UV Exposure
Source: PLoS One. 2014 May 13;9(5):e97089. doi: 10.1371/journal.pone.0097089 (PMC4019648; doi:10.1371/journal.pone.0097089)
Supplement: Table S2 — Differentially expressed genes. The numbers of differentially expressed genes (DEGs, log2 FC>1) found in both experiments, if compared to time-point 0 (A & B, Figure 3A & B); after additional removal of genes that were differentially expressed in any untreated sample (C & D, Figure 3C & D); after re-calculation using the associated dose = 0 sample (log2 FC>1) (E & F, Figure 3E & F). Excluded samples are indicated in grey. (PDF) [file pone.0097089.s006.pdf]

Table_S2. Differentially expressed genes

The numbers of differentially expressed genes (DEGs, log2 FC > 1) found in both experiments, if compared to time-point 0 (A & B, Figure 3A & B); after additional removal of genes that were differentially expressed in any untreated sample (C & D, Figure 3C & D); after re-calculation using the associated dose=0 sample (log2 FC > 1) (E & F, Figure 3E & F). Excluded samples are indicated in grey.

## *In-vitro* MEF exposure

### A

| | 0.17 | 0.5 | 1 | 3 | 6 | 12 | 24 | 48 | union | intersect |
|---|---|---|---|---|---|---|---|---|---|---|
| 20 | 410 | 406 | 750 | 2802 | 3411 | 3919 | 3156 | 2817 | 1120 (8839) | 95 (22) |
| 6.7 | 200 | 238 | 541 | 1944 | 1809 | 2195 | 2846 | 2105 | 3344 (5962) | 30 (22) |
| 2.25 | 626 | 534 | | 637 | 1000 | 2418 | 2344 | 2085 | 2078 (4763) | 44 (27) |
| 0.75 | 434 | 839 | 344 | 773 | 969 | 1999 | 2127 | 2021 | 2543 (4974) | 26 (23) |
| 0.25 | 694 | 288 | 384 | 460 | 912 | 2203 | 2221 | 2241 | 1955 (4796) | 31 (24) |
| 0 | 275 | 343 | 313 | 434 | 1194 | 1988 | 4179 | 2462 | 1854 (6288) | 30 (22) |
| Union | 1817 | 1806 | 1448 | 2751 | 2984 | | | | | |
| | | | | 4559 | 5007 | 6155 | 6969 | 4956 | | |
| Intersect | 28 | 36 | 55 | 112 | 319 | | | | | |
| | | | | 75 | 221 | 612 | 846 | 865 | | |

### C

| | 0.17 | 0.5 | 1 | 3 | 6 | 12 | 24 | 48 | union | intersect |
|---|---|---|---|---|---|---|---|---|---|---|
| 20 | 133 | 144 | 363 | | | | | | 556 | 7 |
| 6.7 | 42 | 82 | 282 | 1333 | 1193 | | | | 2319 | 1 |
| 2.25 | 303 | 179 | | 299 | 386 | | | | 1042 | 0 |
| 0.75 | 182 | 630 | 126 | 338 | 290 | | | | 1448 | 0 |
| 0.25 | 504 | 134 | 125 | 114 | 222 | | | | 967 | 0 |
| Union | 1042 | 1044 | 724 | 1727 | 1625 | | | | | |
| intersect | 0 | 0 | 2 | 7 | 27 | | | | | |

### E

| | 0.17 | 0.5 | 1 | 3 | 6 | 12 | 24 | 48 | union | intersect |
|---|---|---|---|---|---|---|---|---|---|---|
| 20 | 142 | 163 | 240 | | | | | | 498 | 3 |
| 6.7 | 81 | 143 | 150 | 1169 | 983 | | | | 2098 | 1 |
| 2.25 | 353 | 118 | | 307 | 195 | | | | 901 | 0 |
| 0.75 | 178 | 737 | 177 | 270 | 107 | | | | 1345 | 0 |
| 0.25 | 561 | 200 | 136 | 88 | 86 | | | | 978 | 0 |
| Union | 1140 | 1153 | 613 | 1502 | 1204 | | | | | |
| intersect | 0 | 0 | 0 | 0 | 0 | | | | | |

## *In-vivo* skin exposure

### B

| | 1 | 3 | 6 | 9 | 12 | 24 | 48 | union | intersect |
|---|---|---|---|---|---|---|---|---|---|
| 720 | 618 | 472 | 892 | 957 | | 1169 | 654 | 2441 | 67 |
| 540 | 1153 | 1198 | 986 | 1285 | 775 | 1353 | 881 | 3494 | 46 |
| 360 | 247 | 192 | 695 | 531 | 570 | 357 | 748 | 1689 | 5 |
| 180 | 325 | 986 | 604 | 939 | 1015 | 508 | 520 | 2170 | 68 |
| 90 | | 466 | 643 | 442 | 711 | 375 | 468 | 1674 | 66 |
| 0 | 198 | 494 | 553 | 479 | 795 | 341 | 461 | 1814 | 14 |
| Union | 1811 | 2452 | 2203 | 2735 | 2052 | 2287 | 1786 | | |
| intersect | 16 | 4 | 154 | 30 | 148 | 65 | 80 | | |

### D

| | 1 | 3 | 6 | 9 | 12 | 24 | 48 | union | intersect |
|---|---|---|---|---|---|---|---|---|---|
| 720 | 172 | 271 | 365 | 551 | | 715 | 164 | 1471 | 5 |
| 540 | 648 | 587 | 429 | 742 | 323 | 681 | 452 | 2247 | 7 |
| 360 | 96 | 51 | 229 | 171 | 149 | 77 | 272 | 787 | 0 |
| 180 | 106 | 485 | 218 | 472 | 395 | 187 | 127 | 1177 | 20 |
| 90 | | 255 | 265 | 124 | 183 | 144 | 89 | 824 | 0 |
| Union | 906 | 1343 | 1150 | 1545 | 880 | 1296 | 816 | | |
| intersect | 5 | 0 | 1 | 6 | 2 | 2 | 2 | | |

### F

| | 1 | 3 | 6 | 9 | 12 | 24 | 48 | union | intersect |
|---|---|---|---|---|---|---|---|---|---|
| 720 | 386 | 512 | 562 | 835 | | 877 | 176 | 2364 | 2 |
| 540 | 1057 | 684 | 405 | 1460 | 398 | 673 | 355 | 3371 | 3 |
| 360 | 215 | 520 | 441 | 358 | 235 | 162 | 138 | 1665 | 0 |
| 180 | 161 | 294 | 314 | 621 | 434 | 323 | 119 | 1521 | 12 |
| 90 | | 776 | 566 | 634 | 411 | 288 | 154 | 2155 | 1 |
| Union | 1553 | 1988 | 1613 | 2513 | 1172 | 1547 | 723 | | |
| intersect | 0 | 0 | 0 | 0 | 0 | 0 | 0 | | |
