# Supplementary material for: A Range Finding Protocol to Support Design for Transcriptomics Experimentation: Examples of In-Vitro and In-Vivo Murine UV Exposure
Source: PLoS One. 2014 May 13;9(5):e97089. doi: 10.1371/journal.pone.0097089 (PMC4019648; doi:10.1371/journal.pone.0097089)
Supplement: Table S3 — In-vitro examples of dose-response correlations of individual genes per time point. Dose response correlations per time point of individual genes that belong to gene sets: KEGG nucleotide excision repair, KEGG cell cycle, KEGG extrinsic apoptosis and IARC p53 responsive elements, in the in-vitro experiment (set up as Table 1). (PDF) [file pone.0097089.s007.pdf]

Table_S3. *In-vitro* examples of dose-response correlations of individual genes per time point

Dose response correlations per time point of individual genes that belong to gene sets: KEGG nucleotide excision repair, KEGG cell cycle, KEGG extrinsic apoptosis and IARC p53 responsive elements, in the *in-vitro* experiment (set up as Table 1).

Nucleotide Excision Repair					
gene	0.17h	0.5h	1h	3h	6h
Rfc3		0.82			0.8
Ercc2			-0.93		
Pole3			0.88	0.95	
Cetn2			0.88		0.94
Pold3			-0.85		
Pold4			0.81		
Mnat1				-0.97	-0.86
Xpa				0.88	
Rbx1				0.87	
Gtf2h2				-0.85	
Ercc5				0.82	0.88
Gtf2h1				0.82	
Rfc4					0.91
Rfc5					0.89
Pold2					-0.88
Rad23b					0.88
Ercc4					0.87
Ercc8					0.85
Gtf2h5					0.83
Ercc1					-0.82
Xpc					0.82

(grey shading) location of the sweet spot

Cell Cycle					
gene	0.17h	0.5h	1h	3h	6h
Gsk3b	-0.93			-0.86	
Mcm4	-0.92				0.89
Mdm2	-0.87			0.9	0.88
Cdkn2c	0.84				
Ywhae	-0.8				-0.91
Cdk6		0.95		-0.96	
Gadd45a		0.92	0.99		-0.92
Atm		0.92			0.95
Cdc25a		-0.83	-0.85		0.84
Anapc1		0.83			
Espl1		-0.82		0.97	
Tgfb2			0.97	-0.95	
Ccnb1			0.97	-0.91	
Cdkn2a			0.93		0.94
Cdc27			-0.91		
Smad2			-0.9		0.8
Gadd45g			-0.88	-0.83	-0.96
Cdc20			-0.87	-0.82	
Wee1			-0.87		0.93
Smad3			-0.86	-0.93	
Ep300			-0.86		
Anapc7			-0.86		
Abl1			-0.85		
Cdc25c			0.84		
Gm5593				-1	0.82
Stag1				-0.97	-0.81
Bub1b				-0.97	
Cdc14a				-0.96	-0.87
Stag2				-0.96	
Tfdp2				-0.96	
Mad1l1				-0.94	
Cdc7				0.93	0.89
Atr				-0.92	
Rb1				-0.91	-0.83
Cdc25b				-0.91	
Ccna2				-0.9	
Cdc14b				-0.89	
Dbf4				0.89	
Anapc11				0.89	
Cdkn1a				0.88	0.91
Cdkn1c				0.87	
Rbx1				0.87	
Mad2l2				0.86	0.81
Ccnb2				0.85	
Chek1				0.83	0.98
Anapc10				-0.81	
Skp2					0.95
Tgfb3					-0.94
Orc3l					-0.93
Anapc5					-0.9
Cdk4					0.89
Gadd45b					-0.88
Ywhab					-0.88
Skp1a					0.87
Anapc13					0.87
E2f2					0.86
Ccnd1					0.84
Cdkn2b					0.84
Cdkn2d					-0.82
Prkdc					-0.82
Orc6l					0.82
Myc					-0.81

Extrinsic Apoptosis					
gene	0.17h	0.5h	1h	3h	6h
Apaf1	-0.92				
Il1b	0.91				0.83
Tnf		0.92	0.9	0.82	
Atm		0.92			0.95
Pik3r5		0.92			-0.85
Irak2		-0.9			
Prkar2a		-0.9			
Tradd			0.96		
Traf2			-0.95		0.9
Capn2			-0.92		
Nfkb1			-0.91		
Chuk			-0.89		
Cflar			-0.88	0.92	0.81
Akt3			-0.87	-0.97	-0.88
Bcl2l1			-0.85		
Tnfsf10			0.81		
Casp9			0.8		0.95
Fadd			0.8		
Birc7				1	0.82
Casp3				0.96	0.95
Prkar1b				-0.91	-0.87
1500003O03Rik				0.89	
Pik3cb				-0.88	
Prkx				0.87	0.92
Ppp3cb				-0.87	
Ppp3cc				-0.83	-0.94
Cycs				0.82	0.85
Pik3cg				-0.81	
Tnfrsf10b					0.95
Casp12					0.94
Casp6					0.88
Ripk1					0.88
Ppp3ca					-0.87
Prkacb					-0.84
Akt1					-0.83
Prkar2b					-0.82
Irak1					-0.8

p53 Responsive Elements					
gene	0.17h	0.5h	1h	3h	6h
Mdm2	-0.87			0.9	0.88
Ccng1			0.95	0.85	0.84
Lrdd				0.99	0.8
Cdkn1a				0.88	0.91
Tnfrsf10b					0.95
Gadd45b					-0.88
