# Supplementary material for: A Range Finding Protocol to Support Design for Transcriptomics Experimentation: Examples of In-Vitro and In-Vivo Murine UV Exposure
Source: PLoS One. 2014 May 13;9(5):e97089. doi: 10.1371/journal.pone.0097089 (PMC4019648; doi:10.1371/journal.pone.0097089)
Supplement: Table S4 — In-vivo examples of dose-response correlations of individual genes per time point. Dose response correlations per time point of individual genes that belong to gene sets: KEGG nucleotide excision repair, KEGG cell cycle, KEGG extrinsic apoptosis and IARC p53 responsive elements, in the in-vivo experiment (set up as Table 2). (PDF) [file pone.0097089.s008.pdf]

Table S4. *In-vivo* examples of dose-response correlations of individual genes per time point

Dose response correlations per time point of individual genes that belong to gene sets: KEGG nucleotide excision repair, KEGG cell cycle, KEGG extrinsic apoptosis and IARC p53 responsive elements, in the in-vivo experiment (set up as Table 2).

Nucleotide Excision Repair							
gene	1h	3h	6h	9h	12h	24h	48h
Pole3	-0.97			0.97	0.97	0.92	
Pold4		0.89	0.91				
Mnat1		-0.87	-0.95				
Pole4		0.86	0.93	0.85			
Ercc1		0.86		0.82	0.81		
Ercc6			0.96				
Rfc5			0.9	-0.8	0.95		0.94
Ercc5			0.88	0.81			
Rfc1			-0.86				0.85
Ccnh			-0.84			0.95	
Ddb2				-0.96			
Xpc				-0.93			
Gtf2h3				0.88			
Rfc3				0.87	0.98		0.92
Pcna				0.81	0.86		
Cdk7				0.81			
Xpa					-1		
Pole2					0.99		
Gm9840					0.97	0.92	
Gtf2h1					0.95	0.87	
Gtf2h5					0.92	0.9	
Rfc2					0.91		0.84
Rpa2					0.91		0.82
Lig1					0.85		0.81
Rfc4					0.84		
Pole					0.8		0.92
Rbx1					0.8		
Ddb1							0.88
Ercc2							0.86
Pold3							0.84
Rpa1							0.81

location of the sweet spot

Cell Cycle							
gene	1h	3h	6h	9h	12h	24h	48h
Ccnd3	1						
E2f3	-0.91						
Rb1	-0.9		-0.91				
Mad2l2	0.88						0.93
Cdc25c	-0.86						0.8
Ccnb2	-0.85		-0.86				
Fzr1	0.83		0.86				
Tgfb1	0.81		-0.84				
Smad3	0.81						
Pkmyt1		0.98					0.94
Smc3		-0.97				-0.88	
Cdc14b		-0.96	-0.88	-0.81	-0.91		
E2f5		0.93					
Myc		0.91					
Anapc7		0.88			0.91		
Anapc4		-0.86					
Gadd45g		0.83					
Cdc14a		0.82				-0.81	
Tgfb3			-0.93				0.83
Mdm2			0.92	0.89	0.8	0.88	
Cdkn1a			0.91	0.86	0.95	0.94	0.85
Chek2			-0.89		0.9		
Stag2			-0.88				
Atr			-0.86		-0.94		
Mad2l1			-0.85		0.96		
Ccna2			-0.85				0.88
Cdkn2d			-0.85				
Bub1b			-0.84	-0.9			
Ccnh			-0.84			0.95	
Ccnd2			-0.83				
Cdk6			-0.83			0.85	
Cdc20			-0.82		0.8		0.92
Cdc25b			-0.81				
Cdc7			0.8		0.93		0.94
Stag1			-0.8				
Rbl2				-0.97		-0.86	
Bub1				-0.91			0.86
Rbl1				-0.91			0.92
Tfdp2				-0.89		-0.85	
Ywhab				0.85		0.85	
Cdc16				0.82			
Pcna				0.81	0.86		
Cdk7				0.81			
Mcm4					0.98		0.86
Gm9840					0.97	0.92	
Ccnd1					0.95		
Cdkn1c					-0.95		
Gm2423					0.94		
Ccnb3					-0.94		
Mcm7					0.93		0.94
Dbf4					0.92		0.84
Cdc23					0.92		
Cdc2a					0.91		0.87
Ywhaq					0.91		
Bub3					0.89		0.88
Cdc45l					0.89		0.95
Ep300					0.88	0.89	
Prkdc					-0.85	-0.86	
Orc6l					0.85		
Ywhag					0.84		
Orc3l					-0.84		
Orc2l					0.83		
Cdkn2b					0.82		
Orc1l					0.82		0.88
Orc4l					0.82	0.82	
Rbx1					0.8		
E2f4						0.81	
Ccne2							0.84
Mcm3							0.94
Mcm2							0.85
Mcm5							0.9
Tfdp1							0.8
Tgfb2						-0.92	
Trp53						0.88	
Pttg1							0.85
Sfn						0.86	
Anapc11							0.82

Extrinsic Apoptosis							
gene	1h	3h	6h	9h	12h	24h	48h
Irak4	0.93						
Il1rap	-0.91	-0.82	-0.9	-0.85	-0.8		
Akt3	0.89				-0.92		
Ppp3cc	-0.87		-0.82				
Akt2	0.86						
Pik3cb	-0.83	-0.83		-0.85	-0.92	-0.8	
Prkar1b	0.83						
Csf2rb	0.82						
Bcl2	-0.81		-0.87			-0.94	
Irak2		-0.93					
Ngf		0.9	0.9	0.81	0.99	0.84	
Dffa		-0.87	-0.91		-0.88		
Prkacb		-0.87	-0.85			-0.9	
Bid		0.87					
Pik3cd		-0.87					
Prkar1a		0.85		0.9			
Fas		-0.85					
Tnfsf10		-0.83					
Pik3r5		-0.8				0.81	
Tnfrsf10b			0.9	0.89	0.86	0.89	
Myd88			0.9			0.82	
Pik3ca			-0.9				
Ppp3ca			-0.89	-0.89	-0.98		
Pik3r2			-0.87				
Nfkb1			-0.85				
Tradd			0.84		0.85		
Birc7				-0.85			
Il1a					-0.87		
Traf2					0.85		
Fasl					0.83		
Ppp3r1					0.83		
2010110P09Rik					-0.82		
Chuk							-0.8
Csf2rb2						0.85	
Dffb						0.92	
Ntrk1						0.85	
Trp53						0.88	
1500003O03Rik							0.92

p53 Responsive Elements							
gene	1h	3h	6h	9h	12h	24h	48h
Msh2	-0.97						
Btg2	0.95						
Igfbp3	-0.87	-0.86		-0.8	-0.86	-0.86	
Sesn1		-0.98		-0.87	-1		
S100a2		0.94					
Pmaip1		0.87	0.81		0.97		
Fas		-0.85					
Mdm2			0.92	0.89	0.8	0.88	
Cdkn1a			0.91	0.86	0.95	0.94	0.85
Gml			0.91				
Tnfrsf10b			0.9	0.89	0.86	0.89	
Pcna				0.81	0.86		
Sfn						0.86	
