# Supplementary material for: A Range Finding Protocol to Support Design for Transcriptomics Experimentation: Examples of In-Vitro and In-Vivo Murine UV Exposure
Source: PLoS One. 2014 May 13;9(5):e97089. doi: 10.1371/journal.pone.0097089 (PMC4019648; doi:10.1371/journal.pone.0097089)
Supplement: Data S1 — Multiple longitudinal biopsies sampling in individual mice. (PDF) [file pone.0097089.s010.pdf]

Data S1. Multiple longitudinal biopsies sampling in individual mice

A study to determine the maximum number of biopsies in a mouse model.

Setup: four mice were biopsied, each with a maximum of 6 (A1-A). RNA from each sample was used in a standard microarray-based gene-expression analysis. Standard bioinformatics including Principal component analysis (PCA), EDGE analysis (http://www.genomine.org/edge) and Overrepresentation analyses were performed to examine the data.

Results: The mice underwent the biopsy sampling without any visible inconvenience. The animals did not show signs of irritation, scratching or biting around the areas of sampling until 48 hours after sampling of the first biopsy. PCA of microarray-based gene expression signals showed no significant variation between animals and time (A1-B). Gene expression analysis identified 169 differentially expressed genes (fdr-corrected p-value < 0.1). Overrepresentation analysis of these DEGs showed 26 biological processes being significantly affected in the response to the treatment (A1-C).

Conclusions: As no regulation of wound healing or immunological processes were present in the significant biological processes – processes that are likely to be caused by previous biopsies –, plus significant regulation was observed for the Circadian rhythm pathways, – a process that can be expected during sampling over time –, we concluded that biopsy sampling using several longitudinal samples in one animal – with a maximum of six biopsies per animal – over time is suitable for researching gene-expression in skin.

Data: The data from this study is available through GEO Series accession number GSE51347.

A

B

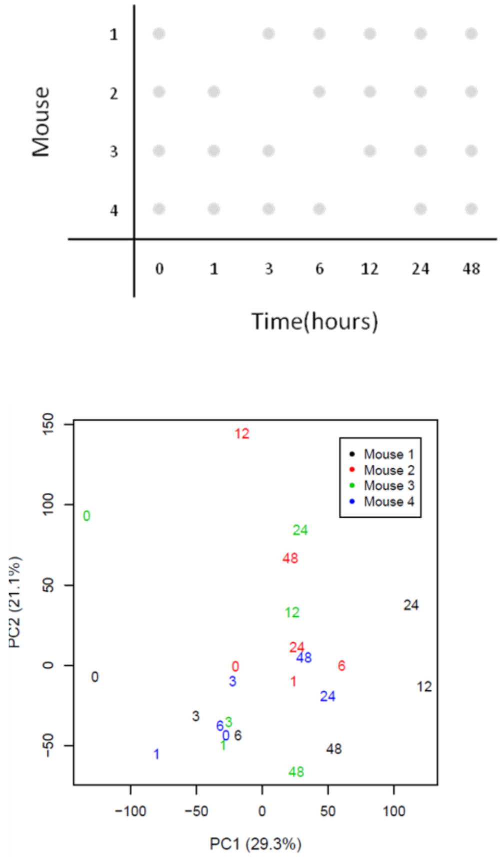

C

Significant biological processes ($p<0.05$)

Biological process	P-Value
detoxification of copper ion	3.55E-07
transcription	3.71E-05
regulation of transcription, DNA-dependent	9.55E-06
**rhythmic process**	1.53E-04
nitric oxide mediated signal transduction	5.05E-05
**circadian rhythm**	9.16E-04
cellular zinc ion homeostasis	7.43E-04
transcription termination	7.43E-04
cellular copper ion homeostasis	0.002
inter-male aggressive behavior	0.007
copper ion export	0.007
mating behavior, sex discrimination	0.007
regulation of RNA export from nucleus	0.007
regulation of oxidative phosphorylation	0.007
copper ion import	0.007
activation of phospholipase C	0.007
epinephrine metabolic process	0.007
negative regulation of metalloenzyme activity	0.007
retroviral genome replication	0.007
basal protein localization	0.007
establishment or maintenance of actin cytoskeleton polarity	0.007
synaptic vesicle priming	0.007
elastin biosynthetic process	0.007
territorial aggressive behavior	0.007
peptidyl-lysine modification	0.028
